# The Impact of Protein Architecture on Adaptive Evolution

**DOI:** 10.1093/molbev/msz134

**Published:** 2019-05-30

**Authors:** Ana Filipa Moutinho, Fernanda Fontes Trancoso, Julien Yann Dutheil

**Affiliations:** 1 Department of Evolutionary Genetics, Max Planck Institute for Evolutionary Biology, Plön, Germany; 2 Unité Mixte de Recherche 5554 Institut des Sciences de l’Evolution, CNRS, IRD, EPHE, Université de Montpellier, Montpellier, France

**Keywords:** protein structure, protein function, adaptation, population genetics, *Drosophila melanogaster*, *Arabidopsis thaliana*

## Abstract

Adaptive mutations play an important role in molecular evolution. However, the frequency and nature of these mutations at the intramolecular level are poorly understood. To address this, we analyzed the impact of protein architecture on the rate of adaptive substitutions, aiming to understand how protein biophysics influences fitness and adaptation. Using *Drosophila melanogaster* and *Arabidopsis thaliana* population genomics data, we fitted models of distribution of fitness effects and estimated the rate of adaptive amino-acid substitutions both at the protein and amino-acid residue level. We performed a comprehensive analysis covering genome, gene, and protein structure, by exploring a multitude of factors with a plausible impact on the rate of adaptive evolution, such as intron number, protein length, secondary structure, relative solvent accessibility, intrinsic protein disorder, chaperone affinity, gene expression, protein function, and protein–protein interactions. We found that the relative solvent accessibility is a major determinant of adaptive evolution, with most adaptive mutations occurring at the surface of proteins. Moreover, we observe that the rate of adaptive substitutions differs between protein functional classes, with genes encoding for protein biosynthesis and degradation signaling exhibiting the fastest rates of protein adaptation. Overall, our results suggest that adaptive evolution in proteins is mainly driven by intermolecular interactions, with host–pathogen coevolution likely playing a major role.

## Introduction

A long-standing focus in the study of molecular evolution is the role of natural selection in protein evolution (Eyre-Walker 2006). One can measure the strength and direction of selection at the divergence level through the $d_{N}/d_{S}$ ratio (ω). However, because ω represents a summary statistic across nucleotide sites, it can only provide the average trend, while proteins will typically undergo both negative and positive selection. Branch-site models address this issue by fitting phylogenetic models with heterogeneous $d_{N}/d_{S}$ ratio among codons and branches, thus considering the great heterogeneity in selective constraints among sites, both in space and time (Nielsen and Yang 1998; Yang et al. 2005; Zhang et al. 2005). Although these methods potentially allow studying adaptation at the site level, they require large amounts of data across species and are therefore restricted to more conserved genes along the phylogeny. Conversely, the McDonald and Kreitman (MK) test (McDonald and Kreitman 1991) is applied at the population level and it only requires data from two closely related species, usually several individuals from the study species and one individual from the other. Because adaptive mutations contribute relatively more to substitution than to polymorphism, the MK test disentangles positive and negative selection by contrasting the number of substitutions to the number of polymorphisms at synonymous and nonsynonymous sites. Charlesworth (1994) extended this method to estimate the proportion of substitutions that is adaptive (α). Yet, one limitation of this approach was that it did not account for the segregation of slightly deleterious mutations, which can either over- or underestimate measurements of α according to the demography of the population (Eyre-Walker 2002; Smith and Eyre-Walker 2002). Recent methods solved this issue by taking into consideration the distribution of fitness effects (DFE) of both slightly deleterious (Fay et al. 2001; Smith and Eyre-Walker 2002; Bierne and Eyre-Walker 2004; Eyre-Walker et al. 2006; Eyre-Walker and Keightley 2009; Stoletzki and Eyre-Walker 2011) and slightly beneficial mutations (Galtier 2016; Tataru et al. 2017). By allowing the estimation of the rate of nonadaptive ($\omega_{\mathrm{na}}=\hat{d_{N}^{\mathrm{na}}}/d_{S}$) and adaptive ($\omega_{a}=\omega -\omega_{\mathrm{na}}$) nonsynonymous substitutions, in addition to measurements of α ($\omega_{a}/\omega$), these methods triggered new insights on the impact of both negative and positive selection on the rate of protein evolution.

Several studies have reported substantial levels of adaptive protein evolution in various animal species, including the fruit fly (Smith and Eyre-Walker 2002; Sawyer et al. 2003; Bierne and Eyre-Walker 2004; Haddrill et al. 2010), the wild mouse (Halligan et al. 2010), and the European rabbit (Carneiro et al. 2012), but also in bacteria (Charlesworth and Eyre-Walker 2006) and in plants (Ingvarsson 2010; Slotte et al. 2010; Strasburg et al. 2011). Whereas for other taxa, such as primates (Boyko et al. 2008; Hvilsom et al. 2012; Galtier 2016) and many other plants (Gossmann et al. 2010), the rate of adaptive mutations was observed to be very low, wherein amino-acid substitutions are expected to be nearly neutral and fixed mainly through random genetic drift (Boyko et al. 2008). Several authors proposed that this across-species variation in the molecular adaptive rate is explained by an effective population size ($N_{e}$) effect, where higher rates of adaptive evolution are observed for species with larger $N_{e}$ due to a lower impact of genetic drift (Eyre-Walker 2006; Eyre-Walker and Keightley 2009; Gossmann et al. 2012). Galtier (2016), however, reported that $N_{e}$ had an impact on α and $\omega_{\mathrm{na}}$but not $\omega_{a}$. Hence, he proposed that the relationship with $N_{e}$ is mainly explained by deleterious effects, wherein slightly deleterious nonsynonymous substitutions accumulate at lower rates in large-$N_{e}$ species due to the higher efficiency of purifying selection, thus decreasing $\omega_{\mathrm{na}}$ and consequently inflating α.

The rate of adaptive substitutions, however, was observed to vary extensively along the genome. On a genome-wide scale, it was reported that $\omega_{a}$ correlates positively with both the recombination and mutation rates, but negatively with gene density (Campos et al. 2014; Castellano et al. 2016). When looking at the gene level, previous studies have demonstrated the role of protein function in the rate of adaptive evolution, wherein genes involved in immune defense mechanisms appear with higher rates of adaptive mutations in Drosophila (Sackton et al. 2007; Obbard et al. 2009), humans, and chimpanzees (Nielsen et al. 2005). In Drosophila, sex-related genes also display higher levels of adaptive evolution, being directly linked with species differentiation (Pröschel et al. 2006; Haerty et al. 2007). At the intragenic level, however, the factors impacting the frequency and nature of adaptive mutations remain poorly understood.

There are several structural factors that have been reported to influence the rate of protein evolution but have not been investigated at the population level. Molecular evolution studies of protein families revealed that protein structure, for instance, significantly impacts the rate of amino-acid substitutions, with exposed residues evolving faster than buried ones (Liberles et al. 2012). As a stable conformation is often required to ensure proper protein function, mutations that impair the stability or the structural conformation of the folded protein are more likely to be counter-selected. Moreover, distinct sites in a protein sequence differ in the extent of conformational change they endure upon mutation, a pattern generally well predicted by the relative solvent accessibility (RSA) of a residue (Goldman et al. 1998; Mirny and Shakhnovich 1999; Franzosa and Xia 2009). In this way, residues at the core of proteins evolve slower than the ones at the surface due to their role in maintaining a stable protein structure (Perutz et al. 1965; Overington et al. 1992; Goldman et al. 1998; Bustamante et al. 2000; Dean et al. 2002; Choi et al. 2006; Lin et al. 2007; Conant and Stadler 2009; Franzosa and Xia 2009; Ramsey et al. 2011). Interspecific comparative sequence analyses also revealed that positively selected sites are often found at the surface of proteins (Proux et al. 2009; Adams et al. 2017). Hence, exploring the role that these structural elements play in shaping the rate of adaptive evolution is crucial in order to fully understand what are the main drivers of adaptation within proteomes.

Our study addresses protein adaptive evolution at a fine scale by analyzing the impact of several functional variables among protein-coding regions at the population level. To further assess the potential generality of the inferred effects, we carried our comparison on two model species with distinct life-history traits: the dipter *Drosophila melanogaster* and the brassicaceae *Arabidopsis thaliana*. We fitted models of DFE and estimated the rate of adaptive substitutions, both at the protein and amino-acid residue scale, across several variables and found that solvent exposure is the most significant factor influencing protein adaptation, with exposed residues undergoing ten times faster $\omega_{a}$ than buried ones. Moreover, we observed that the functional class of proteins has also a strong impact on the rate of protein adaptation, with genes encoding for processes of protein regulation and signaling pathways exhibiting the highest $\omega_{a}$ values. We, therefore, hypothesized that intermolecular interactions are the main drivers of adaptive substitutions in proteins. This hypothesis is consistent with the proposal that, at the interorganism level, coevolution with pathogens constitute a so-far under-assessed component of protein evolution (Sackton et al. 2007; Obbard et al. 2009; Enard et al. 2016; Mauch-Mani et al. 2017).

## Results and Discussion

In order to identify the genomic and structural variants driving protein adaptive evolution, we looked at 10,318 protein-coding genes in 114 *Drosophila melanogaster* genomes, analyzing polymorphism data from an admixed sub-Saharan population from Phase 2 of the *Drosophila* Population Genomics Project (DPGP2, Pool et al. 2012) and divergence out to *D. simulans*; and 18,669 protein-coding genes in 110 *Arabidopsis thaliana* genomes, with polymorphism data from a Spanish population (1001 Genomes Project, Weigel and Mott 2009) and divergence to *A. lyrata*. The rate of adaptive evolution was estimated with the Grapes program (Galtier 2016). The Grapes method extends the approach pioneered by the DoFE program (Fay et al. 2001; Smith and Eyre-Walker 2002; Bierne and Eyre-Walker 2004; Eyre-Walker et al. 2006; Eyre-Walker and Keightley 2009; Stoletzki and Eyre-Walker 2011), by explicitly accounting for mutations with slightly advantageous effects. Grapes estimates the rate of nonadaptive nonsynonymous substitutions ($\omega_{\mathrm{na}}$), which is then used to estimate the rate of adaptive nonsynonymous substitutions ($\omega_{a}$) and the proportion of adaptive nonsynonymous substitutions (α). A high α can be potentially explained both by a higher $\omega_{a}$ or a lower $\omega_{\mathrm{na}}$, and therefore does not allow to disentangle the two effects. Thus, we explored whether, and how, $\omega_{a}$ and $\omega_{\mathrm{na}}$, as well as the total ω, depend on the different functional variables analyzed here.

Results from the model comparison of DFE showed that the Gamma-Exponential model is the one that best fits our data according to Akaike’s information criterion (Akaike 1973) (supplementary table S1 in supplementary file S1, Supplementary Material online). This model combines a Gamma distribution of deleterious mutations with an exponential distribution of beneficial mutations. In agreement with previous surveys within animal species, this model suggests the existence of slightly deleterious, as well as slightly beneficial segregating mutations in *D. melanogaster* and *A. thaliana* genomes (Galtier 2016). Genome-wide estimates of $\omega_{a}$ for *A. thaliana* and *D. melanogaster* are 0.05 and 0.09, respectively, and are in the range of previously reported estimates for these species (Smith and Eyre-Walker 2002; Bierne and Eyre-Walker 2004; Gossmann et al. 2012).

In order to investigate the main drivers of protein adaptive evolution, we divided the data sets into sets of genes and amino-acid residues according to the variables analyzed, and fitted models of DFE in each subset independently. We distinguished two types of analyses: gene-based and site-based, where we looked into how the molecular adaptive rate varies across different categories of genes and amino-acid residues, respectively. Gene-based analyses allowed us to explore the impact of the background recombination rate, the number of introns, mean expression levels, and breadth of expression. At the protein level, we investigated the effect of binding affinity to the molecular chaperone *DnaK*, protein length, cellular localization of proteins, protein functional class, and number of protein–protein interactions (PPI). Finally, site-based analyses enabled us to study the effect of the secondary structure (SS) of the protein, by comparing residues present in β-sheets, α-helices, and loops; the tertiary structure, by considering the RSA of a residue and the residue intrinsic disorder; and whether an amino-acid residue participated or not in an annotated active site.

### The Impact of Gene and Genome Architecture on Adaptive Evolution

To study the impact of gene and genome architecture on the rate of adaptive evolution, we looked at recombination rate and the number of introns. Recombination rate was previously reported to favor the fixation of adaptive mutations in Drosophila by breaking down linkage disequilibrium (Marais and Charlesworth 2003; Castellano et al. 2016). Our results are consistent with previous observations by showing a significant positive correlation in estimates of $\omega_{a}$ with increasing levels of recombination rate for *D. melanogaster* (table 1 and supplementary fig. S1 and file S2, Supplementary Material online). This was also observed in *A. thaliana* (table 1 and supplementary fig. S1 and file S2, Supplementary Material online), thus corroborating the effect of recombination in the rate of adaptive evolution.

**Table 1.** Number of Genes and Categories Analyzed for Each Continuous Variable and the Corresponding Kendall’s τ with the Respective Significance (*P < 0.05; **P < 0.01; ***P < 0.001; “.” 0.05 ≤ *P* < 0.10) for ω, $\omega_{\mathrm{na}}$, and $\omega_{a}$ for *Arabidopsis thaliana* and *Drosophila melanogaster*.

	*A. thaliana*					*D. melanogaster*				
	Number of Categories	Number of Genes	$\omega_{a}$	$\omega_{\mathrm{na}}$	ω	Number of Categories	Number of Genes	$\omega_{a}$	$\omega_{\mathrm{na}}$	ω
Recombination rate	50	18,668	0.2065 (*)	−0.2212 (*)	0.0857	30	8,485	0.3839 (**)	−0.402 (**)	0.0759
Intron number	13	15,347	−0.1538	−0.3590 (.)	−0.7949 (***)	10	10,318	−0.3333	−0.866 (***)	−0.7333 (**)
Protein length	30	18,669	−0.1310	−0.6735 (***)	−0.6782 (***)	50	10,318	−0.4775 (***)	−0.6963 (***)	−0.7763 (***)
Relative solvent accessibility	28	9,034	0.7513 (***)	0.8466 (***)	0.9841 (***)	19	4,944	0.8129 (***)	0.5789(***)	0.9766 (***)
Protein intrinsic disorder (site)	30	18,668	0.6000 (***)	0.9172 (***)	0.9770 (***)	30	8,485	0.7057 (***)	0.6690(***)	0.9540 (***)
Proportion of disordered residues (gene)	30	18,668	0.1908	0.7333 (***)	0.7517 (***)	20	8,485	0.7263 (***)	0.0631	0.5684 (***)
Breadth of expression	4	17,999	−0.6667	−1.0000 (*)	−1.0000 (*)	6	4,601	−0.7333 (*)	−0.4667	−0.7333 (*)
Mean gene expression	40	17,999	−0.1385	−0.9154 (***)	−0.9282 (***)	15	6,247	−0.5048 (**)	−0.6190 (**)	−0.7714 (***)
Protein–protein interactions	–	–	–	–	–	19	5,628	−0.3099 (.)	−0.1111	−0.3684 (*)

Previous studies proposed that genes containing more introns are under stronger selective constraints due to the high cost of transcription, especially in highly expressed genes (Castillo-Davis et al. 2002). Hence, we would expect regions with more introns to be under stronger purifying selection. Conversely, by increasing the total gene length, introns might also effectively increase the intragenic recombination rate, which could in turn increase the efficacy of positive selection and have a positive impact on $\omega_{a}$. To disentangle the two effects, analyses were performed by comparing genes with different intron content. Results showed a significant negative correlation of $\omega_{\mathrm{na}}$ with an increasing number of introns in *D. melanogaster* (table 1 and supplementary fig. S2 and file S2, Supplementary Material online). Conversely, the number of introns did not significantly correlate with $\omega_{a}$ (table 1 and supplementary fig. S2 and file S2, Supplementary Material online). These findings suggest that the effect of the intron content on the rate of protein evolution is essentially due to stronger purifying selection while having a negligible influence on the rate of adaptive substitutions.

### The Impact of Protein Structure on Adaptive Evolution

We further explored the impact of three different levels of protein structure (i.e., primary, secondary, and tertiary) on the rate of adaptive evolution. We first looked at the primary structure by categorizing proteins according to their length. Former studies correlating gene length and $d_{N}/d_{S}$ have shown that smaller genes evolve more rapidly (Zhang 2000; Lipman et al. 2002; Liao et al. 2006). Here, we investigated whether this faster evolution is followed by a higher rate of adaptive substitutions. Results show significant negative correlations with protein length for values of ω and $\omega_{\mathrm{na}}$ in both species (table 1 and supplementary fig. S3 and file S2, Supplementary Material online). The same trend was observed for $\omega_{a}$, although it was only significant in *D. melanogaster* (table 1 and supplementary fig. S3 and file S2, Supplementary Material online). These findings suggest that smaller protein-coding regions are indeed under more relaxed purifying selection but might also evolve, in some cases, under a higher rate of adaptive substitutions.

**Fig. 1.**
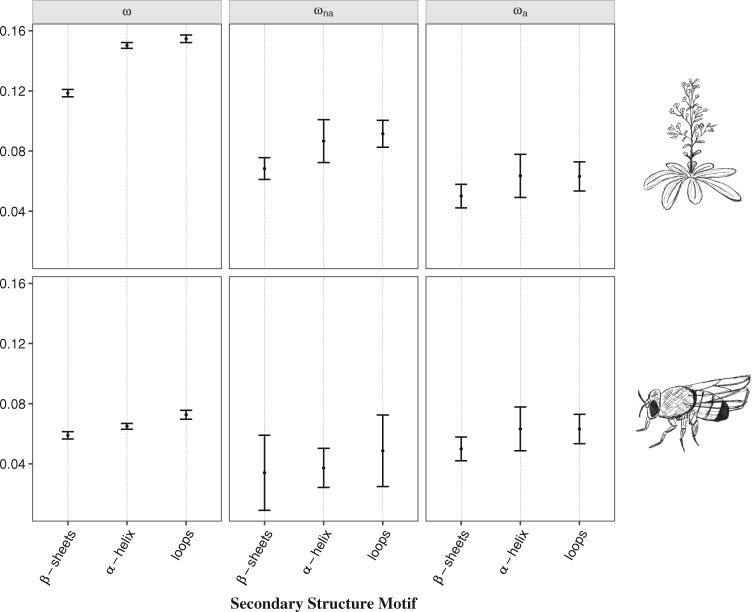
Estimates of the rate of protein evolution (ω), nondaptive nonsynonymous substitutions ($\omega_{\mathrm{na}}$), and adaptive nonsynonymous substitutions ($\omega_{a}$) for each of the secondary structural motif (β-sheets, α-helices, and loops) in *Arabidopsis thaliana* (top) and *Drosophila melanogaster* (bottom). Mean values of ω, $\omega_{\mathrm{na}}$, and $\omega_{a}$ for each motif are represented with the black points. Error bars denote for the 95% confidence interval for each category, computed over 100 bootstrap replicates. The hand-drawings of *A. thaliana* and *D. melanogaster* were made by A.F.M.

The analysis at the secondary structural level showed significant differences in the evolutionary rate between the structural motifs, with loops demonstrating the highest values of ω, followed by α-helices and β-sheets (table 2 and fig. 1). When considering adaptive and nonadaptive substitutions separately, β-sheets show significantly lower values of $\omega_{\mathrm{na}}$ in *A. thaliana* and $\omega_{a}$ in both species, with marginally significant values observed for *D. melanogaster* (table 2, fig. 1 and supplementary file S3, Supplementary Material online). This implies that the structural motif has an impact on the selective constraints in *A. thaliana* and also contributes to the rate of adaptation in the two species. Previous studies investigating protein tolerance to amino-acid change have similarly shown that loops and turns are the most mutable, followed by α-helices and β-sheets (Goldman et al. 1998; Guo et al. 2004; Choi et al. 2006). Some authors posed this relationship as an outcome of residue exposure (Goldman et al. 1998; Guo et al. 2004), while others associate it to the degree of structural disorder, where ordered proteins are under stronger selective constraint (Choi et al. 2006). In order to clarify this, we further look into the impact of tertiary structure, by exploring the relationship between residue exposure to solvent and intrinsic protein disorder with the rate of adaptive evolution.

**Table 2.** Number of Genes and Categories Analyzed for Each Discrete Variable and the Corresponding Difference between the Mean Values of Each Category is Reported for ω, $\omega_{\mathrm{na}}$, and $\omega_{a}$ for *Arabidopsis thaliana* and *Drosophila melanogaster*.

	Pairwise Comparisons	*A. thaliana*					*D. melanogaster*				
		Number of Categories	Number of Genes	$\omega_{a}$	$\omega_{\mathrm{na}}$	ω	Number of Categories	Number of Genes	$\omega_{a}$	$\omega_{\mathrm{na}}$	ω
Secondary structure	β-sheets–α-helices	3	9,034	−0.01346 (*)	−0.0182 (.)	−0.0317 (*)	3	4,944	−0.0132 (.)	−0.0033	−0.0060 (*)
	β-sheets–loops			−0.0130 (*)	−0.0231 (*)	−0.0361 (*)			−0.0131 (.)	−0.0146	−0.0137 (*)
	α-helices–loops			0.0004	−0.0049	−0.0045 (*)			0.00009	−0.0114	−0.0076 (*)
Affinity to molecular Chaperone	Binder–Non-Binder	2	17,775	0.0092	0.0260	0.0352 (*)	2	9,420	0.00009	0.0606 (*)	0.0515 (*)
Protein location		7	18,669				7	10,318			
Protein functional class		27	3,780				23	2,948			

Note.—Significance levels as in table 1.

a Due to the large amount of comparisons, the detailed pairwise comparisons and the corresponding *P* values are detailed in supplementary files S3 and S4, Supplementary Material online.

**Fig. 2.**
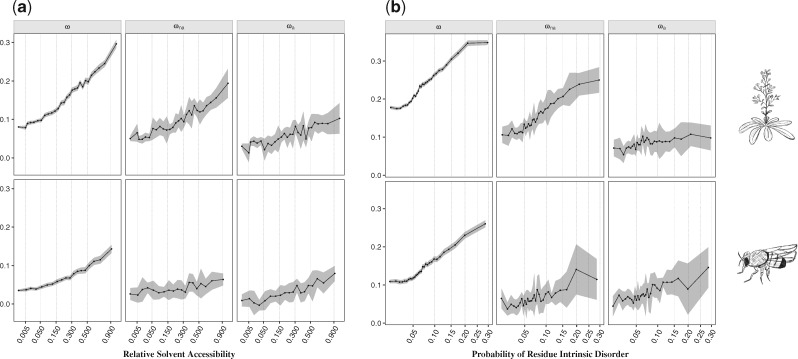
Relationship between ω, $\omega_{\mathrm{na}}$, and $\omega_{a}$ with (*a*) the relative solvent accessibility (RSA) and (*b*) the probability of residue intrinsic disorder for *Arabidopsis thaliana* (top) and *Drosophila melanogaster* (bottom). The *x* axis is scaled using a squared root function. Mean values of each estimate for each category are represented with connected black dots. The shaded area represents the 95% confidence interval of each category, computed over 100 bootstrap replicates.

Considering the RSA, several studies previously demonstrated that residues at the surface of proteins evolve faster than the ones at the core (Goldman et al. 1998; Choi et al. 2007; Lin et al. 2007; Franzosa and Xia 2009). This higher substitution rate can be either due to a reduced selective constraint at exposed residues and/or to an increased rate of adaptive substitutions. To disentangle the two effects, we compared the site frequency spectra (SFS) across several categories of RSA. Our results recapitulate those of previous studies on divergence and demonstrate a significant positive correlation with solvent exposure for values of ω (table 1 and fig. 2*a*). Moreover, we demonstrate that both relaxation of the selective constraints ($\omega_{\mathrm{na}}$) and a higher rate of adaptive nonsynonymous substitutions ($\omega_{a}$) explain the higher evolutionary rate at the surface of proteins (table 1, fig. 2*a* and supplementary file S2, Supplementary Material online).

Intrinsically disordered proteins are defined by lacking a well-defined 3D fold (Dunker et al. 2002; Dyson and Wright 2005), more specifically, proteins that have a higher degree of loop dynamics (“hotloops”) (Linding et al. 2003). As these structures are more flexible, we expect them to be under less structural constraint and to accumulate more substitutions (Guo et al. 2004; Wilke et al. 2005; Choi et al. 2006; Afanasyeva et al. 2018), either deleterious and/or beneficial. To test this hypothesis, we asked two different questions: 1) Are intrinsically disordered protein regions more likely to respond to adaptation? 2) Are proteins with more disordered regions undergoing more adaptive substitutions? For the first question, we divided amino-acid residues based on their predicted value of intrinsic disorder. We report a significant positive correlation with ω, $\omega_{a}$, and $\omega_{\mathrm{na}}$ with residue intrinsic disorder for both species (table 1, fig. 2*b* and supplementary file S2, Supplementary Material online). For the second question, proteins were categorized according to their proportion of disordered residues (see Materials and Methods). Our results reveal a significant positive correlation of protein disorder with ω in both species, $\omega_{\mathrm{na}}$ in *A. thaliana* and $\omega_{a}$ in *D. melanogaster* (table 1 and supplementary fig. S4 and file S2, Supplementary Material online). These findings suggest that, at the residue level, intrinsically disordered regions are more likely to respond to adaptation and are also under less selective constraint in both species. However, when considering the whole protein, we observe that intrinsically disordered proteins have different effects between species. In particular, they contribute to the relaxation of purifying selection in *A. thaliana* and to a higher rate of adaptation in *D. melanogaster*. The reason for the difference between species is unclear and will require further analyses.

Finally, we tested whether the rate of adaptive substitutions is affected by the binding affinity of proteins to molecular chaperones. It has been suggested that binding to a chaperone leads to a higher evolutionary rate due to the buffering effect for slightly deleterious mutations (Bogumil and Dagan 2010; Kadibalban et al. 2016). Here, we investigate whether binding to the chaperone *DnaK* could also favor the fixation of adaptive mutations. In agreement with previous studies, we find a higher ω and $\omega_{\mathrm{na}}$ in proteins binding to *DnaK* in *D. melanogaster* (table 2 and supplementary fig. S5, Supplementary Material online), but no impact on $\omega_{a}$ (table 2 and supplementary fig. S5 and file S3, Supplementary Material online), suggesting that the interaction with a molecular chaperone does not influence the fixation of beneficial mutations.

### Protein Function and Adaptive Evolution

**Fig. 3.**
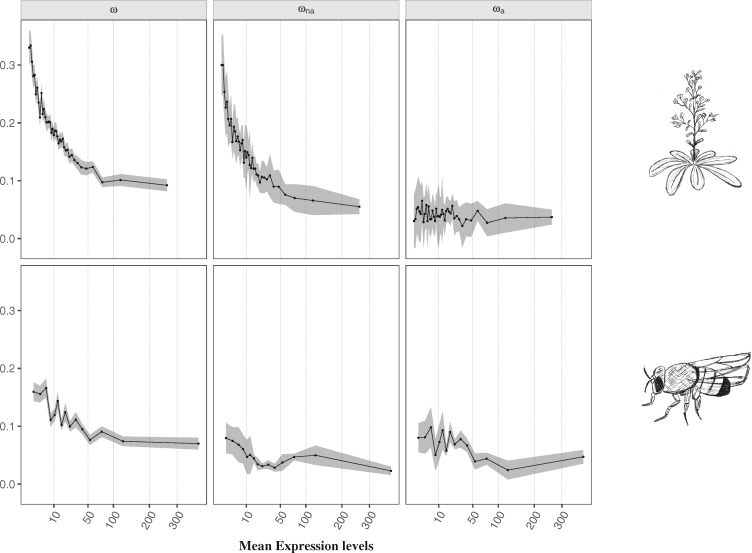
Estimates of ω, $\omega_{\mathrm{na}}$, and $\omega_{a}$ for each category of genes with distinct mean gene expression levels for *Arabidopsis thaliana* (top) and *Drosophila melanogaster* (bottom). The *x* axis is scaled using a squared root function. Legend as in figure 2.

We further explored the impact of protein function on sequence evolution. To do so, we analyzed the effect of mean gene expression, breadth of expression, protein location, and protein functional class on the rate of adaptive substitutions. Several studies on both Eukaryote (Pal et al. 2001; Subramanian and Kumar 2004; Wright et al. 2004; Lemos et al. 2005) and Prokaryote (Rocha and Danchin 2004) organisms have shown that highly expressed genes have lower rates of protein sequence evolution. Here, we investigated if the lower evolutionary rate is followed by a reduced rate of adaptive substitutions. Our results support previous findings by displaying a significant negative correlation of mean gene expression with estimates of ω and $\omega_{\mathrm{na}}$ in both species (table 1, fig. 3 and supplementary file S2, Supplementary Material online). Besides, we find that mean gene expression is also significantly negatively correlated with $\omega_{a}$ in *D. melanogaster* (table 1, fig. 3 and supplementary file S2, Supplementary Material online), suggesting that gene expression also constrains the rate of adaptation, in addition to the well-known effect on purifying selection. It has been hypothesized that the higher selective constraint in highly expressed genes could be driven by the reduced probability of protein misfolding, wherein selection acts by favoring protein sequences that accumulate less translational missense errors (Drummond et al. 2005). Hence, the higher selective pressure to increase stability in highly expressed proteins could also be hampering the fixation of adaptive mutations. Moreover, as mean gene expression is positively correlated with the breadth of expression (Kendall’s *τ* = 0.3376, *P *<* *2.2e-16 in *A. thaliana*; Kendall’s *τ* = 0.2170, *P *<* *2.2e-16 in *D. melanogaster*; supplementary fig. S6, Supplementary Material online), and the latter is a good proxy for the pleiotropic effect of a gene, which is known to impose high selective constraints (i.e., Salvador-Martínez et al. 2018), we also analyzed the impact of the number of tissues where a gene is expressed on the rate of adaptive evolution. We report a significant negative correlation of the breadth of expression (number of tissues) with ω in both species (table 1 and supplementary fig. S7, Supplementary Material online), thus corroborating previous findings (Duret and Mouchiroud 2000; Slotte et al. 2011; Salvador-Martínez et al. 2018). When looking at adaptive and nonadaptive substitutions separately, we observe a significant negative impact on values of $\omega_{a}$ in *D. melanogaster* and $\omega_{\mathrm{na}}$ in *A. thaliana* (table 1 and supplementary fig. S7 and file S2, Supplementary Material online). This suggests that the breadth of expression is acting together with the mean expression levels, although with an apparently lower magnitude effect both in $\omega_{\mathrm{na}}$ and $\omega_{a}$.

In order to assess the impact of protein location, we classified genes into the following cellular categories: cytoplasmic, endomembrane system, mitochondrial, nuclear, plasma membrane, and secreted proteins (supplementary tables S2 and S3 in supplementary file S1, Supplementary Material online). Results show significantly higher rates of protein evolution in nuclear and secreted proteins, with the lowest values observed in the mitochondria, plasma membrane, and endomembrane system (pairwise comparisons; *P *=* *0.0128 in *A. thaliana*; *P *=* *0.0104 in *D. melanogaster*; supplementary fig. S8, Supplementary Material online). However, this result seems to be explained by a reduced purifying selection, with significantly higher values of $\omega_{\mathrm{na}}$ observed in cytoplasmic, nuclear, and secreted proteins (pairwise comparisons; *P *=* *0.0128 in *A. thaliana*; *P *>* *0.0729 in *D. melanogaster*; supplementary fig. S8, Supplementary Material online), and not by a higher rate of adaptive substitutions, since no significant differences were found between the categories in the estimates of $\omega_{a}$ (supplementary fig. S8 and file S3, Supplementary Material online).

**Fig. 4.**
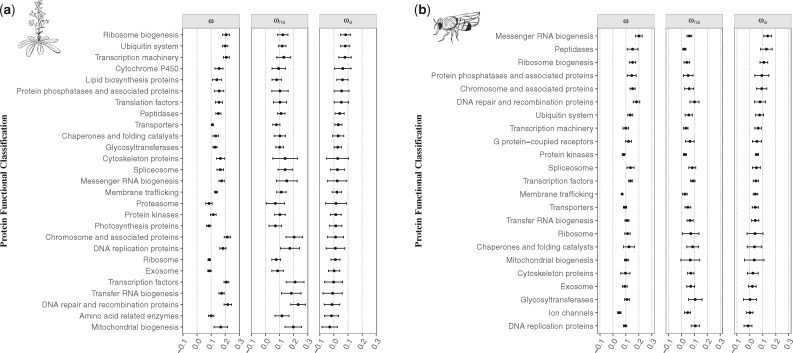
Estimates of ω, $\omega_{\mathrm{na}}$, and $\omega_{a}$ for each category of protein functional class in (*a*) *Arabidopsis thaliana* and (*b*) *Drosophila melanogaster*. Categories are ordered according to the values of $\omega_{a}$. Mean values of ω, $\omega_{\mathrm{na}}$, and $\omega_{a}$ for each class are represented with the black points. Error bars denote the 95% confidence interval for each category, computed over 100 bootstrap replicates.

By analyzing the different categories of protein functional class (supplementary tables S2 and S3 in supplementary file S1, Supplementary Material online), we observe that genes involved in protein biosynthesis (i.e., mRNA and ribosome biogenesis and transcription machinery) and signaling for protein degradation (ubiquitin system) exhibit the highest rates of adaptive substitutions (fig. 4 and supplementary file S4, Supplementary Material online), functions coded mostly by nuclear and cytoplasmic proteins. Signal transduction pathways also appear to play a role in adaptation, since protein phosphatases also present high rates of adaptive mutations (Hunter 1995). Moreover, in *A. thaliana*, cytochrome P450 proteins are also in the top categories of $\omega_{a}$ (fig. 4 and supplementary file S4, Supplementary Material online). We fitted a linear model to the $\omega_{a}$ values of the shared categories (21 categories in total) to see if results were consistent between the two species and found a positive correlation (Kendall’s τ = 0.257, *P* = 0.1101; supplementary fig. S9*a*, Supplementary Material online), which is stronger after discarding the two outliers, mRNA biogenesis and glycosyltransferases (Kendall’s τ = 0.333, *P* = 0.0490; supplementary fig. S9*b*, Supplementary Material online). Our findings, therefore, suggest that adaptive mutations occur mainly through processes of protein regulation and signaling pathways.

### What Are the Major Drivers of Adaptive Evolution along the Genome?

**Fig. 5.**
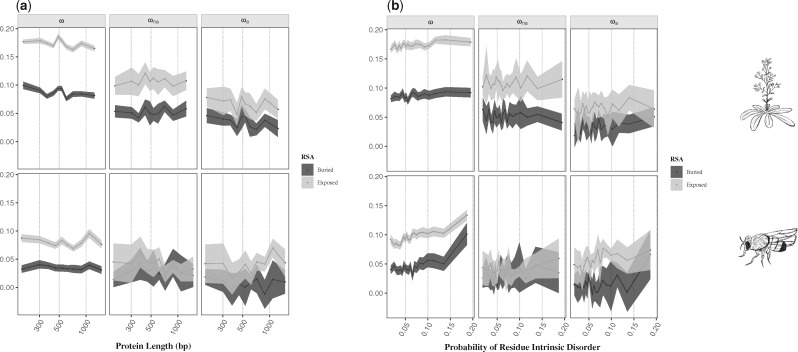
Estimates of ω, $\omega_{\mathrm{na}}$, and $\omega_{a}$ plotted as a function of (*a*) the relative solvent accessibility and protein length and (*b*) the relative solvent accessibility and the probability of residue intrinsic disorder in *Arabidopsis thaliana* (top) and *Drosophila melanogaster* (bottom). The *x* axis is log-scaled. Analyses were performed by comparing buried (RSA <0.05) and exposed (RSA ≥0.05) residues across ten categories of protein length in (*a*) and 20 categories of intrinsic disorder in (*b*) for both species. Legend as in figure 2.

Overall, we found multiple factors influencing protein adaptive evolution, specifically recombination rate (positive correlation), protein length (negative correlation), secondary structural motif (lower values observed for β-sheets), RSA (positive correlation), protein intrinsic disorder (positive correlation), gene expression levels (negative correlation), and protein functional class. Since some of these variables are intrinsically correlated, we next asked whether some of the inferred effects are spurious. First of all, it is known that protein length and gene expression are negatively correlated, wherein highly expressed genes tend to be shorter, as previously reported for vertebrates (Subramanian and Kumar 2004), yeast (Coghlan and Wolfe 2000; Akashi 2003), and observed in this study (Kendall’s τ = −0.015, *P *=* *1.22e-02 in *A. thaliana*; τ = −0.093, *P *=* *1.70e-28 in *D. melanogaster*; supplementary fig. S10, Supplementary Material online). Since highly expressed genes have lower rates of adaptive substitutions and shorter genes have higher rates of adaptive evolution, we may conclude that these two variables independently impact the rate of adaptation in proteins. Protein length is also negatively correlated with the proportion of exposed residues (Kendall’s τ = −0.310, *P *=* *0.00 in *A. thaliana*; τ = −0.404, *P *=* *1.03e-223 in *D. melanogaster*; supplementary fig. S11, Supplementary Material online), as the surface/volume ratio of globular proteins decreases when protein length increases (Janin 1979). By estimating the rate of adaptive mutations of buried and exposed sites separately, we observe that the effect of protein length is no longer significant (table 3, fig. 5*a* and supplementary file S5, Supplementary Material online). This suggests that the effect of protein length on the rate of adaptive substitutions is a by-product of the effect of the residue’s solvent exposure. Furthermore, mean gene expression is positively correlated with solvent exposure (Kendall’s τ = 0.016, *P *=* *0.1037 in *A. thaliana*; τ = 0.327, *P *=* *4.50e-45 in *D. melanogaster*; supplementary fig. S12, Supplementary Material online), as expected since highly expressed genes are shorter and shorter genes have a greater proportion of exposed residues (supplementary figs. S10 and S11, Supplementary Material online). These two variables, however, have opposite effects on $\omega_{a}$, and we therefore conclude that gene expression is acting independently from solvent exposure on the rate of adaptive protein evolution.

**Table 3.** Statistical Results for the Comparisons Performed Including RSA as a Cofactor.

	Categories	Statistics	*Arabidopsis thaliana*		*Drosophila melanogaster*	
			RSA		RSA	
			Buried	Exposed	Buried	Exposed
Protein length	10	$\omega_{a}$	−0.4222 (.)	−0.2889	−0.0667	0.3333
		$\omega_{\mathrm{na}}$	−0.0222	0.0667	−0.0667 (.)	−0.4222 (.)
Protein disorder	20	$\omega_{a}$	0.2105	0.2105	0.0842	0.5368 (***)
		$\omega_{\mathrm{na}}$	−0.0631	−0.0211	0.2947	−0.0316
Secondary structure	Β-sheets–α-helices	$\omega_{a}$	−0.0073	−0.0074	0.0118	−0.0040
		$\omega_{\mathrm{na}}$	0.0003	−0.0230 (.)	−0.0063	−0.0006
	Β-sheets–loops	$\omega_{a}$	−0.0021	−0.0078	0.0178	−0.0056
		$\omega_{\mathrm{na}}$	0.0050	−0.0173 (*)	−0.0133	−0.0039
	α-helices–loops	$\omega_{a}$	0.0052	−0.0003	0.0059	−0.0016
		$\omega_{\mathrm{na}}$	0.0047	0.0056	−0.0071	−0.0033
Active site	Active–nonactive	$\omega_{a}$	−0.0004	−0.0048	−0.0078	0.0055
		$\omega_{\mathrm{na}}$	−0.0057	0.0070	0.0042	−0.0045

Note.—For each comparison, the value for buried and exposed residues is indicated. For continuous variables (protein length and protein disorder), the Kendall’s τ with the respective significance for $\omega_{\mathrm{na}}$ and $\omega_{a}$ is reported. For discrete variables (secondary structure motif and active site) the difference between the mean values of each category is reported for $\omega_{\mathrm{na}}$ and $\omega_{a}$. Significance levels as in table 1.

We further note that the SS motif is intrinsically correlated with the degree of intrinsic disorder, where loops and turns represent the most flexible motifs (supplementary fig. S13, Supplementary Material online), consistent with previous studies (Choi et al. 2006). When analyzing different degrees of protein disorder across the structural motifs, we observe that SS has only an impact on estimates of ω, while intrinsic protein disorder is significantly positively correlated with ω within the three motifs in both species, and $\omega_{a}$ within β-sheets in *A. thaliana* and within α-helices in *D. melanogaster* (supplementary fig. S14 and file S5, Supplementary Material online). Moreover, we report that the SS motif is correlated with solvent exposure (supplementary fig. S15, Supplementary Material online), β-sheets being mostly found at the core of proteins, while α-helices and loops have, on an average, higher solvent exposure (Bowie et al. 1990; Guo et al. 2004). By estimating the rate of adaptive substitutions in buried and exposed residues across the three motifs, the impact of SS is no longer noticeable on estimates of $\omega_{a}$ (table 3 and supplementary fig. S16 and file S5, Supplementary Material online), thus suggesting that the effect of SS motif is also a by-product of solvent exposure. When looking at the tertiary structure level, in agreement with Choi et al. (2006), we report that structures with more exposed residues tend to be more flexible (Kendall’s τ = 0.001, *P *=* *0.4726 in *A. thaliana*; τ = 0.015, *P *=* *0.0256 in *D. melanogaster*; supplementary fig. S17, Supplementary Material online). Estimation of the rate of adaptive mutations in buried and exposed sites across different levels of residue intrinsic disorder shows that solvent exposure plays the main role in protein adaptive evolution, with a significant positive impact of protein disorder only observed in values of ω in both species and $\omega_{a}$ in exposed residues for *D. melanogaster* (table 3, fig. 5*b* and supplementary file S5, Supplementary Material online). To further clarify the relative contribution of solvent exposure and protein disorder on the rate of adaptive evolution, we performed an analysis of covariance (ANCOVA), using both measures and their interaction as explanatory variables. Results show that the RSA explains 95% (*P *=* *3.176e-14) and 99% (*P *<* *2.2e-16) of the variation in $\omega_{a}$ and $\omega_{\mathrm{na}}$, respectively, in *A. thaliana*; and 87% (*P *=* *1.011e-13) and 62% (*P *=* *0.00012) in $\omega_{a}$ and $\omega_{\mathrm{na}}$, respectively, in *D. melanogaster*. These findings suggest that the level of exposure of a residue in the protein structure is the main driver of adaptive evolution, and that structural flexibility potentially constitutes a comparatively small, if any, effect to protein adaptation. By comparing the level of exposure of the residues across the different classes of protein function, no differences were observed (supplementary fig. S18, Supplementary Material online), thus suggesting that these two variables independently affect the rate of protein adaptation.

Summarizing, after accounting for potentially confounding effects, our results show that besides population genetic processes such as recombination and mutation rate (Hill and Robertson 1966; Marais and Charlesworth 2003; Castellano et al. 2016), three major protein features significantly impact the rate of protein adaptive evolution: gene expression, RSA, and the protein functional class. When looking at the magnitude effect of each of these variables, we observe that exposed residues have a 10-fold higher rate of adaptive substitutions when compared with completely buried sites (fig. 2*a* and supplementary file S2, Supplementary Material online). The effect of gene expression seems to be of lower magnitude, wherein less expressed genes have a 2-fold higher rate of adaptive substitutions with a significant negative correlation observed only in *D. melanogaster* (fig. 3 and supplementary file S2, Supplementary Material online). As a comparison, genes in highly recombining regions have up to a 10-fold higher rate of adaptive substitutions compared with genes within regions with the lowest recombination rates (supplementary fig. S1 and file S2, Supplementary Material online), being therefore similar to that observed with solvent exposure. Previous studies reported that the type of amino-acid change also plays an important role in protein adaptive evolution, where more similar amino-acids present higher rates of adaptive substitutions (Grantham 1974; Miyata et al. 1979; Bergman and Eyre-Walker 2019). In order to evaluate a potential bias on the type of amino-acid at the surface and at the core of proteins, we computed the proportion of conservative and radical residue changes, according to volume and polarity indices, as defined by Grantham (Grantham 1974). We found similar frequencies of conserved and radical changes in buried and exposed residues, thus suggesting that our results at the structural level are not influenced by the type of amino-acid mutation (97% of conservative and 3% changes on buried residues; 96% of conservative and 4% changes on exposed sites). Our findings therefore suggest that protein architecture strongly influences the rate of adaptive protein evolution, wherein selection acts by favoring a greater accumulation of adaptive mutations at the surface of proteins.

### Why Does Adaptation Occur Mainly at the Surface of Proteins?

Our results show that solvent exposure is the protein feature with the strongest impact on the rate of adaptive substitutions at the intramolecular level. To explain this effect, we discuss three hypotheses in which protein adaptive evolution occurs through 1) the acquisition of new biochemical activities at the surface of proteins, 2) the emergence of new functions via network rewiring at the level of PPI, and 3) intermolecular interactions between organisms, as a consequence of host–pathogen coevolution.

We first hypothesized that protein adaptation results from new catalytic activities, wherein adaptive mutations arise within active sites. Bartlett et al. (2002) reported that active sites are mostly present in more intrinsically disordered regions of the protein. Moreover, they proposed that apo-enzymes, which are not yet bound to the substrate or cofactor, present greater residue flexibility, and more exposed catalytic residues, which could favor a higher rate of adaptive substitutions. In order to test this, we estimated the rate of adaptive substitutions on active and nonactive sites, controlling for solvent exposure, and observed only significant differences in ω within buried residues in *A. thaliana* (table 3 and supplementary fig. S19 and file S5, Supplementary Material online), although with higher values observed for nonactive sites. While the nonsignificant differences in the rate of adaptive mutations could result from incomplete annotations, which tend to be biased toward motifs highly conserved across species (De Castro et al. 2006), this suggests that being present in an active site does not influence the rate of adaptation. Active sites, however, are rather mobile, presenting different levels of solvent exposure and residue flexibility according to the stage of the enzymatic reaction (Bartlett et al. 2002). Therefore, it may be arbitrary to assign them a certain solvent exposure class based on the phase the enzymes were crystallized, limiting our capacity to test their role on adaptive evolution.

Several studies discussed the impact of PPI on the rate of protein evolution. Valdar and Thornton (2001) and Caffrey et al. (2004) proposed that PPI may be acting as an inhibitor of protein evolution by enhancing the efficiency of purifying selection due to a higher degree of protein connectivity, typically associated with more complex functions. Mintseris and Weng (2005) supported this assumption but proposed that the proteins evolving slowly are the ones involved in obligate interactions, while proteins involved in transient interactions evolve at faster rates due to higher interface plasticity. Here, we ask whether the higher rate of adaptive mutations at the surface of proteins could have arisen through intermolecular interactions at the protein network level. We addressed this question by estimating the rate of adaptive mutations in genes with different degrees of PPI. This was only possible in *D. melanogaster* since there was limited data available for *A. thaliana*. We report a negative correlation between the number of PPI and ω, $\omega_{\mathrm{na}}$, and $\omega_{a}$, respectively, with only significant values observed for ω (table 1 and supplementary fig. S20 and file S2, Supplementary Material online). These findings suggest that a higher degree of protein connectivity leads to lower rates of protein sequence evolution, but prevent us to assess with confidence whether this effect is due to a stronger purifying selection and/or a slower rate of adaptive substitutions. A potential limitation of this analysis is the low number of genes with PPI information available and the noise associated with the BioGRID annotations. As a physical interaction does not necessarily imply a functional link, we might lack statistical power to detect any putative effect of PPI on $\omega_{a}$ (Chatr-aryamontri et al. 2017).

In support to our third hypothesis, several studies have described the role of the immune and defense responses in molecular evolution across taxa (Sackton et al. 2007; Obbard et al. 2009; Enard et al. 2016; Mauch-Mani et al. 2017). These studies suggest that pathogens could be key drivers of protein adaptation, by acting as a powerful selective pressure through the coevolutionary arms race between hosts and parasites. This could be driving the higher rate of adaptive mutations in protein biosynthesis enzymes (fig. 4), which are the ones typically hijacked by pathogens during host infection (Dangl and Jones 2001; Enard et al. 2016). Moreover, one of the fastest evolving protein class is the ubiquitin system (fig. 4), which is known to be involved in the defense mechanism, both by the host, through processes like the activation of innate immune responses and degradation signaling of pathogenic proteins; and by the pathogen, which inhibits and/or uses this system in order to modulate host responses (Loureiro and Ploegh 2006; Collins and Brown 2010; Dielen et al. 2010; Trujillo and Shirasu 2010; Hiroshi et al. 2014). Membrane trafficking proteins are also well-known for being involved in the immune response mechanisms, a functional class that also presents high values of $\omega_{a}$, and “DNA replication” together with “mRNA biogenesis” and “transcription machinery” are typical signatures of viruses’ activities (fig. 4). Likewise, in *A. thaliana*, cytochrome P450 proteins present a high rate of adaptive mutations (fig. 4), which have been reported to play a crucial role in the defense response in plants (Schuler and Werck-Reichhart 2003). Besides, the reduced selective pressure on nuclear and secreted proteins (supplementary fig. S6, Supplementary Material online) may be also a consequence of their role in disease and pathogen immunity (i.e., Motion et al. 2015; Mosmann et al. 2016), as observed in yeast (Julenius and Pedersen 2006), insects (Sackton et al. 2007; Obbard et al. 2009), and primates (Nielsen et al. 2005).

Our findings, therefore, support the hypothesis that coevolutionary arms race of the host–pathogen interactions, in particular, intracellular pathogens such as viruses, are a major driver of adaptation in proteins. While we do not rule out that PPI and the acquisition of new biochemical functions could also have an impact, more and better annotation data is required to further evaluate their role. In conclusion, our study reveals that, in addition to genome architecture, protein structure has a substantial impact on adaptive evolution consistent between *D. melanogaster* and *A. thaliana*, unraveling the potential generality of such effect. Our study further emphasizes that the rate of adaptation not only varies substantially between genes but also at the intragenic scale, and we posit that accounting for a fine-scale, intramolecular evolution is necessary to fully understand the patterns of molecular adaptation at the species level.

## Materials and Methods

### Population Genomic Data and Data Filtering

The *D. melanogaster* data set included alignments of 114 genomes for one chromosome arm of the two large autosomes (2 L, 2 R, 3 L, and 3 R) and one sex chromosome (X) pooled from 22 sub-Saharan populations with a negligible amount of population structure ($F_{\mathrm{ST}}$ = 0.05; DPGP2, Pool et al. 2012). Release 5 of the Berkeley Drosophila Genome Project (BDGP5, http://www.fruitfly.org/sequence/release5genomic.shtml, last accessed July 2017) was used as the reference genome. Estimations of divergence were performed with *D. simulans*, for which genome alignments with the reference genome were available (http://www.johnpool.net/genomes.html; last accessed July 2017). For *A. thaliana*, analyses were carried out with 110 genomes for the five chromosomes of the Spanish population from the 1001 Genomes Project (Weigel and Mott 2009), using the release 10 from The Arabidopsis Information Resource (TAIR10, ftp://ftp.ensemblgenomes.org/pub/plants/release-40/fasta/arabidopsis_thaliana/dna/; last accessed March 2018) as the reference genome. Divergence estimates were made with *A. lyrata* as an outgroup species, for which a pairwise alignment with the reference genome was available (ftp://ftp.ensemblgenomes.org/pub/plants/release-38/maf; last accessed March 2018). Data processing was conducted with the help of GNU parallel (Tange 2011).

### Estimation of the Population Genetic Parameters and Model Selection

Coding DNA sequences (CDS) were extracted from the alignments with MafFilter (Dutheil et al. 2014) according to the General Feature Format (GFF) file of the reference genome of both species. First, a cleaning and filtering process was performed to keep only nonoverlapping genes with the longest transcript, in cases of multiple transcripts per gene. At this stage, 12,801 and 27,072 genes, for *D. melanogaster* and *A. thaliana*, respectively, were kept for further analysis. CDS sequences were then concatenated in order to obtain the full coding region per gene. For the analysis with *A. thaliana*, the alignment of *A. lyrata* with the reference sequence was realigned with each gene alignment of the ingroup using MAFFT v7.38 (Katoh and Standley 2013) with the options *add* and *keeplength* so that no gaps were included in the ingroup. CDS alignments with premature stop codons were excluded and alignment positions lacking a corresponding sequence in the outgroup were discarded. Final data sets included 10,318 genes for *D. melanogaster/D. simulans* and 18,669 genes for *A. thaliana/A. lyrata*. These data sets were then used to infer both the synonymous and nonsynonymous unfolded and folded SFS, and synonymous and nonsynonymous divergence based on the rate of synonymous and nonsynonymous substitutions. Sites for which the outgroup allele was missing were considered as missing data. All calculations were performed using the BppPopStats program from the Bio++ Program Suite (Guéguen et al. 2013). The Grapes program was then used to compute a genome-wide estimate of the rate of nonadaptive ($\omega_{\mathrm{na}}$) and adaptive nonsynonymous substitutions ($\omega_{a}$) (Galtier 2016). This method assumes that all sites were sampled in the same number of chromosomes and since some sites were not successfully sampled in all individuals, the original data set was reduced to 110 and 105 individuals for *D. melanogaster* and *A. thaliana*, respectively, by randomly down-sampling polymorphic alleles at each site. The following models were fitted and compared using Akaike’s information criterion: Neutral, Gamma, Gamma-Exponential, Displaced Gamma, Scaled Beta, and Bessel K. A model selection procedure was conducted on the two data sets using the complete set of genes for comparison (see supplementary table S1 in supplementary file S1, Supplementary Material online). As results were comparable when using the unfolded and folded SFS, subsequent analyses were performed on the unfolded SFS only. Following analyses consist in fitting the selected model on several subsets of the data according to the variables analyzed, comprising sets of genes (see supplementary tables S2 and S3 in supplementary file S1, Supplementary Material online, for detailed information on the genes used for each variable as well as the population genetic parameters estimated per gene for *A. thaliana* and *D. melanogaster*, respectively) and amino-acid residues (see supplementary tables S4 and S5 in supplementary file S1, Supplementary Material online, for detailed information on the amino-acid residues used for each category as well as the population genetic parameters estimated per site for *A. thaliana* and *D. melanogaster*, respectively). We next described the different variables analyzed.

### Categorization of Gene and Genome Architecture

Recombination rates were obtained with the R package “MareyMap” (Rezvoy et al. 2007), by using the cubic splines interpolation method. Hereafter, we computed the mean recombination rate in cM/Mb units for each gene. Discretization of the observed distribution of recombination rate was performed in 50 and 30 categories with around 350 and 280 genes each for *A. thaliana* and *D. melanogaster*, respectively. Intronic information was obtained using the GenomeTools from a GFF with exon annotation and the option *addintrons* (Gremme et al. 2013). Genes were discretized into 13 and 10 categories according to their intron content for *A. thaliana* and *D. melanogaster*, respectively.

### Categorization of Protein Structure

Genes were discretized according to the total size of the coding region, for which 30 and 50 categories with around 620 and 210 genes each were made for *A. thaliana* and *D. melanogaster*, respectively.

In order to obtain structural information for each protein sequence, blastp (Schaffer 2001) was first used to assign each protein sequence to a PDB structure, and respective chain, by using the “pdbaa” library and an *E*-value threshold of 1e-10. When multiple matches occurred, for instance in cases of multimeric proteins, the match with the lowest *E*-value was kept. This resulted in 5,008 genes for which a PDB structure was available, making a total of 3,834 PDB structures for *D. melanogaster* and 9,121 genes with a total of 3,832 PDB structures for *A. thaliana*. The corresponding PDB structures were then downloaded and further processed to only keep the corresponding chain per polymer. PDB manipulation and analysis were carried on using the R package “bio3d” (Grant et al. 2006). Values for SS and solvent accessibility (SA) per residue were obtained using the “dssp” program with default options and were successfully retrieved for 3,613 PDB files corresponding to 4,944 genes for *D. melanogaster* and 3,806 PDB files for a total of 9,106 genes for *A. thaliana*. Subsequently, to map SS and SA values to each residue of the protein sequence a pairwise alignment between each protein and the respective PDB sequence was performed with MAFFT, allowing gaps in both sequences in order to increase the block size of sites aligned. The final data set comprised a total of 1,397,885 and 1,395,666 sites with SS and SA information, respectively, out of 4,821,113 total codon sites obtained with BppPopStats for the complete set of genes of *D. melanogaster*; and 2,585,468 and 2,585,467 sites mapped with SS and SA information, respectively, out of 7,479,808 codon sites of *A. thaliana*. We computed the RSA by dividing SA by the amino-acid’s solvent accessible area (Tien et al. 2013).

Categorization of SS was performed by comparing 460,702, 975,934, and 523,880 amino-acid residues in β-sheets, α-helices, and loops, respectively, in *A. thaliana*, and 258,898, 516,356, and 282,588 sites in β-sheets, α-helices, and loops, respectively, in *D. melanogaster*. RSA values were analyzed with 28 categories with around 85,000 sites each, with the exception of the totally buried residues (RSA = 0) category containing 299,684 sites in *A. thaliana*; and 19 categories with approximately 69,000 residues each, except for 151,417 completely buried residues in *D. melanogaster*. For the analysis of correlation between variables two categories of RSA were considered, comparing buried (RSA <0.05) and exposed (RSA ≥0.05) residues, following Miller et al. (1987).

Estimates of intrinsic protein disorder were acquired via the software DisEMBL (Linding et al. 2003), wherein intrinsic disorder was estimated per site and classified according to the degree of “hot loops,” meaning loops with a high degree of mobility. This analysis was successfully achieved for a total of 7,479,807 out of 7,479,808 sites for *A. thaliana* and 3,952,602 out of 4,821,113 sites for *D. melanogaster*. Amino-acid residues were divided into 30 categories with an average of 249,000 and 131,000 sites in *A. thaliana* and *D. melanogaster*, respectively. For the proportion of disordered regions per protein, we considered a residue “disordered” if it was in the top 25% of the measured probabilities of disorder across the proteomes of each species. Analyses were performed with 30 categories with around 620 and 420 genes for *A. thaliana* and *D. melanogaster*, respectively.

### Identification of Proteins Binding to a Molecular Chaperone

Prediction of the molecular chaperone *DnaK* binding sites in the protein sequence was estimated with the LIMBO software using the default option *Best overall prediction*. This setting implies 99% specificity and 77.2% sensitivity (Van Durme et al. 2009). Genes were categorized according to this prediction setting, which suggests that every peptide scoring >11.08 is a predicted *DnaK* binder. Genes scoring below that value were not considered as possible binders.

### Categorization of Gene Expression

Mean gene expression data were obtained from the database Expression Atlas (http://www.ebi.ac.uk/gxa; last accessed March 2019. Petryszak et al. 2016), wherein one baseline experiment was used for each species (*D. melanogaster*, E-MTAB-4723; *A. thaliana*, E-GEOD-38612). In addition, for *D. melanogaster*, we obtained the breadth of expression data over the embryo anatomy from the BDGP database (Tomancak et al. 2007) and the data were processed and analyzed as in Salvador-Martínez et al. (2018). Mean gene expression levels were obtained by averaging across samples and tissues for each gene, ending up with 40 and 15 categories with around 450 and 430 genes each for *A. thaliana* and *D. melanogaster*, respectively. For the analysis on the breadth of expression, expression patterns in *A. thaliana* were analyzed in four different tissues: roots, flowers, leaves, and siliques; and for *D. melanogaster*, we used the anatomical structures of the embryo development, analyzing 18 structures (see Tomancak et al. 2007 and Salvador-Martínez et al. 2018). Analyses were carried with four and six categories in *A. thaliana* and *D. melanogaster*, respectively, according to the number of tissues/organs a gene is expressed (see supplementary tables S2 and S3 in supplementary file S1, Supplementary Material online, for detailed information).

### Protein Cellular Localization and Protein Functional Class

Cellular localization of each protein sequence was predicted with the software ProtComp v9.0 online (from Softberry, http://www.softberry.com/; last accessed May 2018) with the default options and genes were classified into the following cellular categories: cytoplasmic, endomembrane system, mitochondrial, nuclear, peroxisome, plasma membrane, and secreted proteins. The category peroxisome was excluded from further analysis due to the small number of annotated genes (114 and 250 genes in *D. melanogaster* and *A. thaliana*, respectively; detailed information in supplementary tables S2 and S3 in supplementary file S1, Supplementary Material online). Protein functional classes were obtained with the Bioconductor package for R “KEGGREST,” using the KEGG BRITE database (Kanehisa et al. 2002). Analysis was carried out with 2,950 and 3,780 genes for *D. melanogaster* and *A. thaliana*, respectively, discretized into the highest levels of each of the three top categories of protein classification: metabolism, genetic information processing and signaling, and cellular processes (see supplementary tables S2 and S3 in supplementary file S1, Supplementary Material online).

### Enzymatic Active Sites and PPI

In order to check whether a residue was present in an active site, we used the ScanProsite software (De Castro et al. 2006). Data sets included 1,061,876 and 1,870,166 active sites for *D. melanogaster* and *A. thaliana*, respectively. All sites that were not predicted by the program were considered as nonactive (see supplementary tables S4 and S5 in supplementary file S1, Supplementary Material online). Data on the degree of PPI were obtained with the BioGRID database (Chatr-aryamontri et al. 2017). This was only possible for *D. melanogaster* since the data available for *A. thaliana* was very limited (only 878 annotated genes mapping to our data set). Analyses were carried out with 5,628 genes divided into 19 categories, with 1,114 genes in the first category, and the others ranging from 700 to 130 according to the respective number of interactions (see supplementary tables S2 and S3 in supplementary file S1, Supplementary Material online).

### Estimation of the Adaptive and Nonadaptive Rate of Nonsynonymous Substitutions

For all gene and amino-acid sets, 100 bootstrap replicates were generated by randomly sampling genes or sites in each category. The Grapes program was then run on each category and replicate with the Gamma-Exponential DFE (Galtier 2016). The first step included the removal of replicates for which the DFE parameters were not successfully fitted. For this purpose, we discarded 1% in the maximum and minimum values for the mean and shape parameters of the DFE (see supplementary files, Supplementary Material online, for detailed R scripts). Results for ω, $\omega_{\mathrm{na}}$ and $\omega_{a}$ were plotted using the R package “ggplot2” (Wickham 2017) by taking the mean value and the 95% confidence interval of the 100 bootstrap replicates computed for each category (both for main and supplementary figures, for continuous and discrete variables, see supplementary files, Supplementary Material online).

### Statistical Analyses

Significance for all continuous variables, including protein length, number of introns, gene expression, intrinsic residue disorder, proportion of disordered regions, recombination rate, number of PPI, and RSA, was assessed through Kendall’s correlation tests. Kendall’s correlation test is nonparametric and does not make any assumption on the distribution of the input data. Furthermore, it can be applied to ordinal data, making it appropriate to analyze discretized continuous variables. To do so, the mean value of the 100 bootstrap replicates was taken for each category (see detailed script as well as all statistical results in supplementary file S2, Supplementary Material online). Significance values for discrete variables, comprising binding affinity to *DnaK*, protein location, protein functional class and SS motif, were achieved by estimating the differences between each pair of the categories analyzed, by randomly subtracting each bootstrap replicate. The following steps included counting the number of times the differences between categories were below and above 0, which by taking the minimum of those values gives us a statistic that we call k. The two-tailed *P* value was then estimated by applying the following equation: *P* = (2*k* + 1)/(*N* + 1), where *N* in the number of bootstrap replicates used. For variables comparing more than two categories, we corrected the *P* value for multiple testing using the FDR method (Benjamini and Hochberg 1995) as implemented in R (R Core Team 2017) (see detailed script and all statistical results in supplementary files S3 and S4, Supplementary Material online). Analyses on the correlations between variables are described in supplementary files S5 and S6, Supplementary Material online. The ANCOVA was performed by applying a linear model to the values of $\omega_{\mathrm{na}}$ and $\omega_{a}$ with the interaction between RSA and protein disorder following a control for the normality, homoscedasticity, and independence of the corresponding error (supplementary file S5, Supplementary Material online).

## Supplementary Material

Supplementary data are available at *Molecular Biology and Evolution* online.

## Supplementary Material

msz134_Supplementary_Data
