# Supplementary material for: The Impact of Protein Architecture on Adaptive Evolution
Source: Mol Biol Evol. 2019 May 30;36(9):2013–28. doi: 10.1093/molbev/msz134 (PMC6735723; doi:10.1093/molbev/msz134)
Supplement: msz134_Supplementary_Data [file msz134_supplementary_data.zip › FileS1.pdf]

Supplementary File S1 is available in the link https://figshare.com/s/640f86a1dd78f2c41c55.
