# Supplementary material for: The Impact of Protein Architecture on Adaptive Evolution
Source: Mol Biol Evol. 2019 May 30;36(9):2013–28. doi: 10.1093/molbev/msz134 (PMC6735723; doi:10.1093/molbev/msz134)
Supplement: msz134_Supplementary_Data [file msz134_supplementary_data.zip › FileS2.pdf]

Continuous variables

This is the script with all plots and statistical analysis regarding the analysis of continuous variables, refering to: Intrinsic Residue Disorder, Gene Expression, Number of Introns, Number of Protein-Protein Interactions, Proportion of Disordered Residues, Protein Length, Recombination Rate and Relative Solvent Accessibility.

A function was created to call all files for each analysis performed. In this respect the order of the files is as follows:
files.continuous[1] <- Intrinsic Residue Disorder
files.continuous[2] <- Breadth of Expression
files.continuous[3] <- Number of Introns
files.continuous[4] <- Mean Gene Expression
files.continuous[5] <- Number of Protein-Protein interactions
files.continuous[6] <- Proportion of Disordered Residues
files.continuous[7] <- Protein Length
files.continuous[8] <- Recombination Rate
files.continuous[9] <- Relative Solvent Accessibility (RSA)

The first part of the script removes bootstrap replicates for which the fitness effects parameters were not successfully fitted. For this purpose we discard 1% of the values above the maximum and below the minimum of each of the four parameters of fitness effects: Geman.neg, Gshape.neg, Gmean.neg and prop.pos.

```
setwd("/Users/moutinho/Dropbox/Data/Continuous/")

# Libraries
library(plyr)
library(dplyr)
library(data.table)
library(ggplot2)
library(reshape2)
library(doBy)
library(knitr)
library(kableExtra)
#

# calling all output tables
files.continuous <- list.files(".", ".csv")

# reading each table into a list
tbl.list <- lapply(files.continuous, read.table, header = TRUE)

## remove the outliers: 1% of the replicates below the min and
## 1% above the maximum
sub.tbl <- lapply(tbl.list, function(x) {
  ddply(x, c("species", "var.value"), function(x) {
    sum.gmeanNeg <- summary(x$Gmean.neg)
    gmeanNeg.min1 <- as.numeric(sum.gmeanNeg[1]) + 0.01*as.numeric(sum.gmeanNeg[1])
    gmeanNeg.max1 <- as.numeric(sum.gmeanNeg[6]) - 0.01*as.numeric(sum.gmeanNeg[6])
    sum.gshapeNeg <- summary(x$Gshape.neg)
    gshapeNeg.min1 <- as.numeric(sum.gshapeNeg[1]) + 0.01*as.numeric(sum.gshapeNeg[1])
    gshapeNeg.max1 <- as.numeric(sum.gshapeNeg[6]) - 0.01*as.numeric(sum.gshapeNeg[6])
    sum.gmeanPos <- summary(x$Gmean.pos)
    gmeanPos.min1 <- as.numeric(sum.gmeanPos[1]) + 0.01*as.numeric(sum.gmeanPos[1])
    gmeanPos.max1 <- as.numeric(sum.gmeanPos[6]) - 0.01*as.numeric(sum.gmeanPos[6])
```

```
    sum.propPos <- summary(x$prop.pos)
    propPos.min1 <- as.numeric(sum.propPos[1]) + 0.01*as.numeric(sum.propPos[1])
    propPos.max1 <- as.numeric(sum.propPos[6]) - 0.01*as.numeric(sum.propPos[6])
    tbl <- x[!(x$Gmean.neg < gmeanNeg.min1 | x$Gmean.neg > gmeanNeg.max1 &
                 x$Gshape.neg < gshapeNeg.min1 | x$Gshape.neg > gshapeNeg.max1 &
                 x$Gmean.pos < gmeanPos.min1 | x$Gmean.pos > gmeanPos.max1 &
                 x$prop.pos < propPos.min1 | x$prop.pos > propPos.max1),]
  })
})
```

In the next chunk will take only the estimates concerning the rate of adaptive and non-adaptive substitutions, particularly: dnds, omegaNA and omegaA.

```
# In order to keep only the variables that we want to plot:
tbl.rates <- lapply(sub.tbl, function(x) {
  ddply(x, c("var", "species"), function(x) {
    melt(x, id.vars = c("var.value"), measure.vars = c("dnds", "omegaNA", "omegaA"))
  })
})

# function to estimate the mean and satandard deviation to plot the results with the
# mean of the bootstrap replicates and the 95% confidence interval

fun <- function(x){
  c(mean=mean(x), sd=sd(x))
}

# applying the above function to each output table for each value of each estimate
# (dnds, omegaA, omegaNA) for each value of the variable being analyzed for each species

tbl.stats <- lapply(tbl.rates, function(x) {
  summaryBy(value ~ variable + var.value + species + var, data=x, FUN = fun)
})

# to change the estimate name to the respective symbol
tbl.stats[[1]]$variable <- factor(tbl.stats[[1]]$variable,
                                  levels = c("dnds", "omegaNA", "omegaA"))
levels(tbl.stats[[1]]$variable) <- c(expression(omega), expression(omega[na]),
                                     expression(omega[a]))
tbl.stats[[2]]$variable <- factor(tbl.stats[[2]]$variable,
                                  levels = c("dnds", "omegaNA", "omegaA"))
levels(tbl.stats[[2]]$variable) <- c(expression(omega), expression(omega[na]),
                                     expression(omega[a]))
tbl.stats[[3]]$variable <- factor(tbl.stats[[3]]$variable,
                                  levels = c("dnds", "omegaNA", "omegaA"))
levels(tbl.stats[[3]]$variable) <- c(expression(omega), expression(omega[na]),
                                     expression(omega[a]))
tbl.stats[[4]]$variable <- factor(tbl.stats[[4]]$variable,
                                  levels = c("dnds", "omegaNA", "omegaA"))
levels(tbl.stats[[4]]$variable) <- c(expression(omega), expression(omega[na]),
                                     expression(omega[a]))
tbl.stats[[5]]$variable <- factor(tbl.stats[[5]]$variable,
                                  levels = c("dnds", "omegaNA", "omegaA"))
levels(tbl.stats[[5]]$variable) <- c(expression(omega), expression(omega[na]),
```

```
                                    expression(omega[a]))
tbl.stats[[6]]$variable <- factor(tbl.stats[[6]]$variable,
                                  levels = c("dnds", "omegaNA", "omegaA"))
levels(tbl.stats[[6]]$variable) <- c(expression(omega), expression(omega[na]),
                                    expression(omega[a]))
tbl.stats[[7]]$variable <- factor(tbl.stats[[7]]$variable,
                                  levels = c("dnds", "omegaNA", "omegaA"))
levels(tbl.stats[[7]]$variable) <- c(expression(omega), expression(omega[na]),
                                    expression(omega[a]))
tbl.stats[[8]]$variable <- factor(tbl.stats[[8]]$variable,
                                  levels = c("dnds", "omegaNA", "omegaA"))
levels(tbl.stats[[8]]$variable) <- c(expression(omega), expression(omega[na]),
                                    expression(omega[a]))
tbl.stats[[9]]$variable <- factor(tbl.stats[[9]]$variable,
                                  levels = c("dnds", "omegaNA", "omegaA"))
levels(tbl.stats[[9]]$variable) <- c(expression(omega), expression(omega[na]),
                                    expression(omega[a]))
```

In the next chunk the script to plot the results is represented. The same order will follow.

```
# theme of the plot
theme.plot <- function(x) {
  theme(axis.title = element_text(face = "bold", color = "black", size=14),
        text = element_text(size=14),
        axis.title.x = element_text(margin = margin(t = 18, r = 10, b = 0, l = 0)),
        axis.title.y = element_text(margin = margin(t = 18, r = 10, b = 0, l = 0)),
        panel.grid.minor=element_blank(),
        panel.grid.major = element_line(colour = "grey", linetype = "dashed", size = 0.2),
        panel.grid.major.y=element_blank(),
        #strip.text.y = element_blank(),
        axis.text.x = element_text(angle = 60, hjust = 1))
}

# plotting each of the output tables
plot.continuous <- lapply(tbl.stats, function(x) {
  ggplot(x, aes(x = var.value, y = value.mean)) +
  geom_line(col = "black", size = 0.2)+
  geom_ribbon(aes(ymin=value.mean + 1.96*value.sd,
                  ymax=value.mean - 1.96*value.sd), alpha=0.2) +
  geom_point(size=.2)+
  facet_grid(species~variable, scales = "free_x", labeller = label_parsed) +
  ylab("") +
  xlab(as.character(x$var)) +
  scale_x_sqrt() +
  theme_bw() +
  theme.plot()
})

plot.continuous
```

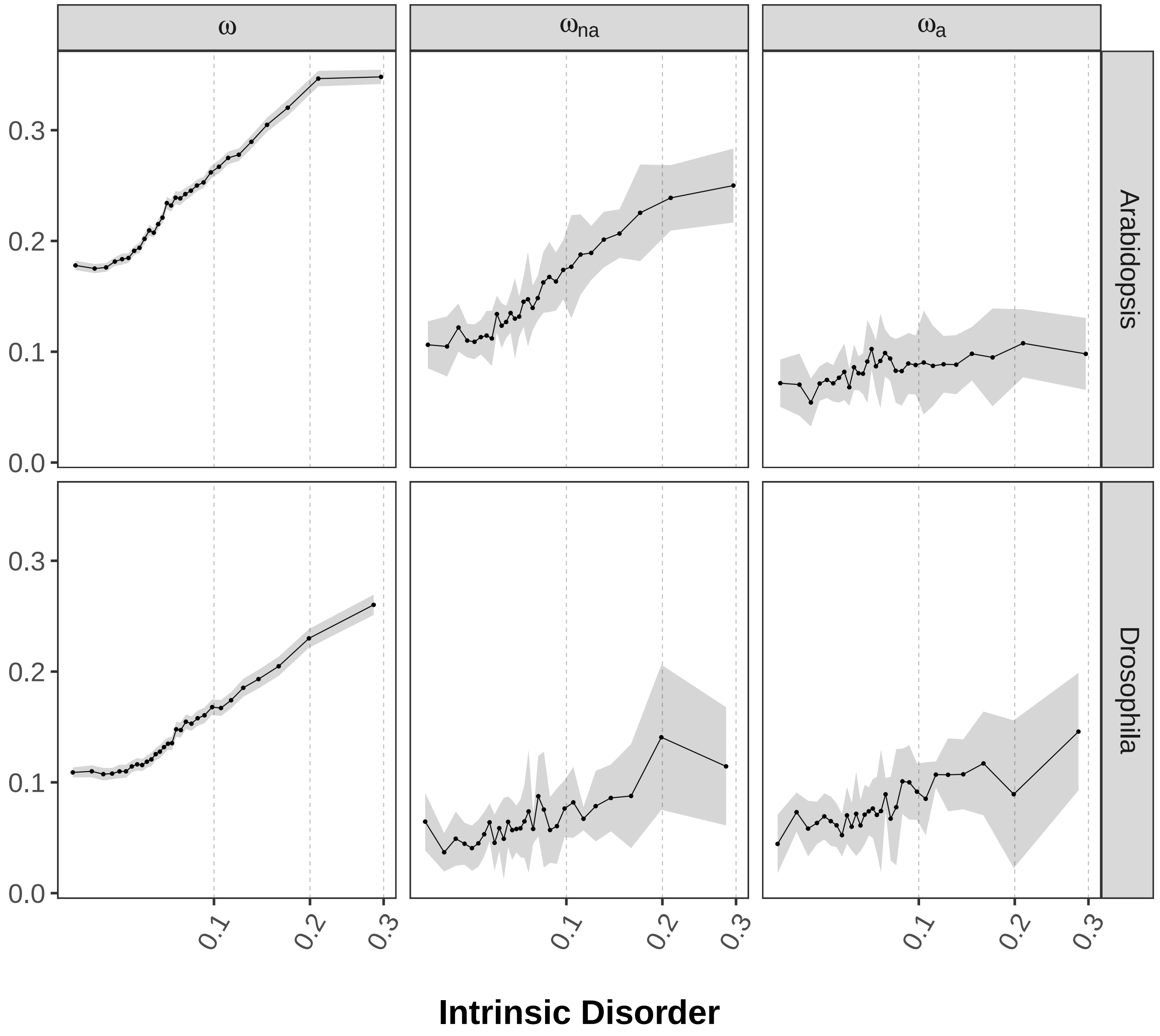
ω
ωna
ωa
Arabidopsis
Drosophila
0.0
0.1
0.2
0.3
0.1
0.2
0.3
Intrinsic Disorder

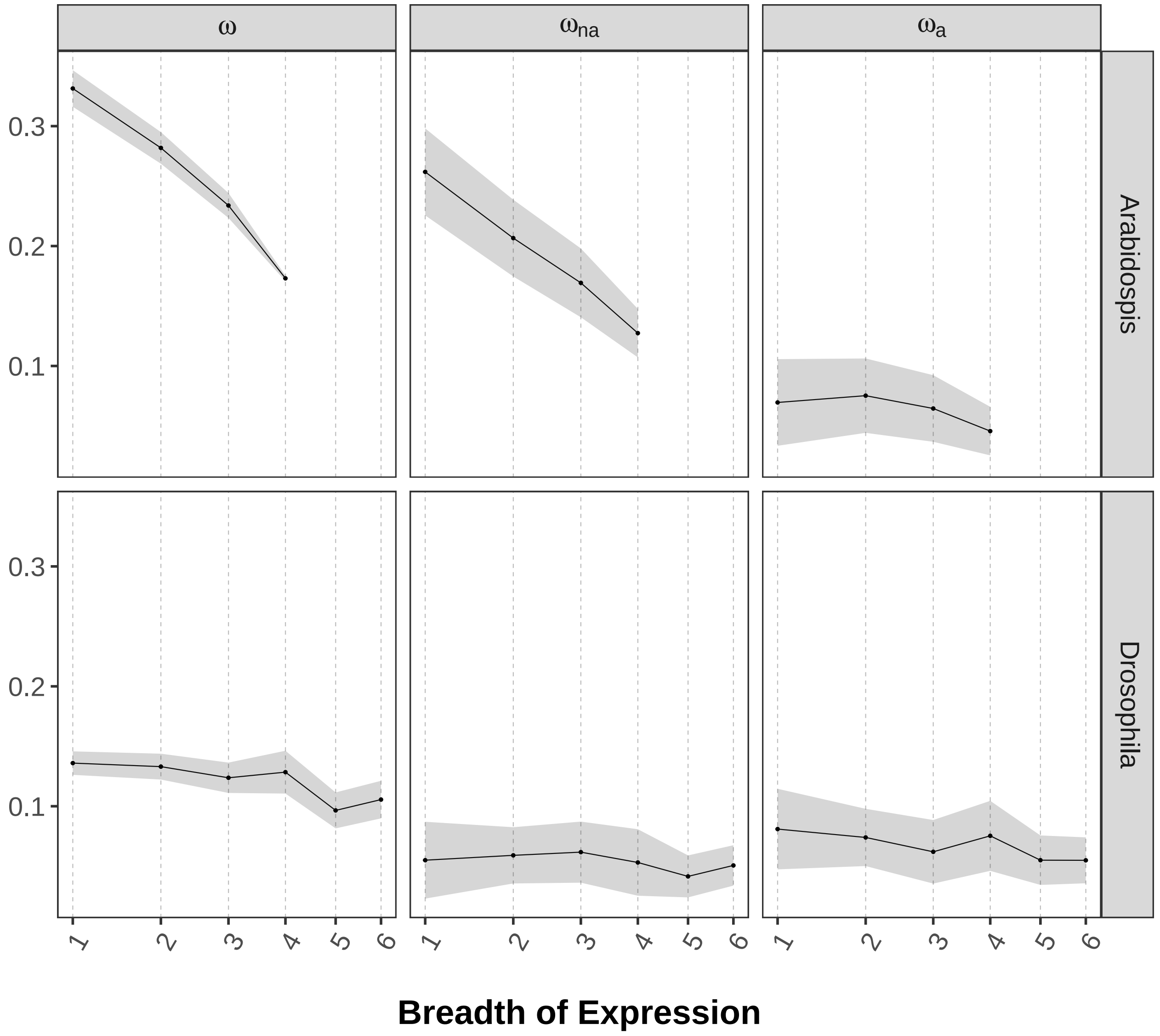
ω
ω_{na}
ω_a
Arabidospis
Drosophila
0.3
0.2
0.1
1
2
3
4
5
6
Breadth of Expression

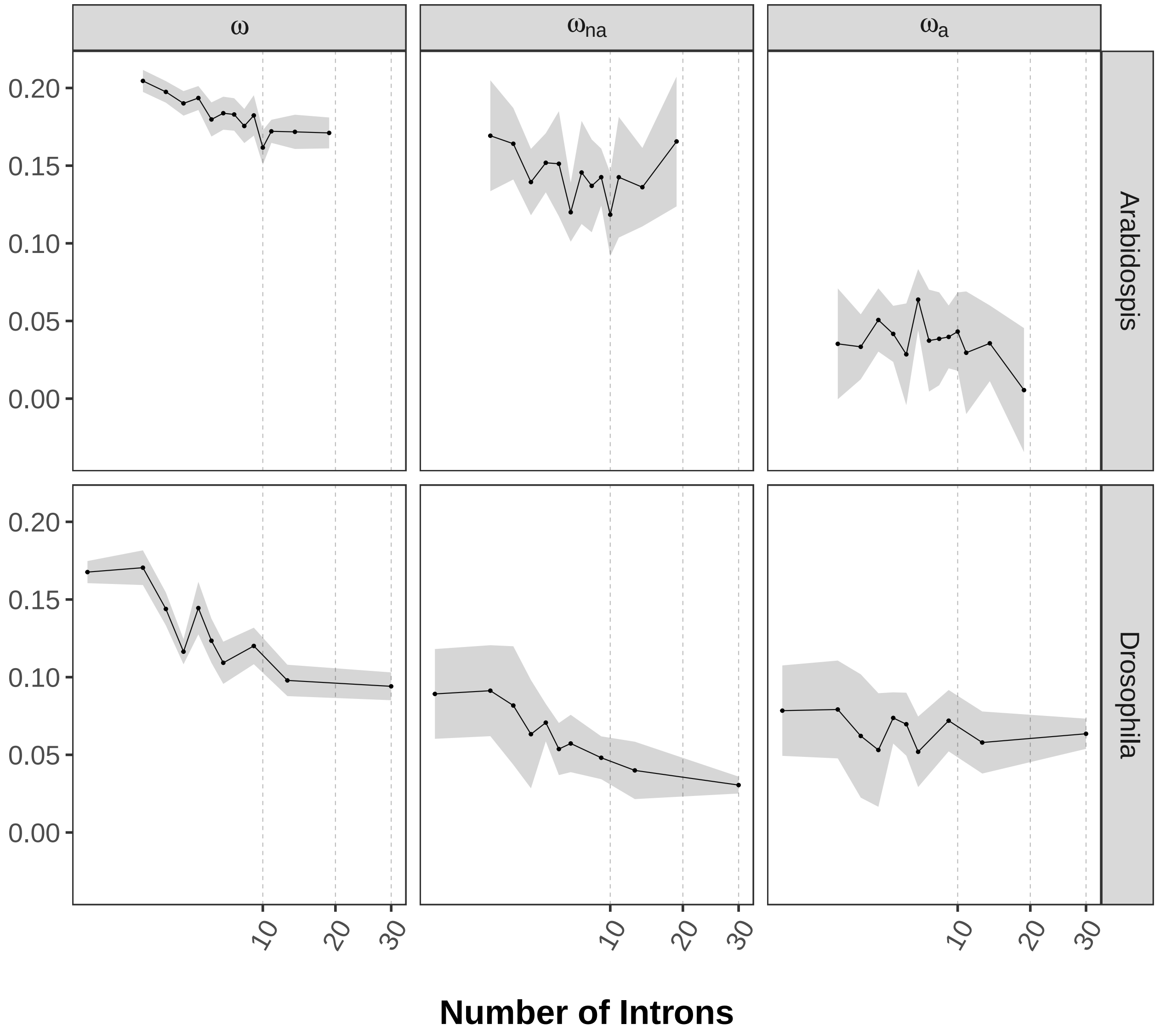

ω
ω_na
ω_a
Arabidospis
Drosophila
0.20
0.15
0.10
0.05
0.00
10
20
30
Number of Introns

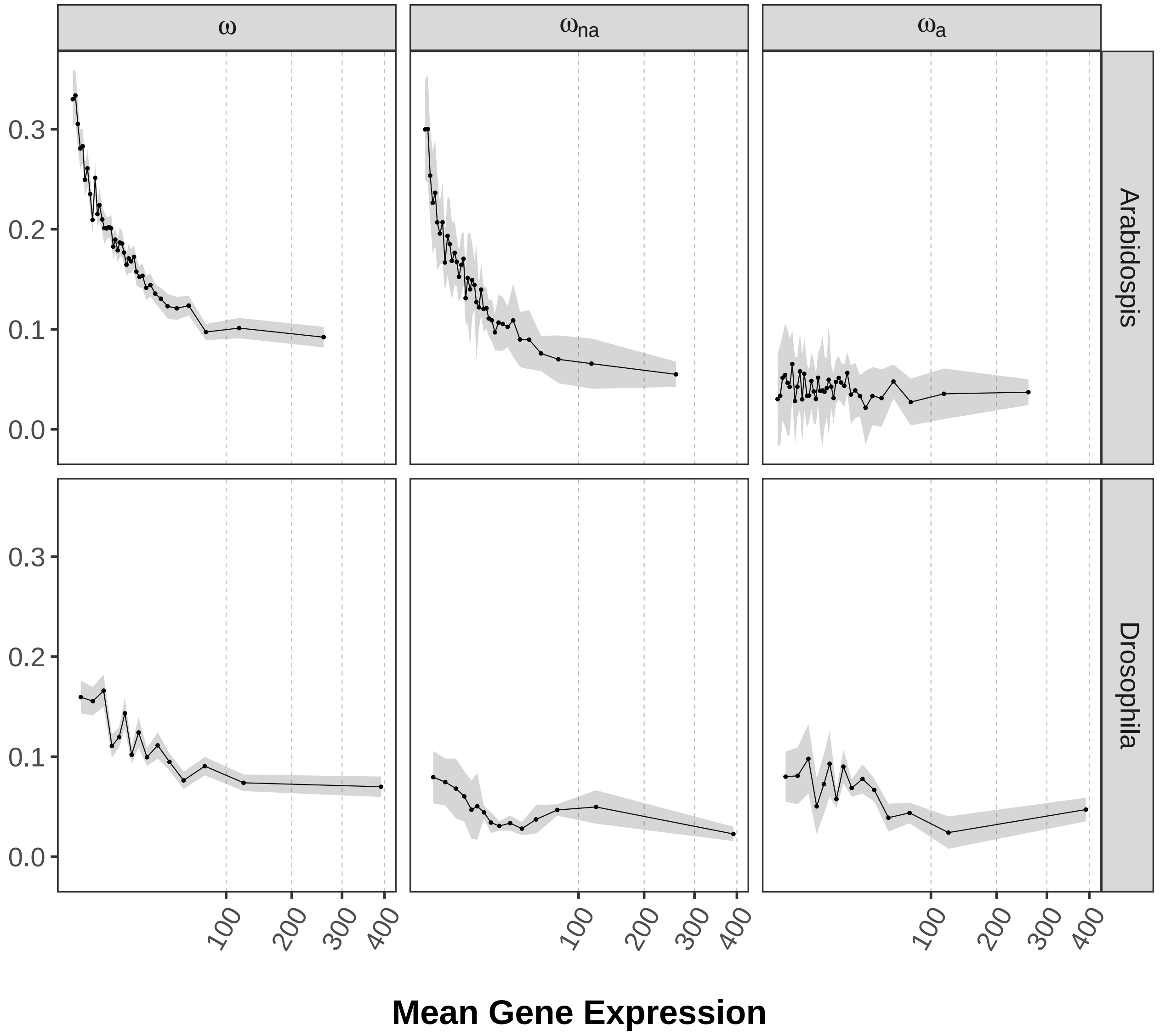
ω
ωna
ωa
Arabidospis
Drosophila
0.0
0.1
0.2
0.3
100
200
300
400
Mean Gene Expression

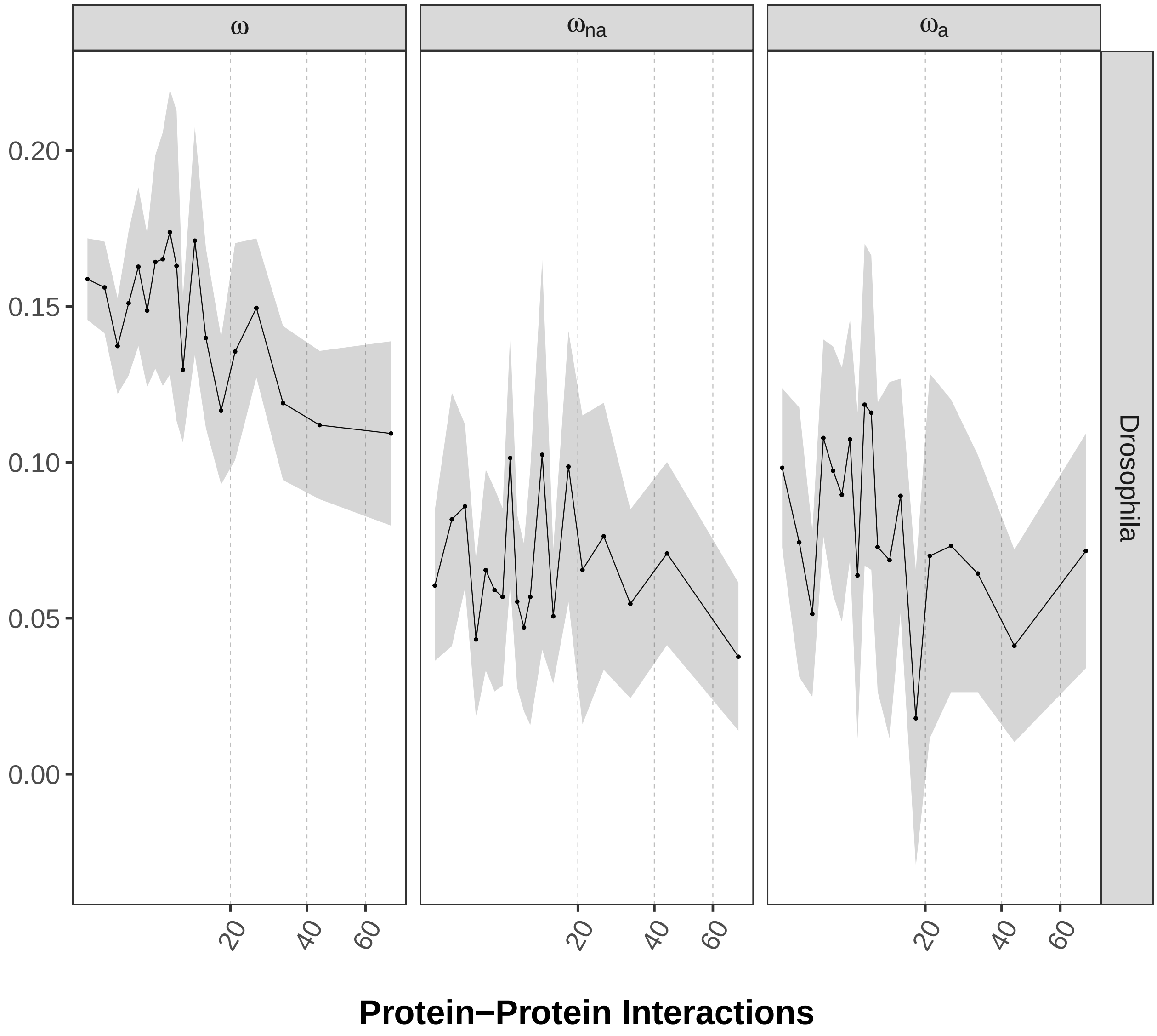
ω
ω_{na}
ω_a
Drosophila
0.20
0.15
0.10
0.05
0.00
20
40
60
Protein−Protein Interactions

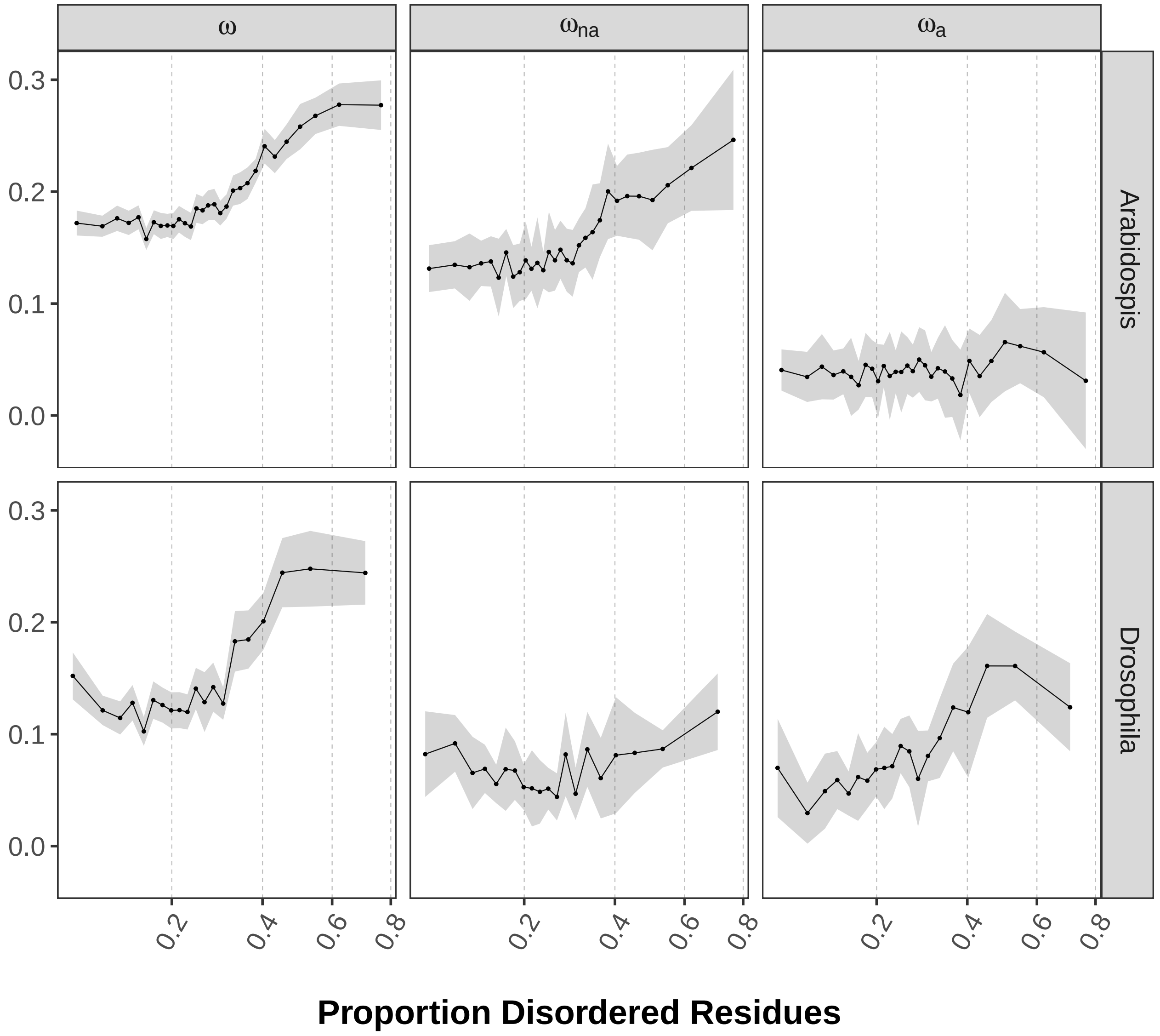
ω
ω_{na}
ω_a
Arabidospis
Drosophila
0.3
0.2
0.1
0.0
0.2
0.4
0.6
0.8
Proportion Disordered Residues

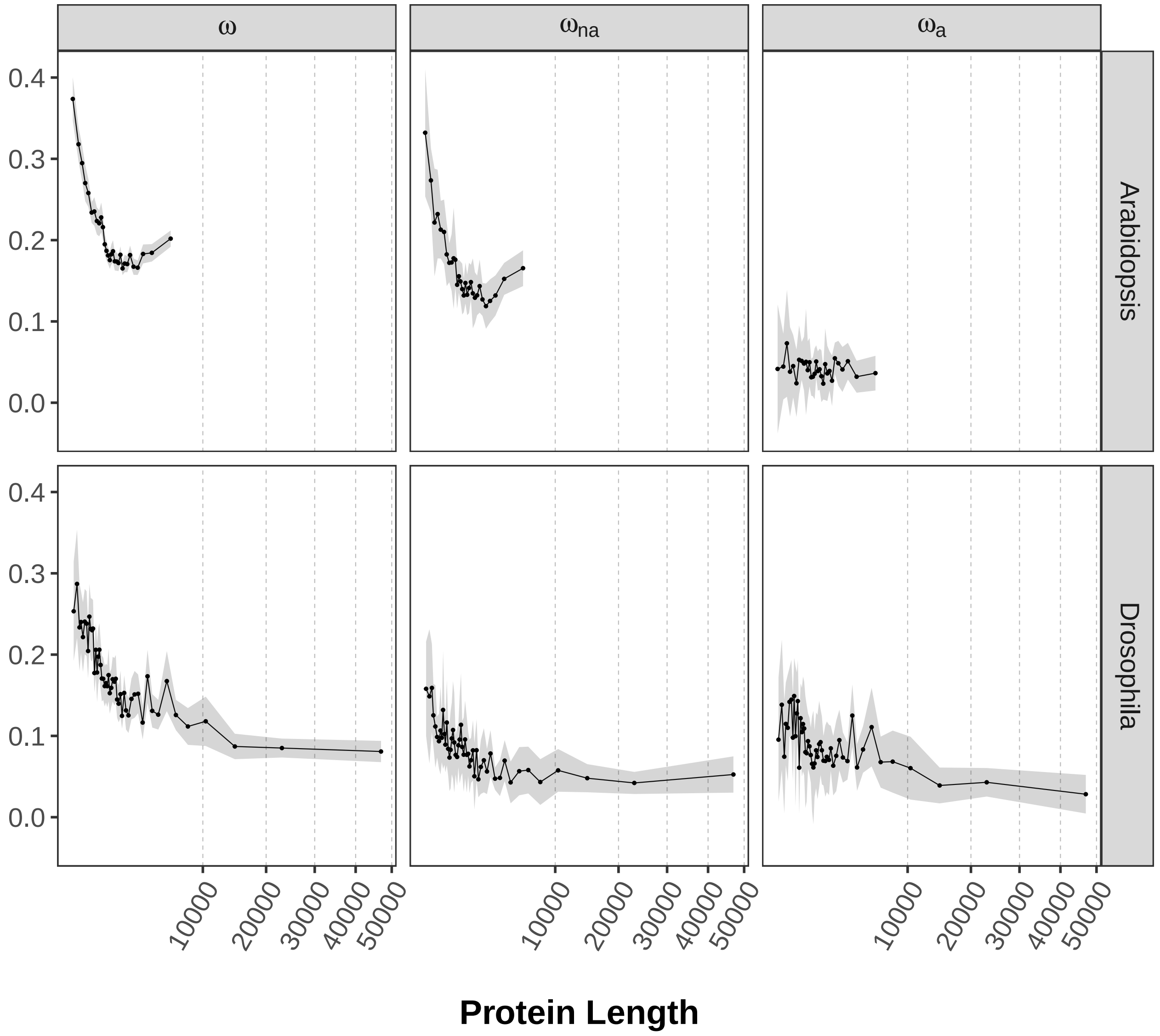
ω
ω_{na}
ω_a
Arabidopsis
Drosophila
0.4
0.3
0.2
0.1
0.0
10000
20000
30000
40000
50000
Protein Length

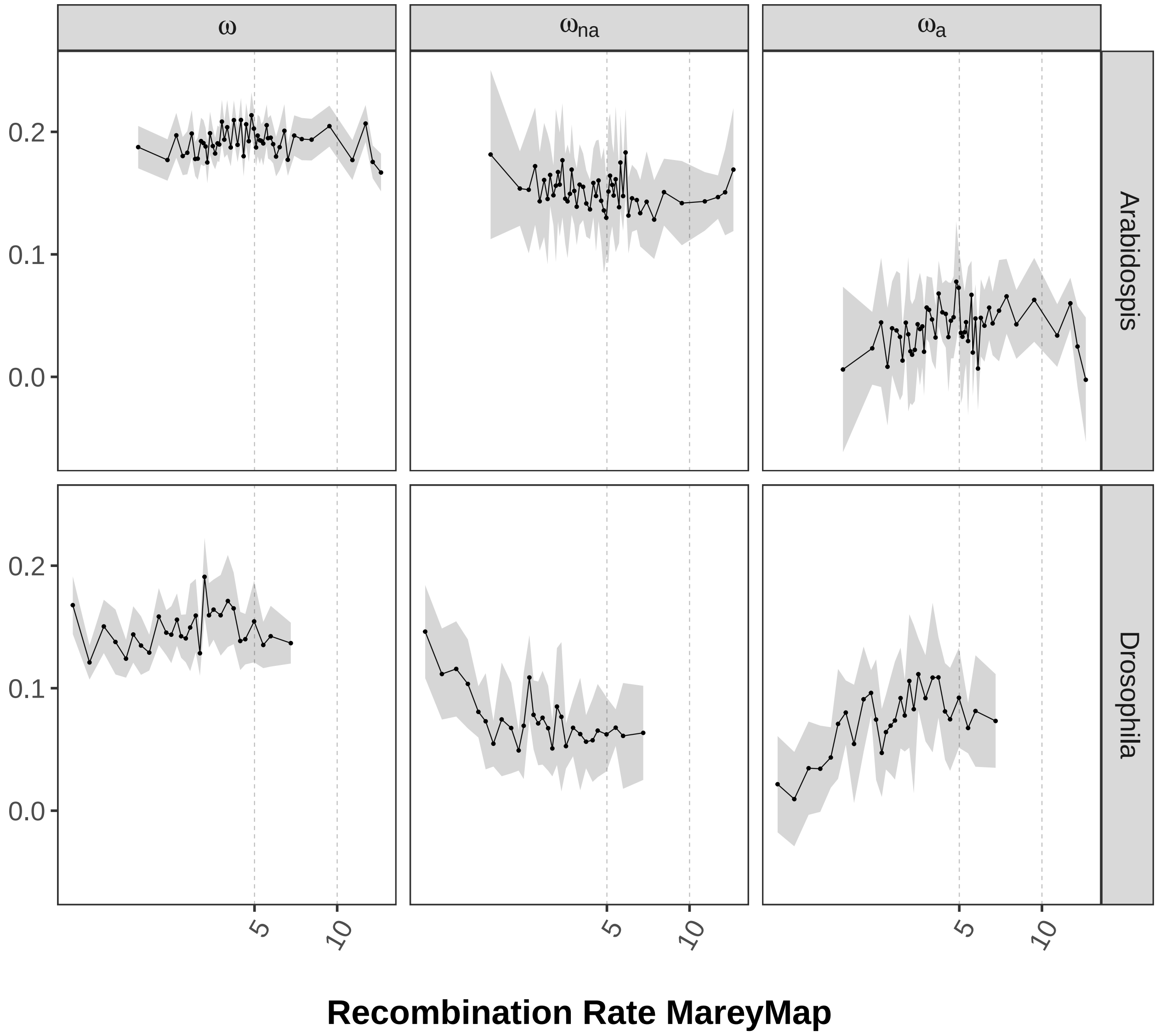

ω
ωna
ωa
Arabidospis
Drosophila
0.2
0.1
0.0
5
10
Recombination Rate MareyMap

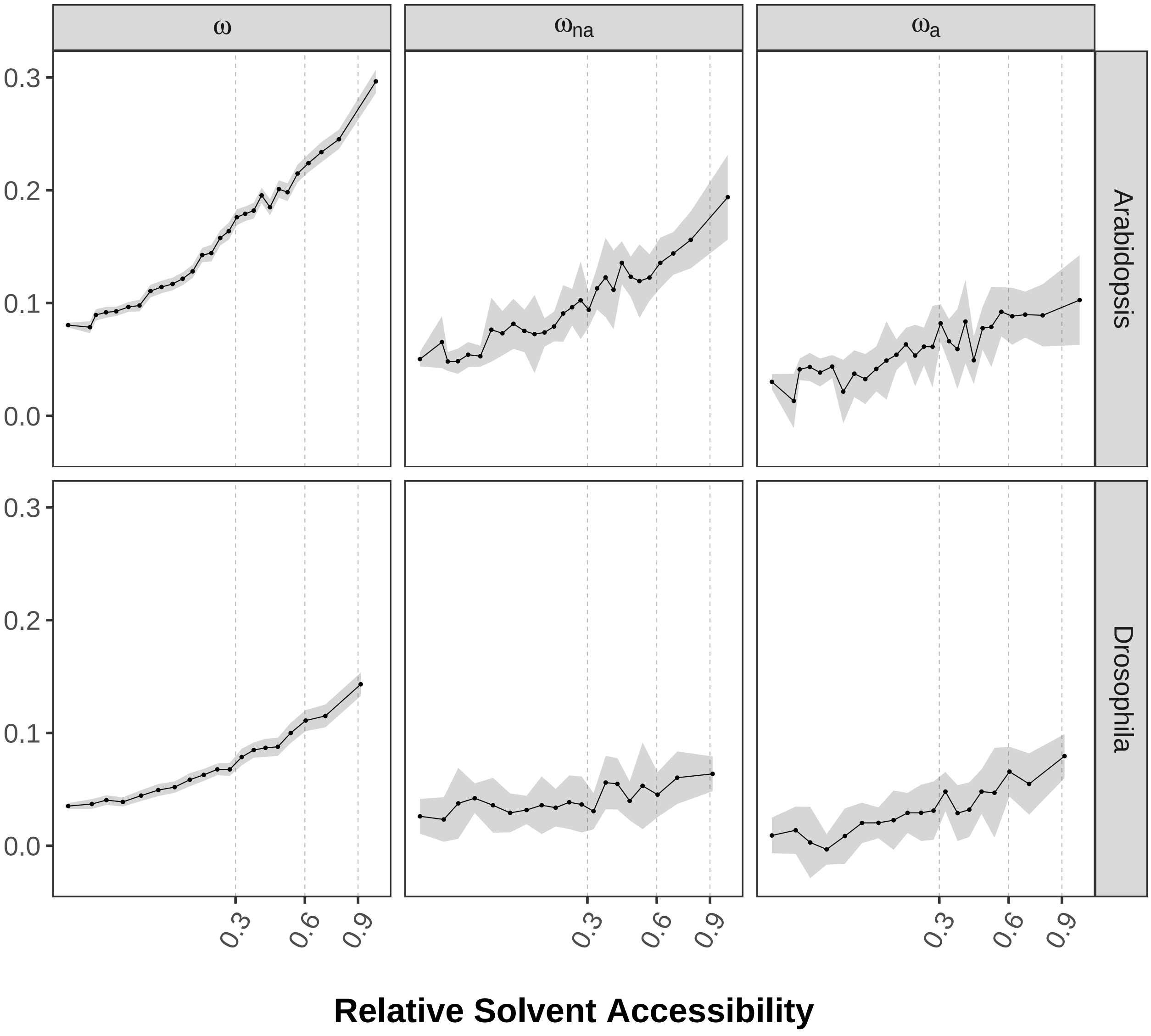

The last section shows how the statistical analyses were performed.

```
stat <- lapply(tbl.stats, function(x) {
  ddply(x, c("var", "species", "variable"), function(x) {
    var <- as.numeric(factor(x$var.value))
    variable.value <- as.numeric(factor(x$value.mean))
    corr = cor.test(var, variable.value, method = "kendall", exact = FALSE)
    Kendall.tau = corr$estimate
    p.value = corr$p.value
    dat = data.frame(Kendall.tau, p.value)
  })
})

# showing the tables
for(i in stat) {
  print(kable(x = i, caption = paste0("Statistics for ", unique(i$var))))
}
```

Table 1: Statistics for Intrinsic Disorder

var	species	variable	Kendall.tau	p.value
Intrinsic Disorder	Arabidopsis	omega	0.9770115	0.0e+00
Intrinsic Disorder	Arabidopsis	omega[na]	0.9172414	0.0e+00
Intrinsic Disorder	Arabidopsis	omega[a]	0.6000000	3.2e-06
Intrinsic Disorder	Drosophila	omega	0.9540230	0.0e+00
Intrinsic Disorder	Drosophila	omega[na]	0.6689655	2.0e-07
Intrinsic Disorder	Drosophila	omega[a]	0.7057471	0.0e+00

Table 2: Statistics for Breadth of Expression

var	species	variable	Kendall.tau	p.value
Breadth of Expression	Arabidopsis	omega	-1.0000000	0.0415401
Breadth of Expression	Arabidopsis	omega[na]	-1.0000000	0.0415401
Breadth of Expression	Arabidopsis	omega[a]	-0.6666667	0.1742314
Breadth of Expression	Drosophila	omega	-0.7333333	0.0387775
Breadth of Expression	Drosophila	omega[na]	-0.4666667	0.1884860
Breadth of Expression	Drosophila	omega[a]	-0.7333333	0.0387775

Table 3: Statistics for Number of Introns

var	species	variable	Kendall.tau	p.value
Number of Introns	Arabidopsis	omega	-0.7948718	0.0001552
Number of Introns	Arabidopsis	omega[na]	-0.3589744	0.0875902
Number of Introns	Arabidopsis	omega[a]	-0.1538462	0.4641035
Number of Introns	Drosophila	omega	-0.7333333	0.0031612
Number of Introns	Drosophila	omega[na]	-0.8666667	0.0004862
Number of Introns	Drosophila	omega[a]	-0.3333333	0.1797125

Table 4: Statistics for Mean Gene Expression

var	species	variable	Kendall.tau	p.value
Mean Gene Expression	Arabidopsis	omega	-0.9282051	0.0000000
Mean Gene Expression	Arabidopsis	omega[na]	-0.9153846	0.0000000
Mean Gene Expression	Arabidopsis	omega[a]	-0.1384615	0.2082790
Mean Gene Expression	Drosophila	omega	-0.7714286	0.0000611
Mean Gene Expression	Drosophila	omega[na]	-0.6190476	0.0012969
Mean Gene Expression	Drosophila	omega[a]	-0.5047619	0.0087205

Table 5: Statistics for Protein-Protein Interactions

var	species	variable	Kendall.tau	p.value
Protein-Protein Interactions	Drosophila	omega	-0.3684211	0.0275179
Protein-Protein Interactions	Drosophila	omega[na]	-0.1111111	0.5062259
Protein-Protein Interactions	Drosophila	omega[a]	-0.3099415	0.0637055

Table 6: Statistics for Proportion Disordered Residues

var	species	variable	Kendall.tau	p.value
Proportion Disordered Residues	Arabidospis	omega	0.7517241	0.0000000
Proportion Disordered Residues	Arabidospis	omega[na]	0.7333333	0.0000000
Proportion Disordered Residues	Arabidospis	omega[a]	0.1908046	0.1386584
Proportion Disordered Residues	Drosophila	omega	0.5684211	0.0004584
Proportion Disordered Residues	Drosophila	omega[na]	0.0631579	0.6970310
Proportion Disordered Residues	Drosophila	omega[a]	0.7263158	0.0000076

Table 7: Statistics for Protein Length

var	species	variable	Kendall.tau	p.value
Protein Length	Arabidopsis	omega	-0.6781609	0.0000001
Protein Length	Arabidopsis	omega[na]	-0.6735632	0.0000002
Protein Length	Arabidopsis	omega[a]	-0.1310345	0.3091826
Protein Length	Drosophila	omega	-0.7763265	0.0000000
Protein Length	Drosophila	omega[na]	-0.6963265	0.0000000
Protein Length	Drosophila	omega[a]	-0.4775510	0.0000010

Table 8: Statistics for Recombination Rate MareyMap

var	species	variable	Kendall.tau	p.value
Recombination Rate MareyMap	Arabidospis	omega	0.0857143	0.3797755
Recombination Rate MareyMap	Arabidospis	omega[na]	-0.2212245	0.0233978
Recombination Rate MareyMap	Arabidospis	omega[a]	0.2065306	0.0343185
Recombination Rate MareyMap	Drosophila	omega	0.0758621	0.5560263
Recombination Rate MareyMap	Drosophila	omega[na]	-0.4022989	0.0017952
Recombination Rate MareyMap	Drosophila	omega[a]	0.3839080	0.0028876

Table 9: Statistics for Relative Solvent Accessibility

var	species	variable	Kendall.tau	p.value
Relative Solvent Accessibility	Arabidopsis	omega	0.9841270	0.0000000
Relative Solvent Accessibility	Arabidopsis	omega[na]	0.8465608	0.0000000
Relative Solvent Accessibility	Arabidopsis	omega[a]	0.7513228	0.0000000
Relative Solvent Accessibility	Drosophila	omega	0.9766082	0.0000000
Relative Solvent Accessibility	Drosophila	omega[na]	0.5789474	0.0005331
Relative Solvent Accessibility	Drosophila	omega[a]	0.8128655	0.0000012
