# Supplementary material for: The Impact of Protein Architecture on Adaptive Evolution
Source: Mol Biol Evol. 2019 May 30;36(9):2013–28. doi: 10.1093/molbev/msz134 (PMC6735723; doi:10.1093/molbev/msz134)
Supplement: msz134_Supplementary_Data [file msz134_supplementary_data.zip › FileS3.pdf]

Discrete variables

This is the script describing all plots and statistical analysis regarding the analysis of discrete variables, refering to: secondary structure motif, binding affinity to dnak and protein location. A function was created to call all files for each analysis performed. In this respect the order of the files is as follows:
files.discrete[1] <- Molecular Chaperone dnaK output table
files.discrete[2] <- Protein Location output table
files.discrete[3] <- Secondary Structure motif output table

The first part of the script removes bootstrap replicates for which the fitness effects parameters were not successfully fitted. For this purpose we discard 1% of the values above the maximum and below the minimum of each of the four parameters of fitness effects: Geman.neg, Gshape.neg, Gmean.neg and prop.pos.

```
setwd("/Users/moutinho/Dropbox/Data/Discrete/")

# Libraries
library(plyr)
library(dplyr)
library(data.table)
library(ggplot2)
library(reshape2)
library(doBy)
library(knitr)
library(kableExtra)
#

# calling all output tables
files.discrete <- list.files(".", ".csv")

# reading each table into a list
tbl.list <- lapply(files.discrete, read.table, header = TRUE)

## remove the outliers: 1% of the replicates below the min and
## 1% above the maximum
sub.tbl <- lapply(tbl.list, function(x) {
  ddply(x, c("species", "var.value"), function(x) {
    sum.gmeanNeg <- summary(x$Gmean.neg)
    gmeanNeg.min1 <- as.numeric(sum.gmeanNeg[1]) + 0.01*as.numeric(sum.gmeanNeg[1])
    gmeanNeg.max1 <- as.numeric(sum.gmeanNeg[6]) - 0.01*as.numeric(sum.gmeanNeg[6])
    sum.gshapeNeg <- summary(x$Gshape.neg)
    gshapeNeg.min1 <- as.numeric(sum.gshapeNeg[1]) + 0.01*as.numeric(sum.gshapeNeg[1])
    gshapeNeg.max1 <- as.numeric(sum.gshapeNeg[6]) - 0.01*as.numeric(sum.gshapeNeg[6])
    sum.gmeanPos <- summary(x$Gmean.pos)
    gmeanPos.min1 <- as.numeric(sum.gmeanPos[1]) + 0.01*as.numeric(sum.gmeanPos[1])
    gmeanPos.max1 <- as.numeric(sum.gmeanPos[6]) - 0.01*as.numeric(sum.gmeanPos[6])
    sum.propPos <- summary(x$prop.pos)
    propPos.min1 <- as.numeric(sum.propPos[1]) + 0.01*as.numeric(sum.propPos[1])
    propPos.max1 <- as.numeric(sum.propPos[6]) - 0.01*as.numeric(sum.propPos[6])
    tbl <- x[!(x$Gmean.neg < gmeanNeg.min1 | x$Gmean.neg > gmeanNeg.max1 &
                 x$Gshape.neg < gshapeNeg.min1 | x$Gshape.neg > gshapeNeg.max1 &
                 x$Gmean.pos < gmeanPos.min1 | x$Gmean.pos > gmeanPos.max1 &
                 x$prop.pos < propPos.min1 | x$prop.pos > propPos.max1),]
  })
```

```
})
```

In the next chunk will take only the estimates concerning the rate of adaptive and non-adaptive substitutions, particularly: dnds, omegaNA and omegaA.

```
# In order to keep only the variables that we want to plot:
tbl.rates <- lapply(sub.tbl, function(x) {
  ddply(x, c("var", "species"), function(x) {
    melt(x, id.vars = c("var.value"), measure.vars = c("dnds", "omegaNA", "omegaA"))
  })
})

# function to estimate the mean and satandard deviation to plot the results with the
# mean of the bootstrap replicates and the 95% confidence interval

fun <- function(x){
  c(mean=mean(x), sd=sd(x))
}

# applying the above function to each output table for each value of each estimate
# (dnds, omegaA, omegaNA) for each value of the variable being analyzed for each species

tbl.stats <- lapply(tbl.rates, function(x) {
  summaryBy(value ~ variable + var.value + species + var, data=x, FUN = fun)
})

# to change the estimate name to the respective symbol
tbl.stats[[1]]$variable <- factor(tbl.stats[[1]]$variable,
                                  levels = c("dnds", "omegaNA", "omegaA"))
levels(tbl.stats[[1]]$variable) <- c(expression(omega), expression(omega[na]),
                                   expression(omega[a]))
tbl.stats[[2]]$variable <- factor(tbl.stats[[2]]$variable,
                                  levels = c("dnds", "omegaNA", "omegaA"))
levels(tbl.stats[[2]]$variable) <- c(expression(omega), expression(omega[na]),
                                   expression(omega[a]))
tbl.stats[[3]]$variable <- factor(tbl.stats[[3]]$variable,
                                  levels = c("dnds", "omegaNA", "omegaA"))
levels(tbl.stats[[3]]$variable) <- c(expression(omega), expression(omega[na]),
                                   expression(omega[a]))
```

The next chunck of the script shows the code used for plotting the results.

```
# theme of the plot
theme.plot <- function(x) {
  theme(axis.title = element_text(face = "bold", color = "black", size=14),
        text = element_text(size=14),
        axis.title.x = element_text(margin = margin(t = 18, r = 10, b = 0, l = 0)),
        axis.title.y = element_text(margin = margin(t = 18, r = 10, b = 0, l = 0)),
        panel.grid.minor=element_blank(),
        panel.grid.major = element_line(colour = "grey", linetype = "dashed", size = 0.2),
        panel.grid.major.y=element_blank(),
        #strip.text.y = element_blank(),
        axis.text.x = element_text(angle = 60, hjust = 1))
}
```

```r
# plotting each of the output tables
plot.discrete <- lapply(tbl.stats, function(x) {
  ggplot(x, aes(x = var.value, y = value.mean)) +
  geom_point(size=0.5, col = "black") +
  geom_errorbar(aes(ymin=value.mean + 1.96*value.sd,
                    ymax=value.mean - 1.96*value.sd), width = .2) +
  facet_grid(species~variable, scales = "free_x", labeller = label_parsed) +
  ylab("") +
  xlab(as.character(x$var)) +
  theme_bw() +
  theme.plot()
})

plot.discrete
```

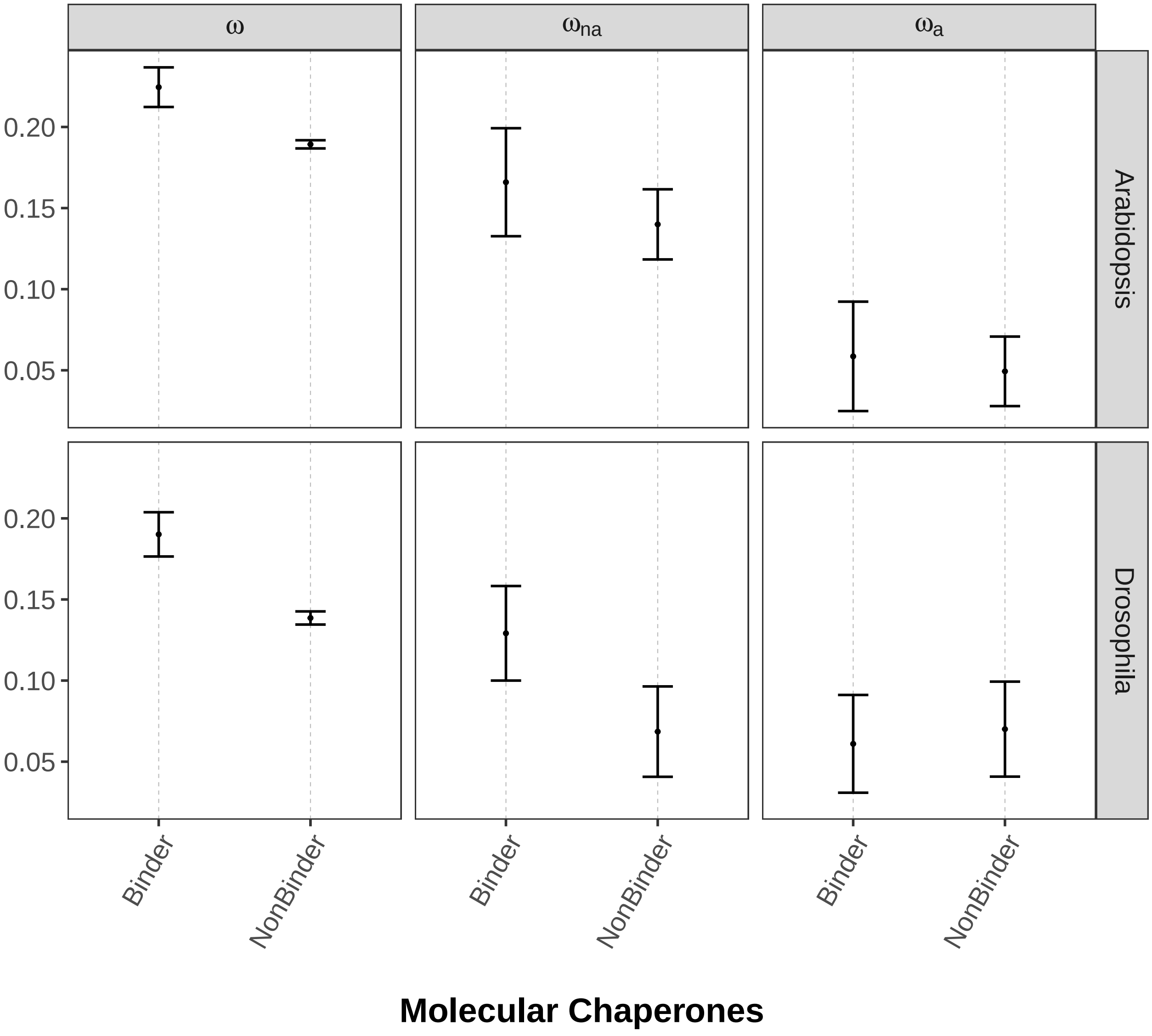

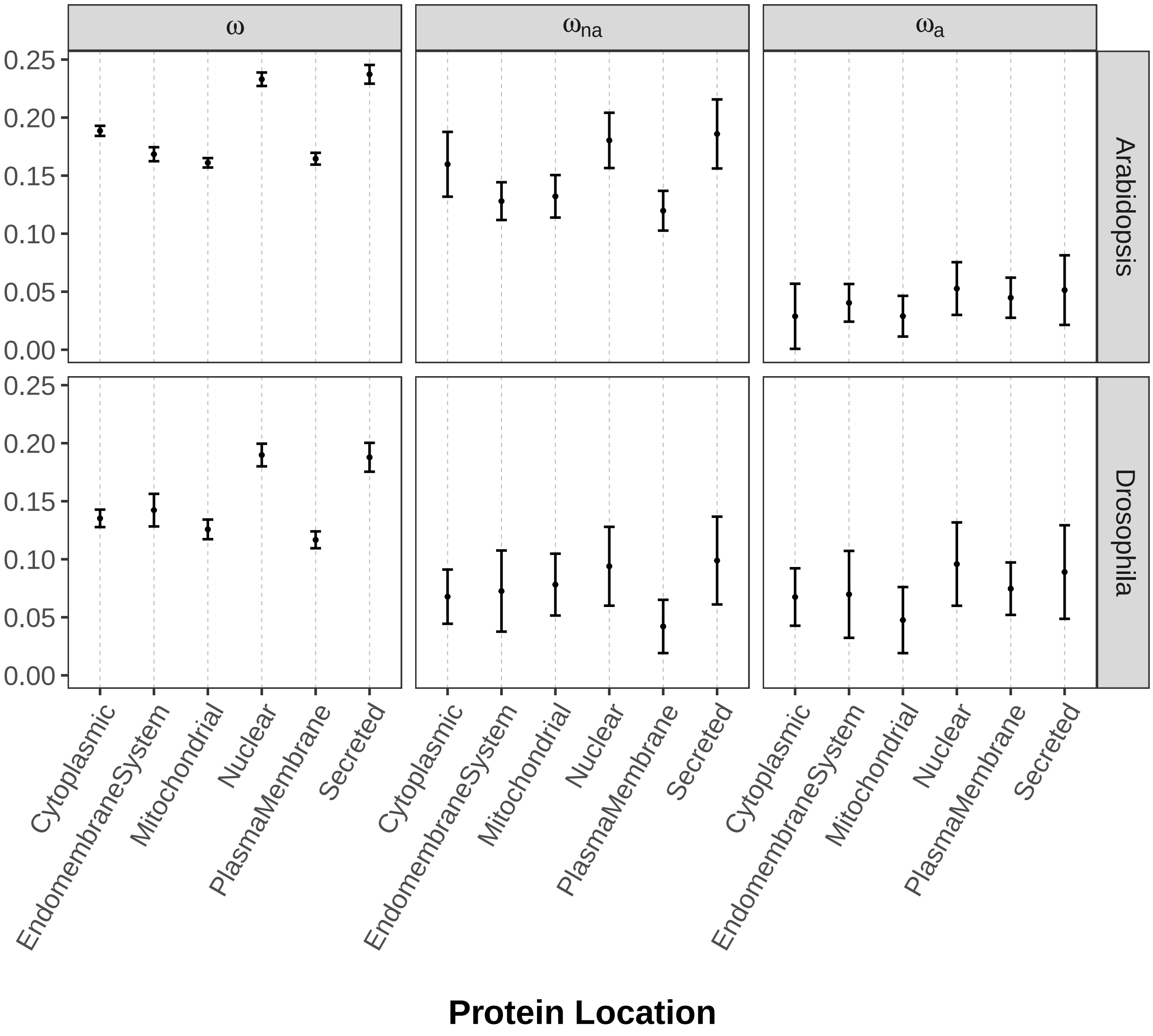
ω
ω$_{na}$
ω$_a$
Arabidopsis
Drosophila
0.25
0.20
0.15
0.10
0.05
0.00
Cytoplasmic
EndomembraneSystem
Mitochondrial
Nuclear
PlasmaMembrane
Secreted
Protein Location

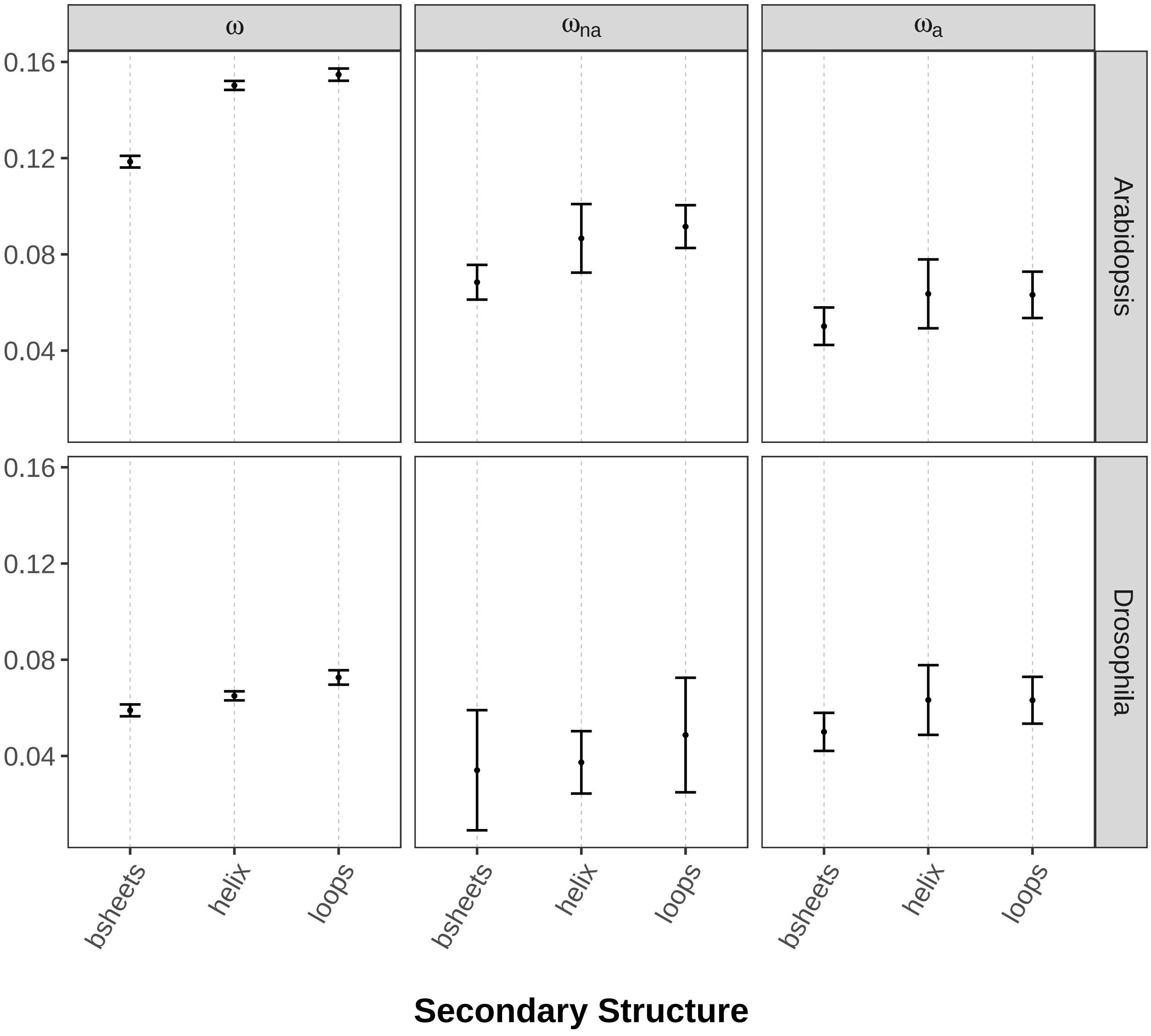

The next chunck of the script describes the statistical tests applied to the variables comparing two categories, specifically:
Binding affinity to dnaK

```
# libraries
library(rlist)
library(stringr)
#

######
###### Binding affinity to dnaK
######

# getting the difference between the mean values of each category
dif.tbl <- ddply(tbl.stats[[1]], c("variable", "species"), function(x) {
  dif <- as.numeric(x$value.mean[x$var.value == "Binder"] -
                          x$value.mean[x$var.value == "NonBinder"])
  data.frame(dif)
})
```

Table 1: Difference between the mean values for each category

variable	species	dif
omega	Arabidopsis	0.0352311
omega	Drosophila	0.0515411
omega[na]	Arabidopsis	0.0259939
omega[na]	Drosophila	0.0606116
omega[a]	Arabidopsis	0.0092371
omega[a]	Drosophila	-0.0090705

```
kable(dif.tbl, caption = "Difference between the mean values for each category")
```

```
# checking the number of replicates per var.value, variable
# and species after removing 1% outliers
dnak.nrep <- ddply(tbl.rates[[1]], c("species", "variable", "var.value"),
                   function(x) {
    nrep <- nrow(x)
    data.frame(nrep)
})

# take the minimum number of replicates between categories
# take the minimum number of replicates between categories
min.nrep <- ddply(dnak.nrep, c("species", "variable"), function(x) {
  min.n <- min(x$nrep)
  data.frame(min.n)
})
kable(min.nrep)
```

species	variable	min.n
Arabidopsis	dnds	91
Arabidopsis	omegaNA	91
Arabidopsis	omegaA	91
Drosophila	dnds	57
Drosophila	omegaNA	57
Drosophila	omegaA	57

```
# 91 replicates Arabidopsis
nrep.arab <- subset(tbl.rates[[1]], tbl.rates[[1]]$species == "Arabidopsis")
tbl.arab <- ddply(nrep.arab, c("species", "var.value", "variable"),
                  function(x) {
  x[sample(nrow(x), 91, replace = FALSE), ]
})

# 57 replicates for Drosophila
nrep.dmel <- subset(tbl.rates[[1]], tbl.rates[[1]]$species == "Drosophila")
tbl.dmel <- ddply(nrep.dmel, c("species", "var.value", "variable"),
                  function(x) {
  x[sample(nrow(x), 57, replace = FALSE), ]
})

dat.dnak <- rbind(tbl.arab, tbl.dmel)

# function to split the tables by the name of each variable
```

```r
dnak.split <- by(dat.dnak, dat.dnak[,c("var.value")], function(y) y)

# to change the column name "value" to the respective id of the category
dnak.value <- lapply(dnak.split, function(x) {
  tbl <- data.frame(x)
  colnames(tbl)[5] <- as.character(unique(tbl$var.value))
  tbl <- tbl[,-3]
  return(tbl)
})

# binding all values as columns
names.vars <- dnak.value[[1]][,1:3]

tbl.estimate <- lapply(dnak.value, function(x) {
  val <- x[,4]
})
tbl.dnak <- list.cbind(tbl.estimate)
tbl.dnak <- as.data.frame(tbl.dnak)
tbl.dnak <- cbind(names.vars, tbl.dnak)

# now lets estimate the differences between the two variables
dnak.dif <- ddply(tbl.dnak, c("variable", "species"), function(x){
  dif <- x$Binder - x$NonBinder
  tbl <- data.frame(dif)
})

# Getting the p-value for each difference. Since we are sampling across
# bootstrap replicates, the p-values will be estimated 100 times and
# then the minimum p-value will be used

# Arabidopsis
arab.dif <- subset(dnak.dif, dnak.dif$species == "Arabidopsis")
nboots <- 100
arab.p <- list()
for (i in 1:nboots) {
  arab.p[[i]] <- ddply(arab.dif, c("variable", "species"), function(x, N=91){
    c <- as.numeric(nrow(x[x$dif < 0,]))
    c2 <- as.numeric(nrow(x[x$dif > 0,]))
    m <- min(c, c2)
    p <- (2*m+1)/(N+1)
    tbl <- data.frame(m, p)
  })
}
arab.p.tbl <- rbindlist(arab.p)
arab.pvalue <- ddply(arab.p.tbl, c("variable", "species"), function(x) {
  p.value <- min(x$p)
  data.frame(p.value)
})

# showing the table
kable(arab.pvalue, caption = "Statistics for the binding affinity to dnaK")
```

```r
# Drosophila
dmel.dif <- subset(dnak.dif, dnak.dif$species == "Drosophila")
```

Table 2: Statistics for the binding affinity to dnaK

variable	species	p.value
dnds	Arabidopsis	0.0108696
omegaNA	Arabidopsis	0.2282609
omegaA	Arabidopsis	0.6630435

Table 3: Statistics for the binding affinity to dnaK

variable	species	p.value
dnds	Drosophila	0.0172414
omegaNA	Drosophila	0.0172414
omegaA	Drosophila	0.7413793

```
nboots <- 100
dmel.p <- list()
for (i in 1:nboots) {
  dmel.p[[i]] <- ddply(dmel.dif, c("variable", "species"), function(x, N=57){
    c <- as.numeric(nrow(x[x$dif < 0,]))
    c2 <- as.numeric(nrow(x[x$dif > 0,]))
    m <- min(c, c2)
    p <- (2*m+1)/(N+1)
    tbl <- data.frame(m, p)
  })
}
dmel.p.tbl <- rbindlist(dmel.p)
dmel.pvalue <- ddply(dmel.p.tbl, c("variable", "species"), function(x) {
  p.value <- min(x$p)
  data.frame(p.value)
})

# showing the table
kable(dmel.pvalue, caption = "Statistics for the binding affinity to dnaK")
```

The next section displays the statistical tests used for variables comparing three or more categories, specifically:
Protein Location
Secondary structure

```
# libraries
library(rlist)
library(stringr)
#

######
###### Protein Location
######

# checking the number of replicates per var.value, variable
# and species after removing 1% outliers
loc.nrep <- ddply(tbl.rates[[2]], c("species", "variable", "var.value"),
                  function(x) {
    nrep <- nrow(x)
```

```
    data.frame(nrep)
})

# take the minimum number of replicates between categories
min.nrep <- ddply(loc.nrep, c("species", "variable"), function(x) {
  min.n <- min(x$nrep)
  data.frame(min.n)
})
kable(min.nrep)
```

species	variable	min.n
Arabidopsis	dnds	77
Arabidopsis	omegaNA	77
Arabidopsis	omegaA	77
Drosophila	dnds	95
Drosophila	omegaNA	95
Drosophila	omegaA	95

```
# 77 replicates Arabidopsis
nrep.arab <- subset(tbl.rates[[2]], tbl.rates[[2]]$species == "Arabidopsis")
tbl.arab <- ddply(nrep.arab, c("species", "var.value", "variable"),
                  function(x) {
  x[sample(nrow(x), 77, replace = FALSE), ]
})

# 95 replicates for Drosophila
nrep.dmel <- subset(tbl.rates[[2]], tbl.rates[[2]]$species == "Drosophila")
tbl.dmel <- ddply(nrep.dmel, c("species", "var.value", "variable"),
                  function(x) {
  x[sample(nrow(x), 95, replace = FALSE), ]
})

dat.loc <- rbind(tbl.arab, tbl.dmel)

# function to split the tables by the name of each variable
loc.split <- by(dat.loc, dat.loc[,c("var.value")], function(y) y)

# to change the column name "value" to the respective id of the category
loc.value <- lapply(loc.split, function(x) {
  tbl <- data.frame(x)
  colnames(tbl)[5] <- as.character(unique(tbl$var.value))
  tbl <- tbl[,-3]
  return(tbl)
})

# binding all values as columns
names.vars <- loc.value[[1]][,1:3]

tbl.estimate <- lapply(loc.value, function(x) {
  val <- x[,4]
})
tbl.loc <- list.cbind(tbl.estimate)
tbl.loc <- as.data.frame(tbl.loc)
tbl.loc <- cbind(tbl.loc, names.vars)
```

```r
colnames(tbl.loc)[9] <- "estimate"

# estimate the differences between columns
# to do so will duplicate the data.frame in order to subtract each column
tbl2.loc <- tbl.loc[,1:6]

# doing the differences in a way that we count only for one of the differences
loc.dif <- cbind(tbl.loc[, c(8,9), drop=F],
        do.call(cbind, lapply(tbl2.loc[,2:6],
                              function(x) tbl.loc[,1:2]-x)),
        do.call(cbind, lapply(tbl2.loc[,4:6],
                              function(x) tbl.loc[,3:4]-x)))


# putting all variables in one column
# not counting with the columns comparing the same value
loc.hist <- melt(loc.dif, id.vars = c("estimate", "species"),
                 measure.vars = c(names(loc.dif[3]), names(loc.dif[5:13]),
                                  names(loc.dif[15:18])))

# puting NA values for when the difference is 0
# (i.e. when comparing the same variables)
split <- str_split_fixed(loc.hist$variable, "\\.", 2)
loc.hist$var1 <- split[,1]
loc.hist$var2 <- split[,2]

## getting the p-value for each difference. Since we are sampling across
# bootstrap replicates, the p-values will be estimated 100 times and
# then the minimum p-value will be used

## Arabidopsis
arab.hist <- subset(loc.hist, loc.hist$species == "Arabidopsis")
nboots <- 100
arab.p <- list()
for (i in 1:nboots) {
  arab.p[[i]] <- ddply(arab.hist, c("species", "estimate", "var1", "var2"),
                       function(x, N=77){
    c <- as.numeric(nrow(x[x$value < 0,]))
    c2 <- as.numeric(nrow(x[x$value > 0,]))
    m <- min(c, c2)
    p <- (2*m+1)/(N+1)
    tbl <- data.frame(m, p)
  })
}
# correcting the p-value for multiple testing
arab.p.adj <- lapply(arab.p, function(x) {
  ddply(x,  c("species","estimate", "var1", "var2"), function(x) {
    p.value <- p.adjust(x$p)
    data.frame(p.value)
  })
})

# taking the minimum p-value
```

```
tbl.arab.p.adj <- rbindlist(arab.p.adj)
arab.pvalue <- ddply(tbl.arab.p.adj, c("species","estimate", "var1", "var2"),
                     function(x) {
  p.value <- min(x$p.value)
  data.frame(p.value)
})

# showing the table
kable(arab.pvalue, caption= "Statistics for protein location")
```

```
## Drosophila
dmel.hist <- subset(loc.hist, loc.hist$species == "Drosophila")
nboots <- 100
dmel.p <- list()
for (i in 1:100) {
  dmel.p[[i]] <- ddply(dmel.hist, c("species", "estimate", "var1", "var2"),
                       function(x, N=95){
    c <- as.numeric(nrow(x[x$value < 0,]))
    c2 <- as.numeric(nrow(x[x$value > 0,]))
    m <- min(c, c2)
    p <- (2*m+1)/(N+1)
    tbl <- data.frame(m, p)
  })
}

## correcting the p-value for multiple testing
dmel.p.adj <- lapply(dmel.p, function(x) {
  ddply(x,  c("species","estimate", "var1", "var2"),
        function(x) {
    p.value <- p.adjust(x$p)
    data.frame(p.value)
  })
})

# taking the minimum p-value
tbl.dmel.p.adj <- rbindlist(dmel.p.adj)
dmel.pvalue <- ddply(tbl.dmel.p.adj, c("species","estimate", "var1", "var2"),
                     function(x) {
  p.value <- min(x$p.value)
  data.frame(p.value)
})

# showing the table
kable(dmel.pvalue, caption = "Statistics for protein location")
```

```
######
###### Secondary Structure
######

# getting the difference between the mean values of each category
dif.ss <- ddply(tbl.stats[[3]], c("variable", "species"), function(x) {
  bsheets.loops <- as.numeric(x$value.mean[x$var.value == "bsheets"] -
                      x$value.mean[x$var.value == "loops"])
  helix.loops <- as.numeric(x$value.mean[x$var.value == "helix"] -
```

Table 4: Statistics for protein location

species	estimate	var1	var2	p.value
Arabidopsis	dnds	EndomembraneSystem	Cytoplasmic	0.0128205
Arabidopsis	dnds	Mitochondrial	Cytoplasmic	0.0128205
Arabidopsis	dnds	Mitochondrial	EndomembraneSystem	0.0641026
Arabidopsis	dnds	Nuclear	Cytoplasmic	0.0128205
Arabidopsis	dnds	Nuclear	EndomembraneSystem	0.0128205
Arabidopsis	dnds	Nuclear	Mitochondrial	0.0128205
Arabidopsis	dnds	PlasmaMembrane	Cytoplasmic	0.0128205
Arabidopsis	dnds	PlasmaMembrane	EndomembraneSystem	0.4487179
Arabidopsis	dnds	PlasmaMembrane	Mitochondrial	0.2948718
Arabidopsis	dnds	PlasmaMembrane	Nuclear	0.0128205
Arabidopsis	dnds	Secreted	Cytoplasmic	0.0128205
Arabidopsis	dnds	Secreted	EndomembraneSystem	0.0128205
Arabidopsis	dnds	Secreted	Mitochondrial	0.0128205
Arabidopsis	dnds	Secreted	Nuclear	0.3717949
Arabidopsis	omegaNA	EndomembraneSystem	Cytoplasmic	0.0384615
Arabidopsis	omegaNA	Mitochondrial	Cytoplasmic	0.1410256
Arabidopsis	omegaNA	Mitochondrial	EndomembraneSystem	0.7051282
Arabidopsis	omegaNA	Nuclear	Cytoplasmic	0.1923077
Arabidopsis	omegaNA	Nuclear	EndomembraneSystem	0.0128205
Arabidopsis	omegaNA	Nuclear	Mitochondrial	0.0128205
Arabidopsis	omegaNA	PlasmaMembrane	Cytoplasmic	0.0128205
Arabidopsis	omegaNA	PlasmaMembrane	EndomembraneSystem	0.5769231
Arabidopsis	omegaNA	PlasmaMembrane	Mitochondrial	0.3461538
Arabidopsis	omegaNA	PlasmaMembrane	Nuclear	0.0128205
Arabidopsis	omegaNA	Secreted	Cytoplasmic	0.2948718
Arabidopsis	omegaNA	Secreted	EndomembraneSystem	0.0128205
Arabidopsis	omegaNA	Secreted	Mitochondrial	0.0384615
Arabidopsis	omegaNA	Secreted	Nuclear	0.7307692
Arabidopsis	omegaA	EndomembraneSystem	Cytoplasmic	0.5256410
Arabidopsis	omegaA	Mitochondrial	Cytoplasmic	0.9102564
Arabidopsis	omegaA	Mitochondrial	EndomembraneSystem	0.2692308
Arabidopsis	omegaA	Nuclear	Cytoplasmic	0.2179487
Arabidopsis	omegaA	Nuclear	EndomembraneSystem	0.4743590
Arabidopsis	omegaA	Nuclear	Mitochondrial	0.1410256
Arabidopsis	omegaA	PlasmaMembrane	Cytoplasmic	0.3717949
Arabidopsis	omegaA	PlasmaMembrane	EndomembraneSystem	0.8846154
Arabidopsis	omegaA	PlasmaMembrane	Mitochondrial	0.1923077
Arabidopsis	omegaA	PlasmaMembrane	Nuclear	0.5512821
Arabidopsis	omegaA	Secreted	Cytoplasmic	0.2435897
Arabidopsis	omegaA	Secreted	EndomembraneSystem	0.5000000
Arabidopsis	omegaA	Secreted	Mitochondrial	0.1666667
Arabidopsis	omegaA	Secreted	Nuclear	0.9871795

Table 5: Statistics for protein location

species	estimate	var1	var2	p.value
Drosophila	dnds	EndomembraneSystem	Cytoplasmic	0.4062500
Drosophila	dnds	Mitochondrial	Cytoplasmic	0.0729167
Drosophila	dnds	Mitochondrial	EndomembraneSystem	0.0520833
Drosophila	dnds	Nuclear	Cytoplasmic	0.0104167
Drosophila	dnds	Nuclear	EndomembraneSystem	0.0104167
Drosophila	dnds	Nuclear	Mitochondrial	0.0104167
Drosophila	dnds	PlasmaMembrane	Cytoplasmic	0.0104167
Drosophila	dnds	PlasmaMembrane	EndomembraneSystem	0.0312500
Drosophila	dnds	PlasmaMembrane	Mitochondrial	0.1562500
Drosophila	dnds	PlasmaMembrane	Nuclear	0.0104167
Drosophila	dnds	Secreted	Cytoplasmic	0.0104167
Drosophila	dnds	Secreted	EndomembraneSystem	0.0104167
Drosophila	dnds	Secreted	Mitochondrial	0.0104167
Drosophila	dnds	Secreted	Nuclear	0.7604167
Drosophila	omegaNA	EndomembraneSystem	Cytoplasmic	0.9479167
Drosophila	omegaNA	Mitochondrial	Cytoplasmic	0.4270833
Drosophila	omegaNA	Mitochondrial	EndomembraneSystem	0.9270833
Drosophila	omegaNA	Nuclear	Cytoplasmic	0.2187500
Drosophila	omegaNA	Nuclear	EndomembraneSystem	0.2812500
Drosophila	omegaNA	Nuclear	Mitochondrial	0.3437500
Drosophila	omegaNA	PlasmaMembrane	Cytoplasmic	0.2395833
Drosophila	omegaNA	PlasmaMembrane	EndomembraneSystem	0.1770833
Drosophila	omegaNA	PlasmaMembrane	Mitochondrial	0.0520833
Drosophila	omegaNA	PlasmaMembrane	Nuclear	0.0520833
Drosophila	omegaNA	Secreted	Cytoplasmic	0.0937500
Drosophila	omegaNA	Secreted	EndomembraneSystem	0.3645833
Drosophila	omegaNA	Secreted	Mitochondrial	0.4062500
Drosophila	omegaNA	Secreted	Nuclear	0.9479167
Drosophila	omegaA	EndomembraneSystem	Cytoplasmic	0.9895833
Drosophila	omegaA	Mitochondrial	Cytoplasmic	0.2604167
Drosophila	omegaA	Mitochondrial	EndomembraneSystem	0.3854167
Drosophila	omegaA	Nuclear	Cytoplasmic	0.2187500
Drosophila	omegaA	Nuclear	EndomembraneSystem	0.3437500
Drosophila	omegaA	Nuclear	Mitochondrial	0.0520833
Drosophila	omegaA	PlasmaMembrane	Cytoplasmic	0.4270833
Drosophila	omegaA	PlasmaMembrane	EndomembraneSystem	0.8854167
Drosophila	omegaA	PlasmaMembrane	Mitochondrial	0.2604167
Drosophila	omegaA	PlasmaMembrane	Nuclear	0.1562500
Drosophila	omegaA	Secreted	Cytoplasmic	0.4687500
Drosophila	omegaA	Secreted	EndomembraneSystem	0.5104167
Drosophila	omegaA	Secreted	Mitochondrial	0.1770833
Drosophila	omegaA	Secreted	Nuclear	0.9270833

Table 6: Difference between the mean values for each category

variable	species	bsheets.helix	bsheets.loops	helix.loops
omega	Arabidopsis	-0.0317002	-0.0361829	-0.0044828
omega	Drosophila	-0.0060471	-0.0136806	-0.0076335
omega[na]	Arabidopsis	-0.0182438	-0.0231339	-0.0048901
omega[na]	Drosophila	-0.0032633	-0.0146317	-0.0113684
omega[a]	Arabidopsis	-0.0134564	-0.0130490	0.0004074
omega[a]	Drosophila	-0.0132371	-0.0131432	0.0000939

```
                              x$value.mean[x$var.value == "loops"])
  bsheets.helix <- as.numeric(x$value.mean[x$var.value == "bsheets"] -
                                    x$value.mean[x$var.value == "helix"])
  data.frame(bsheets.helix, bsheets.loops, helix.loops)
})
kable(dif.ss, caption = "Difference between the mean values for each category")
```

```
# checking the number of replicates per var.value,
# variable and species after removing 1% outliers
SS.nrep <- ddply(tbl.rates[[3]], c("species", "variable", "var.value"),
                 function(x) {
    nrep <- nrow(x)
    data.frame(nrep)
})

# take the minimum number of replicates between categories
min.nrep <- ddply(SS.nrep, c("species", "variable"),
                  function(x) {
  min.n <- min(x$nrep)
  data.frame(min.n)
})
kable(min.nrep)
```

species	variable	min.n
Arabidopsis	dnds	98
Arabidopsis	omegaNA	98
Arabidopsis	omegaA	98
Drosophila	dnds	95
Drosophila	omegaNA	95
Drosophila	omegaA	95

```
# 98 replicates Arabidopsis
nrep.arab <- subset(tbl.rates[[3]], tbl.rates[[3]]$species == "Arabidopsis")
tbl.arab <- ddply(nrep.arab, c("species", "var.value", "variable"),
                  function(x) {
  x[sample(nrow(x), 98), ]
})

# 95 replicates for Drosophila
nrep.dmel <- subset(tbl.rates[[3]], tbl.rates[[3]]$species == "Drosophila")
tbl.dmel <- ddply(nrep.dmel, c("species", "var.value", "variable"),
                  function(x) {
  x[sample(nrow(x), 95), ]
```

```r
})

dat.SS <- rbind(tbl.arab, tbl.dmel)

# function to split the tables by the name of each variable
SS.split <- by(dat.SS, dat.SS[,c("var.value")], function(y) y)

# to change the column name "value" to the respective id of the category
SS.value <- lapply(SS.split, function(x) {
  tbl <- data.frame(x)
  colnames(tbl)[5] <- as.character(unique(tbl$var.value))
  tbl <- tbl[,-3]
  return(tbl)
})

# binding all values as columns
names.vars <- SS.value[[1]][,1:3]

tbl.estimate <- lapply(SS.value, function(x) {
  val <- x[,4]
})
tbl.SS <- list.cbind(tbl.estimate)
tbl.SS <- as.data.frame(tbl.SS)
tbl.SS <- cbind(tbl.SS, names.vars)
colnames(tbl.SS)[6] <- "estimate"

# estimate the differences between columns
# to do so will duplicate the data.frame in order to subtract each column
tbl2.SS <- tbl.SS[,1:3]

SS.dif <- cbind(tbl.SS[, c(5,6), drop=F],
        do.call(cbind, lapply(tbl2.SS[,1:2],
                              function(x) tbl.SS[,2:3]-x)))

# putting all variables in one column
SS.hist <- melt(SS.dif, id.vars = c("estimate", "species"),
                     measure.vars = c(names(SS.dif[3:4]),
                                      names(SS.dif[6])))

# puting NA values for when the difference is 0
# (i.e. when comparing the same variables)
split <- str_split_fixed(SS.hist$variable, "\\.", 2)
SS.hist$var1 <- split[,2]
SS.hist$var2 <- split[,1]

## getting the p-value for each difference
## N is not 100 because we took out the values that were outliers
## Arabidopsis
arab.hist <- subset(SS.hist, SS.hist$species == "Arabidopsis")
nboots <- 100
arab.p <- list()
for (i in 1:100) {
  arab.p[[i]] <- ddply(arab.hist, c("species","estimate", "var1", "var2"),
```

Table 7: Statistics for secondary structure

species	estimate	var1	var2	p.value
Arabidopsis	dnds	helix	bsheets	0.0101010
Arabidopsis	dnds	loops	bsheets	0.0101010
Arabidopsis	dnds	loops	helix	0.0101010
Arabidopsis	omegaNA	helix	bsheets	0.0505051
Arabidopsis	omegaNA	loops	bsheets	0.0101010
Arabidopsis	omegaNA	loops	helix	0.7373737
Arabidopsis	omegaA	helix	bsheets	0.0303030
Arabidopsis	omegaA	loops	bsheets	0.0303030
Arabidopsis	omegaA	loops	helix	0.8181818

```
                       function(x, N=98){
    c <- as.numeric(nrow(x[x$value < 0,]))
    c2 <- as.numeric(nrow(x[x$value > 0,]))
    m <- min(c, c2)
    p <- (2*m+1)/(N+1)
    tbl <- data.frame(c, c2, m, p)
  })
}

# correcting the p-value for multiple testing
arab.p.adj <- lapply(arab.p, function(x) {
  ddply(x,  c("species","estimate", "var1", "var2"),
        function(x) {
    p.value <- p.adjust(x$p)
    data.frame(p.value)
  })
})

# taking the minimum p-value
tbl.arab.p.adj <- rbindlist(arab.p.adj)
arab.pvalue <- ddply(tbl.arab.p.adj, c("species","estimate", "var1", "var2"),
                     function(x) {
  p.value <- min(x$p.value)
  data.frame(p.value)
})

# showing the table
kable(arab.pvalue, caption = "Statistics for secondary structure")
```

```
## Drosophila
dmel.hist <- subset(SS.hist, SS.hist$species == "Drosophila")
nboots <- 100
dmel.p <- list()
for (i in 1:nboots) {
  dmel.p[[i]] <- ddply(dmel.hist, c("species","estimate", "var1", "var2"),
                       function(x, N=95){
    c <- as.numeric(nrow(x[x$value < 0,]))
    c2 <- as.numeric(nrow(x[x$value > 0,]))
    m <- min(c, c2)
```

Table 8: Statistics for secondary structure

species	estimate	var1	var2	p.value
Drosophila	dnds	helix	bsheets	0.0104167
Drosophila	dnds	loops	bsheets	0.0104167
Drosophila	dnds	loops	helix	0.0104167
Drosophila	omegaNA	helix	bsheets	0.8645833
Drosophila	omegaNA	loops	bsheets	0.3854167
Drosophila	omegaNA	loops	helix	0.4270833
Drosophila	omegaA	helix	bsheets	0.0729167
Drosophila	omegaA	loops	bsheets	0.0520833
Drosophila	omegaA	loops	helix	0.8854167

```
    p <- (2*m+1)/(N+1)
    tbl <- data.frame(c, c2, m, p)
  })
}

# correcting the p-value for multiple testing
dmel.p.adj <- lapply(dmel.p, function(x) {
  ddply(x,  c("species","estimate", "var1", "var2"), function(x) {
    p.value <- p.adjust(x$p)
    tbl <- data.frame(p.value)
  })
})

# taking the minimum p-value
tbl.dmel.p.adj <- rbindlist(dmel.p.adj)
dmel.pvalue <- ddply(tbl.dmel.p.adj, c("species","estimate", "var1", "var2"),
                     function(x) {
  p.value <- min(x$p.value)
  data.frame(p.value)
})

# showing the table
kable(dmel.pvalue, caption = "Statistics for secondary structure")
```
