# Supplementary material for: The Impact of Protein Architecture on Adaptive Evolution
Source: Mol Biol Evol. 2019 May 30;36(9):2013–28. doi: 10.1093/molbev/msz134 (PMC6735723; doi:10.1093/molbev/msz134)
Supplement: msz134_Supplementary_Data [file msz134_supplementary_data.zip › FileS4.pdf]

Protein Functional Class

This notebook describes all plots and statistical analysis performed with the categories of protein functional class.

The first part of the script removes bootstrap replicates for which the fitness effects parameters were not successfully fitted. For this purpose we discard 1% of the values above the maximum and below the minimum of each of the four parameters of fitness effects: Geman.neg, Gshape.neg, Gmean.neg and prop.pos.

```
setwd("/Users/moutinho/Dropbox/Data/Discrete/FunctionalClass/")

# Libraries
library(plyr)
library(dplyr)
library(data.table)
library(ggplot2)
library(reshape2)
library(doBy)
library(knitr)
library(kableExtra)
#

# calling all output tables
tbl.keggsmall <- read.table(file = "SmallProteinClass.csv", sep = "\t", header = TRUE)

## remove the outliers: 1% of the replicates below the min and
## 1% above the maximum

sub.keggSmall <- ddply(tbl.keggsmall, c("species", "var.value"), function(x) {
  sum.gmeanNeg <- summary(x$Gmean.neg)
  gmeanNeg.min1 <- as.numeric(sum.gmeanNeg[1]) + 0.01*as.numeric(sum.gmeanNeg[1])
  gmeanNeg.max1 <- as.numeric(sum.gmeanNeg[6]) - 0.01*as.numeric(sum.gmeanNeg[6])
  sum.gshapeNeg <- summary(x$Gshape.neg)
  gshapeNeg.min1 <- as.numeric(sum.gshapeNeg[1]) + 0.01*as.numeric(sum.gshapeNeg[1])
  gshapeNeg.max1 <- as.numeric(sum.gshapeNeg[6]) - 0.01*as.numeric(sum.gshapeNeg[6])
  sum.gmeanPos <- summary(x$Gmean.pos)
  gmeanPos.min1 <- as.numeric(sum.gmeanPos[1]) + 0.01*as.numeric(sum.gmeanPos[1])
  gmeanPos.max1 <- as.numeric(sum.gmeanPos[6]) - 0.01*as.numeric(sum.gmeanPos[6])
  sum.propPos <- summary(x$prop.pos)
  propPos.min1 <- as.numeric(sum.propPos[1]) + 0.01*as.numeric(sum.propPos[1])
  propPos.max1 <- as.numeric(sum.propPos[6]) - 0.01*as.numeric(sum.propPos[6])
  tbl <- x[!(x$Gmean.neg < gmeanNeg.min1 | x$Gmean.neg > gmeanNeg.max1 &
             x$Gshape.neg < gshapeNeg.min1 | x$Gshape.neg > gshapeNeg.max1 &
             x$Gmean.pos < gmeanPos.min1 | x$Gmean.pos > gmeanPos.max1 &
             x$prop.pos < propPos.min1 | x$prop.pos > propPos.max1),]
})
```

In the next chunk will take only the estimates concerning the rate of adaptive and non-adaptive substitutions, particularly: dnds, omegaNA and omegaA.

```
# In order to keep only the variables that we want to plot:
tbl.rates <- ddply(sub.keggSmall, c("species"), function(x) {
    melt(x, id.vars = c("var.value"), measure.vars = c("dnds", "omegaNA", "omegaA"))
})
```

```
# function to estimate the mean and satandard deviation to plot the results with the
# mean of the bootstrap replicates and the 95% confidence interval

fun <- function(x){
  c(mean=mean(x), sd=sd(x))
}

# applying the above function to each output table for each value of each estimate
# (dnds, omegaA, omegaNA) for each value of the variable being analyzed for each species

tbl.stats <- summaryBy(value ~ variable + var.value + species, data=tbl.rates, FUN = fun)

# to change the estimate name to the respective symbol
tbl.stats$variable <- factor(tbl.stats$variable, levels = c("dnds", "omegaNA", "omegaA"))
levels(tbl.stats$variable) <- c(expression(omega), expression(omega[na]),
                                   expression(omega[a]))
```

The next chunck of the script shows the code used for plotting the results. The plots were done separately for the two species because the categories analysed did not overlap completely.

```
# theme of the plot
theme.plot <- function(x) {
  theme(axis.title = element_text(face = "bold", color = "black", size=12,
                                  family = "Times"),
        text = element_text(size=12),
        axis.title.x = element_text(margin = margin(t = 9, r = 18, b = 5, l = 5)),
        axis.title.y = element_text(margin = margin(t = 9, r = 18, b = 5, l = 5)),
        panel.grid.minor=element_blank(),
        panel.grid.major = element_line(colour = "grey", linetype = "dashed", size = 0.1),
        panel.grid.major.y=element_blank(),
        strip.text.y = element_blank(),
        axis.text.x = element_text(angle = 60, hjust = 1),
        strip.background.x = element_rect(colour = "grey", fill = "gray92"),
        panel.spacing = unit(0.75, "lines"))
}

###
### Arabidopsis
###

stat.arab <- subset(tbl.stats, tbl.stats$species == "Arabidopsis")

# to order the plot according to values of omegaA
omegaA.arab <- subset(stat.arab, stat.arab$variable == "omega[a]")
omegaA.arab <- omegaA.arab[order(omegaA.arab[,4]),]

plot.arab <- ggplot(stat.arab, aes(x = var.value, y = value.mean)) +
  geom_point(size=1, col = "black") +
  geom_errorbar(aes(ymin=value.mean + 1.96*value.sd,
                    ymax=value.mean - 1.96*value.sd), width = .2) +
  scale_x_discrete(limits = as.character(omegaA.arab$var.value)) +
  ylab("") +
  xlab("Protein Functional Classification") +
  facet_grid(~variable, labeller = label_parsed) +
```

```r
  theme_bw() +
  theme.plot() +
  coord_flip()

plot.arab
```

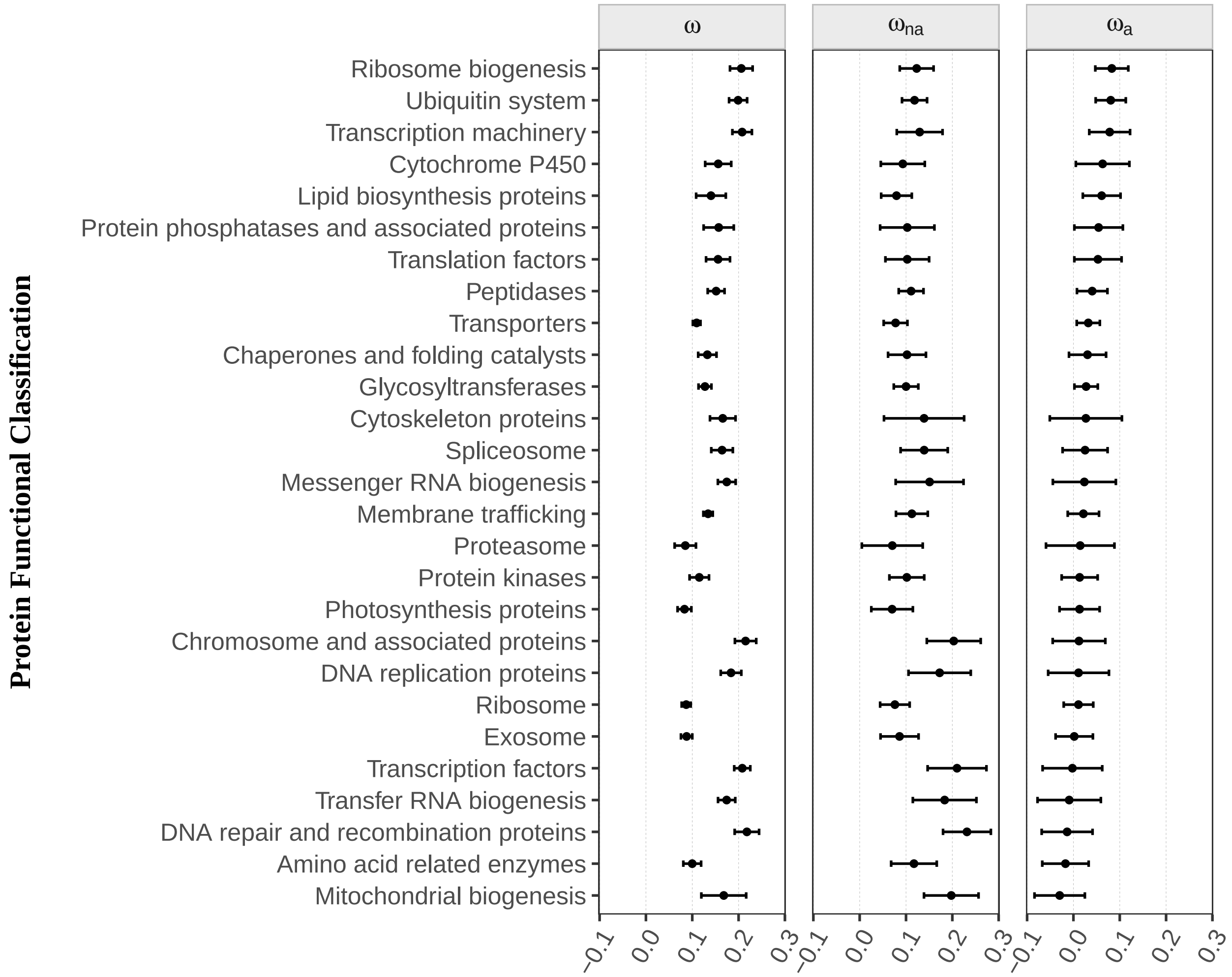


```r
###
### Drosophila
###

stat.dmel <- subset(tbl.stats, tbl.stats$species == "Drosophila")

# to order the plot according to values of omegaA
omegaA.dmel <- subset(stat.dmel, stat.dmel$variable == "omega[a]")
omegaA.dmel <- omegaA.dmel[order(omegaA.dmel[,4]),]

plot.dmel <- ggplot(stat.dmel, aes(x = var.value, y = value.mean)) +
  geom_point(size=1, col = "black") +
  geom_errorbar(aes(ymin=value.mean + 1.96*value.sd,
                    ymax=value.mean - 1.96*value.sd), width = .2) +
  scale_x_discrete(limits = as.character(omegaA.dmel$var.value)) +
  ylab("") +
```

```
  xlab("Protein Functional Classification") +
  scale_y_continuous(limits = c(-0.1, 0.30)) +
  facet_grid(~variable, labeller = label_parsed) +
  theme_bw() +
  theme.plot() +
  coord_flip()

plot.dmel
```

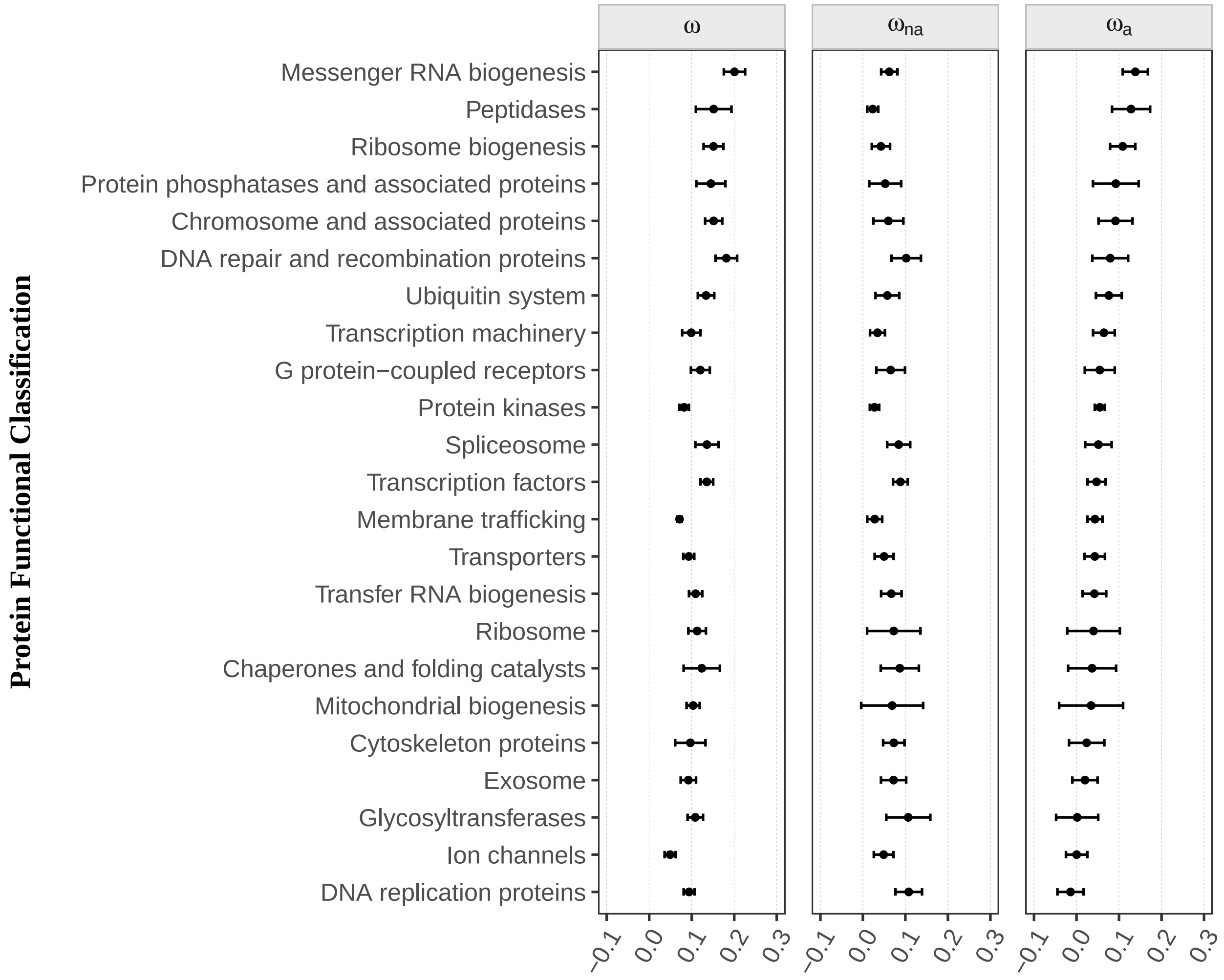

The next chunck of the script describes the statistical analysis.

```
# libraries
library(rlist)
library(stringr)
library(rowr)
#

# checking the number of replicates per var.value, variable and
# species after removing 1% outliers
kegg.nrep <- ddply(tbl.rates, c("species", "variable", "var.value"),
                   function(x) {
    nrep <- nrow(x)
```

```r
    data.frame(nrep)
})

# take the minimum number of replicates between categories
min.nrep <- ddply(kegg.nrep, c("species", "variable"), function(x) {
  min.n <- min(x$nrep)
  data.frame(min.n)
})
min.nrep
```

species	variable	min.n
Arabidopsis	dnds	28
Arabidopsis	omegaNA	28
Arabidopsis	omegaA	28
Drosophila	dnds	53
Drosophila	omegaNA	53
Drosophila	omegaA	53

```r
# 28 replicates for Arabidopsis
nrep.arab <- subset(tbl.rates, tbl.rates$species == "Arabidopsis")
tbl.arab <- ddply(nrep.arab, c("species", "var.value", "variable"),
                  function(x) {
  x[sample(nrow(x), 28), ]
})

# 53 replicates for Drosophila
nrep.dmel <- subset(tbl.rates, tbl.rates$species == "Drosophila")
tbl.dmel <- ddply(nrep.dmel, c("species", "var.value", "variable"),
                  function(x) {
  x[sample(nrow(x), 53), ]
})

dat.kegg <- rbind(tbl.arab, tbl.dmel)

# function to split the tables by the name of each variable
kegg.split <- by(dat.kegg, dat.kegg[,c("var.value")], function(y) y)

# to change the column name "value" to the respective id of the category
kegg.value <- lapply(kegg.split, function(x) {
  tbl <- data.frame(x)
  colnames(tbl)[4] <- as.character(unique(tbl$var.value))
  tbl <- tbl[,-2]
  return(tbl)
})

# checking which category has the maximum rows to combine all variables
row.n <- lapply(kegg.value, function(x) {
  nrow(x)
})

species <- kegg.value[[2]][,1]
estimate <- kegg.value[[2]][,2]

tbl.estimate <- lapply(kegg.value, function(x) {
```

```
  val <- x[,3]
})

# binding all values as columns
tbl.kegg <- list.cbind(tbl.estimate)
tbl.kegg <- as.data.frame(tbl.kegg)
tbl.kegg <- data.frame(tbl.kegg, species, estimate, fix.empty.names = TRUE)
names(tbl.kegg) <- gsub(x = names(tbl.kegg), pattern = "\\.", replacement = "_")

# estimate the differences between columns
# to do so will duplicate the data.frame in order to subtract each column
tbl2.kegg <- tbl.kegg[,1:29]

# doing the differences in a way that we count only for one of the differences
kegg.dif <- cbind(tbl.kegg[, c(30,31), drop=F],
        do.call(cbind, lapply(tbl2.kegg[,2:29],
                              function(x) tbl.kegg[,1:2]-x)),
        do.call(cbind, lapply(tbl2.kegg[,4:29],
                              function(x) tbl.kegg[,3:4]-x)),
        do.call(cbind, lapply(tbl2.kegg[,6:29],
                              function(x) tbl.kegg[,5:6]-x)),
        do.call(cbind, lapply(tbl2.kegg[,8:29],
                              function(x) tbl.kegg[,7:8]-x)),
        do.call(cbind, lapply(tbl2.kegg[,10:29],
                              function(x) tbl.kegg[,9:10]-x)),
        do.call(cbind, lapply(tbl2.kegg[,12:29],
                              function(x) tbl.kegg[,11:12]-x)),
        do.call(cbind, lapply(tbl2.kegg[,14:29],
                              function(x) tbl.kegg[,13:14]-x)),
        do.call(cbind, lapply(tbl2.kegg[,16:29],
                              function(x) tbl.kegg[,15:16]-x)),
        do.call(cbind, lapply(tbl2.kegg[,18:29],
                              function(x) tbl.kegg[,17:18]-x)),
        do.call(cbind, lapply(tbl2.kegg[,20:29],
                              function(x) tbl.kegg[,19:20]-x)),
        do.call(cbind, lapply(tbl2.kegg[,22:29],
                              function(x) tbl.kegg[,21:22]-x)),
        do.call(cbind, lapply(tbl2.kegg[,24:29],
                              function(x) tbl.kegg[,23:24]-x)),
        do.call(cbind, lapply(tbl2.kegg[,26:29],
                              function(x) tbl.kegg[,25:26]-x)),
        do.call(cbind, lapply(tbl2.kegg[,28:29],
                              function(x) tbl.kegg[,27:28]-x)))


# putting all variables in one column
# not counting with the columns comparing the same value
kegg.hist <- melt(kegg.dif, id.vars = c("estimate", "species"),
                  measure.vars = c(names(kegg.dif[3:ncol(kegg.dif)])))

# puting NA values for when the difference is 0 (i.e. when
# comparing the same variables)
split <- str_split_fixed(kegg.hist$variable, "\\.", 2)
```

```r
kegg.hist$var1 <- split[,1]
kegg.hist$var2 <- split[,2]

# removing the variables being compared to itself
kegg.hist$value[kegg.hist$var1 == kegg.hist$var2] <- NA
kegg.hist <- na.omit(kegg.hist[1:6])

## getting the p-value for each difference
## Arabidopsis
arab.hist <- subset(kegg.hist, kegg.hist$species == "Arabidopsis")
nboots <- 100
arab.smallkegg.p <- list()
for (i in 1:nboots) {
  arab.smallkegg.p[[i]] <- ddply(arab.hist, c("species", "estimate",
                                              "var1", "var2"),
                                 function(x, N=28){
    c <- as.numeric(nrow(x[x$value < 0,]))
    c2 <- as.numeric(nrow(x[x$value > 0,]))
    m <- min(c, c2)
    p <- (2*m+1)/(N+1)
    tbl <- data.frame(m, p)
  })
}

# correcting the p-value for multiple testing
arab.kegg.p.adj <- lapply(arab.smallkegg.p, function(x) {
  ddply(x,  c("species","estimate", "var1", "var2"), function(x) {
    p.value <- p.adjust(x$p)
    data.frame(p.value)
  })
})

# taking the minimum p-value of the replicates performed
tbl.arab.p.adj <- rbindlist(arab.kegg.p.adj)
arab.pvalue <- ddply(tbl.arab.p.adj, c("species","estimate", "var1", "var2"),
                     function(x) {
  p.value <- min(x$p.value)
  data.frame(p.value)
})
# showing the table
kable(arab.pvalue, format = "latex", booktabs = TRUE, longtable = TRUE) %>%
  kable_styling(latex_options = c("hold_position", "repeat_header"),
                font_size = 7)
```

species	estimate	var1	var2	p.value
Arabidopsis	dnds	Chaperones_and_folding_catalysts	Amino_acid_related_enzymes	0.1034483
Arabidopsis	dnds	Chromosome_and_associated_proteins	Amino_acid_related_enzymes	0.0344828
Arabidopsis	dnds	Chromosome_and_associated_proteins	Chaperones_and_folding_catalysts	0.0344828
Arabidopsis	dnds	Cytochrome_P450	Amino_acid_related_enzymes	0.0344828
Arabidopsis	dnds	Cytochrome_P450	Chaperones_and_folding_catalysts	0.2413793
Arabidopsis	dnds	Cytochrome_P450	Chromosome_and_associated_proteins	0.0344828
Arabidopsis	dnds	Cytoskeleton_proteins	Amino_acid_related_enzymes	0.0344828
Arabidopsis	dnds	Cytoskeleton_proteins	Chaperones_and_folding_catalysts	0.0344828
Arabidopsis	dnds	Cytoskeleton_proteins	Chromosome_and_associated_proteins	0.0344828
Arabidopsis	dnds	Cytoskeleton_proteins	Cytochrome_P450	0.5172414

(continued)

species	estimate	var1	var2	p.value
Arabidopsis	dnds	DNA__repair__and__recombination__proteins	Amino__acid__related__enzymes	0.0344828
Arabidopsis	dnds	DNA__repair__and__recombination__proteins	Chaperones__and__folding__catalysts	0.0344828
Arabidopsis	dnds	DNA__repair__and__recombination__proteins	Chromosome__and__associated__proteins	0.9310345
Arabidopsis	dnds	DNA__repair__and__recombination__proteins	Cytochrome__P450	0.0344828
Arabidopsis	dnds	DNA__repair__and__recombination__proteins	Cytoskeleton__proteins	0.0344828
Arabidopsis	dnds	DNA__replication__proteins	Amino__acid__related__enzymes	0.0344828
Arabidopsis	dnds	DNA__replication__proteins	Chaperones__and__folding__catalysts	0.0344828
Arabidopsis	dnds	DNA__replication__proteins	Chromosome__and__associated__proteins	0.0344828
Arabidopsis	dnds	DNA__replication__proteins	Cytochrome__P450	0.3103448
Arabidopsis	dnds	DNA__replication__proteins	Cytoskeleton__proteins	0.3103448
Arabidopsis	dnds	DNA__replication__proteins	DNA__repair__and__recombination__proteins	0.1034483
Arabidopsis	dnds	Exosome	Amino__acid__related__enzymes	0.1724138
Arabidopsis	dnds	Exosome	Chaperones__and__folding__catalysts	0.0344828
Arabidopsis	dnds	Exosome	Chromosome__and__associated__proteins	0.0344828
Arabidopsis	dnds	Exosome	Cytochrome__P450	0.0344828
Arabidopsis	dnds	Exosome	Cytoskeleton__proteins	0.0344828
Arabidopsis	dnds	Exosome	DNA__repair__and__recombination__proteins	0.0344828
Arabidopsis	dnds	Exosome	DNA__replication__proteins	0.0344828
Arabidopsis	dnds	G__protein__coupled__receptors	Amino__acid__related__enzymes	0.3103448
Arabidopsis	dnds	G__protein__coupled__receptors	Chaperones__and__folding__catalysts	0.3793103
Arabidopsis	dnds	G__protein__coupled__receptors	Chromosome__and__associated__proteins	0.0344828
Arabidopsis	dnds	G__protein__coupled__receptors	Cytochrome__P450	0.1034483
Arabidopsis	dnds	G__protein__coupled__receptors	Cytoskeleton__proteins	0.0344828
Arabidopsis	dnds	G__protein__coupled__receptors	DNA__repair__and__recombination__proteins	0.0344828
Arabidopsis	dnds	G__protein__coupled__receptors	DNA__replication__proteins	0.0344828
Arabidopsis	dnds	G__protein__coupled__receptors	Exosome	0.0344828
Arabidopsis	dnds	Glycosyltransferases	Amino__acid__related__enzymes	0.1034483
Arabidopsis	dnds	Glycosyltransferases	Chaperones__and__folding__catalysts	0.7931034
Arabidopsis	dnds	Glycosyltransferases	Chromosome__and__associated__proteins	0.0344828
Arabidopsis	dnds	Glycosyltransferases	Cytochrome__P450	0.0344828
Arabidopsis	dnds	Glycosyltransferases	Cytoskeleton__proteins	0.0344828
Arabidopsis	dnds	Glycosyltransferases	DNA__repair__and__recombination__proteins	0.0344828
Arabidopsis	dnds	Glycosyltransferases	DNA__replication__proteins	0.0344828
Arabidopsis	dnds	Glycosyltransferases	Exosome	0.0344828
Arabidopsis	dnds	Glycosyltransferases	G__protein__coupled__receptors	0.3793103
Arabidopsis	dnds	Ion__channels	Amino__acid__related__enzymes	0.0344828
Arabidopsis	dnds	Ion__channels	Chaperones__and__folding__catalysts	0.0344828
Arabidopsis	dnds	Ion__channels	Chromosome__and__associated__proteins	0.0344828
Arabidopsis	dnds	Ion__channels	Cytochrome__P450	0.0344828
Arabidopsis	dnds	Ion__channels	Cytoskeleton__proteins	0.0344828
Arabidopsis	dnds	Ion__channels	DNA__repair__and__recombination__proteins	0.0344828
Arabidopsis	dnds	Ion__channels	DNA__replication__proteins	0.0344828
Arabidopsis	dnds	Ion__channels	Exosome	0.0344828
Arabidopsis	dnds	Ion__channels	G__protein__coupled__receptors	0.0344828
Arabidopsis	dnds	Ion__channels	Glycosyltransferases	0.0344828
Arabidopsis	dnds	Lipid__biosynthesis__proteins	Amino__acid__related__enzymes	0.0344828
Arabidopsis	dnds	Lipid__biosynthesis__proteins	Chaperones__and__folding__catalysts	0.8620690
Arabidopsis	dnds	Lipid__biosynthesis__proteins	Chromosome__and__associated__proteins	0.0344828
Arabidopsis	dnds	Lipid__biosynthesis__proteins	Cytochrome__P450	0.4482759
Arabidopsis	dnds	Lipid__biosynthesis__proteins	Cytoskeleton__proteins	0.3793103
Arabidopsis	dnds	Lipid__biosynthesis__proteins	DNA__repair__and__recombination__proteins	0.0344828
Arabidopsis	dnds	Lipid__biosynthesis__proteins	DNA__replication__proteins	0.1034483
Arabidopsis	dnds	Lipid__biosynthesis__proteins	Exosome	0.0344828
Arabidopsis	dnds	Lipid__biosynthesis__proteins	G__protein__coupled__receptors	0.3793103
Arabidopsis	dnds	Lipid__biosynthesis__proteins	Glycosyltransferases	0.6551724
Arabidopsis	dnds	Lipid__biosynthesis__proteins	Ion__channels	0.0344828
Arabidopsis	dnds	Membrane__trafficking	Amino__acid__related__enzymes	0.1034483
Arabidopsis	dnds	Membrane__trafficking	Chaperones__and__folding__catalysts	1.0000000
Arabidopsis	dnds	Membrane__trafficking	Chromosome__and__associated__proteins	0.0344828
Arabidopsis	dnds	Membrane__trafficking	Cytochrome__P450	0.1034483
Arabidopsis	dnds	Membrane__trafficking	Cytoskeleton__proteins	0.0344828

(continued)

species	estimate	var1	var2	p.value
Arabidopsis	dnds	Membrane__trafficking	DNA__repair__and__recombination__proteins	0.0344828
Arabidopsis	dnds	Membrane__trafficking	DNA__replication__proteins	0.0344828
Arabidopsis	dnds	Membrane__trafficking	Exosome	0.0344828
Arabidopsis	dnds	Membrane__trafficking	G__protein__coupled__receptors	0.3103448
Arabidopsis	dnds	Membrane__trafficking	Glycosyltransferases	0.5172414
Arabidopsis	dnds	Membrane__trafficking	Ion__channels	0.0344828
Arabidopsis	dnds	Membrane__trafficking	Lipid__biosynthesis__proteins	1.0000000
Arabidopsis	dnds	Messenger__RNA__biogenesis	Amino__acid__related__enzymes	0.0344828
Arabidopsis	dnds	Messenger__RNA__biogenesis	Chaperones__and__folding__catalysts	0.0344828
Arabidopsis	dnds	Messenger__RNA__biogenesis	Chromosome__and__associated__proteins	0.0344828
Arabidopsis	dnds	Messenger__RNA__biogenesis	Cytochrome__P450	0.3793103
Arabidopsis	dnds	Messenger__RNA__biogenesis	Cytoskeleton__proteins	0.6551724
Arabidopsis	dnds	Messenger__RNA__biogenesis	DNA__repair__and__recombination__proteins	0.0344828
Arabidopsis	dnds	Messenger__RNA__biogenesis	DNA__replication__proteins	0.4482759
Arabidopsis	dnds	Messenger__RNA__biogenesis	Exosome	0.0344828
Arabidopsis	dnds	Messenger__RNA__biogenesis	G__protein__coupled__receptors	0.0344828
Arabidopsis	dnds	Messenger__RNA__biogenesis	Glycosyltransferases	0.0344828
Arabidopsis	dnds	Messenger__RNA__biogenesis	Ion__channels	0.0344828
Arabidopsis	dnds	Messenger__RNA__biogenesis	Lipid__biosynthesis__proteins	0.1724138
Arabidopsis	dnds	Messenger__RNA__biogenesis	Membrane__trafficking	0.0344828
Arabidopsis	dnds	Mitochondrial__biogenesis	Amino__acid__related__enzymes	0.0344828
Arabidopsis	dnds	Mitochondrial__biogenesis	Chaperones__and__folding__catalysts	0.3793103
Arabidopsis	dnds	Mitochondrial__biogenesis	Chromosome__and__associated__proteins	0.1034483
Arabidopsis	dnds	Mitochondrial__biogenesis	Cytochrome__P450	0.8620690
Arabidopsis	dnds	Mitochondrial__biogenesis	Cytoskeleton__proteins	0.7931034
Arabidopsis	dnds	Mitochondrial__biogenesis	DNA__repair__and__recombination__proteins	0.1034483
Arabidopsis	dnds	Mitochondrial__biogenesis	DNA__replication__proteins	0.5862069
Arabidopsis	dnds	Mitochondrial__biogenesis	Exosome	0.0344828
Arabidopsis	dnds	Mitochondrial__biogenesis	G__protein__coupled__receptors	0.1034483
Arabidopsis	dnds	Mitochondrial__biogenesis	Glycosyltransferases	0.2413793
Arabidopsis	dnds	Mitochondrial__biogenesis	Ion__channels	0.0344828
Arabidopsis	dnds	Mitochondrial__biogenesis	Lipid__biosynthesis__proteins	0.3793103
Arabidopsis	dnds	Mitochondrial__biogenesis	Membrane__trafficking	0.2413793
Arabidopsis	dnds	Mitochondrial__biogenesis	Messenger__RNA__biogenesis	0.7241379
Arabidopsis	dnds	Peptidases	Amino__acid__related__enzymes	0.0344828
Arabidopsis	dnds	Peptidases	Chaperones__and__folding__catalysts	0.1724138
Arabidopsis	dnds	Peptidases	Chromosome__and__associated__proteins	0.0344828
Arabidopsis	dnds	Peptidases	Cytochrome__P450	0.6551724
Arabidopsis	dnds	Peptidases	Cytoskeleton__proteins	0.4482759
Arabidopsis	dnds	Peptidases	DNA__repair__and__recombination__proteins	0.0344828
Arabidopsis	dnds	Peptidases	DNA__replication__proteins	0.0344828
Arabidopsis	dnds	Peptidases	Exosome	0.0344828
Arabidopsis	dnds	Peptidases	G__protein__coupled__receptors	0.1034483
Arabidopsis	dnds	Peptidases	Glycosyltransferases	0.1034483
Arabidopsis	dnds	Peptidases	Ion__channels	0.0344828
Arabidopsis	dnds	Peptidases	Lipid__biosynthesis__proteins	0.5172414
Arabidopsis	dnds	Peptidases	Membrane__trafficking	0.1724138
Arabidopsis	dnds	Peptidases	Messenger__RNA__biogenesis	0.1724138
Arabidopsis	dnds	Peptidases	Mitochondrial__biogenesis	0.8620690
Arabidopsis	dnds	Photosynthesis__proteins	Amino__acid__related__enzymes	0.2413793
Arabidopsis	dnds	Photosynthesis__proteins	Chaperones__and__folding__catalysts	0.0344828
Arabidopsis	dnds	Photosynthesis__proteins	Chromosome__and__associated__proteins	0.0344828
Arabidopsis	dnds	Photosynthesis__proteins	Cytochrome__P450	0.0344828
Arabidopsis	dnds	Photosynthesis__proteins	Cytoskeleton__proteins	0.0344828
Arabidopsis	dnds	Photosynthesis__proteins	DNA__repair__and__recombination__proteins	0.0344828
Arabidopsis	dnds	Photosynthesis__proteins	DNA__replication__proteins	0.0344828
Arabidopsis	dnds	Photosynthesis__proteins	Exosome	0.9310345
Arabidopsis	dnds	Photosynthesis__proteins	G__protein__coupled__receptors	0.0344828
Arabidopsis	dnds	Photosynthesis__proteins	Glycosyltransferases	0.0344828
Arabidopsis	dnds	Photosynthesis__proteins	Ion__channels	0.0344828
Arabidopsis	dnds	Photosynthesis__proteins	Lipid__biosynthesis__proteins	0.0344828
Arabidopsis	dnds	Photosynthesis__proteins	Membrane__trafficking	0.0344828

(continued)

species	estimate	var1	var2	p.value
Arabidopsis	dnds	Photosynthesis__proteins	Messenger__RNA__biogenesis	0.0344828
Arabidopsis	dnds	Photosynthesis__proteins	Mitochondrial__biogenesis	0.0344828
Arabidopsis	dnds	Photosynthesis__proteins	Peptidases	0.0344828
Arabidopsis	dnds	Proteasome	Amino__acid__related__enzymes	0.3793103
Arabidopsis	dnds	Proteasome	Chaperones__and__folding__catalysts	0.0344828
Arabidopsis	dnds	Proteasome	Chromosome__and__associated__proteins	0.0344828
Arabidopsis	dnds	Proteasome	Cytochrome__P450	0.0344828
Arabidopsis	dnds	Proteasome	Cytoskeleton__proteins	0.0344828
Arabidopsis	dnds	Proteasome	DNA__repair__and__recombination__proteins	0.0344828
Arabidopsis	dnds	Proteasome	DNA__replication__proteins	0.0344828
Arabidopsis	dnds	Proteasome	Exosome	0.7931034
Arabidopsis	dnds	Proteasome	G__protein__coupled__receptors	0.0344828
Arabidopsis	dnds	Proteasome	Glycosyltransferases	0.0344828
Arabidopsis	dnds	Proteasome	Ion__channels	0.0344828
Arabidopsis	dnds	Proteasome	Lipid__biosynthesis__proteins	0.0344828
Arabidopsis	dnds	Proteasome	Membrane__trafficking	0.0344828
Arabidopsis	dnds	Proteasome	Messenger__RNA__biogenesis	0.0344828
Arabidopsis	dnds	Proteasome	Mitochondrial__biogenesis	0.0344828
Arabidopsis	dnds	Proteasome	Peptidases	0.0344828
Arabidopsis	dnds	Proteasome	Photosynthesis__proteins	0.7931034
Arabidopsis	dnds	Protein__kinases	Amino__acid__related__enzymes	0.3793103
Arabidopsis	dnds	Protein__kinases	Chaperones__and__folding__catalysts	0.3103448
Arabidopsis	dnds	Protein__kinases	Chromosome__and__associated__proteins	0.0344828
Arabidopsis	dnds	Protein__kinases	Cytochrome__P450	0.0344828
Arabidopsis	dnds	Protein__kinases	Cytoskeleton__proteins	0.0344828
Arabidopsis	dnds	Protein__kinases	DNA__repair__and__recombination__proteins	0.0344828
Arabidopsis	dnds	Protein__kinases	DNA__replication__proteins	0.0344828
Arabidopsis	dnds	Protein__kinases	Exosome	0.0344828
Arabidopsis	dnds	Protein__kinases	G__protein__coupled__receptors	0.9310345
Arabidopsis	dnds	Protein__kinases	Glycosyltransferases	0.3103448
Arabidopsis	dnds	Protein__kinases	Ion__channels	0.0344828
Arabidopsis	dnds	Protein__kinases	Lipid__biosynthesis__proteins	0.2413793
Arabidopsis	dnds	Protein__kinases	Membrane__trafficking	0.2413793
Arabidopsis	dnds	Protein__kinases	Messenger__RNA__biogenesis	0.0344828
Arabidopsis	dnds	Protein__kinases	Mitochondrial__biogenesis	0.0344828
Arabidopsis	dnds	Protein__kinases	Peptidases	0.0344828
Arabidopsis	dnds	Protein__kinases	Photosynthesis__proteins	0.1034483
Arabidopsis	dnds	Protein__kinases	Proteasome	0.1034483
Arabidopsis	dnds	Protein__phosphatases__and__associated__proteins	Amino__acid__related__enzymes	0.0344828
Arabidopsis	dnds	Protein__phosphatases__and__associated__proteins	Chaperones__and__folding__catalysts	0.2413793
Arabidopsis	dnds	Protein__phosphatases__and__associated__proteins	Chromosome__and__associated__proteins	0.0344828
Arabidopsis	dnds	Protein__phosphatases__and__associated__proteins	Cytochrome__P450	0.8620690
Arabidopsis	dnds	Protein__phosphatases__and__associated__proteins	Cytoskeleton__proteins	0.6551724
Arabidopsis	dnds	Protein__phosphatases__and__associated__proteins	DNA__repair__and__recombination__proteins	0.0344828
Arabidopsis	dnds	Protein__phosphatases__and__associated__proteins	DNA__replication__proteins	0.3103448
Arabidopsis	dnds	Protein__phosphatases__and__associated__proteins	Exosome	0.0344828
Arabidopsis	dnds	Protein__phosphatases__and__associated__proteins	G__protein__coupled__receptors	0.0344828
Arabidopsis	dnds	Protein__phosphatases__and__associated__proteins	Glycosyltransferases	0.2413793
Arabidopsis	dnds	Protein__phosphatases__and__associated__proteins	Ion__channels	0.0344828
Arabidopsis	dnds	Protein__phosphatases__and__associated__proteins	Lipid__biosynthesis__proteins	0.4482759
Arabidopsis	dnds	Protein__phosphatases__and__associated__proteins	Membrane__trafficking	0.1724138
Arabidopsis	dnds	Protein__phosphatases__and__associated__proteins	Messenger__RNA__biogenesis	0.5862069
Arabidopsis	dnds	Protein__phosphatases__and__associated__proteins	Mitochondrial__biogenesis	0.7931034
Arabidopsis	dnds	Protein__phosphatases__and__associated__proteins	Peptidases	0.8620690
Arabidopsis	dnds	Protein__phosphatases__and__associated__proteins	Photosynthesis__proteins	0.0344828
Arabidopsis	dnds	Protein__phosphatases__and__associated__proteins	Proteasome	0.0344828
Arabidopsis	dnds	Protein__phosphatases__and__associated__proteins	Protein__kinases	0.0344828
Arabidopsis	dnds	Ribosome	Amino__acid__related__enzymes	0.3103448
Arabidopsis	dnds	Ribosome	Chaperones__and__folding__catalysts	0.0344828
Arabidopsis	dnds	Ribosome	Chromosome__and__associated__proteins	0.0344828
Arabidopsis	dnds	Ribosome	Cytochrome__P450	0.0344828
Arabidopsis	dnds	Ribosome	Cytoskeleton__proteins	0.0344828

(continued)

species	estimate	var1	var2	p.value
Arabidopsis	dnds	Ribosome	DNA__repair__and__recombination__proteins	0.0344828
Arabidopsis	dnds	Ribosome	DNA__replication__proteins	0.0344828
Arabidopsis	dnds	Ribosome	Exosome	0.9310345
Arabidopsis	dnds	Ribosome	G__protein__coupled__receptors	0.0344828
Arabidopsis	dnds	Ribosome	Glycosyltransferases	0.0344828
Arabidopsis	dnds	Ribosome	Ion__channels	0.0344828
Arabidopsis	dnds	Ribosome	Lipid__biosynthesis__proteins	0.0344828
Arabidopsis	dnds	Ribosome	Membrane__trafficking	0.0344828
Arabidopsis	dnds	Ribosome	Messenger__RNA__biogenesis	0.0344828
Arabidopsis	dnds	Ribosome	Mitochondrial__biogenesis	0.0344828
Arabidopsis	dnds	Ribosome	Peptidases	0.0344828
Arabidopsis	dnds	Ribosome	Photosynthesis__proteins	0.6551724
Arabidopsis	dnds	Ribosome	Proteasome	0.9310345
Arabidopsis	dnds	Ribosome	Protein__kinases	0.0344828
Arabidopsis	dnds	Ribosome	Protein__phosphatases__and__associated__proteins	0.0344828
Arabidopsis	dnds	Ribosome__biogenesis	Amino__acid__related__enzymes	0.0344828
Arabidopsis	dnds	Ribosome__biogenesis	Chaperones__and__folding__catalysts	0.0344828
Arabidopsis	dnds	Ribosome__biogenesis	Chromosome__and__associated__proteins	0.6551724
Arabidopsis	dnds	Ribosome__biogenesis	Cytochrome__P450	0.0344828
Arabidopsis	dnds	Ribosome__biogenesis	Cytoskeleton__proteins	0.0344828
Arabidopsis	dnds	Ribosome__biogenesis	DNA__repair__and__recombination__proteins	0.6551724
Arabidopsis	dnds	Ribosome__biogenesis	DNA__replication__proteins	0.1034483
Arabidopsis	dnds	Ribosome__biogenesis	Exosome	0.0344828
Arabidopsis	dnds	Ribosome__biogenesis	G__protein__coupled__receptors	0.0344828
Arabidopsis	dnds	Ribosome__biogenesis	Glycosyltransferases	0.0344828
Arabidopsis	dnds	Ribosome__biogenesis	Ion__channels	0.0344828
Arabidopsis	dnds	Ribosome__biogenesis	Lipid__biosynthesis__proteins	0.0344828
Arabidopsis	dnds	Ribosome__biogenesis	Membrane__trafficking	0.0344828
Arabidopsis	dnds	Ribosome__biogenesis	Messenger__RNA__biogenesis	0.0344828
Arabidopsis	dnds	Ribosome__biogenesis	Mitochondrial__biogenesis	0.2413793
Arabidopsis	dnds	Ribosome__biogenesis	Peptidases	0.0344828
Arabidopsis	dnds	Ribosome__biogenesis	Photosynthesis__proteins	0.0344828
Arabidopsis	dnds	Ribosome__biogenesis	Proteasome	0.0344828
Arabidopsis	dnds	Ribosome__biogenesis	Protein__kinases	0.0344828
Arabidopsis	dnds	Ribosome__biogenesis	Protein__phosphatases__and__associated__proteins	0.0344828
Arabidopsis	dnds	Ribosome__biogenesis	Ribosome	0.0344828
Arabidopsis	dnds	Spliceosome	Amino__acid__related__enzymes	0.0344828
Arabidopsis	dnds	Spliceosome	Chaperones__and__folding__catalysts	0.1034483
Arabidopsis	dnds	Spliceosome	Chromosome__and__associated__proteins	0.0344828
Arabidopsis	dnds	Spliceosome	Cytochrome__P450	0.7241379
Arabidopsis	dnds	Spliceosome	Cytoskeleton__proteins	0.9310345
Arabidopsis	dnds	Spliceosome	DNA__repair__and__recombination__proteins	0.0344828
Arabidopsis	dnds	Spliceosome	DNA__replication__proteins	0.2413793
Arabidopsis	dnds	Spliceosome	Exosome	0.0344828
Arabidopsis	dnds	Spliceosome	G__protein__coupled__receptors	0.0344828
Arabidopsis	dnds	Spliceosome	Glycosyltransferases	0.0344828
Arabidopsis	dnds	Spliceosome	Ion__channels	0.0344828
Arabidopsis	dnds	Spliceosome	Lipid__biosynthesis__proteins	0.2413793
Arabidopsis	dnds	Spliceosome	Membrane__trafficking	0.0344828
Arabidopsis	dnds	Spliceosome	Messenger__RNA__biogenesis	0.5862069
Arabidopsis	dnds	Spliceosome	Mitochondrial__biogenesis	0.9310345
Arabidopsis	dnds	Spliceosome	Peptidases	0.3793103
Arabidopsis	dnds	Spliceosome	Photosynthesis__proteins	0.0344828
Arabidopsis	dnds	Spliceosome	Proteasome	0.0344828
Arabidopsis	dnds	Spliceosome	Protein__kinases	0.0344828
Arabidopsis	dnds	Spliceosome	Protein__phosphatases__and__associated__proteins	0.8620690
Arabidopsis	dnds	Spliceosome	Ribosome	0.0344828
Arabidopsis	dnds	Spliceosome	Ribosome__biogenesis	0.0344828
Arabidopsis	dnds	Transcription__factors	Amino__acid__related__enzymes	0.0344828
Arabidopsis	dnds	Transcription__factors	Chaperones__and__folding__catalysts	0.0344828
Arabidopsis	dnds	Transcription__factors	Chromosome__and__associated__proteins	0.7241379

(continued)

species	estimate	var1	var2	p.value
Arabidopsis	dnds	Transcription__factors	Cytochrome__P450	0.0344828
Arabidopsis	dnds	Transcription__factors	Cytoskeleton__proteins	0.0344828
Arabidopsis	dnds	Transcription__factors	DNA__repair__and__recombination__proteins	0.3793103
Arabidopsis	dnds	Transcription__factors	DNA__replication__proteins	0.1034483
Arabidopsis	dnds	Transcription__factors	Exosome	0.0344828
Arabidopsis	dnds	Transcription__factors	G__protein__coupled__receptors	0.0344828
Arabidopsis	dnds	Transcription__factors	Glycosyltransferases	0.0344828
Arabidopsis	dnds	Transcription__factors	Ion__channels	0.0344828
Arabidopsis	dnds	Transcription__factors	Lipid__biosynthesis__proteins	0.0344828
Arabidopsis	dnds	Transcription__factors	Membrane__trafficking	0.0344828
Arabidopsis	dnds	Transcription__factors	Messenger__RNA__biogenesis	0.0344828
Arabidopsis	dnds	Transcription__factors	Mitochondrial__biogenesis	0.1034483
Arabidopsis	dnds	Transcription__factors	Peptidases	0.0344828
Arabidopsis	dnds	Transcription__factors	Photosynthesis__proteins	0.0344828
Arabidopsis	dnds	Transcription__factors	Proteasome	0.0344828
Arabidopsis	dnds	Transcription__factors	Protein__kinases	0.0344828
Arabidopsis	dnds	Transcription__factors	Protein__phosphatases__and__associated__proteins	0.0344828
Arabidopsis	dnds	Transcription__factors	Ribosome	0.0344828
Arabidopsis	dnds	Transcription__factors	Ribosome__biogenesis	0.8620690
Arabidopsis	dnds	Transcription__factors	Spliceosome	0.0344828
Arabidopsis	dnds	Transcription__machinery	Amino__acid__related__enzymes	0.0344828
Arabidopsis	dnds	Transcription__machinery	Chaperones__and__folding__catalysts	0.0344828
Arabidopsis	dnds	Transcription__machinery	Chromosome__and__associated__proteins	0.3103448
Arabidopsis	dnds	Transcription__machinery	Cytochrome__P450	0.0344828
Arabidopsis	dnds	Transcription__machinery	Cytoskeleton__proteins	0.0344828
Arabidopsis	dnds	Transcription__machinery	DNA__repair__and__recombination__proteins	0.5862069
Arabidopsis	dnds	Transcription__machinery	DNA__replication__proteins	0.0344828
Arabidopsis	dnds	Transcription__machinery	Exosome	0.0344828
Arabidopsis	dnds	Transcription__machinery	G__protein__coupled__receptors	0.0344828
Arabidopsis	dnds	Transcription__machinery	Glycosyltransferases	0.0344828
Arabidopsis	dnds	Transcription__machinery	Ion__channels	0.0344828
Arabidopsis	dnds	Transcription__machinery	Lipid__biosynthesis__proteins	0.0344828
Arabidopsis	dnds	Transcription__machinery	Membrane__trafficking	0.0344828
Arabidopsis	dnds	Transcription__machinery	Messenger__RNA__biogenesis	0.0344828
Arabidopsis	dnds	Transcription__machinery	Mitochondrial__biogenesis	0.1034483
Arabidopsis	dnds	Transcription__machinery	Peptidases	0.0344828
Arabidopsis	dnds	Transcription__machinery	Photosynthesis__proteins	0.0344828
Arabidopsis	dnds	Transcription__machinery	Proteasome	0.0344828
Arabidopsis	dnds	Transcription__machinery	Protein__kinases	0.0344828
Arabidopsis	dnds	Transcription__machinery	Protein__phosphatases__and__associated__proteins	0.0344828
Arabidopsis	dnds	Transcription__machinery	Ribosome	0.0344828
Arabidopsis	dnds	Transcription__machinery	Ribosome__biogenesis	0.9310345
Arabidopsis	dnds	Transcription__machinery	Spliceosome	0.0344828
Arabidopsis	dnds	Transcription__machinery	Transcription__factors	0.8620690
Arabidopsis	dnds	Transfer__RNA__biogenesis	Amino__acid__related__enzymes	0.0344828
Arabidopsis	dnds	Transfer__RNA__biogenesis	Chaperones__and__folding__catalysts	0.0344828
Arabidopsis	dnds	Transfer__RNA__biogenesis	Chromosome__and__associated__proteins	0.0344828
Arabidopsis	dnds	Transfer__RNA__biogenesis	Cytochrome__P450	0.3103448
Arabidopsis	dnds	Transfer__RNA__biogenesis	Cytoskeleton__proteins	0.5172414
Arabidopsis	dnds	Transfer__RNA__biogenesis	DNA__repair__and__recombination__proteins	0.0344828
Arabidopsis	dnds	Transfer__RNA__biogenesis	DNA__replication__proteins	0.7931034
Arabidopsis	dnds	Transfer__RNA__biogenesis	Exosome	0.0344828
Arabidopsis	dnds	Transfer__RNA__biogenesis	G__protein__coupled__receptors	0.0344828
Arabidopsis	dnds	Transfer__RNA__biogenesis	Glycosyltransferases	0.0344828
Arabidopsis	dnds	Transfer__RNA__biogenesis	Ion__channels	0.0344828
Arabidopsis	dnds	Transfer__RNA__biogenesis	Lipid__biosynthesis__proteins	0.0344828
Arabidopsis	dnds	Transfer__RNA__biogenesis	Membrane__trafficking	0.0344828
Arabidopsis	dnds	Transfer__RNA__biogenesis	Messenger__RNA__biogenesis	0.6551724
Arabidopsis	dnds	Transfer__RNA__biogenesis	Mitochondrial__biogenesis	0.5862069
Arabidopsis	dnds	Transfer__RNA__biogenesis	Peptidases	0.1034483
Arabidopsis	dnds	Transfer__RNA__biogenesis	Photosynthesis__proteins	0.0344828
Arabidopsis	dnds	Transfer__RNA__biogenesis	Proteasome	0.0344828

(continued)

species	estimate	var1	var2	p.value
Arabidopsis	dnds	Transfer__RNA__biogenesis	Protein__kinases	0.0344828
Arabidopsis	dnds	Transfer__RNA__biogenesis	Protein__phosphatases__and__associated__proteins	0.3103448
Arabidopsis	dnds	Transfer__RNA__biogenesis	Ribosome	0.0344828
Arabidopsis	dnds	Transfer__RNA__biogenesis	Ribosome__biogenesis	0.0344828
Arabidopsis	dnds	Transfer__RNA__biogenesis	Spliceosome	0.3103448
Arabidopsis	dnds	Transfer__RNA__biogenesis	Transcription__factors	0.0344828
Arabidopsis	dnds	Transfer__RNA__biogenesis	Transcription__machinery	0.0344828
Arabidopsis	dnds	Translation__factors	Amino__acid__related__enzymes	0.0344828
Arabidopsis	dnds	Translation__factors	Chaperones__and__folding__catalysts	0.3103448
Arabidopsis	dnds	Translation__factors	Chromosome__and__associated__proteins	0.0344828
Arabidopsis	dnds	Translation__factors	Cytochrome__P450	0.7241379
Arabidopsis	dnds	Translation__factors	Cytoskeleton__proteins	0.7241379
Arabidopsis	dnds	Translation__factors	DNA__repair__and__recombination__proteins	0.0344828
Arabidopsis	dnds	Translation__factors	DNA__replication__proteins	0.3103448
Arabidopsis	dnds	Translation__factors	Exosome	0.0344828
Arabidopsis	dnds	Translation__factors	G__protein__coupled__receptors	0.1034483
Arabidopsis	dnds	Translation__factors	Glycosyltransferases	0.0344828
Arabidopsis	dnds	Translation__factors	Ion__channels	0.0344828
Arabidopsis	dnds	Translation__factors	Lipid__biosynthesis__proteins	0.3793103
Arabidopsis	dnds	Translation__factors	Membrane__trafficking	0.1724138
Arabidopsis	dnds	Translation__factors	Messenger__RNA__biogenesis	0.3103448
Arabidopsis	dnds	Translation__factors	Mitochondrial__biogenesis	0.7931034
Arabidopsis	dnds	Translation__factors	Peptidases	0.7931034
Arabidopsis	dnds	Translation__factors	Photosynthesis__proteins	0.0344828
Arabidopsis	dnds	Translation__factors	Proteasome	0.0344828
Arabidopsis	dnds	Translation__factors	Protein__kinases	0.0344828
Arabidopsis	dnds	Translation__factors	Protein__phosphatases__and__associated__proteins	0.9310345
Arabidopsis	dnds	Translation__factors	Ribosome	0.0344828
Arabidopsis	dnds	Translation__factors	Ribosome__biogenesis	0.0344828
Arabidopsis	dnds	Translation__factors	Spliceosome	0.7241379
Arabidopsis	dnds	Translation__factors	Transcription__factors	0.0344828
Arabidopsis	dnds	Translation__factors	Transcription__machinery	0.0344828
Arabidopsis	dnds	Translation__factors	Transfer__RNA__biogenesis	0.3103448
Arabidopsis	dnds	Transporters	Amino__acid__related__enzymes	0.2413793
Arabidopsis	dnds	Transporters	Chaperones__and__folding__catalysts	0.1034483
Arabidopsis	dnds	Transporters	Chromosome__and__associated__proteins	0.0344828
Arabidopsis	dnds	Transporters	Cytochrome__P450	0.0344828
Arabidopsis	dnds	Transporters	Cytoskeleton__proteins	0.0344828
Arabidopsis	dnds	Transporters	DNA__repair__and__recombination__proteins	0.0344828
Arabidopsis	dnds	Transporters	DNA__replication__proteins	0.0344828
Arabidopsis	dnds	Transporters	Exosome	0.0344828
Arabidopsis	dnds	Transporters	G__protein__coupled__receptors	0.6551724
Arabidopsis	dnds	Transporters	Glycosyltransferases	0.0344828
Arabidopsis	dnds	Transporters	Ion__channels	0.0344828
Arabidopsis	dnds	Transporters	Lipid__biosynthesis__proteins	0.1724138
Arabidopsis	dnds	Transporters	Membrane__trafficking	0.0344828
Arabidopsis	dnds	Transporters	Messenger__RNA__biogenesis	0.0344828
Arabidopsis	dnds	Transporters	Mitochondrial__biogenesis	0.0344828
Arabidopsis	dnds	Transporters	Peptidases	0.0344828
Arabidopsis	dnds	Transporters	Photosynthesis__proteins	0.0344828
Arabidopsis	dnds	Transporters	Proteasome	0.1034483
Arabidopsis	dnds	Transporters	Protein__kinases	0.7931034
Arabidopsis	dnds	Transporters	Protein__phosphatases__and__associated__proteins	0.0344828
Arabidopsis	dnds	Transporters	Ribosome	0.0344828
Arabidopsis	dnds	Transporters	Ribosome__biogenesis	0.0344828
Arabidopsis	dnds	Transporters	Spliceosome	0.0344828
Arabidopsis	dnds	Transporters	Transcription__factors	0.0344828
Arabidopsis	dnds	Transporters	Transcription__machinery	0.0344828
Arabidopsis	dnds	Transporters	Transfer__RNA__biogenesis	0.0344828
Arabidopsis	dnds	Transporters	Translation__factors	0.0344828
Arabidopsis	dnds	Ubiquitin__system	Amino__acid__related__enzymes	0.0344828
Arabidopsis	dnds	Ubiquitin__system	Chaperones__and__folding__catalysts	0.0344828

(continued)

species	estimate	var1	var2	p.value
Arabidopsis	dnds	Ubiquitin__system	Chromosome__and__associated__proteins	0.2413793
Arabidopsis	dnds	Ubiquitin__system	Cytochrome__P450	0.0344828
Arabidopsis	dnds	Ubiquitin__system	Cytoskeleton__proteins	0.0344828
Arabidopsis	dnds	Ubiquitin__system	DNA__repair__and__recombination__proteins	0.3793103
Arabidopsis	dnds	Ubiquitin__system	DNA__replication__proteins	0.2413793
Arabidopsis	dnds	Ubiquitin__system	Exosome	0.0344828
Arabidopsis	dnds	Ubiquitin__system	G__protein__coupled__receptors	0.0344828
Arabidopsis	dnds	Ubiquitin__system	Glycosyltransferases	0.0344828
Arabidopsis	dnds	Ubiquitin__system	Ion__channels	0.0344828
Arabidopsis	dnds	Ubiquitin__system	Lipid__biosynthesis__proteins	0.0344828
Arabidopsis	dnds	Ubiquitin__system	Membrane__trafficking	0.0344828
Arabidopsis	dnds	Ubiquitin__system	Messenger__RNA__biogenesis	0.1034483
Arabidopsis	dnds	Ubiquitin__system	Mitochondrial__biogenesis	0.3103448
Arabidopsis	dnds	Ubiquitin__system	Peptidases	0.0344828
Arabidopsis	dnds	Ubiquitin__system	Photosynthesis__proteins	0.0344828
Arabidopsis	dnds	Ubiquitin__system	Proteasome	0.0344828
Arabidopsis	dnds	Ubiquitin__system	Protein__kinases	0.0344828
Arabidopsis	dnds	Ubiquitin__system	Protein__phosphatases__and__associated__proteins	0.0344828
Arabidopsis	dnds	Ubiquitin__system	Ribosome	0.0344828
Arabidopsis	dnds	Ubiquitin__system	Ribosome__biogenesis	0.7931034
Arabidopsis	dnds	Ubiquitin__system	Spliceosome	0.1034483
Arabidopsis	dnds	Ubiquitin__system	Transcription__factors	0.5172414
Arabidopsis	dnds	Ubiquitin__system	Transcription__machinery	0.5862069
Arabidopsis	dnds	Ubiquitin__system	Transfer__RNA__biogenesis	0.1034483
Arabidopsis	dnds	Ubiquitin__system	Translation__factors	0.1034483
Arabidopsis	dnds	Ubiquitin__system	Transporters	0.0344828
Arabidopsis	omegaNA	Chaperones__and__folding__catalysts	Amino__acid__related__enzymes	0.5172414
Arabidopsis	omegaNA	Chromosome__and__associated__proteins	Amino__acid__related__enzymes	0.1034483
Arabidopsis	omegaNA	Chromosome__and__associated__proteins	Chaperones__and__folding__catalysts	0.0344828
Arabidopsis	omegaNA	Cytochrome__P450	Amino__acid__related__enzymes	0.3793103
Arabidopsis	omegaNA	Cytochrome__P450	Chaperones__and__folding__catalysts	0.7931034
Arabidopsis	omegaNA	Cytochrome__P450	Chromosome__and__associated__proteins	0.0344828
Arabidopsis	omegaNA	Cytoskeleton__proteins	Amino__acid__related__enzymes	0.7241379
Arabidopsis	omegaNA	Cytoskeleton__proteins	Chaperones__and__folding__catalysts	0.5172414
Arabidopsis	omegaNA	Cytoskeleton__proteins	Chromosome__and__associated__proteins	0.2413793
Arabidopsis	omegaNA	Cytoskeleton__proteins	Cytochrome__P450	0.5172414
Arabidopsis	omegaNA	DNA__repair__and__recombination__proteins	Amino__acid__related__enzymes	0.0344828
Arabidopsis	omegaNA	DNA__repair__and__recombination__proteins	Chaperones__and__folding__catalysts	0.0344828
Arabidopsis	omegaNA	DNA__repair__and__recombination__proteins	Chromosome__and__associated__proteins	0.4482759
Arabidopsis	omegaNA	DNA__repair__and__recombination__proteins	Cytochrome__P450	0.0344828
Arabidopsis	omegaNA	DNA__repair__and__recombination__proteins	Cytoskeleton__proteins	0.1724138
Arabidopsis	omegaNA	DNA__replication__proteins	Amino__acid__related__enzymes	0.1034483
Arabidopsis	omegaNA	DNA__replication__proteins	Chaperones__and__folding__catalysts	0.0344828
Arabidopsis	omegaNA	DNA__replication__proteins	Chromosome__and__associated__proteins	0.4482759
Arabidopsis	omegaNA	DNA__replication__proteins	Cytochrome__P450	0.1034483
Arabidopsis	omegaNA	DNA__replication__proteins	Cytoskeleton__proteins	0.3793103
Arabidopsis	omegaNA	DNA__replication__proteins	DNA__repair__and__recombination__proteins	0.1724138
Arabidopsis	omegaNA	Exosome	Amino__acid__related__enzymes	0.2413793
Arabidopsis	omegaNA	Exosome	Chaperones__and__folding__catalysts	0.6551724
Arabidopsis	omegaNA	Exosome	Chromosome__and__associated__proteins	0.0344828
Arabidopsis	omegaNA	Exosome	Cytochrome__P450	0.7241379
Arabidopsis	omegaNA	Exosome	Cytoskeleton__proteins	0.2413793
Arabidopsis	omegaNA	Exosome	DNA__repair__and__recombination__proteins	0.0344828
Arabidopsis	omegaNA	Exosome	DNA__replication__proteins	0.1034483
Arabidopsis	omegaNA	G__protein__coupled__receptors	Amino__acid__related__enzymes	0.9310345
Arabidopsis	omegaNA	G__protein__coupled__receptors	Chaperones__and__folding__catalysts	0.6551724
Arabidopsis	omegaNA	G__protein__coupled__receptors	Chromosome__and__associated__proteins	0.0344828
Arabidopsis	omegaNA	G__protein__coupled__receptors	Cytochrome__P450	0.3793103
Arabidopsis	omegaNA	G__protein__coupled__receptors	Cytoskeleton__proteins	1.0000000
Arabidopsis	omegaNA	G__protein__coupled__receptors	DNA__repair__and__recombination__proteins	0.0344828
Arabidopsis	omegaNA	G__protein__coupled__receptors	DNA__replication__proteins	0.0344828

(continued)

species	estimate	var1	var2	p.value
Arabidopsis	omegaNA	G__protein__coupled__receptors	Exosome	0.2413793
Arabidopsis	omegaNA	Glycosyltransferases	Amino__acid__related__enzymes	0.5172414
Arabidopsis	omegaNA	Glycosyltransferases	Chaperones__and__folding__catalysts	0.9310345
Arabidopsis	omegaNA	Glycosyltransferases	Chromosome__and__associated__proteins	0.0344828
Arabidopsis	omegaNA	Glycosyltransferases	Cytochrome__P450	0.4482759
Arabidopsis	omegaNA	Glycosyltransferases	Cytoskeleton__proteins	0.6551724
Arabidopsis	omegaNA	Glycosyltransferases	DNA__repair__and__recombination__proteins	0.0344828
Arabidopsis	omegaNA	Glycosyltransferases	DNA__replication__proteins	0.0344828
Arabidopsis	omegaNA	Glycosyltransferases	Exosome	0.5172414
Arabidopsis	omegaNA	Glycosyltransferases	G__protein__coupled__receptors	0.3793103
Arabidopsis	omegaNA	Ion__channels	Amino__acid__related__enzymes	0.0344828
Arabidopsis	omegaNA	Ion__channels	Chaperones__and__folding__catalysts	0.0344828
Arabidopsis	omegaNA	Ion__channels	Chromosome__and__associated__proteins	0.0344828
Arabidopsis	omegaNA	Ion__channels	Cytochrome__P450	0.0344828
Arabidopsis	omegaNA	Ion__channels	Cytoskeleton__proteins	0.0344828
Arabidopsis	omegaNA	Ion__channels	DNA__repair__and__recombination__proteins	0.0344828
Arabidopsis	omegaNA	Ion__channels	DNA__replication__proteins	0.0344828
Arabidopsis	omegaNA	Ion__channels	Exosome	0.1034483
Arabidopsis	omegaNA	Ion__channels	G__protein__coupled__receptors	0.1724138
Arabidopsis	omegaNA	Ion__channels	Glycosyltransferases	0.0344828
Arabidopsis	omegaNA	Lipid__biosynthesis__proteins	Amino__acid__related__enzymes	0.3103448
Arabidopsis	omegaNA	Lipid__biosynthesis__proteins	Chaperones__and__folding__catalysts	0.7241379
Arabidopsis	omegaNA	Lipid__biosynthesis__proteins	Chromosome__and__associated__proteins	0.0344828
Arabidopsis	omegaNA	Lipid__biosynthesis__proteins	Cytochrome__P450	0.7931034
Arabidopsis	omegaNA	Lipid__biosynthesis__proteins	Cytoskeleton__proteins	0.2413793
Arabidopsis	omegaNA	Lipid__biosynthesis__proteins	DNA__repair__and__recombination__proteins	0.0344828
Arabidopsis	omegaNA	Lipid__biosynthesis__proteins	DNA__replication__proteins	0.0344828
Arabidopsis	omegaNA	Lipid__biosynthesis__proteins	Exosome	0.8620690
Arabidopsis	omegaNA	Lipid__biosynthesis__proteins	G__protein__coupled__receptors	0.3103448
Arabidopsis	omegaNA	Lipid__biosynthesis__proteins	Glycosyltransferases	0.4482759
Arabidopsis	omegaNA	Lipid__biosynthesis__proteins	Ion__channels	0.1724138
Arabidopsis	omegaNA	Membrane__trafficking	Amino__acid__related__enzymes	0.8620690
Arabidopsis	omegaNA	Membrane__trafficking	Chaperones__and__folding__catalysts	0.5172414
Arabidopsis	omegaNA	Membrane__trafficking	Chromosome__and__associated__proteins	0.0344828
Arabidopsis	omegaNA	Membrane__trafficking	Cytochrome__P450	0.3103448
Arabidopsis	omegaNA	Membrane__trafficking	Cytoskeleton__proteins	0.6551724
Arabidopsis	omegaNA	Membrane__trafficking	DNA__repair__and__recombination__proteins	0.0344828
Arabidopsis	omegaNA	Membrane__trafficking	DNA__replication__proteins	0.0344828
Arabidopsis	omegaNA	Membrane__trafficking	Exosome	0.3103448
Arabidopsis	omegaNA	Membrane__trafficking	G__protein__coupled__receptors	0.7931034
Arabidopsis	omegaNA	Membrane__trafficking	Glycosyltransferases	0.5862069
Arabidopsis	omegaNA	Membrane__trafficking	Ion__channels	0.0344828
Arabidopsis	omegaNA	Membrane__trafficking	Lipid__biosynthesis__proteins	0.2413793
Arabidopsis	omegaNA	Messenger__RNA__biogenesis	Amino__acid__related__enzymes	0.7241379
Arabidopsis	omegaNA	Messenger__RNA__biogenesis	Chaperones__and__folding__catalysts	0.2413793
Arabidopsis	omegaNA	Messenger__RNA__biogenesis	Chromosome__and__associated__proteins	0.3793103
Arabidopsis	omegaNA	Messenger__RNA__biogenesis	Cytochrome__P450	0.1724138
Arabidopsis	omegaNA	Messenger__RNA__biogenesis	Cytoskeleton__proteins	0.6551724
Arabidopsis	omegaNA	Messenger__RNA__biogenesis	DNA__repair__and__recombination__proteins	0.0344828
Arabidopsis	omegaNA	Messenger__RNA__biogenesis	DNA__replication__proteins	0.5862069
Arabidopsis	omegaNA	Messenger__RNA__biogenesis	Exosome	0.1724138
Arabidopsis	omegaNA	Messenger__RNA__biogenesis	G__protein__coupled__receptors	0.4482759
Arabidopsis	omegaNA	Messenger__RNA__biogenesis	Glycosyltransferases	0.2413793
Arabidopsis	omegaNA	Messenger__RNA__biogenesis	Ion__channels	0.0344828
Arabidopsis	omegaNA	Messenger__RNA__biogenesis	Lipid__biosynthesis__proteins	0.1724138
Arabidopsis	omegaNA	Messenger__RNA__biogenesis	Membrane__trafficking	0.3793103
Arabidopsis	omegaNA	Mitochondrial__biogenesis	Amino__acid__related__enzymes	0.1034483
Arabidopsis	omegaNA	Mitochondrial__biogenesis	Chaperones__and__folding__catalysts	0.0344828
Arabidopsis	omegaNA	Mitochondrial__biogenesis	Chromosome__and__associated__proteins	0.7931034
Arabidopsis	omegaNA	Mitochondrial__biogenesis	Cytochrome__P450	0.0344828
Arabidopsis	omegaNA	Mitochondrial__biogenesis	Cytoskeleton__proteins	0.1724138
Arabidopsis	omegaNA	Mitochondrial__biogenesis	DNA__repair__and__recombination__proteins	0.3103448

(continued)

species	estimate	var1	var2	p.value
Arabidopsis	omegaNA	Mitochondrial__biogenesis	DNA__replication__proteins	0.5172414
Arabidopsis	omegaNA	Mitochondrial__biogenesis	Exosome	0.0344828
Arabidopsis	omegaNA	Mitochondrial__biogenesis	G__protein__coupled__receptors	0.0344828
Arabidopsis	omegaNA	Mitochondrial__biogenesis	Glycosyltransferases	0.0344828
Arabidopsis	omegaNA	Mitochondrial__biogenesis	Ion__channels	0.0344828
Arabidopsis	omegaNA	Mitochondrial__biogenesis	Lipid__biosynthesis__proteins	0.0344828
Arabidopsis	omegaNA	Mitochondrial__biogenesis	Membrane__trafficking	0.0344828
Arabidopsis	omegaNA	Mitochondrial__biogenesis	Messenger__RNA__biogenesis	0.2413793
Arabidopsis	omegaNA	Peptidases	Amino__acid__related__enzymes	0.7241379
Arabidopsis	omegaNA	Peptidases	Chaperones__and__folding__catalysts	0.6551724
Arabidopsis	omegaNA	Peptidases	Chromosome__and__associated__proteins	0.0344828
Arabidopsis	omegaNA	Peptidases	Cytochrome__P450	0.2413793
Arabidopsis	omegaNA	Peptidases	Cytoskeleton__proteins	0.7931034
Arabidopsis	omegaNA	Peptidases	DNA__repair__and__recombination__proteins	0.0344828
Arabidopsis	omegaNA	Peptidases	DNA__replication__proteins	0.0344828
Arabidopsis	omegaNA	Peptidases	Exosome	0.3103448
Arabidopsis	omegaNA	Peptidases	G__protein__coupled__receptors	0.7931034
Arabidopsis	omegaNA	Peptidases	Glycosyltransferases	0.5172414
Arabidopsis	omegaNA	Peptidases	Ion__channels	0.0344828
Arabidopsis	omegaNA	Peptidases	Lipid__biosynthesis__proteins	0.1724138
Arabidopsis	omegaNA	Peptidases	Membrane__trafficking	0.8620690
Arabidopsis	omegaNA	Peptidases	Messenger__RNA__biogenesis	0.3793103
Arabidopsis	omegaNA	Peptidases	Mitochondrial__biogenesis	0.0344828
Arabidopsis	omegaNA	Photosynthesis__proteins	Amino__acid__related__enzymes	0.3103448
Arabidopsis	omegaNA	Photosynthesis__proteins	Chaperones__and__folding__catalysts	0.5172414
Arabidopsis	omegaNA	Photosynthesis__proteins	Chromosome__and__associated__proteins	0.0344828
Arabidopsis	omegaNA	Photosynthesis__proteins	Cytochrome__P450	0.5862069
Arabidopsis	omegaNA	Photosynthesis__proteins	Cytoskeleton__proteins	0.3103448
Arabidopsis	omegaNA	Photosynthesis__proteins	DNA__repair__and__recombination__proteins	0.0344828
Arabidopsis	omegaNA	Photosynthesis__proteins	DNA__replication__proteins	0.1034483
Arabidopsis	omegaNA	Photosynthesis__proteins	Exosome	0.7241379
Arabidopsis	omegaNA	Photosynthesis__proteins	G__protein__coupled__receptors	0.2413793
Arabidopsis	omegaNA	Photosynthesis__proteins	Glycosyltransferases	0.3103448
Arabidopsis	omegaNA	Photosynthesis__proteins	Ion__channels	0.3793103
Arabidopsis	omegaNA	Photosynthesis__proteins	Lipid__biosynthesis__proteins	0.8620690
Arabidopsis	omegaNA	Photosynthesis__proteins	Membrane__trafficking	0.1034483
Arabidopsis	omegaNA	Photosynthesis__proteins	Messenger__RNA__biogenesis	0.1724138
Arabidopsis	omegaNA	Photosynthesis__proteins	Mitochondrial__biogenesis	0.0344828
Arabidopsis	omegaNA	Photosynthesis__proteins	Peptidases	0.3103448
Arabidopsis	omegaNA	Proteasome	Amino__acid__related__enzymes	0.2413793
Arabidopsis	omegaNA	Proteasome	Chaperones__and__folding__catalysts	0.5862069
Arabidopsis	omegaNA	Proteasome	Chromosome__and__associated__proteins	0.0344828
Arabidopsis	omegaNA	Proteasome	Cytochrome__P450	0.7931034
Arabidopsis	omegaNA	Proteasome	Cytoskeleton__proteins	0.2413793
Arabidopsis	omegaNA	Proteasome	DNA__repair__and__recombination__proteins	0.0344828
Arabidopsis	omegaNA	Proteasome	DNA__replication__proteins	0.0344828
Arabidopsis	omegaNA	Proteasome	Exosome	0.7241379
Arabidopsis	omegaNA	Proteasome	G__protein__coupled__receptors	0.1724138
Arabidopsis	omegaNA	Proteasome	Glycosyltransferases	0.2413793
Arabidopsis	omegaNA	Proteasome	Ion__channels	0.5172414
Arabidopsis	omegaNA	Proteasome	Lipid__biosynthesis__proteins	0.7241379
Arabidopsis	omegaNA	Proteasome	Membrane__trafficking	0.1724138
Arabidopsis	omegaNA	Proteasome	Messenger__RNA__biogenesis	0.0344828
Arabidopsis	omegaNA	Proteasome	Mitochondrial__biogenesis	0.0344828
Arabidopsis	omegaNA	Proteasome	Peptidases	0.3793103
Arabidopsis	omegaNA	Proteasome	Photosynthesis__proteins	0.8620690
Arabidopsis	omegaNA	Protein__kinases	Amino__acid__related__enzymes	0.5862069
Arabidopsis	omegaNA	Protein__kinases	Chaperones__and__folding__catalysts	0.9310345
Arabidopsis	omegaNA	Protein__kinases	Chromosome__and__associated__proteins	0.0344828
Arabidopsis	omegaNA	Protein__kinases	Cytochrome__P450	0.7241379
Arabidopsis	omegaNA	Protein__kinases	Cytoskeleton__proteins	0.6551724
Arabidopsis	omegaNA	Protein__kinases	DNA__repair__and__recombination__proteins	0.0344828

(continued)

species	estimate	var1	var2	p.value
Arabidopsis	omegaNA	Protein__kinases	DNA__replication__proteins	0.0344828
Arabidopsis	omegaNA	Protein__kinases	Exosome	0.5862069
Arabidopsis	omegaNA	Protein__kinases	G__protein__coupled__receptors	0.7241379
Arabidopsis	omegaNA	Protein__kinases	Glycosyltransferases	0.9310345
Arabidopsis	omegaNA	Protein__kinases	Ion__channels	0.0344828
Arabidopsis	omegaNA	Protein__kinases	Lipid__biosynthesis__proteins	0.5862069
Arabidopsis	omegaNA	Protein__kinases	Membrane__trafficking	0.5172414
Arabidopsis	omegaNA	Protein__kinases	Messenger__RNA__biogenesis	0.1724138
Arabidopsis	omegaNA	Protein__kinases	Mitochondrial__biogenesis	0.0344828
Arabidopsis	omegaNA	Protein__kinases	Peptidases	0.7241379
Arabidopsis	omegaNA	Protein__kinases	Photosynthesis__proteins	0.5172414
Arabidopsis	omegaNA	Protein__kinases	Proteasome	0.3793103
Arabidopsis	omegaNA	Protein__phosphatases__and__associated__proteins	Amino__acid__related__enzymes	0.7241379
Arabidopsis	omegaNA	Protein__phosphatases__and__associated__proteins	Chaperones__and__folding__catalysts	1.0000000
Arabidopsis	omegaNA	Protein__phosphatases__and__associated__proteins	Chromosome__and__associated__proteins	0.1034483
Arabidopsis	omegaNA	Protein__phosphatases__and__associated__proteins	Cytochrome__P450	0.5862069
Arabidopsis	omegaNA	Protein__phosphatases__and__associated__proteins	Cytoskeleton__proteins	0.6551724
Arabidopsis	omegaNA	Protein__phosphatases__and__associated__proteins	DNA__repair__and__recombination__proteins	0.0344828
Arabidopsis	omegaNA	Protein__phosphatases__and__associated__proteins	DNA__replication__proteins	0.1034483
Arabidopsis	omegaNA	Protein__phosphatases__and__associated__proteins	Exosome	0.6551724
Arabidopsis	omegaNA	Protein__phosphatases__and__associated__proteins	G__protein__coupled__receptors	0.7241379
Arabidopsis	omegaNA	Protein__phosphatases__and__associated__proteins	Glycosyltransferases	1.0000000
Arabidopsis	omegaNA	Protein__phosphatases__and__associated__proteins	Ion__channels	0.0344828
Arabidopsis	omegaNA	Protein__phosphatases__and__associated__proteins	Lipid__biosynthesis__proteins	0.7241379
Arabidopsis	omegaNA	Protein__phosphatases__and__associated__proteins	Membrane__trafficking	0.7931034
Arabidopsis	omegaNA	Protein__phosphatases__and__associated__proteins	Messenger__RNA__biogenesis	0.6551724
Arabidopsis	omegaNA	Protein__phosphatases__and__associated__proteins	Mitochondrial__biogenesis	0.1034483
Arabidopsis	omegaNA	Protein__phosphatases__and__associated__proteins	Peptidases	0.7931034
Arabidopsis	omegaNA	Protein__phosphatases__and__associated__proteins	Photosynthesis__proteins	0.4482759
Arabidopsis	omegaNA	Protein__phosphatases__and__associated__proteins	Proteasome	0.3793103
Arabidopsis	omegaNA	Protein__phosphatases__and__associated__proteins	Protein__kinases	0.9310345
Arabidopsis	omegaNA	Ribosome	Amino__acid__related__enzymes	0.1034483
Arabidopsis	omegaNA	Ribosome	Chaperones__and__folding__catalysts	0.4482759
Arabidopsis	omegaNA	Ribosome	Chromosome__and__associated__proteins	0.0344828
Arabidopsis	omegaNA	Ribosome	Cytochrome__P450	0.5172414
Arabidopsis	omegaNA	Ribosome	Cytoskeleton__proteins	0.1724138
Arabidopsis	omegaNA	Ribosome	DNA__repair__and__recombination__proteins	0.0344828
Arabidopsis	omegaNA	Ribosome	DNA__replication__proteins	0.0344828
Arabidopsis	omegaNA	Ribosome	Exosome	1.0000000
Arabidopsis	omegaNA	Ribosome	G__protein__coupled__receptors	0.2413793
Arabidopsis	omegaNA	Ribosome	Glycosyltransferases	0.3793103
Arabidopsis	omegaNA	Ribosome	Ion__channels	0.3793103
Arabidopsis	omegaNA	Ribosome	Lipid__biosynthesis__proteins	0.9310345
Arabidopsis	omegaNA	Ribosome	Membrane__trafficking	0.0344828
Arabidopsis	omegaNA	Ribosome	Messenger__RNA__biogenesis	0.1034483
Arabidopsis	omegaNA	Ribosome	Mitochondrial__biogenesis	0.0344828
Arabidopsis	omegaNA	Ribosome	Peptidases	0.1034483
Arabidopsis	omegaNA	Ribosome	Photosynthesis__proteins	0.9310345
Arabidopsis	omegaNA	Ribosome	Proteasome	0.9310345
Arabidopsis	omegaNA	Ribosome	Protein__kinases	0.3103448
Arabidopsis	omegaNA	Ribosome	Protein__phosphatases__and__associated__proteins	0.4482759
Arabidopsis	omegaNA	Ribosome__biogenesis	Amino__acid__related__enzymes	0.9310345
Arabidopsis	omegaNA	Ribosome__biogenesis	Chaperones__and__folding__catalysts	0.3793103
Arabidopsis	omegaNA	Ribosome__biogenesis	Chromosome__and__associated__proteins	0.0344828
Arabidopsis	omegaNA	Ribosome__biogenesis	Cytochrome__P450	0.2413793
Arabidopsis	omegaNA	Ribosome__biogenesis	Cytoskeleton__proteins	0.9310345
Arabidopsis	omegaNA	Ribosome__biogenesis	DNA__repair__and__recombination__proteins	0.0344828
Arabidopsis	omegaNA	Ribosome__biogenesis	DNA__replication__proteins	0.1724138
Arabidopsis	omegaNA	Ribosome__biogenesis	Exosome	0.2413793
Arabidopsis	omegaNA	Ribosome__biogenesis	G__protein__coupled__receptors	0.8620690
Arabidopsis	omegaNA	Ribosome__biogenesis	Glycosyltransferases	0.4482759

(continued)

species	estimate	var1	var2	p.value
Arabidopsis	omegaNA	Ribosome__biogenesis	Ion__channels	0.0344828
Arabidopsis	omegaNA	Ribosome__biogenesis	Lipid__biosynthesis__proteins	0.1724138
Arabidopsis	omegaNA	Ribosome__biogenesis	Membrane__trafficking	0.7931034
Arabidopsis	omegaNA	Ribosome__biogenesis	Messenger__RNA__biogenesis	0.7241379
Arabidopsis	omegaNA	Ribosome__biogenesis	Mitochondrial__biogenesis	0.1034483
Arabidopsis	omegaNA	Ribosome__biogenesis	Peptidases	0.5862069
Arabidopsis	omegaNA	Ribosome__biogenesis	Photosynthesis__proteins	0.2413793
Arabidopsis	omegaNA	Ribosome__biogenesis	Proteasome	0.1034483
Arabidopsis	omegaNA	Ribosome__biogenesis	Protein__kinases	0.3103448
Arabidopsis	omegaNA	Ribosome__biogenesis	Protein__phosphatases__and__associated__proteins	0.5172414
Arabidopsis	omegaNA	Ribosome__biogenesis	Ribosome	0.1034483
Arabidopsis	omegaNA	Spliceosome	Amino__acid__related__enzymes	0.4482759
Arabidopsis	omegaNA	Spliceosome	Chaperones__and__folding__catalysts	0.3103448
Arabidopsis	omegaNA	Spliceosome	Chromosome__and__associated__proteins	0.1724138
Arabidopsis	omegaNA	Spliceosome	Cytochrome__P450	0.1034483
Arabidopsis	omegaNA	Spliceosome	Cytoskeleton__proteins	0.6551724
Arabidopsis	omegaNA	Spliceosome	DNA__repair__and__recombination__proteins	0.0344828
Arabidopsis	omegaNA	Spliceosome	DNA__replication__proteins	0.3103448
Arabidopsis	omegaNA	Spliceosome	Exosome	0.0344828
Arabidopsis	omegaNA	Spliceosome	G__protein__coupled__receptors	0.1724138
Arabidopsis	omegaNA	Spliceosome	Glycosyltransferases	0.2413793
Arabidopsis	omegaNA	Spliceosome	Ion__channels	0.0344828
Arabidopsis	omegaNA	Spliceosome	Lipid__biosynthesis__proteins	0.1034483
Arabidopsis	omegaNA	Spliceosome	Membrane__trafficking	0.3103448
Arabidopsis	omegaNA	Spliceosome	Messenger__RNA__biogenesis	0.8620690
Arabidopsis	omegaNA	Spliceosome	Mitochondrial__biogenesis	0.1034483
Arabidopsis	omegaNA	Spliceosome	Peptidases	0.3103448
Arabidopsis	omegaNA	Spliceosome	Photosynthesis__proteins	0.1034483
Arabidopsis	omegaNA	Spliceosome	Proteasome	0.1724138
Arabidopsis	omegaNA	Spliceosome	Protein__kinases	0.2413793
Arabidopsis	omegaNA	Spliceosome	Protein__phosphatases__and__associated__proteins	0.4482759
Arabidopsis	omegaNA	Spliceosome	Ribosome	0.1034483
Arabidopsis	omegaNA	Spliceosome	Ribosome__biogenesis	0.6551724
Arabidopsis	omegaNA	Transcription__factors	Amino__acid__related__enzymes	0.1034483
Arabidopsis	omegaNA	Transcription__factors	Chaperones__and__folding__catalysts	0.1034483
Arabidopsis	omegaNA	Transcription__factors	Chromosome__and__associated__proteins	0.7931034
Arabidopsis	omegaNA	Transcription__factors	Cytochrome__P450	0.0344828
Arabidopsis	omegaNA	Transcription__factors	Cytoskeleton__proteins	0.2413793
Arabidopsis	omegaNA	Transcription__factors	DNA__repair__and__recombination__proteins	0.5172414
Arabidopsis	omegaNA	Transcription__factors	DNA__replication__proteins	0.3103448
Arabidopsis	omegaNA	Transcription__factors	Exosome	0.0344828
Arabidopsis	omegaNA	Transcription__factors	G__protein__coupled__receptors	0.0344828
Arabidopsis	omegaNA	Transcription__factors	Glycosyltransferases	0.0344828
Arabidopsis	omegaNA	Transcription__factors	Ion__channels	0.0344828
Arabidopsis	omegaNA	Transcription__factors	Lipid__biosynthesis__proteins	0.0344828
Arabidopsis	omegaNA	Transcription__factors	Membrane__trafficking	0.0344828
Arabidopsis	omegaNA	Transcription__factors	Messenger__RNA__biogenesis	0.1724138
Arabidopsis	omegaNA	Transcription__factors	Mitochondrial__biogenesis	0.7931034
Arabidopsis	omegaNA	Transcription__factors	Peptidases	0.0344828
Arabidopsis	omegaNA	Transcription__factors	Photosynthesis__proteins	0.0344828
Arabidopsis	omegaNA	Transcription__factors	Proteasome	0.0344828
Arabidopsis	omegaNA	Transcription__factors	Protein__kinases	0.0344828
Arabidopsis	omegaNA	Transcription__factors	Protein__phosphatases__and__associated__proteins	0.1034483
Arabidopsis	omegaNA	Transcription__factors	Ribosome	0.0344828
Arabidopsis	omegaNA	Transcription__factors	Ribosome__biogenesis	0.0344828
Arabidopsis	omegaNA	Transcription__factors	Spliceosome	0.1724138
Arabidopsis	omegaNA	Transcription__machinery	Amino__acid__related__enzymes	0.5862069
Arabidopsis	omegaNA	Transcription__machinery	Chaperones__and__folding__catalysts	0.3793103
Arabidopsis	omegaNA	Transcription__machinery	Chromosome__and__associated__proteins	0.1034483
Arabidopsis	omegaNA	Transcription__machinery	Cytochrome__P450	0.2413793
Arabidopsis	omegaNA	Transcription__machinery	Cytoskeleton__proteins	1.0000000
Arabidopsis	omegaNA	Transcription__machinery	DNA__repair__and__recombination__proteins	0.0344828

(continued)

species	estimate	var1	var2	p.value
Arabidopsis	omegaNA	Transcription__machinery	DNA__replication__proteins	0.2413793
Arabidopsis	omegaNA	Transcription__machinery	Exosome	0.3103448
Arabidopsis	omegaNA	Transcription__machinery	G__protein__coupled__receptors	0.5172414
Arabidopsis	omegaNA	Transcription__machinery	Glycosyltransferases	0.3103448
Arabidopsis	omegaNA	Transcription__machinery	Ion__channels	0.0344828
Arabidopsis	omegaNA	Transcription__machinery	Lipid__biosynthesis__proteins	0.2413793
Arabidopsis	omegaNA	Transcription__machinery	Membrane__trafficking	0.6551724
Arabidopsis	omegaNA	Transcription__machinery	Messenger__RNA__biogenesis	0.6551724
Arabidopsis	omegaNA	Transcription__machinery	Mitochondrial__biogenesis	0.1034483
Arabidopsis	omegaNA	Transcription__machinery	Peptidases	0.6551724
Arabidopsis	omegaNA	Transcription__machinery	Photosynthesis__proteins	0.1034483
Arabidopsis	omegaNA	Transcription__machinery	Proteasome	0.0344828
Arabidopsis	omegaNA	Transcription__machinery	Protein__kinases	0.3793103
Arabidopsis	omegaNA	Transcription__machinery	Protein__phosphatases__and__associated__proteins	0.7241379
Arabidopsis	omegaNA	Transcription__machinery	Ribosome	0.1034483
Arabidopsis	omegaNA	Transcription__machinery	Ribosome__biogenesis	0.7241379
Arabidopsis	omegaNA	Transcription__machinery	Spliceosome	0.4482759
Arabidopsis	omegaNA	Transcription__machinery	Transcription__factors	0.1034483
Arabidopsis	omegaNA	Transfer__RNA__biogenesis	Amino__acid__related__enzymes	0.1724138
Arabidopsis	omegaNA	Transfer__RNA__biogenesis	Chaperones__and__folding__catalysts	0.1034483
Arabidopsis	omegaNA	Transfer__RNA__biogenesis	Chromosome__and__associated__proteins	0.6551724
Arabidopsis	omegaNA	Transfer__RNA__biogenesis	Cytochrome__P450	0.1034483
Arabidopsis	omegaNA	Transfer__RNA__biogenesis	Cytoskeleton__proteins	0.3793103
Arabidopsis	omegaNA	Transfer__RNA__biogenesis	DNA__repair__and__recombination__proteins	0.2413793
Arabidopsis	omegaNA	Transfer__RNA__biogenesis	DNA__replication__proteins	0.7241379
Arabidopsis	omegaNA	Transfer__RNA__biogenesis	Exosome	0.0344828
Arabidopsis	omegaNA	Transfer__RNA__biogenesis	G__protein__coupled__receptors	0.1034483
Arabidopsis	omegaNA	Transfer__RNA__biogenesis	Glycosyltransferases	0.0344828
Arabidopsis	omegaNA	Transfer__RNA__biogenesis	Ion__channels	0.0344828
Arabidopsis	omegaNA	Transfer__RNA__biogenesis	Lipid__biosynthesis__proteins	0.0344828
Arabidopsis	omegaNA	Transfer__RNA__biogenesis	Membrane__trafficking	0.1034483
Arabidopsis	omegaNA	Transfer__RNA__biogenesis	Messenger__RNA__biogenesis	0.5172414
Arabidopsis	omegaNA	Transfer__RNA__biogenesis	Mitochondrial__biogenesis	0.6551724
Arabidopsis	omegaNA	Transfer__RNA__biogenesis	Peptidases	0.0344828
Arabidopsis	omegaNA	Transfer__RNA__biogenesis	Photosynthesis__proteins	0.0344828
Arabidopsis	omegaNA	Transfer__RNA__biogenesis	Proteasome	0.0344828
Arabidopsis	omegaNA	Transfer__RNA__biogenesis	Protein__kinases	0.0344828
Arabidopsis	omegaNA	Transfer__RNA__biogenesis	Protein__phosphatases__and__associated__proteins	0.2413793
Arabidopsis	omegaNA	Transfer__RNA__biogenesis	Ribosome	0.0344828
Arabidopsis	omegaNA	Transfer__RNA__biogenesis	Ribosome__biogenesis	0.1724138
Arabidopsis	omegaNA	Transfer__RNA__biogenesis	Spliceosome	0.3793103
Arabidopsis	omegaNA	Transfer__RNA__biogenesis	Transcription__factors	0.5172414
Arabidopsis	omegaNA	Transfer__RNA__biogenesis	Transcription__machinery	0.4482759
Arabidopsis	omegaNA	Translation__factors	Amino__acid__related__enzymes	0.9310345
Arabidopsis	omegaNA	Translation__factors	Chaperones__and__folding__catalysts	0.9310345
Arabidopsis	omegaNA	Translation__factors	Chromosome__and__associated__proteins	0.0344828
Arabidopsis	omegaNA	Translation__factors	Cytochrome__P450	0.4482759
Arabidopsis	omegaNA	Translation__factors	Cytoskeleton__proteins	1.0000000
Arabidopsis	omegaNA	Translation__factors	DNA__repair__and__recombination__proteins	0.0344828
Arabidopsis	omegaNA	Translation__factors	DNA__replication__proteins	0.1724138
Arabidopsis	omegaNA	Translation__factors	Exosome	0.5862069
Arabidopsis	omegaNA	Translation__factors	G__protein__coupled__receptors	0.7931034
Arabidopsis	omegaNA	Translation__factors	Glycosyltransferases	0.6551724
Arabidopsis	omegaNA	Translation__factors	Ion__channels	0.1034483
Arabidopsis	omegaNA	Translation__factors	Lipid__biosynthesis__proteins	0.3103448
Arabidopsis	omegaNA	Translation__factors	Membrane__trafficking	0.9310345
Arabidopsis	omegaNA	Translation__factors	Messenger__RNA__biogenesis	0.3793103
Arabidopsis	omegaNA	Translation__factors	Mitochondrial__biogenesis	0.0344828
Arabidopsis	omegaNA	Translation__factors	Peptidases	0.7241379
Arabidopsis	omegaNA	Translation__factors	Photosynthesis__proteins	0.3793103
Arabidopsis	omegaNA	Translation__factors	Proteasome	0.3103448
Arabidopsis	omegaNA	Translation__factors	Protein__kinases	0.7931034

(continued)

species	estimate	var1	var2	p.value
Arabidopsis	omegaNA	Translation__factors	Protein__phosphatases__and__associated__proteins	0.9310345
Arabidopsis	omegaNA	Translation__factors	Ribosome	0.5172414
Arabidopsis	omegaNA	Translation__factors	Ribosome__biogenesis	0.5862069
Arabidopsis	omegaNA	Translation__factors	Spliceosome	0.3793103
Arabidopsis	omegaNA	Translation__factors	Transcription__factors	0.0344828
Arabidopsis	omegaNA	Translation__factors	Transcription__machinery	0.3793103
Arabidopsis	omegaNA	Translation__factors	Transfer__RNA__biogenesis	0.0344828
Arabidopsis	omegaNA	Transporters	Amino__acid__related__enzymes	0.2413793
Arabidopsis	omegaNA	Transporters	Chaperones__and__folding__catalysts	0.3793103
Arabidopsis	omegaNA	Transporters	Chromosome__and__associated__proteins	0.0344828
Arabidopsis	omegaNA	Transporters	Cytochrome__P450	0.6551724
Arabidopsis	omegaNA	Transporters	Cytoskeleton__proteins	0.2413793
Arabidopsis	omegaNA	Transporters	DNA__repair__and__recombination__proteins	0.0344828
Arabidopsis	omegaNA	Transporters	DNA__replication__proteins	0.0344828
Arabidopsis	omegaNA	Transporters	Exosome	1.0000000
Arabidopsis	omegaNA	Transporters	G__protein__coupled__receptors	0.2413793
Arabidopsis	omegaNA	Transporters	Glycosyltransferases	0.1724138
Arabidopsis	omegaNA	Transporters	Ion__channels	0.1034483
Arabidopsis	omegaNA	Transporters	Lipid__biosynthesis__proteins	0.7931034
Arabidopsis	omegaNA	Transporters	Membrane__trafficking	0.1034483
Arabidopsis	omegaNA	Transporters	Messenger__RNA__biogenesis	0.0344828
Arabidopsis	omegaNA	Transporters	Mitochondrial__biogenesis	0.0344828
Arabidopsis	omegaNA	Transporters	Peptidases	0.1724138
Arabidopsis	omegaNA	Transporters	Photosynthesis__proteins	1.0000000
Arabidopsis	omegaNA	Transporters	Proteasome	0.8620690
Arabidopsis	omegaNA	Transporters	Protein__kinases	0.3793103
Arabidopsis	omegaNA	Transporters	Protein__phosphatases__and__associated__proteins	0.3793103
Arabidopsis	omegaNA	Transporters	Ribosome	1.0000000
Arabidopsis	omegaNA	Transporters	Ribosome__biogenesis	0.0344828
Arabidopsis	omegaNA	Transporters	Spliceosome	0.0344828
Arabidopsis	omegaNA	Transporters	Transcription__factors	0.0344828
Arabidopsis	omegaNA	Transporters	Transcription__machinery	0.0344828
Arabidopsis	omegaNA	Transporters	Transfer__RNA__biogenesis	0.0344828
Arabidopsis	omegaNA	Transporters	Translation__factors	0.3793103
Arabidopsis	omegaNA	Ubiquitin__system	Amino__acid__related__enzymes	0.8620690
Arabidopsis	omegaNA	Ubiquitin__system	Chaperones__and__folding__catalysts	0.4482759
Arabidopsis	omegaNA	Ubiquitin__system	Chromosome__and__associated__proteins	0.1034483
Arabidopsis	omegaNA	Ubiquitin__system	Cytochrome__P450	0.2413793
Arabidopsis	omegaNA	Ubiquitin__system	Cytoskeleton__proteins	1.0000000
Arabidopsis	omegaNA	Ubiquitin__system	DNA__repair__and__recombination__proteins	0.0344828
Arabidopsis	omegaNA	Ubiquitin__system	DNA__replication__proteins	0.0344828
Arabidopsis	omegaNA	Ubiquitin__system	Exosome	0.2413793
Arabidopsis	omegaNA	Ubiquitin__system	G__protein__coupled__receptors	0.7931034
Arabidopsis	omegaNA	Ubiquitin__system	Glycosyltransferases	0.4482759
Arabidopsis	omegaNA	Ubiquitin__system	Ion__channels	0.0344828
Arabidopsis	omegaNA	Ubiquitin__system	Lipid__biosynthesis__proteins	0.1034483
Arabidopsis	omegaNA	Ubiquitin__system	Membrane__trafficking	0.6551724
Arabidopsis	omegaNA	Ubiquitin__system	Messenger__RNA__biogenesis	0.6551724
Arabidopsis	omegaNA	Ubiquitin__system	Mitochondrial__biogenesis	0.0344828
Arabidopsis	omegaNA	Ubiquitin__system	Peptidases	0.5862069
Arabidopsis	omegaNA	Ubiquitin__system	Photosynthesis__proteins	0.1724138
Arabidopsis	omegaNA	Ubiquitin__system	Proteasome	0.0344828
Arabidopsis	omegaNA	Ubiquitin__system	Protein__kinases	0.5172414
Arabidopsis	omegaNA	Ubiquitin__system	Protein__phosphatases__and__associated__proteins	0.6551724
Arabidopsis	omegaNA	Ubiquitin__system	Ribosome	0.0344828
Arabidopsis	omegaNA	Ubiquitin__system	Ribosome__biogenesis	1.0000000
Arabidopsis	omegaNA	Ubiquitin__system	Spliceosome	0.3793103
Arabidopsis	omegaNA	Ubiquitin__system	Transcription__factors	0.0344828
Arabidopsis	omegaNA	Ubiquitin__system	Transcription__machinery	0.7241379
Arabidopsis	omegaNA	Ubiquitin__system	Transfer__RNA__biogenesis	0.1724138
Arabidopsis	omegaNA	Ubiquitin__system	Translation__factors	0.6551724

(continued)

species	estimate	var1	var2	p.value
Arabidopsis	omegaNA	Ubiquitin__system	Transporters	0.0344828
Arabidopsis	omegaA	Chaperones__and__folding__catalysts	Amino__acid__related__enzymes	0.1034483
Arabidopsis	omegaA	Chromosome__and__associated__proteins	Amino__acid__related__enzymes	0.3793103
Arabidopsis	omegaA	Chromosome__and__associated__proteins	Chaperones__and__folding__catalysts	0.4482759
Arabidopsis	omegaA	Cytochrome__P450	Amino__acid__related__enzymes	0.1034483
Arabidopsis	omegaA	Cytochrome__P450	Chaperones__and__folding__catalysts	0.3103448
Arabidopsis	omegaA	Cytochrome__P450	Chromosome__and__associated__proteins	0.1724138
Arabidopsis	omegaA	Cytoskeleton__proteins	Amino__acid__related__enzymes	0.3793103
Arabidopsis	omegaA	Cytoskeleton__proteins	Chaperones__and__folding__catalysts	0.8620690
Arabidopsis	omegaA	Cytoskeleton__proteins	Chromosome__and__associated__proteins	0.7931034
Arabidopsis	omegaA	Cytoskeleton__proteins	Cytochrome__P450	0.4482759
Arabidopsis	omegaA	DNA__repair__and__recombination__proteins	Amino__acid__related__enzymes	0.9310345
Arabidopsis	omegaA	DNA__repair__and__recombination__proteins	Chaperones__and__folding__catalysts	0.2413793
Arabidopsis	omegaA	DNA__repair__and__recombination__proteins	Chromosome__and__associated__proteins	0.6551724
Arabidopsis	omegaA	DNA__repair__and__recombination__proteins	Cytochrome__P450	0.1034483
Arabidopsis	omegaA	DNA__repair__and__recombination__proteins	Cytoskeleton__proteins	0.5862069
Arabidopsis	omegaA	DNA__replication__proteins	Amino__acid__related__enzymes	0.5862069
Arabidopsis	omegaA	DNA__replication__proteins	Chaperones__and__folding__catalysts	0.5172414
Arabidopsis	omegaA	DNA__replication__proteins	Chromosome__and__associated__proteins	0.9310345
Arabidopsis	omegaA	DNA__replication__proteins	Cytochrome__P450	0.2413793
Arabidopsis	omegaA	DNA__replication__proteins	Cytoskeleton__proteins	0.8620690
Arabidopsis	omegaA	DNA__replication__proteins	DNA__repair__and__recombination__proteins	0.5862069
Arabidopsis	omegaA	Exosome	Amino__acid__related__enzymes	0.6551724
Arabidopsis	omegaA	Exosome	Chaperones__and__folding__catalysts	0.3793103
Arabidopsis	omegaA	Exosome	Chromosome__and__associated__proteins	0.9310345
Arabidopsis	omegaA	Exosome	Cytochrome__P450	0.1034483
Arabidopsis	omegaA	Exosome	Cytoskeleton__proteins	0.5862069
Arabidopsis	omegaA	Exosome	DNA__repair__and__recombination__proteins	0.5862069
Arabidopsis	omegaA	Exosome	DNA__replication__proteins	0.7241379
Arabidopsis	omegaA	G__protein__coupled__receptors	Amino__acid__related__enzymes	0.0344828
Arabidopsis	omegaA	G__protein__coupled__receptors	Chaperones__and__folding__catalysts	0.3793103
Arabidopsis	omegaA	G__protein__coupled__receptors	Chromosome__and__associated__proteins	0.0344828
Arabidopsis	omegaA	G__protein__coupled__receptors	Cytochrome__P450	0.9310345
Arabidopsis	omegaA	G__protein__coupled__receptors	Cytoskeleton__proteins	0.5862069
Arabidopsis	omegaA	G__protein__coupled__receptors	DNA__repair__and__recombination__proteins	0.0344828
Arabidopsis	omegaA	G__protein__coupled__receptors	DNA__replication__proteins	0.1724138
Arabidopsis	omegaA	G__protein__coupled__receptors	Exosome	0.0344828
Arabidopsis	omegaA	Glycosyltransferases	Amino__acid__related__enzymes	0.1034483
Arabidopsis	omegaA	Glycosyltransferases	Chaperones__and__folding__catalysts	0.8620690
Arabidopsis	omegaA	Glycosyltransferases	Chromosome__and__associated__proteins	0.7931034
Arabidopsis	omegaA	Glycosyltransferases	Cytochrome__P450	0.3103448
Arabidopsis	omegaA	Glycosyltransferases	Cytoskeleton__proteins	0.9310345
Arabidopsis	omegaA	Glycosyltransferases	DNA__repair__and__recombination__proteins	0.3103448
Arabidopsis	omegaA	Glycosyltransferases	DNA__replication__proteins	0.7241379
Arabidopsis	omegaA	Glycosyltransferases	Exosome	0.3103448
Arabidopsis	omegaA	Glycosyltransferases	G__protein__coupled__receptors	0.0344828
Arabidopsis	omegaA	Ion__channels	Amino__acid__related__enzymes	0.0344828
Arabidopsis	omegaA	Ion__channels	Chaperones__and__folding__catalysts	0.5862069
Arabidopsis	omegaA	Ion__channels	Chromosome__and__associated__proteins	0.1034483
Arabidopsis	omegaA	Ion__channels	Cytochrome__P450	0.7241379
Arabidopsis	omegaA	Ion__channels	Cytoskeleton__proteins	0.7931034
Arabidopsis	omegaA	Ion__channels	DNA__repair__and__recombination__proteins	0.1724138
Arabidopsis	omegaA	Ion__channels	DNA__replication__proteins	0.3103448
Arabidopsis	omegaA	Ion__channels	Exosome	0.0344828
Arabidopsis	omegaA	Ion__channels	G__protein__coupled__receptors	0.6551724
Arabidopsis	omegaA	Ion__channels	Glycosyltransferases	0.1034483
Arabidopsis	omegaA	Lipid__biosynthesis__proteins	Amino__acid__related__enzymes	0.0344828
Arabidopsis	omegaA	Lipid__biosynthesis__proteins	Chaperones__and__folding__catalysts	0.3103448
Arabidopsis	omegaA	Lipid__biosynthesis__proteins	Chromosome__and__associated__proteins	0.1034483
Arabidopsis	omegaA	Lipid__biosynthesis__proteins	Cytochrome__P450	0.7931034
Arabidopsis	omegaA	Lipid__biosynthesis__proteins	Cytoskeleton__proteins	0.4482759
Arabidopsis	omegaA	Lipid__biosynthesis__proteins	DNA__repair__and__recombination__proteins	0.1034483

(continued)

species	estimate	var1	var2	p.value
Arabidopsis	omegaA	Lipid__biosynthesis__proteins	DNA__replication__proteins	0.0344828
Arabidopsis	omegaA	Lipid__biosynthesis__proteins	Exosome	0.0344828
Arabidopsis	omegaA	Lipid__biosynthesis__proteins	G__protein__coupled__receptors	0.9310345
Arabidopsis	omegaA	Lipid__biosynthesis__proteins	Glycosyltransferases	0.2413793
Arabidopsis	omegaA	Lipid__biosynthesis__proteins	Ion__channels	0.6551724
Arabidopsis	omegaA	Membrane__trafficking	Amino__acid__related__enzymes	0.1034483
Arabidopsis	omegaA	Membrane__trafficking	Chaperones__and__folding__catalysts	0.7241379
Arabidopsis	omegaA	Membrane__trafficking	Chromosome__and__associated__proteins	0.7931034
Arabidopsis	omegaA	Membrane__trafficking	Cytochrome__P450	0.1724138
Arabidopsis	omegaA	Membrane__trafficking	Cytoskeleton__proteins	1.0000000
Arabidopsis	omegaA	Membrane__trafficking	DNA__repair__and__recombination__proteins	0.2413793
Arabidopsis	omegaA	Membrane__trafficking	DNA__replication__proteins	0.6551724
Arabidopsis	omegaA	Membrane__trafficking	Exosome	0.3793103
Arabidopsis	omegaA	Membrane__trafficking	G__protein__coupled__receptors	0.1724138
Arabidopsis	omegaA	Membrane__trafficking	Glycosyltransferases	0.8620690
Arabidopsis	omegaA	Membrane__trafficking	Ion__channels	0.1724138
Arabidopsis	omegaA	Membrane__trafficking	Lipid__biosynthesis__proteins	0.1724138
Arabidopsis	omegaA	Messenger__RNA__biogenesis	Amino__acid__related__enzymes	0.5172414
Arabidopsis	omegaA	Messenger__RNA__biogenesis	Chaperones__and__folding__catalysts	0.7931034
Arabidopsis	omegaA	Messenger__RNA__biogenesis	Chromosome__and__associated__proteins	0.8620690
Arabidopsis	omegaA	Messenger__RNA__biogenesis	Cytochrome__P450	0.3793103
Arabidopsis	omegaA	Messenger__RNA__biogenesis	Cytoskeleton__proteins	0.8620690
Arabidopsis	omegaA	Messenger__RNA__biogenesis	DNA__repair__and__recombination__proteins	0.5862069
Arabidopsis	omegaA	Messenger__RNA__biogenesis	DNA__replication__proteins	0.9310345
Arabidopsis	omegaA	Messenger__RNA__biogenesis	Exosome	0.7241379
Arabidopsis	omegaA	Messenger__RNA__biogenesis	G__protein__coupled__receptors	0.3103448
Arabidopsis	omegaA	Messenger__RNA__biogenesis	Glycosyltransferases	0.8620690
Arabidopsis	omegaA	Messenger__RNA__biogenesis	Ion__channels	0.4482759
Arabidopsis	omegaA	Messenger__RNA__biogenesis	Lipid__biosynthesis__proteins	0.1724138
Arabidopsis	omegaA	Messenger__RNA__biogenesis	Membrane__trafficking	0.9310345
Arabidopsis	omegaA	Mitochondrial__biogenesis	Amino__acid__related__enzymes	0.8620690
Arabidopsis	omegaA	Mitochondrial__biogenesis	Chaperones__and__folding__catalysts	0.1034483
Arabidopsis	omegaA	Mitochondrial__biogenesis	Chromosome__and__associated__proteins	0.3793103
Arabidopsis	omegaA	Mitochondrial__biogenesis	Cytochrome__P450	0.1034483
Arabidopsis	omegaA	Mitochondrial__biogenesis	Cytoskeleton__proteins	0.3793103
Arabidopsis	omegaA	Mitochondrial__biogenesis	DNA__repair__and__recombination__proteins	0.6551724
Arabidopsis	omegaA	Mitochondrial__biogenesis	DNA__replication__proteins	0.4482759
Arabidopsis	omegaA	Mitochondrial__biogenesis	Exosome	0.5172414
Arabidopsis	omegaA	Mitochondrial__biogenesis	G__protein__coupled__receptors	0.0344828
Arabidopsis	omegaA	Mitochondrial__biogenesis	Glycosyltransferases	0.0344828
Arabidopsis	omegaA	Mitochondrial__biogenesis	Ion__channels	0.0344828
Arabidopsis	omegaA	Mitochondrial__biogenesis	Lipid__biosynthesis__proteins	0.0344828
Arabidopsis	omegaA	Mitochondrial__biogenesis	Membrane__trafficking	0.2413793
Arabidopsis	omegaA	Mitochondrial__biogenesis	Messenger__RNA__biogenesis	0.3793103
Arabidopsis	omegaA	Peptidases	Amino__acid__related__enzymes	0.0344828
Arabidopsis	omegaA	Peptidases	Chaperones__and__folding__catalysts	0.7931034
Arabidopsis	omegaA	Peptidases	Chromosome__and__associated__proteins	0.5862069
Arabidopsis	omegaA	Peptidases	Cytochrome__P450	0.4482759
Arabidopsis	omegaA	Peptidases	Cytoskeleton__proteins	0.8620690
Arabidopsis	omegaA	Peptidases	DNA__repair__and__recombination__proteins	0.1724138
Arabidopsis	omegaA	Peptidases	DNA__replication__proteins	0.3793103
Arabidopsis	omegaA	Peptidases	Exosome	0.0344828
Arabidopsis	omegaA	Peptidases	G__protein__coupled__receptors	0.3793103
Arabidopsis	omegaA	Peptidases	Glycosyltransferases	0.3793103
Arabidopsis	omegaA	Peptidases	Ion__channels	0.5172414
Arabidopsis	omegaA	Peptidases	Lipid__biosynthesis__proteins	0.3793103
Arabidopsis	omegaA	Peptidases	Membrane__trafficking	0.6551724
Arabidopsis	omegaA	Peptidases	Messenger__RNA__biogenesis	0.5172414
Arabidopsis	omegaA	Peptidases	Mitochondrial__biogenesis	0.0344828
Arabidopsis	omegaA	Photosynthesis__proteins	Amino__acid__related__enzymes	0.2413793
Arabidopsis	omegaA	Photosynthesis__proteins	Chaperones__and__folding__catalysts	0.5862069
Arabidopsis	omegaA	Photosynthesis__proteins	Chromosome__and__associated__proteins	0.9310345

(continued)

species	estimate	var1	var2	p.value
Arabidopsis	omegaA	Photosynthesis__proteins	Cytochrome__P450	0.1724138
Arabidopsis	omegaA	Photosynthesis__proteins	Cytoskeleton__proteins	0.8620690
Arabidopsis	omegaA	Photosynthesis__proteins	DNA__repair__and__recombination__proteins	0.5862069
Arabidopsis	omegaA	Photosynthesis__proteins	DNA__replication__proteins	0.8620690
Arabidopsis	omegaA	Photosynthesis__proteins	Exosome	0.6551724
Arabidopsis	omegaA	Photosynthesis__proteins	G__protein__coupled__receptors	0.0344828
Arabidopsis	omegaA	Photosynthesis__proteins	Glycosyltransferases	0.5862069
Arabidopsis	omegaA	Photosynthesis__proteins	Ion__channels	0.0344828
Arabidopsis	omegaA	Photosynthesis__proteins	Lipid__biosynthesis__proteins	0.1034483
Arabidopsis	omegaA	Photosynthesis__proteins	Membrane__trafficking	0.7931034
Arabidopsis	omegaA	Photosynthesis__proteins	Messenger__RNA__biogenesis	0.7241379
Arabidopsis	omegaA	Photosynthesis__proteins	Mitochondrial__biogenesis	0.2413793
Arabidopsis	omegaA	Photosynthesis__proteins	Peptidases	0.2413793
Arabidopsis	omegaA	Proteasome	Amino__acid__related__enzymes	0.5172414
Arabidopsis	omegaA	Proteasome	Chaperones__and__folding__catalysts	0.6551724
Arabidopsis	omegaA	Proteasome	Chromosome__and__associated__proteins	0.9310345
Arabidopsis	omegaA	Proteasome	Cytochrome__P450	0.3793103
Arabidopsis	omegaA	Proteasome	Cytoskeleton__proteins	0.8620690
Arabidopsis	omegaA	Proteasome	DNA__repair__and__recombination__proteins	0.5172414
Arabidopsis	omegaA	Proteasome	DNA__replication__proteins	1.0000000
Arabidopsis	omegaA	Proteasome	Exosome	0.8620690
Arabidopsis	omegaA	Proteasome	G__protein__coupled__receptors	0.1724138
Arabidopsis	omegaA	Proteasome	Glycosyltransferases	0.7241379
Arabidopsis	omegaA	Proteasome	Ion__channels	0.3793103
Arabidopsis	omegaA	Proteasome	Lipid__biosynthesis__proteins	0.2413793
Arabidopsis	omegaA	Proteasome	Membrane__trafficking	0.7931034
Arabidopsis	omegaA	Proteasome	Messenger__RNA__biogenesis	0.9310345
Arabidopsis	omegaA	Proteasome	Mitochondrial__biogenesis	0.5172414
Arabidopsis	omegaA	Proteasome	Peptidases	0.5172414
Arabidopsis	omegaA	Proteasome	Photosynthesis__proteins	0.9310345
Arabidopsis	omegaA	Protein__kinases	Amino__acid__related__enzymes	0.3793103
Arabidopsis	omegaA	Protein__kinases	Chaperones__and__folding__catalysts	0.3793103
Arabidopsis	omegaA	Protein__kinases	Chromosome__and__associated__proteins	0.9310345
Arabidopsis	omegaA	Protein__kinases	Cytochrome__P450	0.1034483
Arabidopsis	omegaA	Protein__kinases	Cytoskeleton__proteins	0.8620690
Arabidopsis	omegaA	Protein__kinases	DNA__repair__and__recombination__proteins	0.5172414
Arabidopsis	omegaA	Protein__kinases	DNA__replication__proteins	0.9310345
Arabidopsis	omegaA	Protein__kinases	Exosome	0.6551724
Arabidopsis	omegaA	Protein__kinases	G__protein__coupled__receptors	0.0344828
Arabidopsis	omegaA	Protein__kinases	Glycosyltransferases	0.7241379
Arabidopsis	omegaA	Protein__kinases	Ion__channels	0.1034483
Arabidopsis	omegaA	Protein__kinases	Lipid__biosynthesis__proteins	0.0344828
Arabidopsis	omegaA	Protein__kinases	Membrane__trafficking	0.7931034
Arabidopsis	omegaA	Protein__kinases	Messenger__RNA__biogenesis	0.7931034
Arabidopsis	omegaA	Protein__kinases	Mitochondrial__biogenesis	0.3103448
Arabidopsis	omegaA	Protein__kinases	Peptidases	0.1034483
Arabidopsis	omegaA	Protein__kinases	Photosynthesis__proteins	1.0000000
Arabidopsis	omegaA	Protein__kinases	Proteasome	1.0000000
Arabidopsis	omegaA	Protein__phosphatases__and__associated__proteins	Amino__acid__related__enzymes	0.0344828
Arabidopsis	omegaA	Protein__phosphatases__and__associated__proteins	Chaperones__and__folding__catalysts	0.5862069
Arabidopsis	omegaA	Protein__phosphatases__and__associated__proteins	Chromosome__and__associated__proteins	0.1724138
Arabidopsis	omegaA	Protein__phosphatases__and__associated__proteins	Cytochrome__P450	0.8620690
Arabidopsis	omegaA	Protein__phosphatases__and__associated__proteins	Cytoskeleton__proteins	0.5862069
Arabidopsis	omegaA	Protein__phosphatases__and__associated__proteins	DNA__repair__and__recombination__proteins	0.1724138
Arabidopsis	omegaA	Protein__phosphatases__and__associated__proteins	DNA__replication__proteins	0.2413793
Arabidopsis	omegaA	Protein__phosphatases__and__associated__proteins	Exosome	0.1724138
Arabidopsis	omegaA	Protein__phosphatases__and__associated__proteins	G__protein__coupled__receptors	0.8620690
Arabidopsis	omegaA	Protein__phosphatases__and__associated__proteins	Glycosyltransferases	0.3103448
Arabidopsis	omegaA	Protein__phosphatases__and__associated__proteins	Ion__channels	0.7931034
Arabidopsis	omegaA	Protein__phosphatases__and__associated__proteins	Lipid__biosynthesis__proteins	0.7931034
Arabidopsis	omegaA	Protein__phosphatases__and__associated__proteins	Membrane__trafficking	0.3103448

(continued)

species	estimate	var1	var2	p.value
Arabidopsis	omegaA	Protein__phosphatases__and__associated__proteins	Messenger__RNA__biogenesis	0.4482759
Arabidopsis	omegaA	Protein__phosphatases__and__associated__proteins	Mitochondrial__biogenesis	0.0344828
Arabidopsis	omegaA	Protein__phosphatases__and__associated__proteins	Peptidases	0.4482759
Arabidopsis	omegaA	Protein__phosphatases__and__associated__proteins	Photosynthesis__proteins	0.1724138
Arabidopsis	omegaA	Protein__phosphatases__and__associated__proteins	Proteasome	0.2413793
Arabidopsis	omegaA	Protein__phosphatases__and__associated__proteins	Protein__kinases	0.1724138
Arabidopsis	omegaA	Ribosome	Amino__acid__related__enzymes	0.3103448
Arabidopsis	omegaA	Ribosome	Chaperones__and__folding__catalysts	0.5862069
Arabidopsis	omegaA	Ribosome	Chromosome__and__associated__proteins	0.9310345
Arabidopsis	omegaA	Ribosome	Cytochrome__P450	0.1724138
Arabidopsis	omegaA	Ribosome	Cytoskeleton__proteins	0.9310345
Arabidopsis	omegaA	Ribosome	DNA__repair__and__recombination__proteins	0.3793103
Arabidopsis	omegaA	Ribosome	DNA__replication__proteins	1.0000000
Arabidopsis	omegaA	Ribosome	Exosome	0.7931034
Arabidopsis	omegaA	Ribosome	G__protein__coupled__receptors	0.0344828
Arabidopsis	omegaA	Ribosome	Glycosyltransferases	0.5172414
Arabidopsis	omegaA	Ribosome	Ion__channels	0.1724138
Arabidopsis	omegaA	Ribosome	Lipid__biosynthesis__proteins	0.1034483
Arabidopsis	omegaA	Ribosome	Membrane__trafficking	0.7241379
Arabidopsis	omegaA	Ribosome	Messenger__RNA__biogenesis	0.9310345
Arabidopsis	omegaA	Ribosome	Mitochondrial__biogenesis	0.0344828
Arabidopsis	omegaA	Ribosome	Peptidases	0.2413793
Arabidopsis	omegaA	Ribosome	Photosynthesis__proteins	0.7931034
Arabidopsis	omegaA	Ribosome	Proteasome	0.8620690
Arabidopsis	omegaA	Ribosome	Protein__kinases	0.7931034
Arabidopsis	omegaA	Ribosome	Protein__phosphatases__and__associated__proteins	0.2413793
Arabidopsis	omegaA	Ribosome__biogenesis	Amino__acid__related__enzymes	0.0344828
Arabidopsis	omegaA	Ribosome__biogenesis	Chaperones__and__folding__catalysts	0.1034483
Arabidopsis	omegaA	Ribosome__biogenesis	Chromosome__and__associated__proteins	0.0344828
Arabidopsis	omegaA	Ribosome__biogenesis	Cytochrome__P450	0.5862069
Arabidopsis	omegaA	Ribosome__biogenesis	Cytoskeleton__proteins	0.1724138
Arabidopsis	omegaA	Ribosome__biogenesis	DNA__repair__and__recombination__proteins	0.0344828
Arabidopsis	omegaA	Ribosome__biogenesis	DNA__replication__proteins	0.1034483
Arabidopsis	omegaA	Ribosome__biogenesis	Exosome	0.0344828
Arabidopsis	omegaA	Ribosome__biogenesis	G__protein__coupled__receptors	0.5172414
Arabidopsis	omegaA	Ribosome__biogenesis	Glycosyltransferases	0.1034483
Arabidopsis	omegaA	Ribosome__biogenesis	Ion__channels	0.1724138
Arabidopsis	omegaA	Ribosome__biogenesis	Lipid__biosynthesis__proteins	0.5172414
Arabidopsis	omegaA	Ribosome__biogenesis	Membrane__trafficking	0.1034483
Arabidopsis	omegaA	Ribosome__biogenesis	Messenger__RNA__biogenesis	0.1034483
Arabidopsis	omegaA	Ribosome__biogenesis	Mitochondrial__biogenesis	0.0344828
Arabidopsis	omegaA	Ribosome__biogenesis	Peptidases	0.1034483
Arabidopsis	omegaA	Ribosome__biogenesis	Photosynthesis__proteins	0.0344828
Arabidopsis	omegaA	Ribosome__biogenesis	Proteasome	0.0344828
Arabidopsis	omegaA	Ribosome__biogenesis	Protein__kinases	0.0344828
Arabidopsis	omegaA	Ribosome__biogenesis	Protein__phosphatases__and__associated__proteins	0.5862069
Arabidopsis	omegaA	Ribosome__biogenesis	Ribosome	0.0344828
Arabidopsis	omegaA	Spliceosome	Amino__acid__related__enzymes	0.1724138
Arabidopsis	omegaA	Spliceosome	Chaperones__and__folding__catalysts	0.8620690
Arabidopsis	omegaA	Spliceosome	Chromosome__and__associated__proteins	0.9310345
Arabidopsis	omegaA	Spliceosome	Cytochrome__P450	0.3103448
Arabidopsis	omegaA	Spliceosome	Cytoskeleton__proteins	0.9310345
Arabidopsis	omegaA	Spliceosome	DNA__repair__and__recombination__proteins	0.3793103
Arabidopsis	omegaA	Spliceosome	DNA__replication__proteins	0.7241379
Arabidopsis	omegaA	Spliceosome	Exosome	0.3793103
Arabidopsis	omegaA	Spliceosome	G__protein__coupled__receptors	0.1034483
Arabidopsis	omegaA	Spliceosome	Glycosyltransferases	0.9310345
Arabidopsis	omegaA	Spliceosome	Ion__channels	0.2413793
Arabidopsis	omegaA	Spliceosome	Lipid__biosynthesis__proteins	0.1034483
Arabidopsis	omegaA	Spliceosome	Membrane__trafficking	0.9310345
Arabidopsis	omegaA	Spliceosome	Messenger__RNA__biogenesis	0.8620690
Arabidopsis	omegaA	Spliceosome	Mitochondrial__biogenesis	0.2413793

(continued)

species	estimate	var1	var2	p.value
Arabidopsis	omegaA	Spliceosome	Peptidases	0.5862069
Arabidopsis	omegaA	Spliceosome	Photosynthesis__proteins	0.5172414
Arabidopsis	omegaA	Spliceosome	Proteasome	0.7241379
Arabidopsis	omegaA	Spliceosome	Protein__kinases	0.5862069
Arabidopsis	omegaA	Spliceosome	Protein__phosphatases__and__associated__proteins	0.1724138
Arabidopsis	omegaA	Spliceosome	Ribosome	0.4482759
Arabidopsis	omegaA	Spliceosome	Ribosome__biogenesis	0.1034483
Arabidopsis	omegaA	Transcription__factors	Amino__acid__related__enzymes	0.5862069
Arabidopsis	omegaA	Transcription__factors	Chaperones__and__folding__catalysts	0.5172414
Arabidopsis	omegaA	Transcription__factors	Chromosome__and__associated__proteins	0.7931034
Arabidopsis	omegaA	Transcription__factors	Cytochrome__P450	0.1724138
Arabidopsis	omegaA	Transcription__factors	Cytoskeleton__proteins	0.7241379
Arabidopsis	omegaA	Transcription__factors	DNA__repair__and__recombination__proteins	0.7931034
Arabidopsis	omegaA	Transcription__factors	DNA__replication__proteins	0.7241379
Arabidopsis	omegaA	Transcription__factors	Exosome	0.9310345
Arabidopsis	omegaA	Transcription__factors	G__protein__coupled__receptors	0.0344828
Arabidopsis	omegaA	Transcription__factors	Glycosyltransferases	0.6551724
Arabidopsis	omegaA	Transcription__factors	Ion__channels	0.1034483
Arabidopsis	omegaA	Transcription__factors	Lipid__biosynthesis__proteins	0.0344828
Arabidopsis	omegaA	Transcription__factors	Membrane__trafficking	0.5172414
Arabidopsis	omegaA	Transcription__factors	Messenger__RNA__biogenesis	0.5862069
Arabidopsis	omegaA	Transcription__factors	Mitochondrial__biogenesis	0.5862069
Arabidopsis	omegaA	Transcription__factors	Peptidases	0.3103448
Arabidopsis	omegaA	Transcription__factors	Photosynthesis__proteins	0.7931034
Arabidopsis	omegaA	Transcription__factors	Proteasome	0.7241379
Arabidopsis	omegaA	Transcription__factors	Protein__kinases	0.6551724
Arabidopsis	omegaA	Transcription__factors	Protein__phosphatases__and__associated__proteins	0.1724138
Arabidopsis	omegaA	Transcription__factors	Ribosome	0.7931034
Arabidopsis	omegaA	Transcription__factors	Ribosome__biogenesis	0.1034483
Arabidopsis	omegaA	Transcription__factors	Spliceosome	0.5172414
Arabidopsis	omegaA	Transcription__machinery	Amino__acid__related__enzymes	0.0344828
Arabidopsis	omegaA	Transcription__machinery	Chaperones__and__folding__catalysts	0.1034483
Arabidopsis	omegaA	Transcription__machinery	Chromosome__and__associated__proteins	0.1034483
Arabidopsis	omegaA	Transcription__machinery	Cytochrome__P450	0.5862069
Arabidopsis	omegaA	Transcription__machinery	Cytoskeleton__proteins	0.2413793
Arabidopsis	omegaA	Transcription__machinery	DNA__repair__and__recombination__proteins	0.0344828
Arabidopsis	omegaA	Transcription__machinery	DNA__replication__proteins	0.0344828
Arabidopsis	omegaA	Transcription__machinery	Exosome	0.1034483
Arabidopsis	omegaA	Transcription__machinery	G__protein__coupled__receptors	0.5862069
Arabidopsis	omegaA	Transcription__machinery	Glycosyltransferases	0.1724138
Arabidopsis	omegaA	Transcription__machinery	Ion__channels	0.3103448
Arabidopsis	omegaA	Transcription__machinery	Lipid__biosynthesis__proteins	0.4482759
Arabidopsis	omegaA	Transcription__machinery	Membrane__trafficking	0.1034483
Arabidopsis	omegaA	Transcription__machinery	Messenger__RNA__biogenesis	0.1034483
Arabidopsis	omegaA	Transcription__machinery	Mitochondrial__biogenesis	0.0344828
Arabidopsis	omegaA	Transcription__machinery	Peptidases	0.2413793
Arabidopsis	omegaA	Transcription__machinery	Photosynthesis__proteins	0.1034483
Arabidopsis	omegaA	Transcription__machinery	Proteasome	0.1724138
Arabidopsis	omegaA	Transcription__machinery	Protein__kinases	0.1034483
Arabidopsis	omegaA	Transcription__machinery	Protein__phosphatases__and__associated__proteins	0.7241379
Arabidopsis	omegaA	Transcription__machinery	Ribosome	0.1034483
Arabidopsis	omegaA	Transcription__machinery	Ribosome__biogenesis	0.9310345
Arabidopsis	omegaA	Transcription__machinery	Spliceosome	0.1034483
Arabidopsis	omegaA	Transcription__machinery	Transcription__factors	0.0344828
Arabidopsis	omegaA	Transfer__RNA__biogenesis	Amino__acid__related__enzymes	0.6551724
Arabidopsis	omegaA	Transfer__RNA__biogenesis	Chaperones__and__folding__catalysts	0.4482759
Arabidopsis	omegaA	Transfer__RNA__biogenesis	Chromosome__and__associated__proteins	0.7931034
Arabidopsis	omegaA	Transfer__RNA__biogenesis	Cytochrome__P450	0.1724138
Arabidopsis	omegaA	Transfer__RNA__biogenesis	Cytoskeleton__proteins	0.7931034
Arabidopsis	omegaA	Transfer__RNA__biogenesis	DNA__repair__and__recombination__proteins	0.7931034
Arabidopsis	omegaA	Transfer__RNA__biogenesis	DNA__replication__proteins	0.6551724
Arabidopsis	omegaA	Transfer__RNA__biogenesis	Exosome	0.7931034

(continued)

species	estimate	var1	var2	p.value
Arabidopsis	omegaA	Transfer__RNA__biogenesis	G__protein__coupled__receptors	0.0344828
Arabidopsis	omegaA	Transfer__RNA__biogenesis	Glycosyltransferases	0.2413793
Arabidopsis	omegaA	Transfer__RNA__biogenesis	Ion__channels	0.0344828
Arabidopsis	omegaA	Transfer__RNA__biogenesis	Lipid__biosynthesis__proteins	0.1034483
Arabidopsis	omegaA	Transfer__RNA__biogenesis	Membrane__trafficking	0.5172414
Arabidopsis	omegaA	Transfer__RNA__biogenesis	Messenger__RNA__biogenesis	0.5172414
Arabidopsis	omegaA	Transfer__RNA__biogenesis	Mitochondrial__biogenesis	0.7241379
Arabidopsis	omegaA	Transfer__RNA__biogenesis	Peptidases	0.1724138
Arabidopsis	omegaA	Transfer__RNA__biogenesis	Photosynthesis__proteins	0.6551724
Arabidopsis	omegaA	Transfer__RNA__biogenesis	Proteasome	0.8620690
Arabidopsis	omegaA	Transfer__RNA__biogenesis	Protein__kinases	0.8620690
Arabidopsis	omegaA	Transfer__RNA__biogenesis	Protein__phosphatases__and__associated__proteins	0.1724138
Arabidopsis	omegaA	Transfer__RNA__biogenesis	Ribosome	0.7931034
Arabidopsis	omegaA	Transfer__RNA__biogenesis	Ribosome__biogenesis	0.0344828
Arabidopsis	omegaA	Transfer__RNA__biogenesis	Spliceosome	0.5862069
Arabidopsis	omegaA	Transfer__RNA__biogenesis	Transcription__factors	0.7931034
Arabidopsis	omegaA	Transfer__RNA__biogenesis	Transcription__machinery	0.1034483
Arabidopsis	omegaA	Translation__factors	Amino__acid__related__enzymes	0.1034483
Arabidopsis	omegaA	Translation__factors	Chaperones__and__folding__catalysts	0.3793103
Arabidopsis	omegaA	Translation__factors	Chromosome__and__associated__proteins	0.1724138
Arabidopsis	omegaA	Translation__factors	Cytochrome__P450	0.7931034
Arabidopsis	omegaA	Translation__factors	Cytoskeleton__proteins	0.6551724
Arabidopsis	omegaA	Translation__factors	DNA__repair__and__recombination__proteins	0.1034483
Arabidopsis	omegaA	Translation__factors	DNA__replication__proteins	0.2413793
Arabidopsis	omegaA	Translation__factors	Exosome	0.1034483
Arabidopsis	omegaA	Translation__factors	G__protein__coupled__receptors	0.7931034
Arabidopsis	omegaA	Translation__factors	Glycosyltransferases	0.2413793
Arabidopsis	omegaA	Translation__factors	Ion__channels	0.8620690
Arabidopsis	omegaA	Translation__factors	Lipid__biosynthesis__proteins	0.8620690
Arabidopsis	omegaA	Translation__factors	Membrane__trafficking	0.4482759
Arabidopsis	omegaA	Translation__factors	Messenger__RNA__biogenesis	0.5862069
Arabidopsis	omegaA	Translation__factors	Mitochondrial__biogenesis	0.0344828
Arabidopsis	omegaA	Translation__factors	Peptidases	0.5862069
Arabidopsis	omegaA	Translation__factors	Photosynthesis__proteins	0.2413793
Arabidopsis	omegaA	Translation__factors	Proteasome	0.3793103
Arabidopsis	omegaA	Translation__factors	Protein__kinases	0.1724138
Arabidopsis	omegaA	Translation__factors	Protein__phosphatases__and__associated__proteins	0.7931034
Arabidopsis	omegaA	Translation__factors	Ribosome	0.1034483
Arabidopsis	omegaA	Translation__factors	Ribosome__biogenesis	0.5172414
Arabidopsis	omegaA	Translation__factors	Spliceosome	0.5172414
Arabidopsis	omegaA	Translation__factors	Transcription__factors	0.1724138
Arabidopsis	omegaA	Translation__factors	Transcription__machinery	0.3793103
Arabidopsis	omegaA	Translation__factors	Transfer__RNA__biogenesis	0.1034483
Arabidopsis	omegaA	Transporters	Amino__acid__related__enzymes	0.1034483
Arabidopsis	omegaA	Transporters	Chaperones__and__folding__catalysts	0.8620690
Arabidopsis	omegaA	Transporters	Chromosome__and__associated__proteins	0.4482759
Arabidopsis	omegaA	Transporters	Cytochrome__P450	0.3103448
Arabidopsis	omegaA	Transporters	Cytoskeleton__proteins	0.8620690
Arabidopsis	omegaA	Transporters	DNA__repair__and__recombination__proteins	0.1034483
Arabidopsis	omegaA	Transporters	DNA__replication__proteins	0.5172414
Arabidopsis	omegaA	Transporters	Exosome	0.1724138
Arabidopsis	omegaA	Transporters	G__protein__coupled__receptors	0.1724138
Arabidopsis	omegaA	Transporters	Glycosyltransferases	0.7241379
Arabidopsis	omegaA	Transporters	Ion__channels	0.2413793
Arabidopsis	omegaA	Transporters	Lipid__biosynthesis__proteins	0.2413793
Arabidopsis	omegaA	Transporters	Membrane__trafficking	0.4482759
Arabidopsis	omegaA	Transporters	Messenger__RNA__biogenesis	0.7931034
Arabidopsis	omegaA	Transporters	Mitochondrial__biogenesis	0.0344828
Arabidopsis	omegaA	Transporters	Peptidases	0.8620690
Arabidopsis	omegaA	Transporters	Photosynthesis__proteins	0.3793103
Arabidopsis	omegaA	Transporters	Proteasome	0.8620690

(continued)

species	estimate	var1	var2	p.value
Arabidopsis	omegaA	Transporters	Protein__kinases	0.3103448
Arabidopsis	omegaA	Transporters	Protein__phosphatases__and__associated__proteins	0.3103448
Arabidopsis	omegaA	Transporters	Ribosome	0.4482759
Arabidopsis	omegaA	Transporters	Ribosome__biogenesis	0.1034483
Arabidopsis	omegaA	Transporters	Spliceosome	0.7931034
Arabidopsis	omegaA	Transporters	Transcription__factors	0.3793103
Arabidopsis	omegaA	Transporters	Transcription__machinery	0.1034483
Arabidopsis	omegaA	Transporters	Transfer__RNA__biogenesis	0.1724138
Arabidopsis	omegaA	Transporters	Translation__factors	0.4482759
Arabidopsis	omegaA	Ubiquitin__system	Amino__acid__related__enzymes	0.0344828
Arabidopsis	omegaA	Ubiquitin__system	Chaperones__and__folding__catalysts	0.1724138
Arabidopsis	omegaA	Ubiquitin__system	Chromosome__and__associated__proteins	0.0344828
Arabidopsis	omegaA	Ubiquitin__system	Cytochrome__P450	0.7931034
Arabidopsis	omegaA	Ubiquitin__system	Cytoskeleton__proteins	0.3103448
Arabidopsis	omegaA	Ubiquitin__system	DNA__repair__and__recombination__proteins	0.0344828
Arabidopsis	omegaA	Ubiquitin__system	DNA__replication__proteins	0.1034483
Arabidopsis	omegaA	Ubiquitin__system	Exosome	0.0344828
Arabidopsis	omegaA	Ubiquitin__system	G__protein__coupled__receptors	0.6551724
Arabidopsis	omegaA	Ubiquitin__system	Glycosyltransferases	0.0344828
Arabidopsis	omegaA	Ubiquitin__system	Ion__channels	0.3103448
Arabidopsis	omegaA	Ubiquitin__system	Lipid__biosynthesis__proteins	0.5172414
Arabidopsis	omegaA	Ubiquitin__system	Membrane__trafficking	0.1034483
Arabidopsis	omegaA	Ubiquitin__system	Messenger__RNA__biogenesis	0.1034483
Arabidopsis	omegaA	Ubiquitin__system	Mitochondrial__biogenesis	0.0344828
Arabidopsis	omegaA	Ubiquitin__system	Peptidases	0.1724138
Arabidopsis	omegaA	Ubiquitin__system	Photosynthesis__proteins	0.0344828
Arabidopsis	omegaA	Ubiquitin__system	Proteasome	0.1034483
Arabidopsis	omegaA	Ubiquitin__system	Protein__kinases	0.0344828
Arabidopsis	omegaA	Ubiquitin__system	Protein__phosphatases__and__associated__proteins	0.5862069
Arabidopsis	omegaA	Ubiquitin__system	Ribosome	0.0344828
Arabidopsis	omegaA	Ubiquitin__system	Ribosome__biogenesis	1.0000000
Arabidopsis	omegaA	Ubiquitin__system	Spliceosome	0.1034483
Arabidopsis	omegaA	Ubiquitin__system	Transcription__factors	0.0344828
Arabidopsis	omegaA	Ubiquitin__system	Transcription__machinery	0.7241379
Arabidopsis	omegaA	Ubiquitin__system	Transfer__RNA__biogenesis	0.1034483
Arabidopsis	omegaA	Ubiquitin__system	Translation__factors	0.3103448
Arabidopsis	omegaA	Ubiquitin__system	Transporters	0.1034483

```
## Drosophila
dmel.hist <- subset(kegg.hist, kegg.hist$species == "Drosophila")
nboots <- 100
dmel.smallkegg.p <- list()
for (i in 1:nboots) {
  dmel.smallkegg.p[[i]] <- ddply(dmel.hist, c("species", "estimate",
                                              "var1", "var2"),
                                 function(x, N=53){
    c <- as.numeric(nrow(x[x$value < 0,]))
    c2 <- as.numeric(nrow(x[x$value > 0,]))
    m <- min(c, c2)
    p <- (2*m+1)/(N+1)
    tbl <- data.frame(m, p)
  }
)}

# correcting the p-value for multiple testing
dmel.kegg.p.adj <- lapply(dmel.smallkegg.p, function(x) {
  ddply(x,  c("species","estimate", "var1", "var2"), function(x) {
```

```
    p.value <- p.adjust(x$p)
    data.frame(p.value)
  })
})

# taking the minimum p-value of the replicates performed
tbl.dmel.p.adj <- rbindlist(dmel.kegg.p.adj)
dmel.pvalue <- ddply(tbl.dmel.p.adj, c("species","estimate", "var1", "var2"),
                     function(x) {
  p.value <- min(x$p.value)
  data.frame(p.value)
})

# showing the table
kable(dmel.pvalue, format = "latex", booktabs = TRUE, longtable = TRUE) %>%
  kable_styling(latex_options = c("hold_position", "repeat_header"),
                font_size = 7)
```

species	estimate	var1	var2	p.value
Drosophila	dnds	Chaperones_and_folding_catalysts	Amino_acid_related_enzymes	0.4629630
Drosophila	dnds	Chromosome_and_associated_proteins	Amino_acid_related_enzymes	0.1296296
Drosophila	dnds	Chromosome_and_associated_proteins	Chaperones_and_folding_catalysts	0.1666667
Drosophila	dnds	Cytochrome_P450	Amino_acid_related_enzymes	0.7592593
Drosophila	dnds	Cytochrome_P450	Chaperones_and_folding_catalysts	0.9444444
Drosophila	dnds	Cytochrome_P450	Chromosome_and_associated_proteins	0.6111111
Drosophila	dnds	Cytoskeleton_proteins	Amino_acid_related_enzymes	0.7592593
Drosophila	dnds	Cytoskeleton_proteins	Chaperones_and_folding_catalysts	0.3518519
Drosophila	dnds	Cytoskeleton_proteins	Chromosome_and_associated_proteins	0.0185185
Drosophila	dnds	Cytoskeleton_proteins	Cytochrome_P450	0.6111111
Drosophila	dnds	DNA_repair_and_recombination_proteins	Amino_acid_related_enzymes	0.0555556
Drosophila	dnds	DNA_repair_and_recombination_proteins	Chaperones_and_folding_catalysts	0.0185185
Drosophila	dnds	DNA_repair_and_recombination_proteins	Chromosome_and_associated_proteins	0.0555556
Drosophila	dnds	DNA_repair_and_recombination_proteins	Cytochrome_P450	0.1666667
Drosophila	dnds	DNA_repair_and_recombination_proteins	Cytoskeleton_proteins	0.0185185
Drosophila	dnds	DNA_replication_proteins	Amino_acid_related_enzymes	0.4259259
Drosophila	dnds	DNA_replication_proteins	Chaperones_and_folding_catalysts	0.2037037
Drosophila	dnds	DNA_replication_proteins	Chromosome_and_associated_proteins	0.0185185
Drosophila	dnds	DNA_replication_proteins	Cytochrome_P450	0.6111111
Drosophila	dnds	DNA_replication_proteins	Cytoskeleton_proteins	0.9074074
Drosophila	dnds	DNA_replication_proteins	DNA_repair_and_recombination_proteins	0.0185185
Drosophila	dnds	Exosome	Amino_acid_related_enzymes	0.5000000
Drosophila	dnds	Exosome	Chaperones_and_folding_catalysts	0.2037037
Drosophila	dnds	Exosome	Chromosome_and_associated_proteins	0.0185185
Drosophila	dnds	Exosome	Cytochrome_P450	0.5370370
Drosophila	dnds	Exosome	Cytoskeleton_proteins	0.9074074
Drosophila	dnds	Exosome	DNA_repair_and_recombination_proteins	0.0185185
Drosophila	dnds	Exosome	DNA_replication_proteins	0.8703704
Drosophila	dnds	G_protein_coupled_receptors	Amino_acid_related_enzymes	0.0185185
Drosophila	dnds	G_protein_coupled_receptors	Chaperones_and_folding_catalysts	0.0185185
Drosophila	dnds	G_protein_coupled_receptors	Chromosome_and_associated_proteins	0.0185185
Drosophila	dnds	G_protein_coupled_receptors	Cytochrome_P450	0.0925926
Drosophila	dnds	G_protein_coupled_receptors	Cytoskeleton_proteins	0.1666667
Drosophila	dnds	G_protein_coupled_receptors	DNA_repair_and_recombination_proteins	0.0185185
Drosophila	dnds	G_protein_coupled_receptors	DNA_replication_proteins	0.0555556
Drosophila	dnds	G_protein_coupled_receptors	Exosome	0.0925926
Drosophila	dnds	Glycosyltransferases	Amino_acid_related_enzymes	0.7962963
Drosophila	dnds	Glycosyltransferases	Chaperones_and_folding_catalysts	0.4629630
Drosophila	dnds	Glycosyltransferases	Chromosome_and_associated_proteins	0.0185185
Drosophila	dnds	Glycosyltransferases	Cytochrome_P450	0.7592593
Drosophila	dnds	Glycosyltransferases	Cytoskeleton_proteins	0.6111111

(continued)

species	estimate	var1	var2	p.value
Drosophila	dnds	Glycosyltransferases	DNA__repair__and__recombination__proteins	0.0185185
Drosophila	dnds	Glycosyltransferases	DNA__replication__proteins	0.2037037
Drosophila	dnds	Glycosyltransferases	Exosome	0.2037037
Drosophila	dnds	Glycosyltransferases	G__protein__coupled__receptors	0.0555556
Drosophila	dnds	Ion__channels	Amino__acid__related__enzymes	0.0185185
Drosophila	dnds	Ion__channels	Chaperones__and__folding__catalysts	0.0185185
Drosophila	dnds	Ion__channels	Chromosome__and__associated__proteins	0.0185185
Drosophila	dnds	Ion__channels	Cytochrome__P450	0.0185185
Drosophila	dnds	Ion__channels	Cytoskeleton__proteins	0.0185185
Drosophila	dnds	Ion__channels	DNA__repair__and__recombination__proteins	0.0185185
Drosophila	dnds	Ion__channels	DNA__replication__proteins	0.0185185
Drosophila	dnds	Ion__channels	Exosome	0.0185185
Drosophila	dnds	Ion__channels	G__protein__coupled__receptors	0.1296296
Drosophila	dnds	Ion__channels	Glycosyltransferases	0.0185185
Drosophila	dnds	Lipid__biosynthesis__proteins	Amino__acid__related__enzymes	0.7962963
Drosophila	dnds	Lipid__biosynthesis__proteins	Chaperones__and__folding__catalysts	0.7222222
Drosophila	dnds	Lipid__biosynthesis__proteins	Chromosome__and__associated__proteins	0.2037037
Drosophila	dnds	Lipid__biosynthesis__proteins	Cytochrome__P450	0.6111111
Drosophila	dnds	Lipid__biosynthesis__proteins	Cytoskeleton__proteins	0.7592593
Drosophila	dnds	Lipid__biosynthesis__proteins	DNA__repair__and__recombination__proteins	0.0185185
Drosophila	dnds	Lipid__biosynthesis__proteins	DNA__replication__proteins	0.7222222
Drosophila	dnds	Lipid__biosynthesis__proteins	Exosome	0.6111111
Drosophila	dnds	Lipid__biosynthesis__proteins	G__protein__coupled__receptors	0.1296296
Drosophila	dnds	Lipid__biosynthesis__proteins	Glycosyltransferases	0.9074074
Drosophila	dnds	Lipid__biosynthesis__proteins	Ion__channels	0.0185185
Drosophila	dnds	Membrane__trafficking	Amino__acid__related__enzymes	0.0185185
Drosophila	dnds	Membrane__trafficking	Chaperones__and__folding__catalysts	0.0185185
Drosophila	dnds	Membrane__trafficking	Chromosome__and__associated__proteins	0.0185185
Drosophila	dnds	Membrane__trafficking	Cytochrome__P450	0.2407407
Drosophila	dnds	Membrane__trafficking	Cytoskeleton__proteins	0.1296296
Drosophila	dnds	Membrane__trafficking	DNA__repair__and__recombination__proteins	0.0185185
Drosophila	dnds	Membrane__trafficking	DNA__replication__proteins	0.0185185
Drosophila	dnds	Membrane__trafficking	Exosome	0.0185185
Drosophila	dnds	Membrane__trafficking	G__protein__coupled__receptors	0.2037037
Drosophila	dnds	Membrane__trafficking	Glycosyltransferases	0.0185185
Drosophila	dnds	Membrane__trafficking	Ion__channels	0.0185185
Drosophila	dnds	Membrane__trafficking	Lipid__biosynthesis__proteins	0.2777778
Drosophila	dnds	Messenger__RNA__biogenesis	Amino__acid__related__enzymes	0.0555556
Drosophila	dnds	Messenger__RNA__biogenesis	Chaperones__and__folding__catalysts	0.0185185
Drosophila	dnds	Messenger__RNA__biogenesis	Chromosome__and__associated__proteins	0.0185185
Drosophila	dnds	Messenger__RNA__biogenesis	Cytochrome__P450	0.0185185
Drosophila	dnds	Messenger__RNA__biogenesis	Cytoskeleton__proteins	0.0185185
Drosophila	dnds	Messenger__RNA__biogenesis	DNA__repair__and__recombination__proteins	0.3888889
Drosophila	dnds	Messenger__RNA__biogenesis	DNA__replication__proteins	0.0185185
Drosophila	dnds	Messenger__RNA__biogenesis	Exosome	0.0185185
Drosophila	dnds	Messenger__RNA__biogenesis	G__protein__coupled__receptors	0.0185185
Drosophila	dnds	Messenger__RNA__biogenesis	Glycosyltransferases	0.0185185
Drosophila	dnds	Messenger__RNA__biogenesis	Ion__channels	0.0185185
Drosophila	dnds	Messenger__RNA__biogenesis	Lipid__biosynthesis__proteins	0.0185185
Drosophila	dnds	Messenger__RNA__biogenesis	Membrane__trafficking	0.0185185
Drosophila	dnds	Mitochondrial__biogenesis	Amino__acid__related__enzymes	0.9814815
Drosophila	dnds	Mitochondrial__biogenesis	Chaperones__and__folding__catalysts	0.2777778
Drosophila	dnds	Mitochondrial__biogenesis	Chromosome__and__associated__proteins	0.0185185
Drosophila	dnds	Mitochondrial__biogenesis	Cytochrome__P450	0.7222222
Drosophila	dnds	Mitochondrial__biogenesis	Cytoskeleton__proteins	0.7222222
Drosophila	dnds	Mitochondrial__biogenesis	DNA__repair__and__recombination__proteins	0.0185185
Drosophila	dnds	Mitochondrial__biogenesis	DNA__replication__proteins	0.3518519
Drosophila	dnds	Mitochondrial__biogenesis	Exosome	0.3148148
Drosophila	dnds	Mitochondrial__biogenesis	G__protein__coupled__receptors	0.0185185
Drosophila	dnds	Mitochondrial__biogenesis	Glycosyltransferases	0.5740741
Drosophila	dnds	Mitochondrial__biogenesis	Ion__channels	0.0185185
Drosophila	dnds	Mitochondrial__biogenesis	Lipid__biosynthesis__proteins	0.8333333

(continued)

species	estimate	var1	var2	p.value
Drosophila	dnds	Mitochondrial__biogenesis	Membrane__trafficking	0.0185185
Drosophila	dnds	Mitochondrial__biogenesis	Messenger__RNA__biogenesis	0.0185185
Drosophila	dnds	Peptidases	Amino__acid__related__enzymes	0.1296296
Drosophila	dnds	Peptidases	Chaperones__and__folding__catalysts	0.2777778
Drosophila	dnds	Peptidases	Chromosome__and__associated__proteins	0.9814815
Drosophila	dnds	Peptidases	Cytochrome__P450	0.6851852
Drosophila	dnds	Peptidases	Cytoskeleton__proteins	0.0555556
Drosophila	dnds	Peptidases	DNA__repair__and__recombination__proteins	0.2037037
Drosophila	dnds	Peptidases	DNA__replication__proteins	0.0185185
Drosophila	dnds	Peptidases	Exosome	0.0185185
Drosophila	dnds	Peptidases	G__protein__coupled__receptors	0.0185185
Drosophila	dnds	Peptidases	Glycosyltransferases	0.0185185
Drosophila	dnds	Peptidases	Ion__channels	0.0185185
Drosophila	dnds	Peptidases	Lipid__biosynthesis__proteins	0.3148148
Drosophila	dnds	Peptidases	Membrane__trafficking	0.0185185
Drosophila	dnds	Peptidases	Messenger__RNA__biogenesis	0.0555556
Drosophila	dnds	Peptidases	Mitochondrial__biogenesis	0.0185185
Drosophila	dnds	Photosynthesis__proteins	Amino__acid__related__enzymes	0.2777778
Drosophila	dnds	Photosynthesis__proteins	Chaperones__and__folding__catalysts	0.1296296
Drosophila	dnds	Photosynthesis__proteins	Chromosome__and__associated__proteins	0.0555556
Drosophila	dnds	Photosynthesis__proteins	Cytochrome__P450	0.2777778
Drosophila	dnds	Photosynthesis__proteins	Cytoskeleton__proteins	0.5740741
Drosophila	dnds	Photosynthesis__proteins	DNA__repair__and__recombination__proteins	0.0185185
Drosophila	dnds	Photosynthesis__proteins	DNA__replication__proteins	0.3888889
Drosophila	dnds	Photosynthesis__proteins	Exosome	0.5740741
Drosophila	dnds	Photosynthesis__proteins	G__protein__coupled__receptors	0.2777778
Drosophila	dnds	Photosynthesis__proteins	Glycosyltransferases	0.1296296
Drosophila	dnds	Photosynthesis__proteins	Ion__channels	0.0185185
Drosophila	dnds	Photosynthesis__proteins	Lipid__biosynthesis__proteins	0.4259259
Drosophila	dnds	Photosynthesis__proteins	Membrane__trafficking	0.4259259
Drosophila	dnds	Photosynthesis__proteins	Messenger__RNA__biogenesis	0.0185185
Drosophila	dnds	Photosynthesis__proteins	Mitochondrial__biogenesis	0.1666667
Drosophila	dnds	Photosynthesis__proteins	Peptidases	0.0555556
Drosophila	dnds	Proteasome	Amino__acid__related__enzymes	0.3148148
Drosophila	dnds	Proteasome	Chaperones__and__folding__catalysts	0.1296296
Drosophila	dnds	Proteasome	Chromosome__and__associated__proteins	0.0185185
Drosophila	dnds	Proteasome	Cytochrome__P450	0.3888889
Drosophila	dnds	Proteasome	Cytoskeleton__proteins	0.7222222
Drosophila	dnds	Proteasome	DNA__repair__and__recombination__proteins	0.0185185
Drosophila	dnds	Proteasome	DNA__replication__proteins	0.6111111
Drosophila	dnds	Proteasome	Exosome	0.6851852
Drosophila	dnds	Proteasome	G__protein__coupled__receptors	0.3888889
Drosophila	dnds	Proteasome	Glycosyltransferases	0.2407407
Drosophila	dnds	Proteasome	Ion__channels	0.0185185
Drosophila	dnds	Proteasome	Lipid__biosynthesis__proteins	0.3518519
Drosophila	dnds	Proteasome	Membrane__trafficking	0.6851852
Drosophila	dnds	Proteasome	Messenger__RNA__biogenesis	0.0185185
Drosophila	dnds	Proteasome	Mitochondrial__biogenesis	0.2777778
Drosophila	dnds	Proteasome	Peptidases	0.0555556
Drosophila	dnds	Proteasome	Photosynthesis__proteins	0.9444444
Drosophila	dnds	Protein__kinases	Amino__acid__related__enzymes	0.1666667
Drosophila	dnds	Protein__kinases	Chaperones__and__folding__catalysts	0.0925926
Drosophila	dnds	Protein__kinases	Chromosome__and__associated__proteins	0.0185185
Drosophila	dnds	Protein__kinases	Cytochrome__P450	0.3888889
Drosophila	dnds	Protein__kinases	Cytoskeleton__proteins	0.5740741
Drosophila	dnds	Protein__kinases	DNA__repair__and__recombination__proteins	0.0185185
Drosophila	dnds	Protein__kinases	DNA__replication__proteins	0.2037037
Drosophila	dnds	Protein__kinases	Exosome	0.3148148
Drosophila	dnds	Protein__kinases	G__protein__coupled__receptors	0.0555556
Drosophila	dnds	Protein__kinases	Glycosyltransferases	0.0185185
Drosophila	dnds	Protein__kinases	Ion__channels	0.0185185
Drosophila	dnds	Protein__kinases	Lipid__biosynthesis__proteins	0.3888889

(continued)

species	estimate	var1	var2	p.value
Drosophila	dnds	Protein__kinases	Membrane__trafficking	0.0555556
Drosophila	dnds	Protein__kinases	Messenger__RNA__biogenesis	0.0185185
Drosophila	dnds	Protein__kinases	Mitochondrial__biogenesis	0.0555556
Drosophila	dnds	Protein__kinases	Peptidases	0.0185185
Drosophila	dnds	Protein__kinases	Photosynthesis__proteins	0.9074074
Drosophila	dnds	Protein__kinases	Proteasome	0.9074074
Drosophila	dnds	Protein__phosphatases__and__associated__proteins	Amino__acid__related__enzymes	0.2407407
Drosophila	dnds	Protein__phosphatases__and__associated__proteins	Chaperones__and__folding__catalysts	0.4629630
Drosophila	dnds	Protein__phosphatases__and__associated__proteins	Chromosome__and__associated__proteins	0.4259259
Drosophila	dnds	Protein__phosphatases__and__associated__proteins	Cytochrome__P450	0.9444444
Drosophila	dnds	Protein__phosphatases__and__associated__proteins	Cytoskeleton__proteins	0.0555556
Drosophila	dnds	Protein__phosphatases__and__associated__proteins	DNA__repair__and__recombination__proteins	0.1296296
Drosophila	dnds	Protein__phosphatases__and__associated__proteins	DNA__replication__proteins	0.0185185
Drosophila	dnds	Protein__phosphatases__and__associated__proteins	Exosome	0.0185185
Drosophila	dnds	Protein__phosphatases__and__associated__proteins	G__protein__coupled__receptors	0.0185185
Drosophila	dnds	Protein__phosphatases__and__associated__proteins	Glycosyltransferases	0.0185185
Drosophila	dnds	Protein__phosphatases__and__associated__proteins	Ion__channels	0.0185185
Drosophila	dnds	Protein__phosphatases__and__associated__proteins	Lipid__biosynthesis__proteins	0.4259259
Drosophila	dnds	Protein__phosphatases__and__associated__proteins	Membrane__trafficking	0.0185185
Drosophila	dnds	Protein__phosphatases__and__associated__proteins	Messenger__RNA__biogenesis	0.0185185
Drosophila	dnds	Protein__phosphatases__and__associated__proteins	Mitochondrial__biogenesis	0.0185185
Drosophila	dnds	Protein__phosphatases__and__associated__proteins	Peptidases	0.5000000
Drosophila	dnds	Protein__phosphatases__and__associated__proteins	Photosynthesis__proteins	0.0555556
Drosophila	dnds	Protein__phosphatases__and__associated__proteins	Proteasome	0.0555556
Drosophila	dnds	Protein__phosphatases__and__associated__proteins	Protein__kinases	0.0185185
Drosophila	dnds	Ribosome	Amino__acid__related__enzymes	0.6481481
Drosophila	dnds	Ribosome	Chaperones__and__folding__catalysts	0.6851852
Drosophila	dnds	Ribosome	Chromosome__and__associated__proteins	0.0185185
Drosophila	dnds	Ribosome	Cytochrome__P450	0.8333333
Drosophila	dnds	Ribosome	Cytoskeleton__proteins	0.3888889
Drosophila	dnds	Ribosome	DNA__repair__and__recombination__proteins	0.0185185
Drosophila	dnds	Ribosome	DNA__replication__proteins	0.0555556
Drosophila	dnds	Ribosome	Exosome	0.0555556
Drosophila	dnds	Ribosome	G__protein__coupled__receptors	0.0555556
Drosophila	dnds	Ribosome	Glycosyltransferases	0.5370370
Drosophila	dnds	Ribosome	Ion__channels	0.0185185
Drosophila	dnds	Ribosome	Lipid__biosynthesis__proteins	0.9444444
Drosophila	dnds	Ribosome	Membrane__trafficking	0.0185185
Drosophila	dnds	Ribosome	Messenger__RNA__biogenesis	0.0185185
Drosophila	dnds	Ribosome	Mitochondrial__biogenesis	0.2407407
Drosophila	dnds	Ribosome	Peptidases	0.0925926
Drosophila	dnds	Ribosome	Photosynthesis__proteins	0.1296296
Drosophila	dnds	Ribosome	Proteasome	0.1296296
Drosophila	dnds	Ribosome	Protein__kinases	0.0185185
Drosophila	dnds	Ribosome	Protein__phosphatases__and__associated__proteins	0.0925926
Drosophila	dnds	Ribosome__biogenesis	Amino__acid__related__enzymes	0.1296296
Drosophila	dnds	Ribosome__biogenesis	Chaperones__and__folding__catalysts	0.1666667
Drosophila	dnds	Ribosome__biogenesis	Chromosome__and__associated__proteins	0.6851852
Drosophila	dnds	Ribosome__biogenesis	Cytochrome__P450	0.7222222
Drosophila	dnds	Ribosome__biogenesis	Cytoskeleton__proteins	0.0185185
Drosophila	dnds	Ribosome__biogenesis	DNA__repair__and__recombination__proteins	0.0925926
Drosophila	dnds	Ribosome__biogenesis	DNA__replication__proteins	0.0185185
Drosophila	dnds	Ribosome__biogenesis	Exosome	0.0185185
Drosophila	dnds	Ribosome__biogenesis	G__protein__coupled__receptors	0.0185185
Drosophila	dnds	Ribosome__biogenesis	Glycosyltransferases	0.0185185
Drosophila	dnds	Ribosome__biogenesis	Ion__channels	0.0185185
Drosophila	dnds	Ribosome__biogenesis	Lipid__biosynthesis__proteins	0.3518519
Drosophila	dnds	Ribosome__biogenesis	Membrane__trafficking	0.0185185
Drosophila	dnds	Ribosome__biogenesis	Messenger__RNA__biogenesis	0.0185185
Drosophila	dnds	Ribosome__biogenesis	Mitochondrial__biogenesis	0.0185185
Drosophila	dnds	Ribosome__biogenesis	Peptidases	0.9074074

(continued)

species	estimate	var1	var2	p.value
Drosophila	dnds	Ribosome__biogenesis	Photosynthesis__proteins	0.0185185
Drosophila	dnds	Ribosome__biogenesis	Proteasome	0.0555556
Drosophila	dnds	Ribosome__biogenesis	Protein__kinases	0.0185185
Drosophila	dnds	Ribosome__biogenesis	Protein__phosphatases__and__associated__proteins	0.7592593
Drosophila	dnds	Ribosome__biogenesis	Ribosome	0.0925926
Drosophila	dnds	Spliceosome	Amino__acid__related__enzymes	0.2407407
Drosophila	dnds	Spliceosome	Chaperones__and__folding__catalysts	0.5740741
Drosophila	dnds	Spliceosome	Chromosome__and__associated__proteins	0.4629630
Drosophila	dnds	Spliceosome	Cytochrome__P450	0.9814815
Drosophila	dnds	Spliceosome	Cytoskeleton__proteins	0.2037037
Drosophila	dnds	Spliceosome	DNA__repair__and__recombination__proteins	0.0555556
Drosophila	dnds	Spliceosome	DNA__replication__proteins	0.0185185
Drosophila	dnds	Spliceosome	Exosome	0.0185185
Drosophila	dnds	Spliceosome	G__protein__coupled__receptors	0.0185185
Drosophila	dnds	Spliceosome	Glycosyltransferases	0.1296296
Drosophila	dnds	Spliceosome	Ion__channels	0.0185185
Drosophila	dnds	Spliceosome	Lipid__biosynthesis__proteins	0.6111111
Drosophila	dnds	Spliceosome	Membrane__trafficking	0.0185185
Drosophila	dnds	Spliceosome	Messenger__RNA__biogenesis	0.0555556
Drosophila	dnds	Spliceosome	Mitochondrial__biogenesis	0.0185185
Drosophila	dnds	Spliceosome	Peptidases	0.5000000
Drosophila	dnds	Spliceosome	Photosynthesis__proteins	0.0555556
Drosophila	dnds	Spliceosome	Proteasome	0.0555556
Drosophila	dnds	Spliceosome	Protein__kinases	0.0185185
Drosophila	dnds	Spliceosome	Protein__phosphatases__and__associated__proteins	0.7962963
Drosophila	dnds	Spliceosome	Ribosome	0.2407407
Drosophila	dnds	Spliceosome	Ribosome__biogenesis	0.4629630
Drosophila	dnds	Transcription__factors	Amino__acid__related__enzymes	0.1666667
Drosophila	dnds	Transcription__factors	Chaperones__and__folding__catalysts	0.5000000
Drosophila	dnds	Transcription__factors	Chromosome__and__associated__proteins	0.1296296
Drosophila	dnds	Transcription__factors	Cytochrome__P450	0.9814815
Drosophila	dnds	Transcription__factors	Cytoskeleton__proteins	0.0555556
Drosophila	dnds	Transcription__factors	DNA__repair__and__recombination__proteins	0.0185185
Drosophila	dnds	Transcription__factors	DNA__replication__proteins	0.0185185
Drosophila	dnds	Transcription__factors	Exosome	0.0185185
Drosophila	dnds	Transcription__factors	G__protein__coupled__receptors	0.0185185
Drosophila	dnds	Transcription__factors	Glycosyltransferases	0.0185185
Drosophila	dnds	Transcription__factors	Ion__channels	0.0185185
Drosophila	dnds	Transcription__factors	Lipid__biosynthesis__proteins	0.5370370
Drosophila	dnds	Transcription__factors	Membrane__trafficking	0.0185185
Drosophila	dnds	Transcription__factors	Messenger__RNA__biogenesis	0.0185185
Drosophila	dnds	Transcription__factors	Mitochondrial__biogenesis	0.0185185
Drosophila	dnds	Transcription__factors	Peptidases	0.3148148
Drosophila	dnds	Transcription__factors	Photosynthesis__proteins	0.0555556
Drosophila	dnds	Transcription__factors	Proteasome	0.0555556
Drosophila	dnds	Transcription__factors	Protein__kinases	0.0185185
Drosophila	dnds	Transcription__factors	Protein__phosphatases__and__associated__proteins	0.8333333
Drosophila	dnds	Transcription__factors	Ribosome	0.1296296
Drosophila	dnds	Transcription__factors	Ribosome__biogenesis	0.3888889
Drosophila	dnds	Transcription__factors	Spliceosome	0.9444444
Drosophila	dnds	Transcription__machinery	Amino__acid__related__enzymes	0.7222222
Drosophila	dnds	Transcription__machinery	Chaperones__and__folding__catalysts	0.3518519
Drosophila	dnds	Transcription__machinery	Chromosome__and__associated__proteins	0.0185185
Drosophila	dnds	Transcription__machinery	Cytochrome__P450	0.6111111
Drosophila	dnds	Transcription__machinery	Cytoskeleton__proteins	0.9074074
Drosophila	dnds	Transcription__machinery	DNA__repair__and__recombination__proteins	0.0185185
Drosophila	dnds	Transcription__machinery	DNA__replication__proteins	0.7222222
Drosophila	dnds	Transcription__machinery	Exosome	0.7962963
Drosophila	dnds	Transcription__machinery	G__protein__coupled__receptors	0.0555556
Drosophila	dnds	Transcription__machinery	Glycosyltransferases	0.5740741
Drosophila	dnds	Transcription__machinery	Ion__channels	0.0185185
Drosophila	dnds	Transcription__machinery	Lipid__biosynthesis__proteins	0.7222222

(continued)

species	estimate	var1	var2	p.value
Drosophila	dnds	Transcription__machinery	Membrane__trafficking	0.0185185
Drosophila	dnds	Transcription__machinery	Messenger__RNA__biogenesis	0.0185185
Drosophila	dnds	Transcription__machinery	Mitochondrial__biogenesis	0.6851852
Drosophila	dnds	Transcription__machinery	Peptidases	0.0555556
Drosophila	dnds	Transcription__machinery	Photosynthesis__proteins	0.3518519
Drosophila	dnds	Transcription__machinery	Proteasome	0.3518519
Drosophila	dnds	Transcription__machinery	Protein__kinases	0.1296296
Drosophila	dnds	Transcription__machinery	Protein__phosphatases__and__associated__proteins	0.0555556
Drosophila	dnds	Transcription__machinery	Ribosome	0.3888889
Drosophila	dnds	Transcription__machinery	Ribosome__biogenesis	0.0185185
Drosophila	dnds	Transcription__machinery	Spliceosome	0.0555556
Drosophila	dnds	Transcription__machinery	Transcription__factors	0.0185185
Drosophila	dnds	Transfer__RNA__biogenesis	Amino__acid__related__enzymes	0.8703704
Drosophila	dnds	Transfer__RNA__biogenesis	Chaperones__and__folding__catalysts	0.5000000
Drosophila	dnds	Transfer__RNA__biogenesis	Chromosome__and__associated__proteins	0.0185185
Drosophila	dnds	Transfer__RNA__biogenesis	Cytochrome__P450	0.7962963
Drosophila	dnds	Transfer__RNA__biogenesis	Cytoskeleton__proteins	0.5370370
Drosophila	dnds	Transfer__RNA__biogenesis	DNA__repair__and__recombination__proteins	0.0185185
Drosophila	dnds	Transfer__RNA__biogenesis	DNA__replication__proteins	0.0925926
Drosophila	dnds	Transfer__RNA__biogenesis	Exosome	0.2037037
Drosophila	dnds	Transfer__RNA__biogenesis	G__protein__coupled__receptors	0.0185185
Drosophila	dnds	Transfer__RNA__biogenesis	Glycosyltransferases	0.9814815
Drosophila	dnds	Transfer__RNA__biogenesis	Ion__channels	0.0185185
Drosophila	dnds	Transfer__RNA__biogenesis	Lipid__biosynthesis__proteins	0.9444444
Drosophila	dnds	Transfer__RNA__biogenesis	Membrane__trafficking	0.0185185
Drosophila	dnds	Transfer__RNA__biogenesis	Messenger__RNA__biogenesis	0.0185185
Drosophila	dnds	Transfer__RNA__biogenesis	Mitochondrial__biogenesis	0.4629630
Drosophila	dnds	Transfer__RNA__biogenesis	Peptidases	0.0185185
Drosophila	dnds	Transfer__RNA__biogenesis	Photosynthesis__proteins	0.1296296
Drosophila	dnds	Transfer__RNA__biogenesis	Proteasome	0.1296296
Drosophila	dnds	Transfer__RNA__biogenesis	Protein__kinases	0.0185185
Drosophila	dnds	Transfer__RNA__biogenesis	Protein__phosphatases__and__associated__proteins	0.0555556
Drosophila	dnds	Transfer__RNA__biogenesis	Ribosome	0.9814815
Drosophila	dnds	Transfer__RNA__biogenesis	Ribosome__biogenesis	0.0185185
Drosophila	dnds	Transfer__RNA__biogenesis	Spliceosome	0.1666667
Drosophila	dnds	Transfer__RNA__biogenesis	Transcription__factors	0.0185185
Drosophila	dnds	Transfer__RNA__biogenesis	Transcription__machinery	0.6851852
Drosophila	dnds	Translation__factors	Amino__acid__related__enzymes	0.4629630
Drosophila	dnds	Translation__factors	Chaperones__and__folding__catalysts	0.7962963
Drosophila	dnds	Translation__factors	Chromosome__and__associated__proteins	0.6481481
Drosophila	dnds	Translation__factors	Cytochrome__P450	0.8333333
Drosophila	dnds	Translation__factors	Cytoskeleton__proteins	0.3888889
Drosophila	dnds	Translation__factors	DNA__repair__and__recombination__proteins	0.0925926
Drosophila	dnds	Translation__factors	DNA__replication__proteins	0.3148148
Drosophila	dnds	Translation__factors	Exosome	0.3148148
Drosophila	dnds	Translation__factors	G__protein__coupled__receptors	0.0185185
Drosophila	dnds	Translation__factors	Glycosyltransferases	0.5740741
Drosophila	dnds	Translation__factors	Ion__channels	0.0185185
Drosophila	dnds	Translation__factors	Lipid__biosynthesis__proteins	0.3148148
Drosophila	dnds	Translation__factors	Membrane__trafficking	0.0555556
Drosophila	dnds	Translation__factors	Messenger__RNA__biogenesis	0.0555556
Drosophila	dnds	Translation__factors	Mitochondrial__biogenesis	0.3888889
Drosophila	dnds	Translation__factors	Peptidases	0.6851852
Drosophila	dnds	Translation__factors	Photosynthesis__proteins	0.1666667
Drosophila	dnds	Translation__factors	Proteasome	0.1296296
Drosophila	dnds	Translation__factors	Protein__kinases	0.0925926
Drosophila	dnds	Translation__factors	Protein__phosphatases__and__associated__proteins	0.8703704
Drosophila	dnds	Translation__factors	Ribosome	0.5370370
Drosophila	dnds	Translation__factors	Ribosome__biogenesis	0.7592593
Drosophila	dnds	Translation__factors	Spliceosome	0.9814815
Drosophila	dnds	Translation__factors	Transcription__factors	0.9814815
Drosophila	dnds	Translation__factors	Transcription__machinery	0.3518519

(continued)

species	estimate	var1	var2	p.value
Drosophila	dnds	Translation__factors	Transfer__RNA__biogenesis	0.5370370
Drosophila	dnds	Transporters	Amino__acid__related__enzymes	0.3888889
Drosophila	dnds	Transporters	Chaperones__and__folding__catalysts	0.2037037
Drosophila	dnds	Transporters	Chromosome__and__associated__proteins	0.0185185
Drosophila	dnds	Transporters	Cytochrome__P450	0.6851852
Drosophila	dnds	Transporters	Cytoskeleton__proteins	0.7592593
Drosophila	dnds	Transporters	DNA__repair__and__recombination__proteins	0.0185185
Drosophila	dnds	Transporters	DNA__replication__proteins	0.8703704
Drosophila	dnds	Transporters	Exosome	0.9814815
Drosophila	dnds	Transporters	G__protein__coupled__receptors	0.0925926
Drosophila	dnds	Transporters	Glycosyltransferases	0.1666667
Drosophila	dnds	Transporters	Ion__channels	0.0185185
Drosophila	dnds	Transporters	Lipid__biosynthesis__proteins	0.7222222
Drosophila	dnds	Transporters	Membrane__trafficking	0.0185185
Drosophila	dnds	Transporters	Messenger__RNA__biogenesis	0.0185185
Drosophila	dnds	Transporters	Mitochondrial__biogenesis	0.3518519
Drosophila	dnds	Transporters	Peptidases	0.0185185
Drosophila	dnds	Transporters	Photosynthesis__proteins	0.5000000
Drosophila	dnds	Transporters	Proteasome	0.7962963
Drosophila	dnds	Transporters	Protein__kinases	0.2407407
Drosophila	dnds	Transporters	Protein__phosphatases__and__associated__proteins	0.0185185
Drosophila	dnds	Transporters	Ribosome	0.0555556
Drosophila	dnds	Transporters	Ribosome__biogenesis	0.0185185
Drosophila	dnds	Transporters	Spliceosome	0.0185185
Drosophila	dnds	Transporters	Transcription__factors	0.0185185
Drosophila	dnds	Transporters	Transcription__machinery	0.6851852
Drosophila	dnds	Transporters	Transfer__RNA__biogenesis	0.0925926
Drosophila	dnds	Transporters	Translation__factors	0.2777778
Drosophila	dnds	Ubiquitin__system	Amino__acid__related__enzymes	0.2407407
Drosophila	dnds	Ubiquitin__system	Chaperones__and__folding__catalysts	0.6481481
Drosophila	dnds	Ubiquitin__system	Chromosome__and__associated__proteins	0.1296296
Drosophila	dnds	Ubiquitin__system	Cytochrome__P450	0.8703704
Drosophila	dnds	Ubiquitin__system	Cytoskeleton__proteins	0.0185185
Drosophila	dnds	Ubiquitin__system	DNA__repair__and__recombination__proteins	0.0185185
Drosophila	dnds	Ubiquitin__system	DNA__replication__proteins	0.0185185
Drosophila	dnds	Ubiquitin__system	Exosome	0.0185185
Drosophila	dnds	Ubiquitin__system	G__protein__coupled__receptors	0.0185185
Drosophila	dnds	Ubiquitin__system	Glycosyltransferases	0.0185185
Drosophila	dnds	Ubiquitin__system	Ion__channels	0.0185185
Drosophila	dnds	Ubiquitin__system	Lipid__biosynthesis__proteins	0.5740741
Drosophila	dnds	Ubiquitin__system	Membrane__trafficking	0.0185185
Drosophila	dnds	Ubiquitin__system	Messenger__RNA__biogenesis	0.0185185
Drosophila	dnds	Ubiquitin__system	Mitochondrial__biogenesis	0.0555556
Drosophila	dnds	Ubiquitin__system	Peptidases	0.3888889
Drosophila	dnds	Ubiquitin__system	Photosynthesis__proteins	0.0555556
Drosophila	dnds	Ubiquitin__system	Proteasome	0.0555556
Drosophila	dnds	Ubiquitin__system	Protein__kinases	0.0185185
Drosophila	dnds	Ubiquitin__system	Protein__phosphatases__and__associated__proteins	0.5000000
Drosophila	dnds	Ubiquitin__system	Ribosome	0.1666667
Drosophila	dnds	Ubiquitin__system	Ribosome__biogenesis	0.2037037
Drosophila	dnds	Ubiquitin__system	Spliceosome	0.7592593
Drosophila	dnds	Ubiquitin__system	Transcription__factors	0.9444444
Drosophila	dnds	Ubiquitin__system	Transcription__machinery	0.0185185
Drosophila	dnds	Ubiquitin__system	Transfer__RNA__biogenesis	0.0555556
Drosophila	dnds	Ubiquitin__system	Translation__factors	0.8703704
Drosophila	dnds	Ubiquitin__system	Transporters	0.0185185
Drosophila	omegaNA	Chaperones__and__folding__catalysts	Amino__acid__related__enzymes	0.7222222
Drosophila	omegaNA	Chromosome__and__associated__proteins	Amino__acid__related__enzymes	0.9444444
Drosophila	omegaNA	Chromosome__and__associated__proteins	Chaperones__and__folding__catalysts	0.4259259
Drosophila	omegaNA	Cytochrome__P450	Amino__acid__related__enzymes	0.1666667
Drosophila	omegaNA	Cytochrome__P450	Chaperones__and__folding__catalysts	0.9814815

(continued)

species	estimate	var1	var2	p.value
Drosophila	omegaNA	Cytochrome__P450	Chromosome__and__associated__proteins	0.4629630
Drosophila	omegaNA	Cytoskeleton__proteins	Amino__acid__related__enzymes	0.9444444
Drosophila	omegaNA	Cytoskeleton__proteins	Chaperones__and__folding__catalysts	0.4629630
Drosophila	omegaNA	Cytoskeleton__proteins	Chromosome__and__associated__proteins	0.6111111
Drosophila	omegaNA	Cytoskeleton__proteins	Cytochrome__P450	0.7222222
Drosophila	omegaNA	DNA__repair__and__recombination__proteins	Amino__acid__related__enzymes	0.5740741
Drosophila	omegaNA	DNA__repair__and__recombination__proteins	Chaperones__and__folding__catalysts	0.5740741
Drosophila	omegaNA	DNA__repair__and__recombination__proteins	Chromosome__and__associated__proteins	0.1296296
Drosophila	omegaNA	DNA__repair__and__recombination__proteins	Cytochrome__P450	0.8333333
Drosophila	omegaNA	DNA__repair__and__recombination__proteins	Cytoskeleton__proteins	0.2037037
Drosophila	omegaNA	DNA__replication__proteins	Amino__acid__related__enzymes	0.3518519
Drosophila	omegaNA	DNA__replication__proteins	Chaperones__and__folding__catalysts	0.4629630
Drosophila	omegaNA	DNA__replication__proteins	Chromosome__and__associated__proteins	0.1666667
Drosophila	omegaNA	DNA__replication__proteins	Cytochrome__P450	0.9074074
Drosophila	omegaNA	DNA__replication__proteins	Cytoskeleton__proteins	0.0925926
Drosophila	omegaNA	DNA__replication__proteins	DNA__repair__and__recombination__proteins	0.7962963
Drosophila	omegaNA	Exosome	Amino__acid__related__enzymes	0.9074074
Drosophila	omegaNA	Exosome	Chaperones__and__folding__catalysts	0.5370370
Drosophila	omegaNA	Exosome	Chromosome__and__associated__proteins	0.6111111
Drosophila	omegaNA	Exosome	Cytochrome__P450	0.7222222
Drosophila	omegaNA	Exosome	Cytoskeleton__proteins	0.8333333
Drosophila	omegaNA	Exosome	DNA__repair__and__recombination__proteins	0.2037037
Drosophila	omegaNA	Exosome	DNA__replication__proteins	0.1296296
Drosophila	omegaNA	G__protein__coupled__receptors	Amino__acid__related__enzymes	0.2777778
Drosophila	omegaNA	G__protein__coupled__receptors	Chaperones__and__folding__catalysts	0.9074074
Drosophila	omegaNA	G__protein__coupled__receptors	Chromosome__and__associated__proteins	0.4259259
Drosophila	omegaNA	G__protein__coupled__receptors	Cytochrome__P450	0.6481481
Drosophila	omegaNA	G__protein__coupled__receptors	Cytoskeleton__proteins	0.6851852
Drosophila	omegaNA	G__protein__coupled__receptors	DNA__repair__and__recombination__proteins	0.8703704
Drosophila	omegaNA	G__protein__coupled__receptors	DNA__replication__proteins	0.8333333
Drosophila	omegaNA	G__protein__coupled__receptors	Exosome	0.7592593
Drosophila	omegaNA	Glycosyltransferases	Amino__acid__related__enzymes	0.3888889
Drosophila	omegaNA	Glycosyltransferases	Chaperones__and__folding__catalysts	0.6851852
Drosophila	omegaNA	Glycosyltransferases	Chromosome__and__associated__proteins	0.1666667
Drosophila	omegaNA	Glycosyltransferases	Cytochrome__P450	0.9444444
Drosophila	omegaNA	Glycosyltransferases	Cytoskeleton__proteins	0.2777778
Drosophila	omegaNA	Glycosyltransferases	DNA__repair__and__recombination__proteins	0.8333333
Drosophila	omegaNA	Glycosyltransferases	DNA__replication__proteins	0.9814815
Drosophila	omegaNA	Glycosyltransferases	Exosome	0.3148148
Drosophila	omegaNA	Glycosyltransferases	G__protein__coupled__receptors	0.8333333
Drosophila	omegaNA	Ion__channels	Amino__acid__related__enzymes	0.7962963
Drosophila	omegaNA	Ion__channels	Chaperones__and__folding__catalysts	0.2037037
Drosophila	omegaNA	Ion__channels	Chromosome__and__associated__proteins	0.5000000
Drosophila	omegaNA	Ion__channels	Cytochrome__P450	0.0925926
Drosophila	omegaNA	Ion__channels	Cytoskeleton__proteins	0.1296296
Drosophila	omegaNA	Ion__channels	DNA__repair__and__recombination__proteins	0.0185185
Drosophila	omegaNA	Ion__channels	DNA__replication__proteins	0.0185185
Drosophila	omegaNA	Ion__channels	Exosome	0.1666667
Drosophila	omegaNA	Ion__channels	G__protein__coupled__receptors	0.0555556
Drosophila	omegaNA	Ion__channels	Glycosyltransferases	0.0185185
Drosophila	omegaNA	Lipid__biosynthesis__proteins	Amino__acid__related__enzymes	0.1296296
Drosophila	omegaNA	Lipid__biosynthesis__proteins	Chaperones__and__folding__catalysts	0.9814815
Drosophila	omegaNA	Lipid__biosynthesis__proteins	Chromosome__and__associated__proteins	0.4259259
Drosophila	omegaNA	Lipid__biosynthesis__proteins	Cytochrome__P450	0.8703704
Drosophila	omegaNA	Lipid__biosynthesis__proteins	Cytoskeleton__proteins	0.7962963
Drosophila	omegaNA	Lipid__biosynthesis__proteins	DNA__repair__and__recombination__proteins	0.9074074
Drosophila	omegaNA	Lipid__biosynthesis__proteins	DNA__replication__proteins	0.8703704
Drosophila	omegaNA	Lipid__biosynthesis__proteins	Exosome	0.8333333
Drosophila	omegaNA	Lipid__biosynthesis__proteins	G__protein__coupled__receptors	0.7592593
Drosophila	omegaNA	Lipid__biosynthesis__proteins	Glycosyltransferases	0.7962963
Drosophila	omegaNA	Lipid__biosynthesis__proteins	Ion__channels	0.1666667
Drosophila	omegaNA	Membrane__trafficking	Amino__acid__related__enzymes	0.9444444

(continued)

species	estimate	var1	var2	p.value
Drosophila	omegaNA	Membrane__trafficking	Chaperones__and__folding__catalysts	0.0925926
Drosophila	omegaNA	Membrane__trafficking	Chromosome__and__associated__proteins	0.0185185
Drosophila	omegaNA	Membrane__trafficking	Cytochrome__P450	0.0925926
Drosophila	omegaNA	Membrane__trafficking	Cytoskeleton__proteins	0.0185185
Drosophila	omegaNA	Membrane__trafficking	DNA__repair__and__recombination__proteins	0.0185185
Drosophila	omegaNA	Membrane__trafficking	DNA__replication__proteins	0.0185185
Drosophila	omegaNA	Membrane__trafficking	Exosome	0.0555556
Drosophila	omegaNA	Membrane__trafficking	G__protein__coupled__receptors	0.0925926
Drosophila	omegaNA	Membrane__trafficking	Glycosyltransferases	0.0555556
Drosophila	omegaNA	Membrane__trafficking	Ion__channels	0.8333333
Drosophila	omegaNA	Membrane__trafficking	Lipid__biosynthesis__proteins	0.0925926
Drosophila	omegaNA	Messenger__RNA__biogenesis	Amino__acid__related__enzymes	0.9444444
Drosophila	omegaNA	Messenger__RNA__biogenesis	Chaperones__and__folding__catalysts	0.3148148
Drosophila	omegaNA	Messenger__RNA__biogenesis	Chromosome__and__associated__proteins	0.7962963
Drosophila	omegaNA	Messenger__RNA__biogenesis	Cytochrome__P450	0.5740741
Drosophila	omegaNA	Messenger__RNA__biogenesis	Cytoskeleton__proteins	0.5370370
Drosophila	omegaNA	Messenger__RNA__biogenesis	DNA__repair__and__recombination__proteins	0.0555556
Drosophila	omegaNA	Messenger__RNA__biogenesis	DNA__replication__proteins	0.0555556
Drosophila	omegaNA	Messenger__RNA__biogenesis	Exosome	0.5370370
Drosophila	omegaNA	Messenger__RNA__biogenesis	G__protein__coupled__receptors	0.5000000
Drosophila	omegaNA	Messenger__RNA__biogenesis	Glycosyltransferases	0.2037037
Drosophila	omegaNA	Messenger__RNA__biogenesis	Ion__channels	0.2037037
Drosophila	omegaNA	Messenger__RNA__biogenesis	Lipid__biosynthesis__proteins	0.5370370
Drosophila	omegaNA	Messenger__RNA__biogenesis	Membrane__trafficking	0.0555556
Drosophila	omegaNA	Mitochondrial__biogenesis	Amino__acid__related__enzymes	0.7592593
Drosophila	omegaNA	Mitochondrial__biogenesis	Chaperones__and__folding__catalysts	0.6111111
Drosophila	omegaNA	Mitochondrial__biogenesis	Chromosome__and__associated__proteins	0.8333333
Drosophila	omegaNA	Mitochondrial__biogenesis	Cytochrome__P450	0.4629630
Drosophila	omegaNA	Mitochondrial__biogenesis	Cytoskeleton__proteins	0.7962963
Drosophila	omegaNA	Mitochondrial__biogenesis	DNA__repair__and__recombination__proteins	0.3518519
Drosophila	omegaNA	Mitochondrial__biogenesis	DNA__replication__proteins	0.4629630
Drosophila	omegaNA	Mitochondrial__biogenesis	Exosome	0.6111111
Drosophila	omegaNA	Mitochondrial__biogenesis	G__protein__coupled__receptors	0.5000000
Drosophila	omegaNA	Mitochondrial__biogenesis	Glycosyltransferases	0.3888889
Drosophila	omegaNA	Mitochondrial__biogenesis	Ion__channels	0.5370370
Drosophila	omegaNA	Mitochondrial__biogenesis	Lipid__biosynthesis__proteins	0.5740741
Drosophila	omegaNA	Mitochondrial__biogenesis	Membrane__trafficking	0.1296296
Drosophila	omegaNA	Mitochondrial__biogenesis	Messenger__RNA__biogenesis	0.8703704
Drosophila	omegaNA	Peptidases	Amino__acid__related__enzymes	0.9444444
Drosophila	omegaNA	Peptidases	Chaperones__and__folding__catalysts	0.0185185
Drosophila	omegaNA	Peptidases	Chromosome__and__associated__proteins	0.0555556
Drosophila	omegaNA	Peptidases	Cytochrome__P450	0.1296296
Drosophila	omegaNA	Peptidases	Cytoskeleton__proteins	0.0185185
Drosophila	omegaNA	Peptidases	DNA__repair__and__recombination__proteins	0.0185185
Drosophila	omegaNA	Peptidases	DNA__replication__proteins	0.0185185
Drosophila	omegaNA	Peptidases	Exosome	0.0555556
Drosophila	omegaNA	Peptidases	G__protein__coupled__receptors	0.0925926
Drosophila	omegaNA	Peptidases	Glycosyltransferases	0.0185185
Drosophila	omegaNA	Peptidases	Ion__channels	0.8703704
Drosophila	omegaNA	Peptidases	Lipid__biosynthesis__proteins	0.0185185
Drosophila	omegaNA	Peptidases	Membrane__trafficking	0.8333333
Drosophila	omegaNA	Peptidases	Messenger__RNA__biogenesis	0.0555556
Drosophila	omegaNA	Peptidases	Mitochondrial__biogenesis	0.1666667
Drosophila	omegaNA	Photosynthesis__proteins	Amino__acid__related__enzymes	0.9814815
Drosophila	omegaNA	Photosynthesis__proteins	Chaperones__and__folding__catalysts	0.3518519
Drosophila	omegaNA	Photosynthesis__proteins	Chromosome__and__associated__proteins	0.7592593
Drosophila	omegaNA	Photosynthesis__proteins	Cytochrome__P450	0.1296296
Drosophila	omegaNA	Photosynthesis__proteins	Cytoskeleton__proteins	0.6481481
Drosophila	omegaNA	Photosynthesis__proteins	DNA__repair__and__recombination__proteins	0.2037037
Drosophila	omegaNA	Photosynthesis__proteins	DNA__replication__proteins	0.0925926
Drosophila	omegaNA	Photosynthesis__proteins	Exosome	0.7222222
Drosophila	omegaNA	Photosynthesis__proteins	G__protein__coupled__receptors	0.0925926

(continued)

species	estimate	var1	var2	p.value
Drosophila	omegaNA	Photosynthesis__proteins	Glycosyltransferases	0.1296296
Drosophila	omegaNA	Photosynthesis__proteins	Ion__channels	0.4629630
Drosophila	omegaNA	Photosynthesis__proteins	Lipid__biosynthesis__proteins	0.0925926
Drosophila	omegaNA	Photosynthesis__proteins	Membrane__trafficking	0.7962963
Drosophila	omegaNA	Photosynthesis__proteins	Messenger__RNA__biogenesis	0.9074074
Drosophila	omegaNA	Photosynthesis__proteins	Mitochondrial__biogenesis	0.7222222
Drosophila	omegaNA	Photosynthesis__proteins	Peptidases	0.7592593
Drosophila	omegaNA	Proteasome	Amino__acid__related__enzymes	0.9444444
Drosophila	omegaNA	Proteasome	Chaperones__and__folding__catalysts	0.3888889
Drosophila	omegaNA	Proteasome	Chromosome__and__associated__proteins	0.9074074
Drosophila	omegaNA	Proteasome	Cytochrome__P450	0.2407407
Drosophila	omegaNA	Proteasome	Cytoskeleton__proteins	0.7592593
Drosophila	omegaNA	Proteasome	DNA__repair__and__recombination__proteins	0.2407407
Drosophila	omegaNA	Proteasome	DNA__replication__proteins	0.1296296
Drosophila	omegaNA	Proteasome	Exosome	0.7592593
Drosophila	omegaNA	Proteasome	G__protein__coupled__receptors	0.2037037
Drosophila	omegaNA	Proteasome	Glycosyltransferases	0.1666667
Drosophila	omegaNA	Proteasome	Ion__channels	0.5370370
Drosophila	omegaNA	Proteasome	Lipid__biosynthesis__proteins	0.1666667
Drosophila	omegaNA	Proteasome	Membrane__trafficking	0.6481481
Drosophila	omegaNA	Proteasome	Messenger__RNA__biogenesis	0.9814815
Drosophila	omegaNA	Proteasome	Mitochondrial__biogenesis	0.8703704
Drosophila	omegaNA	Proteasome	Peptidases	0.6481481
Drosophila	omegaNA	Proteasome	Photosynthesis__proteins	0.8703704
Drosophila	omegaNA	Protein__kinases	Amino__acid__related__enzymes	0.9814815
Drosophila	omegaNA	Protein__kinases	Chaperones__and__folding__catalysts	0.0555556
Drosophila	omegaNA	Protein__kinases	Chromosome__and__associated__proteins	0.0185185
Drosophila	omegaNA	Protein__kinases	Cytochrome__P450	0.0925926
Drosophila	omegaNA	Protein__kinases	Cytoskeleton__proteins	0.0185185
Drosophila	omegaNA	Protein__kinases	DNA__repair__and__recombination__proteins	0.0185185
Drosophila	omegaNA	Protein__kinases	DNA__replication__proteins	0.0185185
Drosophila	omegaNA	Protein__kinases	Exosome	0.0185185
Drosophila	omegaNA	Protein__kinases	G__protein__coupled__receptors	0.0555556
Drosophila	omegaNA	Protein__kinases	Glycosyltransferases	0.0185185
Drosophila	omegaNA	Protein__kinases	Ion__channels	0.8333333
Drosophila	omegaNA	Protein__kinases	Lipid__biosynthesis__proteins	0.0925926
Drosophila	omegaNA	Protein__kinases	Membrane__trafficking	0.7962963
Drosophila	omegaNA	Protein__kinases	Messenger__RNA__biogenesis	0.0185185
Drosophila	omegaNA	Protein__kinases	Mitochondrial__biogenesis	0.2777778
Drosophila	omegaNA	Protein__kinases	Peptidases	0.6851852
Drosophila	omegaNA	Protein__kinases	Photosynthesis__proteins	0.7222222
Drosophila	omegaNA	Protein__kinases	Proteasome	0.6851852
Drosophila	omegaNA	Protein__phosphatases__and__associated__proteins	Amino__acid__related__enzymes	0.9074074
Drosophila	omegaNA	Protein__phosphatases__and__associated__proteins	Chaperones__and__folding__catalysts	0.3518519
Drosophila	omegaNA	Protein__phosphatases__and__associated__proteins	Chromosome__and__associated__proteins	0.6851852
Drosophila	omegaNA	Protein__phosphatases__and__associated__proteins	Cytochrome__P450	0.2037037
Drosophila	omegaNA	Protein__phosphatases__and__associated__proteins	Cytoskeleton__proteins	0.3888889
Drosophila	omegaNA	Protein__phosphatases__and__associated__proteins	DNA__repair__and__recombination__proteins	0.1666667
Drosophila	omegaNA	Protein__phosphatases__and__associated__proteins	DNA__replication__proteins	0.0925926
Drosophila	omegaNA	Protein__phosphatases__and__associated__proteins	Exosome	0.4259259
Drosophila	omegaNA	Protein__phosphatases__and__associated__proteins	G__protein__coupled__receptors	0.2407407
Drosophila	omegaNA	Protein__phosphatases__and__associated__proteins	Glycosyltransferases	0.0555556
Drosophila	omegaNA	Protein__phosphatases__and__associated__proteins	Ion__channels	0.7222222
Drosophila	omegaNA	Protein__phosphatases__and__associated__proteins	Lipid__biosynthesis__proteins	0.3888889
Drosophila	omegaNA	Protein__phosphatases__and__associated__proteins	Membrane__trafficking	0.0925926
Drosophila	omegaNA	Protein__phosphatases__and__associated__proteins	Messenger__RNA__biogenesis	0.3888889
Drosophila	omegaNA	Protein__phosphatases__and__associated__proteins	Mitochondrial__biogenesis	0.9444444
Drosophila	omegaNA	Protein__phosphatases__and__associated__proteins	Peptidases	0.0925926
Drosophila	omegaNA	Protein__phosphatases__and__associated__proteins	Photosynthesis__proteins	0.8333333
Drosophila	omegaNA	Protein__phosphatases__and__associated__proteins	Proteasome	0.9444444
Drosophila	omegaNA	Protein__phosphatases__and__associated__proteins	Protein__kinases	0.0925926

(continued)

species	estimate	var1	var2	p.value
Drosophila	omegaNA	Ribosome	Amino__acid__related__enzymes	0.7222222
Drosophila	omegaNA	Ribosome	Chaperones__and__folding__catalysts	0.6851852
Drosophila	omegaNA	Ribosome	Chromosome__and__associated__proteins	0.9074074
Drosophila	omegaNA	Ribosome	Cytochrome__P450	0.7592593
Drosophila	omegaNA	Ribosome	Cytoskeleton__proteins	0.9074074
Drosophila	omegaNA	Ribosome	DNA__repair__and__recombination__proteins	0.4629630
Drosophila	omegaNA	Ribosome	DNA__replication__proteins	0.3148148
Drosophila	omegaNA	Ribosome	Exosome	0.9074074
Drosophila	omegaNA	Ribosome	G__protein__coupled__receptors	0.6851852
Drosophila	omegaNA	Ribosome	Glycosyltransferases	0.3888889
Drosophila	omegaNA	Ribosome	Ion__channels	0.4629630
Drosophila	omegaNA	Ribosome	Lipid__biosynthesis__proteins	0.6851852
Drosophila	omegaNA	Ribosome	Membrane__trafficking	0.0185185
Drosophila	omegaNA	Ribosome	Messenger__RNA__biogenesis	0.9814815
Drosophila	omegaNA	Ribosome	Mitochondrial__biogenesis	0.8703704
Drosophila	omegaNA	Ribosome	Peptidases	0.0555556
Drosophila	omegaNA	Ribosome	Photosynthesis__proteins	0.5370370
Drosophila	omegaNA	Ribosome	Proteasome	0.7592593
Drosophila	omegaNA	Ribosome	Protein__kinases	0.0185185
Drosophila	omegaNA	Ribosome	Protein__phosphatases__and__associated__proteins	0.5740741
Drosophila	omegaNA	Ribosome__biogenesis	Amino__acid__related__enzymes	0.9444444
Drosophila	omegaNA	Ribosome__biogenesis	Chaperones__and__folding__catalysts	0.2037037
Drosophila	omegaNA	Ribosome__biogenesis	Chromosome__and__associated__proteins	0.4259259
Drosophila	omegaNA	Ribosome__biogenesis	Cytochrome__P450	0.1666667
Drosophila	omegaNA	Ribosome__biogenesis	Cytoskeleton__proteins	0.1666667
Drosophila	omegaNA	Ribosome__biogenesis	DNA__repair__and__recombination__proteins	0.0555556
Drosophila	omegaNA	Ribosome__biogenesis	DNA__replication__proteins	0.0185185
Drosophila	omegaNA	Ribosome__biogenesis	Exosome	0.0925926
Drosophila	omegaNA	Ribosome__biogenesis	G__protein__coupled__receptors	0.1296296
Drosophila	omegaNA	Ribosome__biogenesis	Glycosyltransferases	0.0185185
Drosophila	omegaNA	Ribosome__biogenesis	Ion__channels	0.9074074
Drosophila	omegaNA	Ribosome__biogenesis	Lipid__biosynthesis__proteins	0.1296296
Drosophila	omegaNA	Ribosome__biogenesis	Membrane__trafficking	0.1296296
Drosophila	omegaNA	Ribosome__biogenesis	Messenger__RNA__biogenesis	0.2037037
Drosophila	omegaNA	Ribosome__biogenesis	Mitochondrial__biogenesis	0.7222222
Drosophila	omegaNA	Ribosome__biogenesis	Peptidases	0.0925926
Drosophila	omegaNA	Ribosome__biogenesis	Photosynthesis__proteins	0.9814815
Drosophila	omegaNA	Ribosome__biogenesis	Proteasome	0.7962963
Drosophila	omegaNA	Ribosome__biogenesis	Protein__kinases	0.0555556
Drosophila	omegaNA	Ribosome__biogenesis	Protein__phosphatases__and__associated__proteins	0.6111111
Drosophila	omegaNA	Ribosome__biogenesis	Ribosome	0.2777778
Drosophila	omegaNA	Spliceosome	Amino__acid__related__enzymes	0.7592593
Drosophila	omegaNA	Spliceosome	Chaperones__and__folding__catalysts	0.9814815
Drosophila	omegaNA	Spliceosome	Chromosome__and__associated__proteins	0.3518519
Drosophila	omegaNA	Spliceosome	Cytochrome__P450	0.9444444
Drosophila	omegaNA	Spliceosome	Cytoskeleton__proteins	0.6481481
Drosophila	omegaNA	Spliceosome	DNA__repair__and__recombination__proteins	0.5000000
Drosophila	omegaNA	Spliceosome	DNA__replication__proteins	0.3148148
Drosophila	omegaNA	Spliceosome	Exosome	0.5740741
Drosophila	omegaNA	Spliceosome	G__protein__coupled__receptors	0.8333333
Drosophila	omegaNA	Spliceosome	Glycosyltransferases	0.5000000
Drosophila	omegaNA	Spliceosome	Ion__channels	0.0555556
Drosophila	omegaNA	Spliceosome	Lipid__biosynthesis__proteins	0.9444444
Drosophila	omegaNA	Spliceosome	Membrane__trafficking	0.0185185
Drosophila	omegaNA	Spliceosome	Messenger__RNA__biogenesis	0.2037037
Drosophila	omegaNA	Spliceosome	Mitochondrial__biogenesis	0.6111111
Drosophila	omegaNA	Spliceosome	Peptidases	0.0185185
Drosophila	omegaNA	Spliceosome	Photosynthesis__proteins	0.4629630
Drosophila	omegaNA	Spliceosome	Proteasome	0.5370370
Drosophila	omegaNA	Spliceosome	Protein__kinases	0.0185185
Drosophila	omegaNA	Spliceosome	Protein__phosphatases__and__associated__proteins	0.1666667
Drosophila	omegaNA	Spliceosome	Ribosome	0.5740741

(continued)

species	estimate	var1	var2	p.value
Drosophila	omegaNA	Spliceosome	Ribosome__biogenesis	0.0185185
Drosophila	omegaNA	Transcription__factors	Amino__acid__related__enzymes	0.7592593
Drosophila	omegaNA	Transcription__factors	Chaperones__and__folding__catalysts	0.8703704
Drosophila	omegaNA	Transcription__factors	Chromosome__and__associated__proteins	0.2037037
Drosophila	omegaNA	Transcription__factors	Cytochrome__P450	0.9814815
Drosophila	omegaNA	Transcription__factors	Cytoskeleton__proteins	0.2407407
Drosophila	omegaNA	Transcription__factors	DNA__repair__and__recombination__proteins	0.5740741
Drosophila	omegaNA	Transcription__factors	DNA__replication__proteins	0.3148148
Drosophila	omegaNA	Transcription__factors	Exosome	0.3148148
Drosophila	omegaNA	Transcription__factors	G__protein__coupled__receptors	0.8333333
Drosophila	omegaNA	Transcription__factors	Glycosyltransferases	0.5000000
Drosophila	omegaNA	Transcription__factors	Ion__channels	0.0185185
Drosophila	omegaNA	Transcription__factors	Lipid__biosynthesis__proteins	0.9814815
Drosophila	omegaNA	Transcription__factors	Membrane__trafficking	0.0185185
Drosophila	omegaNA	Transcription__factors	Messenger__RNA__biogenesis	0.0555556
Drosophila	omegaNA	Transcription__factors	Mitochondrial__biogenesis	0.5740741
Drosophila	omegaNA	Transcription__factors	Peptidases	0.0185185
Drosophila	omegaNA	Transcription__factors	Photosynthesis__proteins	0.1666667
Drosophila	omegaNA	Transcription__factors	Proteasome	0.3888889
Drosophila	omegaNA	Transcription__factors	Protein__kinases	0.0185185
Drosophila	omegaNA	Transcription__factors	Protein__phosphatases__and__associated__proteins	0.1296296
Drosophila	omegaNA	Transcription__factors	Ribosome	0.6111111
Drosophila	omegaNA	Transcription__factors	Ribosome__biogenesis	0.0185185
Drosophila	omegaNA	Transcription__factors	Spliceosome	0.8703704
Drosophila	omegaNA	Transcription__machinery	Amino__acid__related__enzymes	0.9444444
Drosophila	omegaNA	Transcription__machinery	Chaperones__and__folding__catalysts	0.0555556
Drosophila	omegaNA	Transcription__machinery	Chromosome__and__associated__proteins	0.1666667
Drosophila	omegaNA	Transcription__machinery	Cytochrome__P450	0.1296296
Drosophila	omegaNA	Transcription__machinery	Cytoskeleton__proteins	0.0555556
Drosophila	omegaNA	Transcription__machinery	DNA__repair__and__recombination__proteins	0.0185185
Drosophila	omegaNA	Transcription__machinery	DNA__replication__proteins	0.0185185
Drosophila	omegaNA	Transcription__machinery	Exosome	0.0185185
Drosophila	omegaNA	Transcription__machinery	G__protein__coupled__receptors	0.0555556
Drosophila	omegaNA	Transcription__machinery	Glycosyltransferases	0.0185185
Drosophila	omegaNA	Transcription__machinery	Ion__channels	0.9444444
Drosophila	omegaNA	Transcription__machinery	Lipid__biosynthesis__proteins	0.0925926
Drosophila	omegaNA	Transcription__machinery	Membrane__trafficking	0.5740741
Drosophila	omegaNA	Transcription__machinery	Messenger__RNA__biogenesis	0.0185185
Drosophila	omegaNA	Transcription__machinery	Mitochondrial__biogenesis	0.4259259
Drosophila	omegaNA	Transcription__machinery	Peptidases	0.3518519
Drosophila	omegaNA	Transcription__machinery	Photosynthesis__proteins	0.9444444
Drosophila	omegaNA	Transcription__machinery	Proteasome	0.7962963
Drosophila	omegaNA	Transcription__machinery	Protein__kinases	0.6481481
Drosophila	omegaNA	Transcription__machinery	Protein__phosphatases__and__associated__proteins	0.3148148
Drosophila	omegaNA	Transcription__machinery	Ribosome	0.0185185
Drosophila	omegaNA	Transcription__machinery	Ribosome__biogenesis	0.5370370
Drosophila	omegaNA	Transcription__machinery	Spliceosome	0.0185185
Drosophila	omegaNA	Transcription__machinery	Transcription__factors	0.0185185
Drosophila	omegaNA	Transfer__RNA__biogenesis	Amino__acid__related__enzymes	0.9444444
Drosophila	omegaNA	Transfer__RNA__biogenesis	Chaperones__and__folding__catalysts	0.4259259
Drosophila	omegaNA	Transfer__RNA__biogenesis	Chromosome__and__associated__proteins	0.5000000
Drosophila	omegaNA	Transfer__RNA__biogenesis	Cytochrome__P450	0.5740741
Drosophila	omegaNA	Transfer__RNA__biogenesis	Cytoskeleton__proteins	0.6481481
Drosophila	omegaNA	Transfer__RNA__biogenesis	DNA__repair__and__recombination__proteins	0.0555556
Drosophila	omegaNA	Transfer__RNA__biogenesis	DNA__replication__proteins	0.0185185
Drosophila	omegaNA	Transfer__RNA__biogenesis	Exosome	0.6851852
Drosophila	omegaNA	Transfer__RNA__biogenesis	G__protein__coupled__receptors	0.6851852
Drosophila	omegaNA	Transfer__RNA__biogenesis	Glycosyltransferases	0.2037037
Drosophila	omegaNA	Transfer__RNA__biogenesis	Ion__channels	0.1296296
Drosophila	omegaNA	Transfer__RNA__biogenesis	Lipid__biosynthesis__proteins	0.5740741
Drosophila	omegaNA	Transfer__RNA__biogenesis	Membrane__trafficking	0.0185185
Drosophila	omegaNA	Transfer__RNA__biogenesis	Messenger__RNA__biogenesis	0.6851852

(continued)

species	estimate	var1	var2	p.value
Drosophila	omegaNA	Transfer__RNA__biogenesis	Mitochondrial__biogenesis	0.6851852
Drosophila	omegaNA	Transfer__RNA__biogenesis	Peptidases	0.0185185
Drosophila	omegaNA	Transfer__RNA__biogenesis	Photosynthesis__proteins	0.7962963
Drosophila	omegaNA	Transfer__RNA__biogenesis	Proteasome	0.9074074
Drosophila	omegaNA	Transfer__RNA__biogenesis	Protein__kinases	0.0185185
Drosophila	omegaNA	Transfer__RNA__biogenesis	Protein__phosphatases__and__associated__proteins	0.4259259
Drosophila	omegaNA	Transfer__RNA__biogenesis	Ribosome	0.9074074
Drosophila	omegaNA	Transfer__RNA__biogenesis	Ribosome__biogenesis	0.2037037
Drosophila	omegaNA	Transfer__RNA__biogenesis	Spliceosome	0.3148148
Drosophila	omegaNA	Transfer__RNA__biogenesis	Transcription__factors	0.0925926
Drosophila	omegaNA	Transfer__RNA__biogenesis	Transcription__machinery	0.0185185
Drosophila	omegaNA	Translation__factors	Amino__acid__related__enzymes	0.1666667
Drosophila	omegaNA	Translation__factors	Chaperones__and__folding__catalysts	0.9814815
Drosophila	omegaNA	Translation__factors	Chromosome__and__associated__proteins	0.6111111
Drosophila	omegaNA	Translation__factors	Cytochrome__P450	0.7962963
Drosophila	omegaNA	Translation__factors	Cytoskeleton__proteins	0.9074074
Drosophila	omegaNA	Translation__factors	DNA__repair__and__recombination__proteins	0.7962963
Drosophila	omegaNA	Translation__factors	DNA__replication__proteins	0.8333333
Drosophila	omegaNA	Translation__factors	Exosome	0.8333333
Drosophila	omegaNA	Translation__factors	G__protein__coupled__receptors	0.7222222
Drosophila	omegaNA	Translation__factors	Glycosyltransferases	0.9814815
Drosophila	omegaNA	Translation__factors	Ion__channels	0.1666667
Drosophila	omegaNA	Translation__factors	Lipid__biosynthesis__proteins	0.7962963
Drosophila	omegaNA	Translation__factors	Membrane__trafficking	0.1296296
Drosophila	omegaNA	Translation__factors	Messenger__RNA__biogenesis	0.7592593
Drosophila	omegaNA	Translation__factors	Mitochondrial__biogenesis	0.5740741
Drosophila	omegaNA	Translation__factors	Peptidases	0.2037037
Drosophila	omegaNA	Translation__factors	Photosynthesis__proteins	0.1666667
Drosophila	omegaNA	Translation__factors	Proteasome	0.2407407
Drosophila	omegaNA	Translation__factors	Protein__kinases	0.1296296
Drosophila	omegaNA	Translation__factors	Protein__phosphatases__and__associated__proteins	0.3148148
Drosophila	omegaNA	Translation__factors	Ribosome	0.7962963
Drosophila	omegaNA	Translation__factors	Ribosome__biogenesis	0.3148148
Drosophila	omegaNA	Translation__factors	Spliceosome	0.9814815
Drosophila	omegaNA	Translation__factors	Transcription__factors	0.9444444
Drosophila	omegaNA	Translation__factors	Transcription__machinery	0.2037037
Drosophila	omegaNA	Translation__factors	Transfer__RNA__biogenesis	0.6481481
Drosophila	omegaNA	Transporters	Amino__acid__related__enzymes	0.9444444
Drosophila	omegaNA	Transporters	Chaperones__and__folding__catalysts	0.1666667
Drosophila	omegaNA	Transporters	Chromosome__and__associated__proteins	0.7962963
Drosophila	omegaNA	Transporters	Cytochrome__P450	0.3518519
Drosophila	omegaNA	Transporters	Cytoskeleton__proteins	0.2777778
Drosophila	omegaNA	Transporters	DNA__repair__and__recombination__proteins	0.0185185
Drosophila	omegaNA	Transporters	DNA__replication__proteins	0.0185185
Drosophila	omegaNA	Transporters	Exosome	0.2407407
Drosophila	omegaNA	Transporters	G__protein__coupled__receptors	0.3148148
Drosophila	omegaNA	Transporters	Glycosyltransferases	0.0925926
Drosophila	omegaNA	Transporters	Ion__channels	0.6481481
Drosophila	omegaNA	Transporters	Lipid__biosynthesis__proteins	0.2407407
Drosophila	omegaNA	Transporters	Membrane__trafficking	0.0555556
Drosophila	omegaNA	Transporters	Messenger__RNA__biogenesis	0.3518519
Drosophila	omegaNA	Transporters	Mitochondrial__biogenesis	0.9814815
Drosophila	omegaNA	Transporters	Peptidases	0.0925926
Drosophila	omegaNA	Transporters	Photosynthesis__proteins	0.9814815
Drosophila	omegaNA	Transporters	Proteasome	0.9074074
Drosophila	omegaNA	Transporters	Protein__kinases	0.0185185
Drosophila	omegaNA	Transporters	Protein__phosphatases__and__associated__proteins	0.9074074
Drosophila	omegaNA	Transporters	Ribosome	0.6111111
Drosophila	omegaNA	Transporters	Ribosome__biogenesis	0.7222222
Drosophila	omegaNA	Transporters	Spliceosome	0.1296296
Drosophila	omegaNA	Transporters	Transcription__factors	0.0185185

(continued)

species	estimate	var1	var2	p.value
Drosophila	omegaNA	Transporters	Transcription__machinery	0.2777778
Drosophila	omegaNA	Transporters	Transfer__RNA__biogenesis	0.3888889
Drosophila	omegaNA	Transporters	Translation__factors	0.5370370
Drosophila	omegaNA	Ubiquitin__system	Amino__acid__related__enzymes	0.9444444
Drosophila	omegaNA	Ubiquitin__system	Chaperones__and__folding__catalysts	0.3518519
Drosophila	omegaNA	Ubiquitin__system	Chromosome__and__associated__proteins	0.9814815
Drosophila	omegaNA	Ubiquitin__system	Cytochrome__P450	0.4259259
Drosophila	omegaNA	Ubiquitin__system	Cytoskeleton__proteins	0.5740741
Drosophila	omegaNA	Ubiquitin__system	DNA__repair__and__recombination__proteins	0.0185185
Drosophila	omegaNA	Ubiquitin__system	DNA__replication__proteins	0.0185185
Drosophila	omegaNA	Ubiquitin__system	Exosome	0.4259259
Drosophila	omegaNA	Ubiquitin__system	G__protein__coupled__receptors	0.4259259
Drosophila	omegaNA	Ubiquitin__system	Glycosyltransferases	0.1666667
Drosophila	omegaNA	Ubiquitin__system	Ion__channels	0.3518519
Drosophila	omegaNA	Ubiquitin__system	Lipid__biosynthesis__proteins	0.3888889
Drosophila	omegaNA	Ubiquitin__system	Membrane__trafficking	0.0925926
Drosophila	omegaNA	Ubiquitin__system	Messenger__RNA__biogenesis	0.9444444
Drosophila	omegaNA	Ubiquitin__system	Mitochondrial__biogenesis	0.9814815
Drosophila	omegaNA	Ubiquitin__system	Peptidases	0.0185185
Drosophila	omegaNA	Ubiquitin__system	Photosynthesis__proteins	0.8703704
Drosophila	omegaNA	Ubiquitin__system	Proteasome	0.9444444
Drosophila	omegaNA	Ubiquitin__system	Protein__kinases	0.1296296
Drosophila	omegaNA	Ubiquitin__system	Protein__phosphatases__and__associated__proteins	0.6851852
Drosophila	omegaNA	Ubiquitin__system	Ribosome	0.8333333
Drosophila	omegaNA	Ubiquitin__system	Ribosome__biogenesis	0.4629630
Drosophila	omegaNA	Ubiquitin__system	Spliceosome	0.3148148
Drosophila	omegaNA	Ubiquitin__system	Transcription__factors	0.0925926
Drosophila	omegaNA	Ubiquitin__system	Transcription__machinery	0.2037037
Drosophila	omegaNA	Ubiquitin__system	Transfer__RNA__biogenesis	0.6481481
Drosophila	omegaNA	Ubiquitin__system	Translation__factors	0.6481481
Drosophila	omegaNA	Ubiquitin__system	Transporters	0.6851852
Drosophila	omegaA	Chaperones__and__folding__catalysts	Amino__acid__related__enzymes	0.7962963
Drosophila	omegaA	Chromosome__and__associated__proteins	Amino__acid__related__enzymes	0.9814815
Drosophila	omegaA	Chromosome__and__associated__proteins	Chaperones__and__folding__catalysts	0.2037037
Drosophila	omegaA	Cytochrome__P450	Amino__acid__related__enzymes	0.9074074
Drosophila	omegaA	Cytochrome__P450	Chaperones__and__folding__catalysts	0.3148148
Drosophila	omegaA	Cytochrome__P450	Chromosome__and__associated__proteins	0.8703704
Drosophila	omegaA	Cytoskeleton__proteins	Amino__acid__related__enzymes	0.5370370
Drosophila	omegaA	Cytoskeleton__proteins	Chaperones__and__folding__catalysts	0.7962963
Drosophila	omegaA	Cytoskeleton__proteins	Chromosome__and__associated__proteins	0.0185185
Drosophila	omegaA	Cytoskeleton__proteins	Cytochrome__P450	0.0925926
Drosophila	omegaA	DNA__repair__and__recombination__proteins	Amino__acid__related__enzymes	0.8703704
Drosophila	omegaA	DNA__repair__and__recombination__proteins	Chaperones__and__folding__catalysts	0.2777778
Drosophila	omegaA	DNA__repair__and__recombination__proteins	Chromosome__and__associated__proteins	0.6481481
Drosophila	omegaA	DNA__repair__and__recombination__proteins	Cytochrome__P450	0.9444444
Drosophila	omegaA	DNA__repair__and__recombination__proteins	Cytoskeleton__proteins	0.1296296
Drosophila	omegaA	DNA__replication__proteins	Amino__acid__related__enzymes	0.4259259
Drosophila	omegaA	DNA__replication__proteins	Chaperones__and__folding__catalysts	0.1666667
Drosophila	omegaA	DNA__replication__proteins	Chromosome__and__associated__proteins	0.0185185
Drosophila	omegaA	DNA__replication__proteins	Cytochrome__P450	0.0555556
Drosophila	omegaA	DNA__replication__proteins	Cytoskeleton__proteins	0.1666667
Drosophila	omegaA	DNA__replication__proteins	DNA__repair__and__recombination__proteins	0.0185185
Drosophila	omegaA	Exosome	Amino__acid__related__enzymes	0.6851852
Drosophila	omegaA	Exosome	Chaperones__and__folding__catalysts	0.6111111
Drosophila	omegaA	Exosome	Chromosome__and__associated__proteins	0.0185185
Drosophila	omegaA	Exosome	Cytochrome__P450	0.0925926
Drosophila	omegaA	Exosome	Cytoskeleton__proteins	0.8703704
Drosophila	omegaA	Exosome	DNA__repair__and__recombination__proteins	0.0925926
Drosophila	omegaA	Exosome	DNA__replication__proteins	0.0925926
Drosophila	omegaA	G__protein__coupled__receptors	Amino__acid__related__enzymes	0.6481481
Drosophila	omegaA	G__protein__coupled__receptors	Chaperones__and__folding__catalysts	0.1666667
Drosophila	omegaA	G__protein__coupled__receptors	Chromosome__and__associated__proteins	0.9444444

(continued)

species	estimate	var1	var2	p.value
Drosophila	omegaA	G__protein__coupled__receptors	Cytochrome__P450	0.9444444
Drosophila	omegaA	G__protein__coupled__receptors	Cytoskeleton__proteins	0.1666667
Drosophila	omegaA	G__protein__coupled__receptors	DNA__repair__and__recombination__proteins	0.9444444
Drosophila	omegaA	G__protein__coupled__receptors	DNA__replication__proteins	0.0185185
Drosophila	omegaA	G__protein__coupled__receptors	Exosome	0.0185185
Drosophila	omegaA	Glycosyltransferases	Amino__acid__related__enzymes	0.3888889
Drosophila	omegaA	Glycosyltransferases	Chaperones__and__folding__catalysts	0.3148148
Drosophila	omegaA	Glycosyltransferases	Chromosome__and__associated__proteins	0.0555556
Drosophila	omegaA	Glycosyltransferases	Cytochrome__P450	0.0555556
Drosophila	omegaA	Glycosyltransferases	Cytoskeleton__proteins	0.3518519
Drosophila	omegaA	Glycosyltransferases	DNA__repair__and__recombination__proteins	0.0555556
Drosophila	omegaA	Glycosyltransferases	DNA__replication__proteins	0.5740741
Drosophila	omegaA	Glycosyltransferases	Exosome	0.2777778
Drosophila	omegaA	Glycosyltransferases	G__protein__coupled__receptors	0.0185185
Drosophila	omegaA	Ion__channels	Amino__acid__related__enzymes	0.7222222
Drosophila	omegaA	Ion__channels	Chaperones__and__folding__catalysts	0.7222222
Drosophila	omegaA	Ion__channels	Chromosome__and__associated__proteins	0.0555556
Drosophila	omegaA	Ion__channels	Cytochrome__P450	0.1666667
Drosophila	omegaA	Ion__channels	Cytoskeleton__proteins	0.3148148
Drosophila	omegaA	Ion__channels	DNA__repair__and__recombination__proteins	0.2777778
Drosophila	omegaA	Ion__channels	DNA__replication__proteins	0.0185185
Drosophila	omegaA	Ion__channels	Exosome	0.1296296
Drosophila	omegaA	Ion__channels	G__protein__coupled__receptors	0.4259259
Drosophila	omegaA	Ion__channels	Glycosyltransferases	0.0555556
Drosophila	omegaA	Lipid__biosynthesis__proteins	Amino__acid__related__enzymes	0.9074074
Drosophila	omegaA	Lipid__biosynthesis__proteins	Chaperones__and__folding__catalysts	0.2037037
Drosophila	omegaA	Lipid__biosynthesis__proteins	Chromosome__and__associated__proteins	0.7962963
Drosophila	omegaA	Lipid__biosynthesis__proteins	Cytochrome__P450	0.9074074
Drosophila	omegaA	Lipid__biosynthesis__proteins	Cytoskeleton__proteins	0.0185185
Drosophila	omegaA	Lipid__biosynthesis__proteins	DNA__repair__and__recombination__proteins	0.9814815
Drosophila	omegaA	Lipid__biosynthesis__proteins	DNA__replication__proteins	0.0185185
Drosophila	omegaA	Lipid__biosynthesis__proteins	Exosome	0.0185185
Drosophila	omegaA	Lipid__biosynthesis__proteins	G__protein__coupled__receptors	0.9814815
Drosophila	omegaA	Lipid__biosynthesis__proteins	Glycosyltransferases	0.0185185
Drosophila	omegaA	Lipid__biosynthesis__proteins	Ion__channels	0.2407407
Drosophila	omegaA	Membrane__trafficking	Amino__acid__related__enzymes	0.7222222
Drosophila	omegaA	Membrane__trafficking	Chaperones__and__folding__catalysts	0.7592593
Drosophila	omegaA	Membrane__trafficking	Chromosome__and__associated__proteins	0.0185185
Drosophila	omegaA	Membrane__trafficking	Cytochrome__P450	0.2037037
Drosophila	omegaA	Membrane__trafficking	Cytoskeleton__proteins	0.3888889
Drosophila	omegaA	Membrane__trafficking	DNA__repair__and__recombination__proteins	0.1296296
Drosophila	omegaA	Membrane__trafficking	DNA__replication__proteins	0.0185185
Drosophila	omegaA	Membrane__trafficking	Exosome	0.2777778
Drosophila	omegaA	Membrane__trafficking	G__protein__coupled__receptors	0.0555556
Drosophila	omegaA	Membrane__trafficking	Glycosyltransferases	0.0925926
Drosophila	omegaA	Membrane__trafficking	Ion__channels	0.8333333
Drosophila	omegaA	Membrane__trafficking	Lipid__biosynthesis__proteins	0.1296296
Drosophila	omegaA	Messenger__RNA__biogenesis	Amino__acid__related__enzymes	0.2777778
Drosophila	omegaA	Messenger__RNA__biogenesis	Chaperones__and__folding__catalysts	0.0185185
Drosophila	omegaA	Messenger__RNA__biogenesis	Chromosome__and__associated__proteins	0.0925926
Drosophila	omegaA	Messenger__RNA__biogenesis	Cytochrome__P450	0.2777778
Drosophila	omegaA	Messenger__RNA__biogenesis	Cytoskeleton__proteins	0.0185185
Drosophila	omegaA	Messenger__RNA__biogenesis	DNA__repair__and__recombination__proteins	0.0185185
Drosophila	omegaA	Messenger__RNA__biogenesis	DNA__replication__proteins	0.0185185
Drosophila	omegaA	Messenger__RNA__biogenesis	Exosome	0.0185185
Drosophila	omegaA	Messenger__RNA__biogenesis	G__protein__coupled__receptors	0.0925926
Drosophila	omegaA	Messenger__RNA__biogenesis	Glycosyltransferases	0.0185185
Drosophila	omegaA	Messenger__RNA__biogenesis	Ion__channels	0.0185185
Drosophila	omegaA	Messenger__RNA__biogenesis	Lipid__biosynthesis__proteins	0.2037037
Drosophila	omegaA	Messenger__RNA__biogenesis	Membrane__trafficking	0.0185185
Drosophila	omegaA	Mitochondrial__biogenesis	Amino__acid__related__enzymes	0.6111111
Drosophila	omegaA	Mitochondrial__biogenesis	Chaperones__and__folding__catalysts	0.9814815

(continued)

species	estimate	var1	var2	p.value
Drosophila	omegaA	Mitochondrial__biogenesis	Chromosome__and__associated__proteins	0.1666667
Drosophila	omegaA	Mitochondrial__biogenesis	Cytochrome__P450	0.3518519
Drosophila	omegaA	Mitochondrial__biogenesis	Cytoskeleton__proteins	0.8333333
Drosophila	omegaA	Mitochondrial__biogenesis	DNA__repair__and__recombination__proteins	0.3888889
Drosophila	omegaA	Mitochondrial__biogenesis	DNA__replication__proteins	0.3888889
Drosophila	omegaA	Mitochondrial__biogenesis	Exosome	0.7222222
Drosophila	omegaA	Mitochondrial__biogenesis	G__protein__coupled__receptors	0.3518519
Drosophila	omegaA	Mitochondrial__biogenesis	Glycosyltransferases	0.4259259
Drosophila	omegaA	Mitochondrial__biogenesis	Ion__channels	0.9074074
Drosophila	omegaA	Mitochondrial__biogenesis	Lipid__biosynthesis__proteins	0.3518519
Drosophila	omegaA	Mitochondrial__biogenesis	Membrane__trafficking	0.9444444
Drosophila	omegaA	Mitochondrial__biogenesis	Messenger__RNA__biogenesis	0.0185185
Drosophila	omegaA	Peptidases	Amino__acid__related__enzymes	0.4259259
Drosophila	omegaA	Peptidases	Chaperones__and__folding__catalysts	0.0555556
Drosophila	omegaA	Peptidases	Chromosome__and__associated__proteins	0.2037037
Drosophila	omegaA	Peptidases	Cytochrome__P450	0.4259259
Drosophila	omegaA	Peptidases	Cytoskeleton__proteins	0.0185185
Drosophila	omegaA	Peptidases	DNA__repair__and__recombination__proteins	0.0555556
Drosophila	omegaA	Peptidases	DNA__replication__proteins	0.0185185
Drosophila	omegaA	Peptidases	Exosome	0.0185185
Drosophila	omegaA	Peptidases	G__protein__coupled__receptors	0.3518519
Drosophila	omegaA	Peptidases	Glycosyltransferases	0.0185185
Drosophila	omegaA	Peptidases	Ion__channels	0.0185185
Drosophila	omegaA	Peptidases	Lipid__biosynthesis__proteins	0.2037037
Drosophila	omegaA	Peptidases	Membrane__trafficking	0.0185185
Drosophila	omegaA	Peptidases	Messenger__RNA__biogenesis	0.8333333
Drosophila	omegaA	Peptidases	Mitochondrial__biogenesis	0.0555556
Drosophila	omegaA	Photosynthesis__proteins	Amino__acid__related__enzymes	0.7962963
Drosophila	omegaA	Photosynthesis__proteins	Chaperones__and__folding__catalysts	0.6481481
Drosophila	omegaA	Photosynthesis__proteins	Chromosome__and__associated__proteins	0.3518519
Drosophila	omegaA	Photosynthesis__proteins	Cytochrome__P450	0.3888889
Drosophila	omegaA	Photosynthesis__proteins	Cytoskeleton__proteins	0.5740741
Drosophila	omegaA	Photosynthesis__proteins	DNA__repair__and__recombination__proteins	0.5370370
Drosophila	omegaA	Photosynthesis__proteins	DNA__replication__proteins	0.1296296
Drosophila	omegaA	Photosynthesis__proteins	Exosome	0.5000000
Drosophila	omegaA	Photosynthesis__proteins	G__protein__coupled__receptors	0.3888889
Drosophila	omegaA	Photosynthesis__proteins	Glycosyltransferases	0.2777778
Drosophila	omegaA	Photosynthesis__proteins	Ion__channels	0.7962963
Drosophila	omegaA	Photosynthesis__proteins	Lipid__biosynthesis__proteins	0.4629630
Drosophila	omegaA	Photosynthesis__proteins	Membrane__trafficking	0.7962963
Drosophila	omegaA	Photosynthesis__proteins	Messenger__RNA__biogenesis	0.0555556
Drosophila	omegaA	Photosynthesis__proteins	Mitochondrial__biogenesis	0.7592593
Drosophila	omegaA	Photosynthesis__proteins	Peptidases	0.0925926
Drosophila	omegaA	Proteasome	Amino__acid__related__enzymes	0.7592593
Drosophila	omegaA	Proteasome	Chaperones__and__folding__catalysts	0.5370370
Drosophila	omegaA	Proteasome	Chromosome__and__associated__proteins	0.4259259
Drosophila	omegaA	Proteasome	Cytochrome__P450	0.6111111
Drosophila	omegaA	Proteasome	Cytoskeleton__proteins	0.5740741
Drosophila	omegaA	Proteasome	DNA__repair__and__recombination__proteins	0.5740741
Drosophila	omegaA	Proteasome	DNA__replication__proteins	0.2037037
Drosophila	omegaA	Proteasome	Exosome	0.4629630
Drosophila	omegaA	Proteasome	G__protein__coupled__receptors	0.3518519
Drosophila	omegaA	Proteasome	Glycosyltransferases	0.2037037
Drosophila	omegaA	Proteasome	Ion__channels	0.9074074
Drosophila	omegaA	Proteasome	Lipid__biosynthesis__proteins	0.4629630
Drosophila	omegaA	Proteasome	Membrane__trafficking	0.6851852
Drosophila	omegaA	Proteasome	Messenger__RNA__biogenesis	0.0555556
Drosophila	omegaA	Proteasome	Mitochondrial__biogenesis	0.7592593
Drosophila	omegaA	Proteasome	Peptidases	0.0555556
Drosophila	omegaA	Proteasome	Photosynthesis__proteins	0.9814815
Drosophila	omegaA	Protein__kinases	Amino__acid__related__enzymes	0.7222222

(continued)

species	estimate	var1	var2	p.value
Drosophila	omegaA	Protein__kinases	Chaperones__and__folding__catalysts	0.5370370
Drosophila	omegaA	Protein__kinases	Chromosome__and__associated__proteins	0.0185185
Drosophila	omegaA	Protein__kinases	Cytochrome__P450	0.3148148
Drosophila	omegaA	Protein__kinases	Cytoskeleton__proteins	0.2037037
Drosophila	omegaA	Protein__kinases	DNA__repair__and__recombination__proteins	0.3148148
Drosophila	omegaA	Protein__kinases	DNA__replication__proteins	0.0185185
Drosophila	omegaA	Protein__kinases	Exosome	0.0555556
Drosophila	omegaA	Protein__kinases	G__protein__coupled__receptors	0.3888889
Drosophila	omegaA	Protein__kinases	Glycosyltransferases	0.0925926
Drosophila	omegaA	Protein__kinases	Ion__channels	0.6111111
Drosophila	omegaA	Protein__kinases	Lipid__biosynthesis__proteins	0.3518519
Drosophila	omegaA	Protein__kinases	Membrane__trafficking	0.3148148
Drosophila	omegaA	Protein__kinases	Messenger__RNA__biogenesis	0.0185185
Drosophila	omegaA	Protein__kinases	Mitochondrial__biogenesis	0.7962963
Drosophila	omegaA	Protein__kinases	Peptidases	0.0185185
Drosophila	omegaA	Protein__kinases	Photosynthesis__proteins	0.9814815
Drosophila	omegaA	Protein__kinases	Proteasome	0.9814815
Drosophila	omegaA	Protein__phosphatases__and__associated__proteins	Amino__acid__related__enzymes	0.9074074
Drosophila	omegaA	Protein__phosphatases__and__associated__proteins	Chaperones__and__folding__catalysts	0.1296296
Drosophila	omegaA	Protein__phosphatases__and__associated__proteins	Chromosome__and__associated__proteins	0.7962963
Drosophila	omegaA	Protein__phosphatases__and__associated__proteins	Cytochrome__P450	0.7592593
Drosophila	omegaA	Protein__phosphatases__and__associated__proteins	Cytoskeleton__proteins	0.1296296
Drosophila	omegaA	Protein__phosphatases__and__associated__proteins	DNA__repair__and__recombination__proteins	0.6481481
Drosophila	omegaA	Protein__phosphatases__and__associated__proteins	DNA__replication__proteins	0.0185185
Drosophila	omegaA	Protein__phosphatases__and__associated__proteins	Exosome	0.0555556
Drosophila	omegaA	Protein__phosphatases__and__associated__proteins	G__protein__coupled__receptors	0.8333333
Drosophila	omegaA	Protein__phosphatases__and__associated__proteins	Glycosyltransferases	0.0555556
Drosophila	omegaA	Protein__phosphatases__and__associated__proteins	Ion__channels	0.2037037
Drosophila	omegaA	Protein__phosphatases__and__associated__proteins	Lipid__biosynthesis__proteins	0.7962963
Drosophila	omegaA	Protein__phosphatases__and__associated__proteins	Membrane__trafficking	0.0925926
Drosophila	omegaA	Protein__phosphatases__and__associated__proteins	Messenger__RNA__biogenesis	0.0555556
Drosophila	omegaA	Protein__phosphatases__and__associated__proteins	Mitochondrial__biogenesis	0.2777778
Drosophila	omegaA	Protein__phosphatases__and__associated__proteins	Peptidases	0.4259259
Drosophila	omegaA	Protein__phosphatases__and__associated__proteins	Photosynthesis__proteins	0.4259259
Drosophila	omegaA	Protein__phosphatases__and__associated__proteins	Proteasome	0.3518519
Drosophila	omegaA	Protein__phosphatases__and__associated__proteins	Protein__kinases	0.2407407
Drosophila	omegaA	Ribosome	Amino__acid__related__enzymes	0.6481481
Drosophila	omegaA	Ribosome	Chaperones__and__folding__catalysts	0.8333333
Drosophila	omegaA	Ribosome	Chromosome__and__associated__proteins	0.2407407
Drosophila	omegaA	Ribosome	Cytochrome__P450	0.3148148
Drosophila	omegaA	Ribosome	Cytoskeleton__proteins	0.7222222
Drosophila	omegaA	Ribosome	DNA__repair__and__recombination__proteins	0.3518519
Drosophila	omegaA	Ribosome	DNA__replication__proteins	0.2037037
Drosophila	omegaA	Ribosome	Exosome	0.6111111
Drosophila	omegaA	Ribosome	G__protein__coupled__receptors	0.3148148
Drosophila	omegaA	Ribosome	Glycosyltransferases	0.3518519
Drosophila	omegaA	Ribosome	Ion__channels	0.8703704
Drosophila	omegaA	Ribosome	Lipid__biosynthesis__proteins	0.2407407
Drosophila	omegaA	Ribosome	Membrane__trafficking	0.9074074
Drosophila	omegaA	Ribosome	Messenger__RNA__biogenesis	0.0185185
Drosophila	omegaA	Ribosome	Mitochondrial__biogenesis	0.9444444
Drosophila	omegaA	Ribosome	Peptidases	0.0185185
Drosophila	omegaA	Ribosome	Photosynthesis__proteins	0.7592593
Drosophila	omegaA	Ribosome	Proteasome	0.7592593
Drosophila	omegaA	Ribosome	Protein__kinases	0.7962963
Drosophila	omegaA	Ribosome	Protein__phosphatases__and__associated__proteins	0.2777778
Drosophila	omegaA	Ribosome__biogenesis	Amino__acid__related__enzymes	0.6851852
Drosophila	omegaA	Ribosome__biogenesis	Chaperones__and__folding__catalysts	0.0925926
Drosophila	omegaA	Ribosome__biogenesis	Chromosome__and__associated__proteins	0.3888889
Drosophila	omegaA	Ribosome__biogenesis	Cytochrome__P450	0.5000000
Drosophila	omegaA	Ribosome__biogenesis	Cytoskeleton__proteins	0.0185185
Drosophila	omegaA	Ribosome__biogenesis	DNA__repair__and__recombination__proteins	0.1666667

(continued)

species	estimate	var1	var2	p.value
Drosophila	omegaA	Ribosome__biogenesis	DNA__replication__proteins	0.0185185
Drosophila	omegaA	Ribosome__biogenesis	Exosome	0.0185185
Drosophila	omegaA	Ribosome__biogenesis	G__protein__coupled__receptors	0.7222222
Drosophila	omegaA	Ribosome__biogenesis	Glycosyltransferases	0.0185185
Drosophila	omegaA	Ribosome__biogenesis	Ion__channels	0.0555556
Drosophila	omegaA	Ribosome__biogenesis	Lipid__biosynthesis__proteins	0.3888889
Drosophila	omegaA	Ribosome__biogenesis	Membrane__trafficking	0.0185185
Drosophila	omegaA	Ribosome__biogenesis	Messenger__RNA__biogenesis	0.0555556
Drosophila	omegaA	Ribosome__biogenesis	Mitochondrial__biogenesis	0.0185185
Drosophila	omegaA	Ribosome__biogenesis	Peptidases	0.4259259
Drosophila	omegaA	Ribosome__biogenesis	Photosynthesis__proteins	0.0925926
Drosophila	omegaA	Ribosome__biogenesis	Proteasome	0.0925926
Drosophila	omegaA	Ribosome__biogenesis	Protein__kinases	0.0555556
Drosophila	omegaA	Ribosome__biogenesis	Protein__phosphatases__and__associated__proteins	0.6481481
Drosophila	omegaA	Ribosome__biogenesis	Ribosome	0.0555556
Drosophila	omegaA	Spliceosome	Amino__acid__related__enzymes	0.7222222
Drosophila	omegaA	Spliceosome	Chaperones__and__folding__catalysts	0.6111111
Drosophila	omegaA	Spliceosome	Chromosome__and__associated__proteins	0.2037037
Drosophila	omegaA	Spliceosome	Cytochrome__P450	0.3148148
Drosophila	omegaA	Spliceosome	Cytoskeleton__proteins	0.3148148
Drosophila	omegaA	Spliceosome	DNA__repair__and__recombination__proteins	0.3888889
Drosophila	omegaA	Spliceosome	DNA__replication__proteins	0.0185185
Drosophila	omegaA	Spliceosome	Exosome	0.1666667
Drosophila	omegaA	Spliceosome	G__protein__coupled__receptors	0.5740741
Drosophila	omegaA	Spliceosome	Glycosyltransferases	0.0185185
Drosophila	omegaA	Spliceosome	Ion__channels	0.8333333
Drosophila	omegaA	Spliceosome	Lipid__biosynthesis__proteins	0.3148148
Drosophila	omegaA	Spliceosome	Membrane__trafficking	0.6111111
Drosophila	omegaA	Spliceosome	Messenger__RNA__biogenesis	0.0185185
Drosophila	omegaA	Spliceosome	Mitochondrial__biogenesis	0.8333333
Drosophila	omegaA	Spliceosome	Peptidases	0.0185185
Drosophila	omegaA	Spliceosome	Photosynthesis__proteins	0.9444444
Drosophila	omegaA	Spliceosome	Proteasome	0.9814815
Drosophila	omegaA	Spliceosome	Protein__kinases	0.9074074
Drosophila	omegaA	Spliceosome	Protein__phosphatases__and__associated__proteins	0.2037037
Drosophila	omegaA	Spliceosome	Ribosome	0.9814815
Drosophila	omegaA	Spliceosome	Ribosome__biogenesis	0.0555556
Drosophila	omegaA	Transcription__factors	Amino__acid__related__enzymes	0.7222222
Drosophila	omegaA	Transcription__factors	Chaperones__and__folding__catalysts	0.7222222
Drosophila	omegaA	Transcription__factors	Chromosome__and__associated__proteins	0.0925926
Drosophila	omegaA	Transcription__factors	Cytochrome__P450	0.2037037
Drosophila	omegaA	Transcription__factors	Cytoskeleton__proteins	0.3888889
Drosophila	omegaA	Transcription__factors	DNA__repair__and__recombination__proteins	0.2407407
Drosophila	omegaA	Transcription__factors	DNA__replication__proteins	0.0185185
Drosophila	omegaA	Transcription__factors	Exosome	0.2407407
Drosophila	omegaA	Transcription__factors	G__protein__coupled__receptors	0.3518519
Drosophila	omegaA	Transcription__factors	Glycosyltransferases	0.1296296
Drosophila	omegaA	Transcription__factors	Ion__channels	0.9444444
Drosophila	omegaA	Transcription__factors	Lipid__biosynthesis__proteins	0.2407407
Drosophila	omegaA	Transcription__factors	Membrane__trafficking	0.7592593
Drosophila	omegaA	Transcription__factors	Messenger__RNA__biogenesis	0.0185185
Drosophila	omegaA	Transcription__factors	Mitochondrial__biogenesis	0.9444444
Drosophila	omegaA	Transcription__factors	Peptidases	0.0185185
Drosophila	omegaA	Transcription__factors	Photosynthesis__proteins	0.8333333
Drosophila	omegaA	Transcription__factors	Proteasome	0.8333333
Drosophila	omegaA	Transcription__factors	Protein__kinases	0.4629630
Drosophila	omegaA	Transcription__factors	Protein__phosphatases__and__associated__proteins	0.2037037
Drosophila	omegaA	Transcription__factors	Ribosome	0.9444444
Drosophila	omegaA	Transcription__factors	Ribosome__biogenesis	0.0185185
Drosophila	omegaA	Transcription__factors	Spliceosome	0.9444444
Drosophila	omegaA	Transcription__machinery	Amino__acid__related__enzymes	0.7592593
Drosophila	omegaA	Transcription__machinery	Chaperones__and__folding__catalysts	0.4259259

(continued)

species	estimate	var1	var2	p.value
Drosophila	omegaA	Transcription__machinery	Chromosome__and__associated__proteins	0.3888889
Drosophila	omegaA	Transcription__machinery	Cytochrome__P450	0.5740741
Drosophila	omegaA	Transcription__machinery	Cytoskeleton__proteins	0.2037037
Drosophila	omegaA	Transcription__machinery	DNA__repair__and__recombination__proteins	0.6481481
Drosophila	omegaA	Transcription__machinery	DNA__replication__proteins	0.0185185
Drosophila	omegaA	Transcription__machinery	Exosome	0.0185185
Drosophila	omegaA	Transcription__machinery	G__protein__coupled__receptors	0.5370370
Drosophila	omegaA	Transcription__machinery	Glycosyltransferases	0.0185185
Drosophila	omegaA	Transcription__machinery	Ion__channels	0.3888889
Drosophila	omegaA	Transcription__machinery	Lipid__biosynthesis__proteins	0.6111111
Drosophila	omegaA	Transcription__machinery	Membrane__trafficking	0.1666667
Drosophila	omegaA	Transcription__machinery	Messenger__RNA__biogenesis	0.0185185
Drosophila	omegaA	Transcription__machinery	Mitochondrial__biogenesis	0.4629630
Drosophila	omegaA	Transcription__machinery	Peptidases	0.0925926
Drosophila	omegaA	Transcription__machinery	Photosynthesis__proteins	0.8333333
Drosophila	omegaA	Transcription__machinery	Proteasome	0.8703704
Drosophila	omegaA	Transcription__machinery	Protein__kinases	0.5000000
Drosophila	omegaA	Transcription__machinery	Protein__phosphatases__and__associated__proteins	0.4629630
Drosophila	omegaA	Transcription__machinery	Ribosome	0.6111111
Drosophila	omegaA	Transcription__machinery	Ribosome__biogenesis	0.0925926
Drosophila	omegaA	Transcription__machinery	Spliceosome	0.6851852
Drosophila	omegaA	Transcription__machinery	Transcription__factors	0.2777778
Drosophila	omegaA	Transfer__RNA__biogenesis	Amino__acid__related__enzymes	0.7222222
Drosophila	omegaA	Transfer__RNA__biogenesis	Chaperones__and__folding__catalysts	0.8703704
Drosophila	omegaA	Transfer__RNA__biogenesis	Chromosome__and__associated__proteins	0.0555556
Drosophila	omegaA	Transfer__RNA__biogenesis	Cytochrome__P450	0.1666667
Drosophila	omegaA	Transfer__RNA__biogenesis	Cytoskeleton__proteins	0.5000000
Drosophila	omegaA	Transfer__RNA__biogenesis	DNA__repair__and__recombination__proteins	0.2407407
Drosophila	omegaA	Transfer__RNA__biogenesis	DNA__replication__proteins	0.0925926
Drosophila	omegaA	Transfer__RNA__biogenesis	Exosome	0.2407407
Drosophila	omegaA	Transfer__RNA__biogenesis	G__protein__coupled__receptors	0.2037037
Drosophila	omegaA	Transfer__RNA__biogenesis	Glycosyltransferases	0.1666667
Drosophila	omegaA	Transfer__RNA__biogenesis	Ion__channels	0.6851852
Drosophila	omegaA	Transfer__RNA__biogenesis	Lipid__biosynthesis__proteins	0.1666667
Drosophila	omegaA	Transfer__RNA__biogenesis	Membrane__trafficking	0.9074074
Drosophila	omegaA	Transfer__RNA__biogenesis	Messenger__RNA__biogenesis	0.0185185
Drosophila	omegaA	Transfer__RNA__biogenesis	Mitochondrial__biogenesis	0.9444444
Drosophila	omegaA	Transfer__RNA__biogenesis	Peptidases	0.0185185
Drosophila	omegaA	Transfer__RNA__biogenesis	Photosynthesis__proteins	0.7962963
Drosophila	omegaA	Transfer__RNA__biogenesis	Proteasome	0.6481481
Drosophila	omegaA	Transfer__RNA__biogenesis	Protein__kinases	0.3148148
Drosophila	omegaA	Transfer__RNA__biogenesis	Protein__phosphatases__and__associated__proteins	0.2037037
Drosophila	omegaA	Transfer__RNA__biogenesis	Ribosome	0.9814815
Drosophila	omegaA	Transfer__RNA__biogenesis	Ribosome__biogenesis	0.0185185
Drosophila	omegaA	Transfer__RNA__biogenesis	Spliceosome	0.6481481
Drosophila	omegaA	Transfer__RNA__biogenesis	Transcription__factors	0.7962963
Drosophila	omegaA	Transfer__RNA__biogenesis	Transcription__machinery	0.2407407
Drosophila	omegaA	Translation__factors	Amino__acid__related__enzymes	0.5740741
Drosophila	omegaA	Translation__factors	Chaperones__and__folding__catalysts	0.3148148
Drosophila	omegaA	Translation__factors	Chromosome__and__associated__proteins	0.9444444
Drosophila	omegaA	Translation__factors	Cytochrome__P450	0.8333333
Drosophila	omegaA	Translation__factors	Cytoskeleton__proteins	0.0925926
Drosophila	omegaA	Translation__factors	DNA__repair__and__recombination__proteins	0.7592593
Drosophila	omegaA	Translation__factors	DNA__replication__proteins	0.0185185
Drosophila	omegaA	Translation__factors	Exosome	0.0925926
Drosophila	omegaA	Translation__factors	G__protein__coupled__receptors	0.8703704
Drosophila	omegaA	Translation__factors	Glycosyltransferases	0.0555556
Drosophila	omegaA	Translation__factors	Ion__channels	0.3518519
Drosophila	omegaA	Translation__factors	Lipid__biosynthesis__proteins	0.6851852
Drosophila	omegaA	Translation__factors	Membrane__trafficking	0.2407407
Drosophila	omegaA	Translation__factors	Messenger__RNA__biogenesis	0.3888889

(continued)

species	estimate	var1	var2	p.value
Drosophila	omegaA	Translation__factors	Mitochondrial__biogenesis	0.2777778
Drosophila	omegaA	Translation__factors	Peptidases	0.5000000
Drosophila	omegaA	Translation__factors	Photosynthesis__proteins	0.2777778
Drosophila	omegaA	Translation__factors	Proteasome	0.3148148
Drosophila	omegaA	Translation__factors	Protein__kinases	0.5000000
Drosophila	omegaA	Translation__factors	Protein__phosphatases__and__associated__proteins	0.9074074
Drosophila	omegaA	Translation__factors	Ribosome	0.2407407
Drosophila	omegaA	Translation__factors	Ribosome__biogenesis	0.8703704
Drosophila	omegaA	Translation__factors	Spliceosome	0.4259259
Drosophila	omegaA	Translation__factors	Transcription__factors	0.3888889
Drosophila	omegaA	Translation__factors	Transcription__machinery	0.6111111
Drosophila	omegaA	Translation__factors	Transfer__RNA__biogenesis	0.2777778
Drosophila	omegaA	Transporters	Amino__acid__related__enzymes	0.7222222
Drosophila	omegaA	Transporters	Chaperones__and__folding__catalysts	0.8333333
Drosophila	omegaA	Transporters	Chromosome__and__associated__proteins	0.0925926
Drosophila	omegaA	Transporters	Cytochrome__P450	0.2407407
Drosophila	omegaA	Transporters	Cytoskeleton__proteins	0.4629630
Drosophila	omegaA	Transporters	DNA__repair__and__recombination__proteins	0.2037037
Drosophila	omegaA	Transporters	DNA__replication__proteins	0.0185185
Drosophila	omegaA	Transporters	Exosome	0.2407407
Drosophila	omegaA	Transporters	G__protein__coupled__receptors	0.2407407
Drosophila	omegaA	Transporters	Glycosyltransferases	0.0925926
Drosophila	omegaA	Transporters	Ion__channels	0.7592593
Drosophila	omegaA	Transporters	Lipid__biosynthesis__proteins	0.1666667
Drosophila	omegaA	Transporters	Membrane__trafficking	0.9814815
Drosophila	omegaA	Transporters	Messenger__RNA__biogenesis	0.0185185
Drosophila	omegaA	Transporters	Mitochondrial__biogenesis	0.9444444
Drosophila	omegaA	Transporters	Peptidases	0.0185185
Drosophila	omegaA	Transporters	Photosynthesis__proteins	0.7222222
Drosophila	omegaA	Transporters	Proteasome	0.7592593
Drosophila	omegaA	Transporters	Protein__kinases	0.4259259
Drosophila	omegaA	Transporters	Protein__phosphatases__and__associated__proteins	0.0555556
Drosophila	omegaA	Transporters	Ribosome	0.9444444
Drosophila	omegaA	Transporters	Ribosome__biogenesis	0.0555556
Drosophila	omegaA	Transporters	Spliceosome	0.6851852
Drosophila	omegaA	Transporters	Transcription__factors	0.6111111
Drosophila	omegaA	Transporters	Transcription__machinery	0.2777778
Drosophila	omegaA	Transporters	Transfer__RNA__biogenesis	0.9814815
Drosophila	omegaA	Transporters	Translation__factors	0.2407407
Drosophila	omegaA	Ubiquitin__system	Amino__acid__related__enzymes	0.8333333
Drosophila	omegaA	Ubiquitin__system	Chaperones__and__folding__catalysts	0.3148148
Drosophila	omegaA	Ubiquitin__system	Chromosome__and__associated__proteins	0.5370370
Drosophila	omegaA	Ubiquitin__system	Cytochrome__P450	0.7592593
Drosophila	omegaA	Ubiquitin__system	Cytoskeleton__proteins	0.1296296
Drosophila	omegaA	Ubiquitin__system	DNA__repair__and__recombination__proteins	0.7592593
Drosophila	omegaA	Ubiquitin__system	DNA__replication__proteins	0.0185185
Drosophila	omegaA	Ubiquitin__system	Exosome	0.0185185
Drosophila	omegaA	Ubiquitin__system	G__protein__coupled__receptors	0.9814815
Drosophila	omegaA	Ubiquitin__system	Glycosyltransferases	0.0185185
Drosophila	omegaA	Ubiquitin__system	Ion__channels	0.1296296
Drosophila	omegaA	Ubiquitin__system	Lipid__biosynthesis__proteins	0.9444444
Drosophila	omegaA	Ubiquitin__system	Membrane__trafficking	0.0555556
Drosophila	omegaA	Ubiquitin__system	Messenger__RNA__biogenesis	0.0185185
Drosophila	omegaA	Ubiquitin__system	Mitochondrial__biogenesis	0.4629630
Drosophila	omegaA	Ubiquitin__system	Peptidases	0.1296296
Drosophila	omegaA	Ubiquitin__system	Photosynthesis__proteins	0.7222222
Drosophila	omegaA	Ubiquitin__system	Proteasome	0.6481481
Drosophila	omegaA	Ubiquitin__system	Protein__kinases	0.1666667
Drosophila	omegaA	Ubiquitin__system	Protein__phosphatases__and__associated__proteins	0.5740741
Drosophila	omegaA	Ubiquitin__system	Ribosome	0.3148148
Drosophila	omegaA	Ubiquitin__system	Ribosome__biogenesis	0.2037037
Drosophila	omegaA	Ubiquitin__system	Spliceosome	0.3148148

(continued)

species	estimate	var1	var2	p.value
Drosophila	omegaA	Ubiquitin_system	Transcription_factors	0.1296296
Drosophila	omegaA	Ubiquitin_system	Transcription_machinery	0.5000000
Drosophila	omegaA	Ubiquitin_system	Transfer_RNA_biogenesis	0.1666667
Drosophila	omegaA	Ubiquitin_system	Translation_factors	0.7222222
Drosophila	omegaA	Ubiquitin_system	Transporters	0.0185185

The next chunk presents the correlation performed between the categories of the two species.

```
# Will do this correlation for values of omegaA only
omegaA.arab <- subset(stat.arab, stat.arab$variable == "omega[a]")
omegaA.arab <- omegaA.arab[,-3] # removing the 'species' column
colnames(omegaA.arab) <- c("variable", "FunctionalClass",
                           "value.mean.arab", "value.sd.arab")

omegaA.dmel <- subset(stat.dmel, stat.dmel$variable == "omega[a]")
omegaA.dmel <- omegaA.dmel[,-3] # removing the 'species' column
colnames(omegaA.dmel) <- c("variable", "FunctionalClass",
                           "value.mean.dmel", "value.sd.dmel")

cor.kegg <- merge(omegaA.arab, omegaA.dmel, by = c("variable",
                                                   "FunctionalClass")) # 21 categories

plot.cor <- ggplot(cor.kegg, aes(value.mean.arab, value.mean.dmel,
                                 label = FunctionalClass)) +
  geom_point() +
  geom_smooth(method = "lm", se = TRUE) +
  xlab(expression(paste("Mean",~omega[a],~italic(A.thaliana), sep = ""))) +
  ylab(expression(paste("Mean",~omega[a],~italic(D.melanogaster), sep = ""))) +
  theme_bw() +
  theme.plot()
plot.cor
```

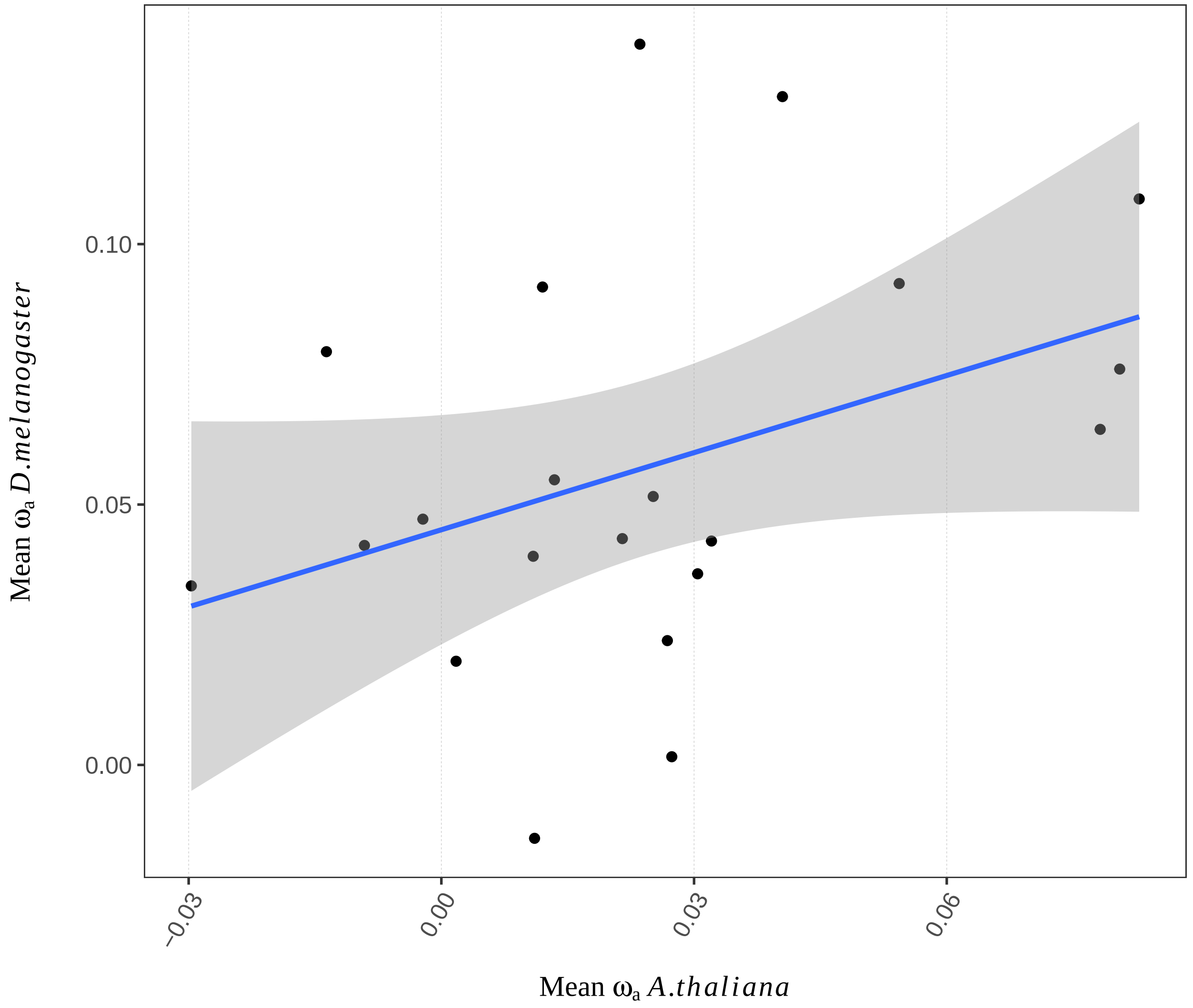


```
# outliers: mRNA biogenesis (row 9) and Glycositransferases (row 7)
sub.cor <- cor.kegg[-c(7,9),]
plot.subcor <- ggplot(sub.cor, aes(value.mean.arab, value.mean.dmel,
                                   label = FunctionalClass)) +
  geom_point() +
  geom_smooth(method = "lm", se = TRUE) +
  xlab(expression(paste("Mean",~omega[a],~italic(A.thaliana), sep = ""))) +
  ylab(expression(paste("Mean",~omega[a],~italic(D.melanogaster), sep = ""))) +
  theme_bw() +
  theme.plot()
plot.subcor
```

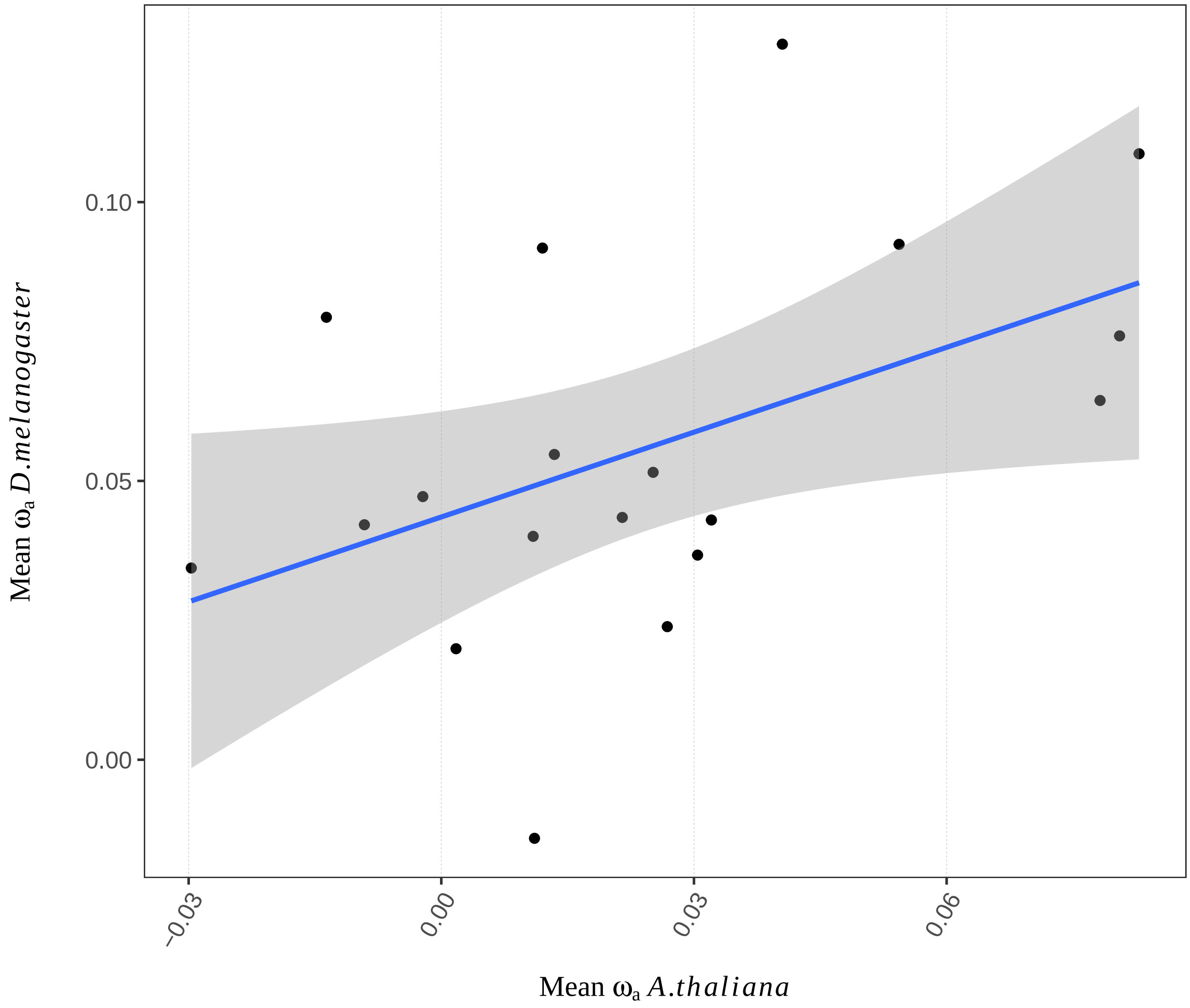

Statistical tests performed on both correlations:

```
# full data:
cor.all <- cor.test(~value.mean.arab+value.mean.dmel,cor.kegg, method = "kendall")
Kendall.tau <- cor.all$estimate
p.value <- cor.all$p.value
stat.all <- data.frame(Kendall.tau, p.value)
stat.all
```

	Kendall.tau	p.value
tau	0.2571429	0.1101277

```
# without outliers
cor.sub <- cor.test(~value.mean.arab+value.mean.dmel,sub.cor, method = "kendall")
Kendall.tau <- cor.sub$estimate
p.value <- cor.sub$p.value
sub.stat <- data.frame(Kendall.tau, p.value)
sub.stat
```

	Kendall.tau	p.value
tau	0.3333333	0.0490493
