# Supplementary material for: The Impact of Protein Architecture on Adaptive Evolution
Source: Mol Biol Evol. 2019 May 30;36(9):2013–28. doi: 10.1093/molbev/msz134 (PMC6735723; doi:10.1093/molbev/msz134)
Supplement: msz134_Supplementary_Data [file msz134_supplementary_data.zip › FileS5.pdf]

Correlation between variables

This is the script all plots and statistical analysis for the correlation between variables, refering to: Intrinsic Disorder/Secondary Structure, RSA/Active Site, RSA/Intrinsic Disorder, RSA/Protein Length and RSA/Secondary structure

For this purpose, two categories of RSA were considered: buried (RSA < 0.05) and exposed (RSA >= 0.05)

A function was created to call all files for each analysis performed. In this respect the order of the files is as follows:
files.correlations[1] <- Intrinsic Residue Disorder and Secondary Structure
files.correlations[2] <- RSA and Active Site
files.correlations[3] <- RSA and Residue Intrinsic Disorder
files.correlations[4] <- RSA and Protein Length
files.correlations[5] <- RSA and Secondary Structure

In these tables var 1 corresponds to the relative solvent accessibility and var 2 corresponds to the respective four different varaibles analysed (active site, disorder, protein length and secondary structure), with the exception of the correlation between intrinsic disorder and secondary structure, for which var1 corresponds to the structural motif and var2 the probability of residue intrinsic disorder.

The first part of the script removes bootstrap replicates for which the fitness effects parameters were not successfully fitted. For this purpose we discard 1% of the values above the maximum and below the minimum of each of the four parameters of fitness effects: Geman.neg, Gshape.neg, Gmean.neg and prop.pos.

```r
setwd("/Users/moutinho/Dropbox/Data/Correlations/")

# Libraries
library(plyr)
library(dplyr)
library(data.table)
library(ggplot2)
library(reshape2)
library(doBy)
library(rlist)
library(stringr)
library(knitr)
library(kableExtra)
#

# calling all output tables
files.correlations <- list.files(".", ".csv")

# reading each table into a list
tbl.list <- lapply(files.correlations, read.table, header = TRUE)

## remove the outliers: 1% of the replicates below the min and
## 1% above the maximum
sub.tbl <- lapply(tbl.list, function(x) {
  ddply(x, c("species", "var1", "var2"), function(x) {
    sum.gmeanNeg <- summary(x$Gmean.neg)
    gmeanNeg.min1 <- as.numeric(sum.gmeanNeg[1]) + 0.01*as.numeric(sum.gmeanNeg[1])
    gmeanNeg.max1 <- as.numeric(sum.gmeanNeg[6]) - 0.01*as.numeric(sum.gmeanNeg[6])
    sum.gshapeNeg <- summary(x$Gshape.neg)
    gshapeNeg.min1 <- as.numeric(sum.gshapeNeg[1]) + 0.01*as.numeric(sum.gshapeNeg[1])
```

```
    gshapeNeg.max1 <- as.numeric(sum.gshapeNeg[6]) - 0.01*as.numeric(sum.gshapeNeg[6])
    sum.gmeanPos <- summary(x$Gmean.pos)
    gmeanPos.min1 <- as.numeric(sum.gmeanPos[1]) + 0.01*as.numeric(sum.gmeanPos[1])
    gmeanPos.max1 <- as.numeric(sum.gmeanPos[6]) - 0.01*as.numeric(sum.gmeanPos[6])
    sum.propPos <- summary(x$prop.pos)
    propPos.min1 <- as.numeric(sum.propPos[1]) + 0.01*as.numeric(sum.propPos[1])
    propPos.max1 <- as.numeric(sum.propPos[6]) - 0.01*as.numeric(sum.propPos[6])
    tbl <- x[!(x$Gmean.neg < gmeanNeg.min1 | x$Gmean.neg > gmeanNeg.max1 &
                 x$Gshape.neg < gshapeNeg.min1 | x$Gshape.neg > gshapeNeg.max1 &
                 x$Gmean.pos < gmeanPos.min1 | x$Gmean.pos > gmeanPos.max1 &
                 x$prop.pos < propPos.min1 | x$prop.pos > propPos.max1),]
  })
})
```

In the next chunk will take only the estimates concerning the rate of adaptive and non-adaptive substitutions, particularly: dnds, omegaNA and omegaA.

```
# In order to keep only the variables that we want to plot:
tbl.rates <- lapply(sub.tbl, function(x) {
  ddply(x, c("var", "species"), function(x) {
    melt(x, id.vars = c("var1", "var2"), measure.vars = c("dnds", "omegaNA", "omegaA"))
  })
})

# function to estimate the mean and satandard deviation
fun <- function(x){
  c(mean=mean(x), sd=sd(x))
}

# applying the above function to each output table for each value of each estimate
# (dnds, omegaA, omegaNA) for each value of the variable being analyzed for each species
tbl.stats <- lapply(tbl.rates, function(x) {
  summaryBy(value ~ variable + var1 + var2 + species + var, data=x, FUN = fun)
})

# to change the estimate name to the respective symbol
tbl.stats[[1]]$variable <- factor(tbl.stats[[1]]$variable,
                                  levels = c("dnds", "omegaNA", "omegaA"))
levels(tbl.stats[[1]]$variable) <- c(expression(omega), expression(omega[na]),
                                   expression(omega[a]))
tbl.stats[[2]]$variable <- factor(tbl.stats[[2]]$variable,
                                  levels = c("dnds", "omegaNA", "omegaA"))
levels(tbl.stats[[2]]$variable) <- c(expression(omega), expression(omega[na]),
                                   expression(omega[a]))
tbl.stats[[3]]$variable <- factor(tbl.stats[[3]]$variable,
                                  levels = c("dnds", "omegaNA", "omegaA"))
levels(tbl.stats[[3]]$variable) <- c(expression(omega), expression(omega[na]),
                                   expression(omega[a]))
tbl.stats[[4]]$variable <- factor(tbl.stats[[4]]$variable,
                                  levels = c("dnds", "omegaNA", "omegaA"))
levels(tbl.stats[[4]]$variable) <- c(expression(omega), expression(omega[na]),
                                   expression(omega[a]))
tbl.stats[[5]]$variable <- factor(tbl.stats[[5]]$variable,
                                  levels = c("dnds", "omegaNA", "omegaA"))
```

```
levels(tbl.stats[[5]]$variable) <- c(expression(omega), expression(omega[na]),
                                     expression(omega[a]))
```

The next chunk describes the script to plot the results of RSA/Active Site and RSA/Secondary Structure

```
# theme of the plot
theme.plot <- function(x) {
  theme(axis.title = element_text(face = "bold", color = "black", size=12,
                                  family = "Times"),
        text = element_text(size=12),
        axis.title.x = element_text(margin = margin(t = 9, r = 10, b = 0, l = 0)),
        axis.title.y = element_text(margin = margin(t = 9, r = 10, b = 0, l = 0)),
        panel.grid.minor=element_blank(),
        panel.grid.major = element_line(colour = "grey", linetype = "dashed", size = 0.1),
        panel.grid.major.y=element_blank(),
        strip.text.y = element_blank(),
        axis.text.x = element_text(angle = 60, hjust = 1),
        strip.background.x = element_rect(colour = "grey", fill = "gray92"),
        legend.text = element_text(size = 7),
        legend.title = element_text(size = 9, face = "bold"))
}

# plotting each of the output tables
plot.correlations.dis <- lapply(tbl.stats[c(2,5)], function(x) {
  ggplot(x, aes(x = var2, y = value.mean, fill = var1)) +
    geom_point(size=0.5, aes(fill = var1, col = var1)) +
    geom_errorbar(aes(ymin=value.mean + 1.96*value.sd,
                      ymax=value.mean - 1.96*value.sd, col = var1), width = .2) +
    facet_grid(species~variable, scales = "free_x", labeller = label_parsed) +
    ylab("") +
    xlab(as.character(x$var)) +
    scale_fill_grey() +
    scale_color_grey() +
    theme_bw() +
    theme.plot()
})

plot.correlations.dis
```

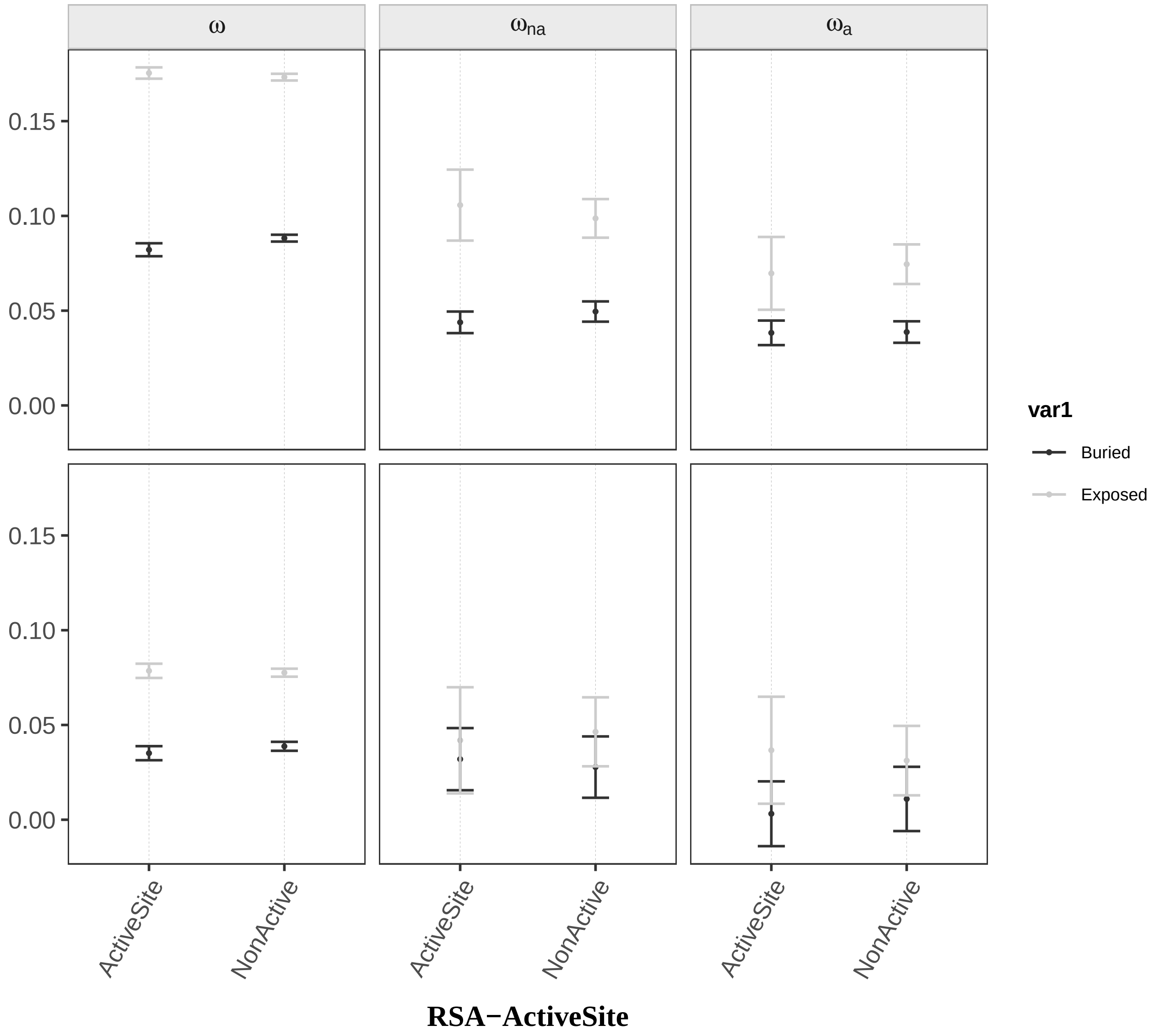
ω
ω_{na}
ω_a
0.15
0.10
0.05
0.00
0.15
0.10
0.05
0.00
ActiveSite
NonActive
ActiveSite
NonActive
ActiveSite
NonActive
RSA−ActiveSite
var1
Buried
Exposed

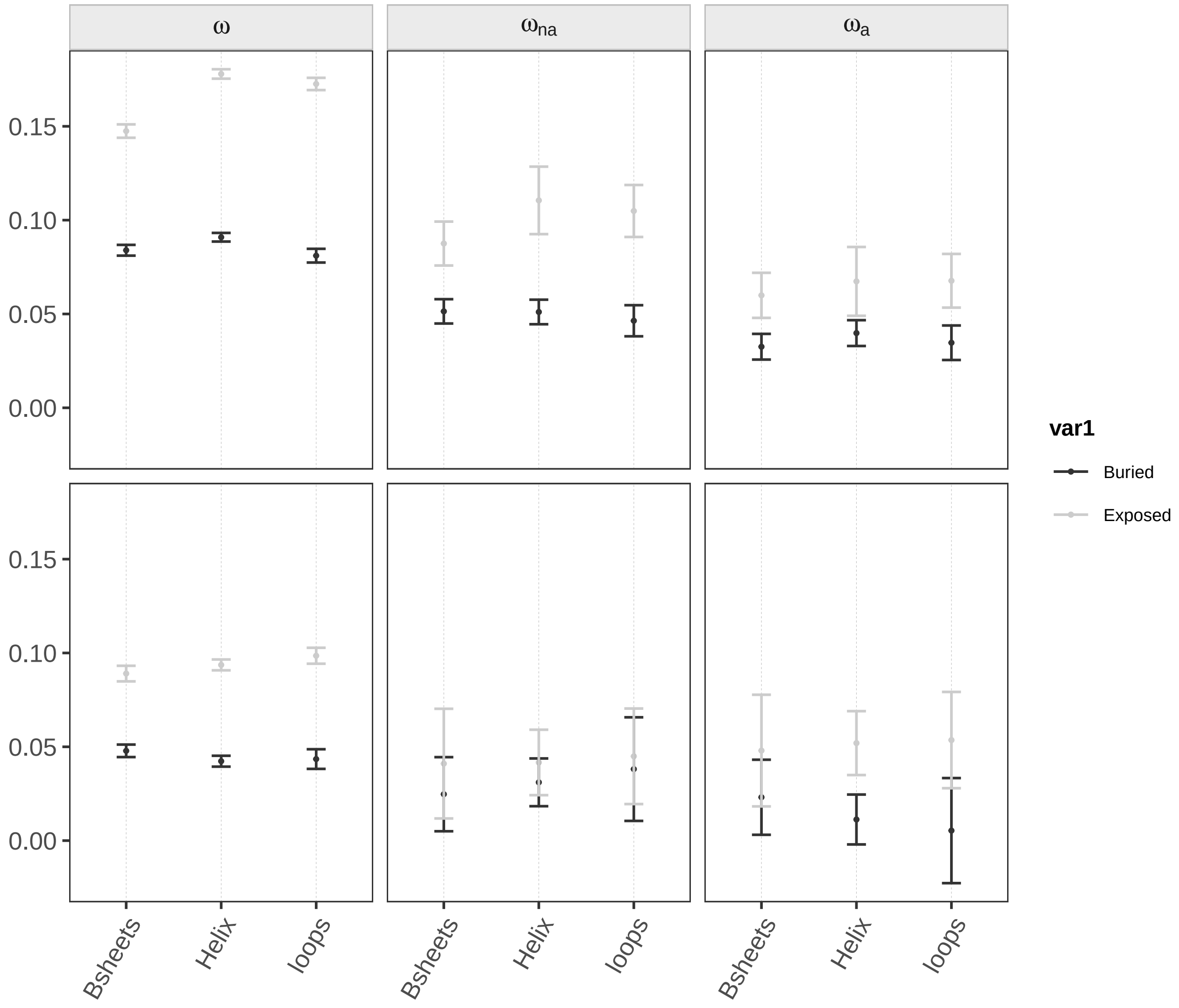

RSA−SecondaryStructure

The next chunk describes the statistical analysis performed for the correlation of RSA/Active Site and RSA/Secondary Structure

```
####
#### RSA/Active Site
####

# getting the difference between the mean values of each category (for the table in the main file)
dif.tbl <- ddply(tbl.stats[[2]], c("variable", "species", "var1"), function(x) {
  dif <- as.numeric(x$value.mean[x$var2 == "ActiveSite"] -
                             x$value.mean[x$var2 == "NonActive"])
  data.frame(dif)
})
kable(dif.tbl, caption = "Difference between the mean values for each category")
```

```
# checking the number of replicates per var.value, variable
# and species after removing 1% outliers
ps.nrep <- ddply(tbl.rates[[2]], c("species", "variable", "var1", "var2"),
                 function(x) {
```

Table 1: Difference between the mean values for each category

variable	species	var1	dif
omega	Arabidopsis	Buried	-0.0061129
omega	Arabidopsis	Exposed	0.0021660
omega	Drosophila	Buried	-0.0036265
omega	Drosophila	Exposed	0.0009797
omega[na]	Arabidopsis	Buried	-0.0056944
omega[na]	Arabidopsis	Exposed	0.0069991
omega[na]	Drosophila	Buried	0.0041936
omega[na]	Drosophila	Exposed	-0.0044935
omega[a]	Arabidopsis	Buried	-0.0004186
omega[a]	Arabidopsis	Exposed	-0.0048330
omega[a]	Drosophila	Buried	-0.0078201
omega[a]	Drosophila	Exposed	0.0054731

```
    nrep <- nrow(x)
    data.frame(nrep)
})

# take the minimum number of replicates between categories
min.nrep <- ddply(ps.nrep, c("species", "var1", "var2"),
                  function(x) {
  min.n <- min(x$nrep)
  data.frame(min.n)
})
min.nrep
```

species	var1	var2	min.n
Arabidopsis	Buried	ActiveSite	97
Arabidopsis	Buried	NonActive	97
Arabidopsis	Exposed	ActiveSite	96
Arabidopsis	Exposed	NonActive	98
Drosophila	Buried	ActiveSite	98
Drosophila	Buried	NonActive	97
Drosophila	Exposed	ActiveSite	97
Drosophila	Exposed	NonActive	98

```
# 96 replicates Arabidopsis
nrep.arab <- subset(tbl.rates[[2]], tbl.rates[[2]]$species == "Arabidopsis")
tbl.arab <- ddply(nrep.arab, c("species", "var1", "var2", "variable"),
                  function(x) {
  x[sample(nrow(x), 96), ]
})

# 97 replicates for Drosophila
nrep.dmel <- subset(tbl.rates[[2]], tbl.rates[[2]]$species == "Drosophila")
tbl.dmel <- ddply(nrep.dmel, c("species", "var1", "var2", "variable"),
                  function(x) {
  x[sample(nrow(x), 97), ]
})

dat.ps <- rbind(tbl.arab, tbl.dmel)
```

```r
# function to split the tables by the name of each variable
ps.split <- by(dat.ps, dat.ps[,c("var2")], function(y) y)

# to change the column name "value" to the respective id of the category
ps.value <- lapply(ps.split, function(x) {
  tbl <- data.frame(x)
  colnames(tbl)[6] <- as.character(unique(tbl$var2))
  tbl <- tbl[,-4]
  return(tbl)
})

# binding all values as columns
names.vars <- ps.value[[1]][,1:4]

tbl.estimate <- lapply(ps.value, function(x) {
  val <- x[,5]
})
tbl.ps <- list.cbind(tbl.estimate)
tbl.ps <- as.data.frame(tbl.ps)
tbl.ps <- cbind(names.vars, tbl.ps)
colnames(tbl.ps)[4] <- "estimate"

# now lets estimate the differences between the two variables
ps.dif <- ddply(tbl.ps, c("estimate", "species", "var1"), function(x){
  dif <- x$ActiveSite - x$NonActive
  tbl <- data.frame(dif)
})


## getting the p-value for each difference
## Arabidopsis
arab.dif <- subset(ps.dif, ps.dif$species == "Arabidopsis")
nboots <- 100
arab.p <- list()
for (i in 1:nboots) {
  arab.p[[i]] <- ddply(arab.dif, c("estimate", "species", "var1"),
                       function(x, N=96){
    c <- as.numeric(nrow(x[x$dif < 0,]))
    c2 <- as.numeric(nrow(x[x$dif > 0,]))
    m <- min(c, c2)
    p <- (2*m+1)/(N+1)
    tbl <- data.frame(m, p)
  })
}

arab.p.tbl <- rbindlist(arab.p)
arab.pvalue <- ddply(arab.p.tbl, c("estimate", "species", "var1"),
                     function(x) {
  p.value <- min(x$p)
  data.frame(p.value)
})

# showing the table
```

Table 2: Statistics for RSA/Active Site

estimate	species	var1	p.value
dnds	Arabidopsis	Buried	0.0103093
dnds	Arabidopsis	Exposed	0.1958763
omegaNA	Arabidopsis	Buried	0.1546392
omegaNA	Arabidopsis	Exposed	0.6288660
omegaA	Arabidopsis	Buried	0.8762887
omegaA	Arabidopsis	Exposed	0.6907216

Table 3: Statistics for RSA/Active Site

estimate	species	var1	p.value
dnds	Drosophila	Buried	0.1122449
dnds	Drosophila	Exposed	0.6224490
omegaNA	Drosophila	Buried	0.7857143
omegaNA	Drosophila	Exposed	0.7653061
omegaA	Drosophila	Buried	0.4591837
omegaA	Drosophila	Exposed	0.8265306

```
kable(arab.pvalue, caption = "Statistics for RSA/Active Site")
```

```
## Drosophila
dmel.dif <- subset(ps.dif, ps.dif$species == "Drosophila")
nboots <- 100
dmel.p <- list()
for (i in 1:nboots) {
  dmel.p[[i]] <- ddply(dmel.dif, c("estimate", "species", "var1"),
                      function(x, N=97){
    c <- as.numeric(nrow(x[x$dif < 0,]))
    c2 <- as.numeric(nrow(x[x$dif > 0,]))
    m <- min(c, c2)
    p <- (2*m+1)/(N+1)
    tbl <- data.frame(m, p)
  })
}
dmel.p.tbl <- rbindlist(dmel.p)
dmel.pvalue <- ddply(dmel.p.tbl, c("estimate", "species", "var1"),
                     function(x) {
  p.value <- min(x$p)
  data.frame(p.value)
})

# showing the table
kable(dmel.pvalue, caption = "Statistics for RSA/Active Site")
```

```
####
#### RSA/Secondary Structure
####

# getting the difference between the mean values of each category (for the table in the main file)
dif.ss <- ddply(tbl.stats[[5]], c("variable", "species", "var1"), function(x) {
```

Table 4: Difference between the mean values for each category

variable	species	var1	bsheets.helix	bsheets.loops	helix.loops
omega	Arabidopsis	Buried	-0.0069399	0.0028876	0.0098275
omega	Arabidopsis	Exposed	-0.0304287	-0.0251116	0.0053171
omega	Drosophila	Buried	0.0055311	0.0043926	-0.0011385
omega	Drosophila	Exposed	-0.0046140	-0.0095011	-0.0048871
omega[na]	Arabidopsis	Buried	0.0003321	0.0050087	0.0046766
omega[na]	Arabidopsis	Exposed	-0.0229944	-0.0173457	0.0056487
omega[na]	Drosophila	Buried	-0.0063247	-0.0133757	-0.0070509
omega[na]	Drosophila	Exposed	-0.0006284	-0.0039051	-0.0032767
omega[a]	Arabidopsis	Buried	-0.0072720	-0.0021211	0.0051509
omega[a]	Arabidopsis	Exposed	-0.0074344	-0.0077660	-0.0003316
omega[a]	Drosophila	Buried	0.0118559	0.0177682	0.0059124
omega[a]	Drosophila	Exposed	-0.0039856	-0.0055960	-0.0016104

```r
  bsheets.loops <- as.numeric(x$value.mean[x$var2 == "Bsheets"] -
                      x$value.mean[x$var2 == "loops"])
  helix.loops <- as.numeric(x$value.mean[x$var2 == "Helix"] -
                             x$value.mean[x$var2 == "loops"])
  bsheets.helix <- as.numeric(x$value.mean[x$var2 == "Bsheets"] -
                                   x$value.mean[x$var2 == "Helix"])
  data.frame(bsheets.helix, bsheets.loops, helix.loops)
})
kable(dif.ss, caption = "Difference between the mean values for each category")
```

```r
# checking the number of replicates per var.value, variable
# and species after removing 1% outliers
SS.nrep <- ddply(tbl.rates[[5]], c("species", "variable", "var1", "var2"),
                 function(x) {
    nrep <- nrow(x)
    data.frame(nrep)
})

# take the minimum number of replicates between categories
min.nrep <- ddply(SS.nrep, c("species", "var1", "var2"), function(x) {
  min.n <- min(x$nrep)
  data.frame(min.n)
})
min.nrep
```

species	var1	var2	min.n
Arabidopsis	Buried	Bsheets	98
Arabidopsis	Buried	Helix	95
Arabidopsis	Buried	loops	97
Arabidopsis	Exposed	Bsheets	97
Arabidopsis	Exposed	Helix	98
Arabidopsis	Exposed	loops	97
Drosophila	Buried	Bsheets	96
Drosophila	Buried	Helix	97
Drosophila	Buried	loops	97
Drosophila	Exposed	Bsheets	99
Drosophila	Exposed	Helix	98
Drosophila	Exposed	loops	99

```
# 95 replicates Arabidopsis
nrep.arab <- subset(tbl.rates[[5]], tbl.rates[[5]]$species == "Arabidopsis")
tbl.arab <- ddply(nrep.arab, c("species", "var1", "var2", "variable"),
                  function(x) {
  x[sample(nrow(x), 95), ]
})

# 96 replicates for Drosophila
nrep.dmel <- subset(tbl.rates[[5]], tbl.rates[[5]]$species == "Drosophila")
tbl.dmel <- ddply(nrep.dmel, c("species", "var1", "var2", "variable"),
                  function(x) {
  x[sample(nrow(x), 96), ]
})

dat.SS <- rbind(tbl.arab, tbl.dmel)

# function to split the tables by the name of each variable
SS.split <- by(dat.SS, dat.SS[,c("var2")], function(y) y)

# to change the column name "value" to the respective id of the category
SS.value <- lapply(SS.split, function(x) {
  tbl <- data.frame(x)
  colnames(tbl)[6] <- as.character(unique(tbl$var2))
  tbl <- tbl[,-4]
  return(tbl)
})

# binding all values as columns
names.vars <- SS.value[[1]][,1:4]

tbl.estimate <- lapply(SS.value, function(x) {
  val <- x[,5]
})
tbl.SS <- list.cbind(tbl.estimate)
tbl.SS <- as.data.frame(tbl.SS)
tbl.SS <- cbind(names.vars, tbl.SS)
colnames(tbl.SS)[4] <- "estimate"

# estimate the differences between columns
# to do so will duplicate the data.frame in order to subtract each column
```

```r
tbl2.SS <- tbl.SS[,5:7]

SS.dif <- cbind(tbl.SS[, c(1:4), drop=F],
        do.call(cbind, lapply(tbl2.SS[,1:2],
                              function(x) tbl.SS[,6:7]-x)))

# putting all variables in one column
SS.hist <- melt(SS.dif, id.vars = c("estimate", "species", "var1"),
                       measure.vars = c(names(SS.dif[5:6]),
                                        names(SS.dif[8])))

# puting NA values for when the difference is 0
# (i.e. when comparing the same variables)
split <- str_split_fixed(SS.hist$variable, "\\.", 2)
SS.hist$cat1 <- split[,2]
SS.hist$cat2 <- split[,1]

## getting the p-value for each difference
## N is not 100 because we took out the values that were outliers

## Arabidopsis
arab.hist <- subset(SS.hist, SS.hist$species == "Arabidopsis")
nboots <- 100
arab.p <- list()
for (i in 1:nboots) {
  arab.p[[i]] <- ddply(arab.hist,
                       c("species","estimate", "var1", "cat1", "cat2"),
                       function(x, N=95){
  c <- as.numeric(nrow(x[x$value < 0,]))
  c2 <- as.numeric(nrow(x[x$value > 0,]))
  m <- min(c, c2)
  p <- (2*m+1)/(N+1)
  tbl <- data.frame(c, c2, m, p)
  })
}

# correcting the p-value for multiple testing
arab.p.adj <- lapply(arab.p, function(x) {
  ddply(x,  c("species","estimate", "var1", "cat1", "cat2"),
        function(x) {
    p.value <- p.adjust(x$p)
    data.frame(p.value)
  })
})

# taking the minimum p-value
tbl.arab.p.adj <- rbindlist(arab.p.adj)
arab.pvalue <- ddply(tbl.arab.p.adj,
                     c("species","estimate", "var1", "cat1", "cat2"),
                     function(x) {
  p.value <- min(x$p.value)
  data.frame(p.value)
})
```

Table 5: Statistics for RSA/Secondary Structure

species	estimate	var1	cat1	cat2	p.value
Arabidopsis	dnds	Buried	Helix	Bsheets	0.0104167
Arabidopsis	dnds	Buried	loops	Bsheets	0.3229167
Arabidopsis	dnds	Buried	loops	Helix	0.0104167
Arabidopsis	dnds	Exposed	Helix	Bsheets	0.0104167
Arabidopsis	dnds	Exposed	loops	Bsheets	0.0104167
Arabidopsis	dnds	Exposed	loops	Helix	0.0104167
Arabidopsis	omegaNA	Buried	Helix	Bsheets	0.9479167
Arabidopsis	omegaNA	Buried	loops	Bsheets	0.4479167
Arabidopsis	omegaNA	Buried	loops	Helix	0.4062500
Arabidopsis	omegaNA	Exposed	Helix	Bsheets	0.0937500
Arabidopsis	omegaNA	Exposed	loops	Bsheets	0.0312500
Arabidopsis	omegaNA	Exposed	loops	Helix	0.6979167
Arabidopsis	omegaA	Buried	Helix	Bsheets	0.1770833
Arabidopsis	omegaA	Buried	loops	Bsheets	0.7604167
Arabidopsis	omegaA	Buried	loops	Helix	0.4062500
Arabidopsis	omegaA	Exposed	Helix	Bsheets	0.5937500
Arabidopsis	omegaA	Exposed	loops	Bsheets	0.3854167
Arabidopsis	omegaA	Exposed	loops	Helix	0.8645833

```
# showing the table
kable(arab.pvalue, caption = "Statistics for RSA/Secondary Structure")
```

```
## Drosophila
dmel.hist <- subset(SS.hist, SS.hist$species == "Drosophila")
nboots <- 100
dmel.p <- list()
for (i in 1:nboots) {
  dmel.p[[i]] <- ddply(dmel.hist,
                       c("species","estimate", "var1", "cat1", "cat2"),
                       function(x, N=96){
  c <- as.numeric(nrow(x[x$value < 0,]))
  c2 <- as.numeric(nrow(x[x$value > 0,]))
  m <- min(c, c2)
  p <- (2*m+1)/(N+1)
  tbl <- data.frame(c, c2, m, p)
  })
}

# correcting the p-value for multiple testing
dmel.p.adj <- lapply(dmel.p, function(x) {
  ddply(x,  c("species","estimate", "var1", "cat1", "cat2"),
        function(x) {
    p.value <- p.adjust(x$p)
    data.frame(p.value)
  })
})

# taking the minimum p-value
```

Table 6: Statistics for RSA/Secondary Structure

species	estimate	var1	cat1	cat2	p.value
Drosophila	dnds	Buried	Helix	Bsheets	0.0103093
Drosophila	dnds	Buried	loops	Bsheets	0.1546392
Drosophila	dnds	Buried	loops	Helix	0.7731959
Drosophila	dnds	Exposed	Helix	Bsheets	0.0515464
Drosophila	dnds	Exposed	loops	Bsheets	0.0309278
Drosophila	dnds	Exposed	loops	Helix	0.0515464
Drosophila	omegaNA	Buried	Helix	Bsheets	0.4432990
Drosophila	omegaNA	Buried	loops	Bsheets	0.3608247
Drosophila	omegaNA	Buried	loops	Helix	0.6494845
Drosophila	omegaNA	Exposed	Helix	Bsheets	0.9587629
Drosophila	omegaNA	Exposed	loops	Bsheets	0.8762887
Drosophila	omegaNA	Exposed	loops	Helix	0.7319588
Drosophila	omegaA	Buried	Helix	Bsheets	0.2783505
Drosophila	omegaA	Buried	loops	Bsheets	0.3402062
Drosophila	omegaA	Buried	loops	Helix	0.6701031
Drosophila	omegaA	Exposed	Helix	Bsheets	0.8144330
Drosophila	omegaA	Exposed	loops	Bsheets	0.7319588
Drosophila	omegaA	Exposed	loops	Helix	0.8969072

```
tbl.dmel.p.adj <- rbindlist(dmel.p.adj)
dmel.pvalue <- ddply(tbl.dmel.p.adj,
                     c("species","estimate", "var1", "cat1", "cat2"),
                     function(x) {
  p.value <- min(x$p.value)
  data.frame(p.value)
})

# showing the table
kable(dmel.pvalue, caption = "Statistics for RSA/Secondary Structure")
```

The next chunk describes the script to plot the results of RSA/Disorder and RSA/Protein length.

```
# theme of the plot
theme.plot <- function(x) {
  theme(axis.title = element_text(face = "bold", color = "black", size=12),
        text = element_text(size=12),
        axis.title.x = element_text(margin = margin(t = 18, r = 10, b = 0, l = 0)),
        axis.title.y = element_text(margin = margin(t = 18, r = 10, b = 0, l = 0)),
        panel.grid.minor=element_blank(),
        panel.grid.major = element_line(colour = "grey", linetype = "dashed", size = 0.2),
        panel.grid.major.y=element_blank(),
        axis.text.x = element_text(angle = 60, hjust = 1),
        legend.text = element_text(size = 7),
        legend.title = element_text(size = 10, face = "bold"))
}

# plotting the continuous variables
plot.correlations.conti <- lapply(tbl.stats[c(1,3:4)], function(x) {
  ggplot(x, aes(x = var2, y = value.mean, fill = var1)) +
```

```
  geom_line(size = 0.3, col = "black")+
  geom_ribbon(aes(ymin=value.mean + 1.96*value.sd,
                  ymax=value.mean - 1.96*value.sd, fill = var1), alpha = 0.8) +
  facet_grid(species~variable, scales = "free_x", labeller = label_parsed) +
  ylab("") +
  xlab(as.character(x$var)) +
  scale_fill_grey() +
  scale_color_grey() +
  theme_bw() +
  theme.plot()
})

plot.correlations.conti
```

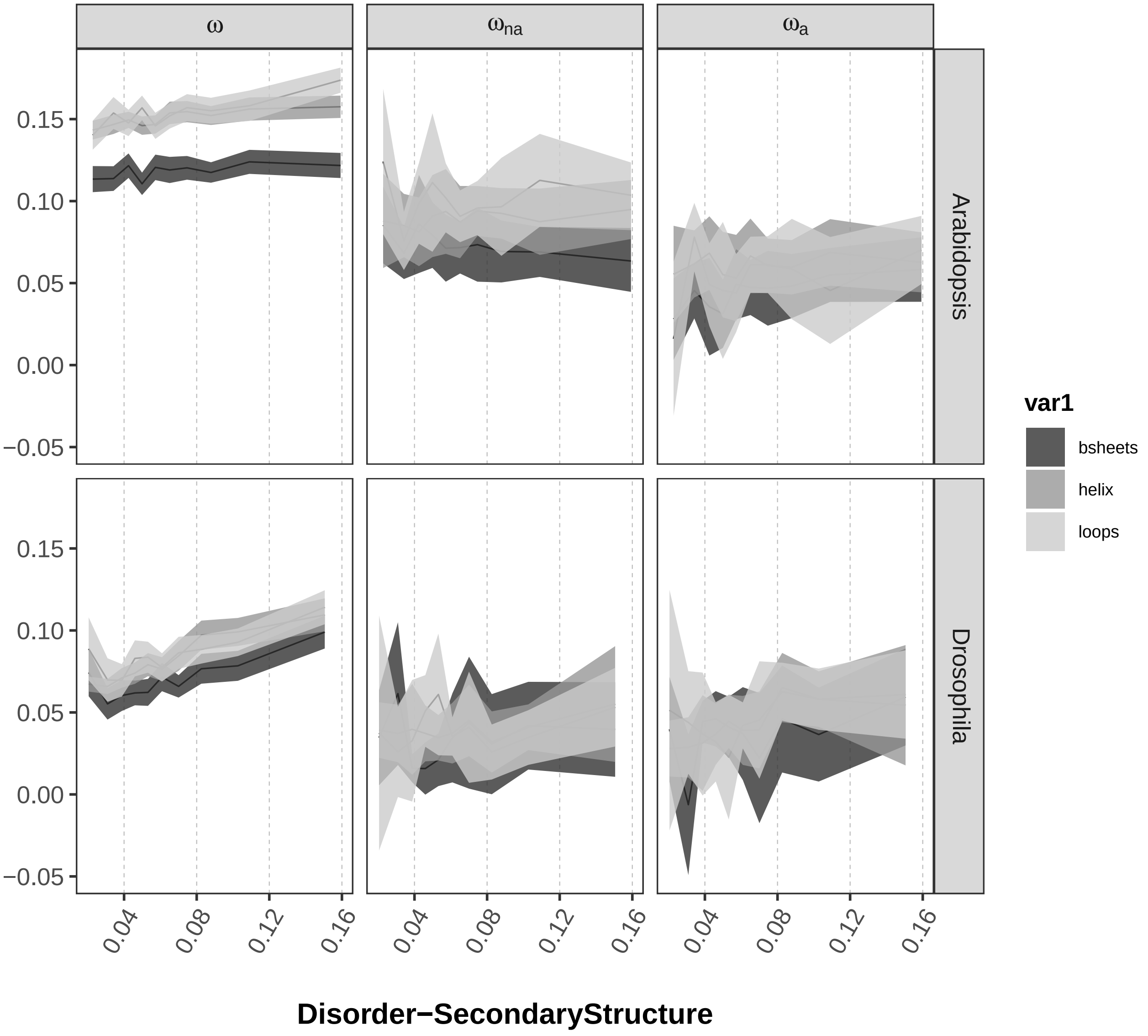

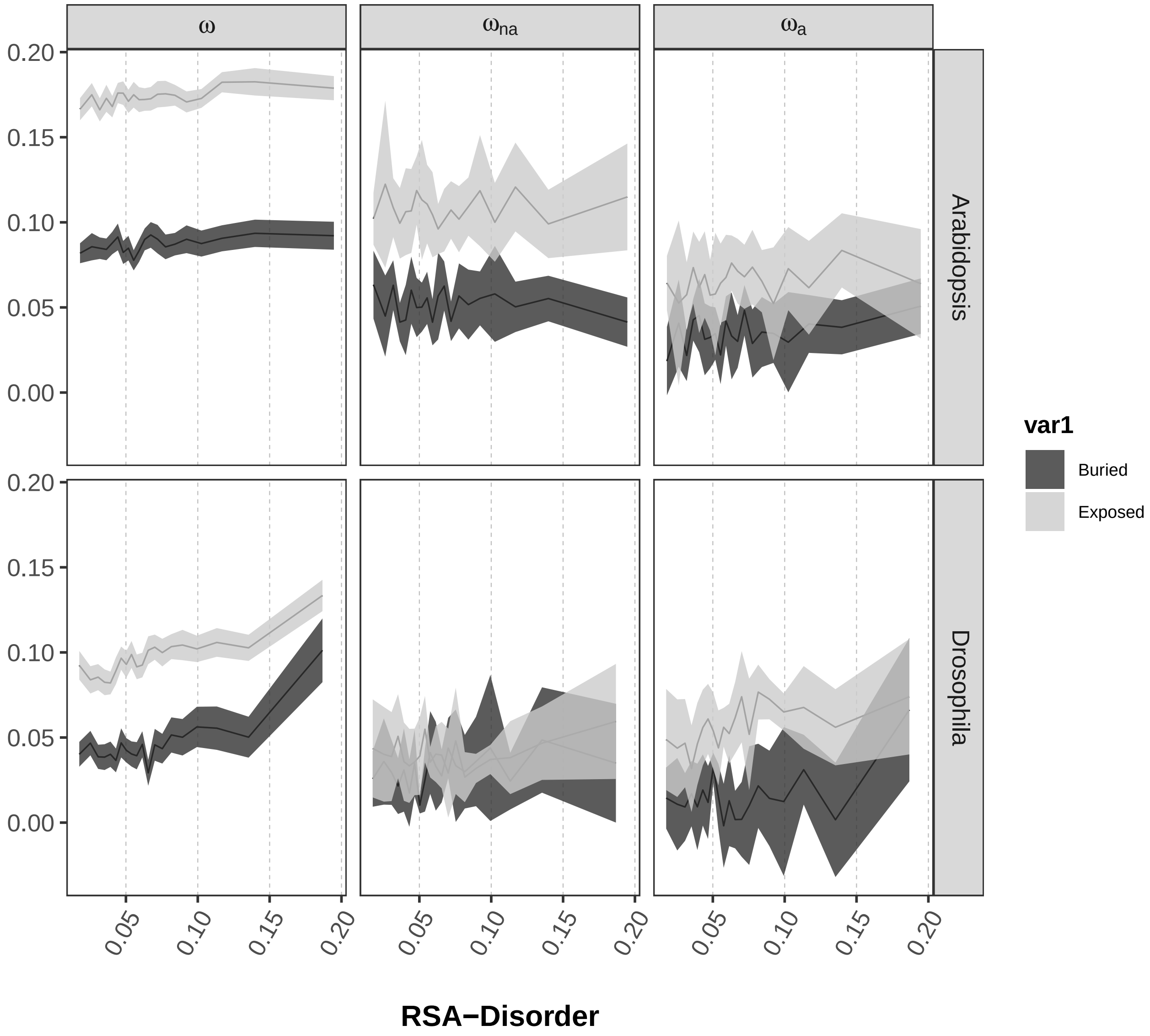
ω
ωna
ωa
0.20
0.15
0.10
0.05
0.00
Arabidopsis
Drosophila
0.05
0.10
0.15
0.20
var1
Buried
Exposed
RSA−Disorder

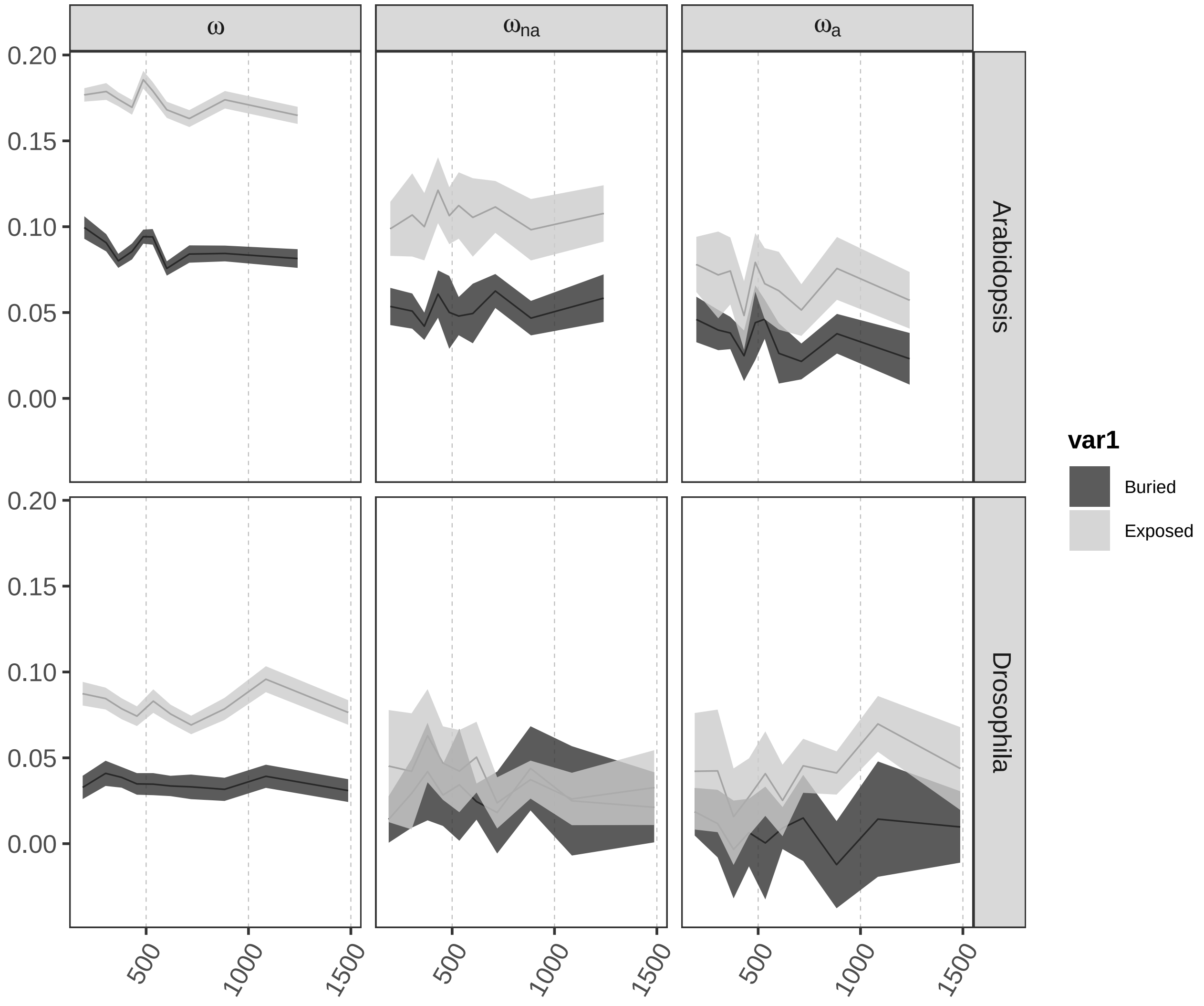

RSA−ProteinLength

The last section shows how the statistical analyses for RSA/Disorder and RSA/Protein length were performed.

```
stat <- lapply(tbl.stats[c(1,3:4)], function(x) {
  ddply(x, c("var", "species", "variable", "var1"), function(x) {
    var <- as.numeric(factor(x$var2))
    variable.value <- as.numeric(factor(x$value.mean))
    corr = cor.test(var, variable.value, method = "kendall", exact = FALSE)
    Kendall.tau = corr$estimate
    p.value = corr$p.value
    dat = data.frame(Kendall.tau, p.value)
  })
})

# showing the tables
for(i in stat) {
  print(kable(x = i, caption = paste0("Statistics for ", unique(i$var))))
}
```

The next chunk describes the linear model performed between RSA and intrinsic protein disorder

Table 7: Statistics for Disorder-SecondaryStructure

var	species	variable	var1	Kendall.tau	p.value
Disorder-SecondaryStructure	Arabidopsis	omega	bsheets	0.4222222	0.0892416
Disorder-SecondaryStructure	Arabidopsis	omega	helix	0.7333333	0.0031612
Disorder-SecondaryStructure	Arabidopsis	omega	loops	0.6444444	0.0094911
Disorder-SecondaryStructure	Arabidopsis	omega[na]	bsheets	-0.5111111	0.0396687
Disorder-SecondaryStructure	Arabidopsis	omega[na]	helix	0.4222222	0.0892416
Disorder-SecondaryStructure	Arabidopsis	omega[na]	loops	0.0666667	0.7884467
Disorder-SecondaryStructure	Arabidopsis	omega[a]	bsheets	0.6888889	0.0055589
Disorder-SecondaryStructure	Arabidopsis	omega[a]	helix	0.2000000	0.4208286
Disorder-SecondaryStructure	Arabidopsis	omega[a]	loops	0.2000000	0.4208286
Disorder-SecondaryStructure	Drosophila	omega	bsheets	0.6888889	0.0055589
Disorder-SecondaryStructure	Drosophila	omega	helix	0.9111111	0.0002453
Disorder-SecondaryStructure	Drosophila	omega	loops	0.5555556	0.0253473
Disorder-SecondaryStructure	Drosophila	omega[na]	bsheets	0.1555556	0.5312500
Disorder-SecondaryStructure	Drosophila	omega[na]	helix	0.2444444	0.3251795
Disorder-SecondaryStructure	Drosophila	omega[na]	loops	0.1111111	0.6547208
Disorder-SecondaryStructure	Drosophila	omega[a]	bsheets	0.1555556	0.5312500
Disorder-SecondaryStructure	Drosophila	omega[a]	helix	0.7777778	0.0017451
Disorder-SecondaryStructure	Drosophila	omega[a]	loops	0.3333333	0.1797125

Table 8: Statistics for RSA-Disorder

var	species	variable	var1	Kendall.tau	p.value
RSA-Disorder	Arabidopsis	omega	Buried	0.4526316	0.0052674
RSA-Disorder	Arabidopsis	omega	Exposed	0.3684211	0.0231409
RSA-Disorder	Arabidopsis	omega[na]	Buried	-0.0631579	0.6970310
RSA-Disorder	Arabidopsis	omega[na]	Exposed	-0.0210526	0.8967428
RSA-Disorder	Arabidopsis	omega[a]	Buried	0.2105263	0.1943659
RSA-Disorder	Arabidopsis	omega[a]	Exposed	0.2105263	0.1943659
RSA-Disorder	Drosophila	omega	Buried	0.5052632	0.0018416
RSA-Disorder	Drosophila	omega	Exposed	0.7157895	0.0000102
RSA-Disorder	Drosophila	omega[na]	Buried	0.2947368	0.0692355
RSA-Disorder	Drosophila	omega[na]	Exposed	-0.0315789	0.8456547
RSA-Disorder	Drosophila	omega[a]	Buried	0.0842105	0.6036850
RSA-Disorder	Drosophila	omega[a]	Exposed	0.5368421	0.0009352

Table 9: Statistics for RSA-ProteinLength

var	species	variable	var1	Kendall.tau	p.value
RSA-ProteinLength	Arabidopsis	omega	Buried	-0.3777778	0.1283788
RSA-ProteinLength	Arabidopsis	omega	Exposed	-0.4222222	0.0892416
RSA-ProteinLength	Arabidopsis	omega[na]	Buried	-0.0222222	0.9287301
RSA-ProteinLength	Arabidopsis	omega[na]	Exposed	0.0666667	0.7884467
RSA-ProteinLength	Arabidopsis	omega[a]	Buried	-0.4222222	0.0892416
RSA-ProteinLength	Arabidopsis	omega[a]	Exposed	-0.2888889	0.2449288
RSA-ProteinLength	Drosophila	omega	Buried	-0.4222222	0.0892416
RSA-ProteinLength	Drosophila	omega	Exposed	-0.2444444	0.3251795
RSA-ProteinLength	Drosophila	omega[na]	Buried	-0.0666667	0.7884467
RSA-ProteinLength	Drosophila	omega[na]	Exposed	-0.4222222	0.0892416
RSA-ProteinLength	Drosophila	omega[a]	Buried	-0.0666667	0.7884467
RSA-ProteinLength	Drosophila	omega[a]	Exposed	0.3333333	0.1797125

```r
####
#### Linear model:
####

arab.omegaA <- subset(tbl.stats[[3]], variable == "omega[a]" & species == "Arabidopsis")
arab.omegaNA <- subset(tbl.stats[[3]], variable == "omega[na]" & species == "Arabidopsis")
dmel.omegaA <- subset(tbl.stats[[3]], variable == "omega[a]" & species == "Drosophila")
dmel.omegaNA <- subset(tbl.stats[[3]], variable == "omega[na]" & species == "Drosophila")

## doing the linear model:
# Note: var1 = RSA, var2 = Protein Disorder
m.arabA <- lm(value.mean ~ var1 * var2, arab.omegaA)
m.arabNA <- lm(value.mean ~ var1 * var2, arab.omegaNA)
m.dmelA <- lm(value.mean ~ var1 * var2, dmel.omegaA)
m.dmelNA <- lm(value.mean ~ var1 * var2, dmel.omegaNA)

## checking the normality of the error:
hist(resid(m.arabA))
```

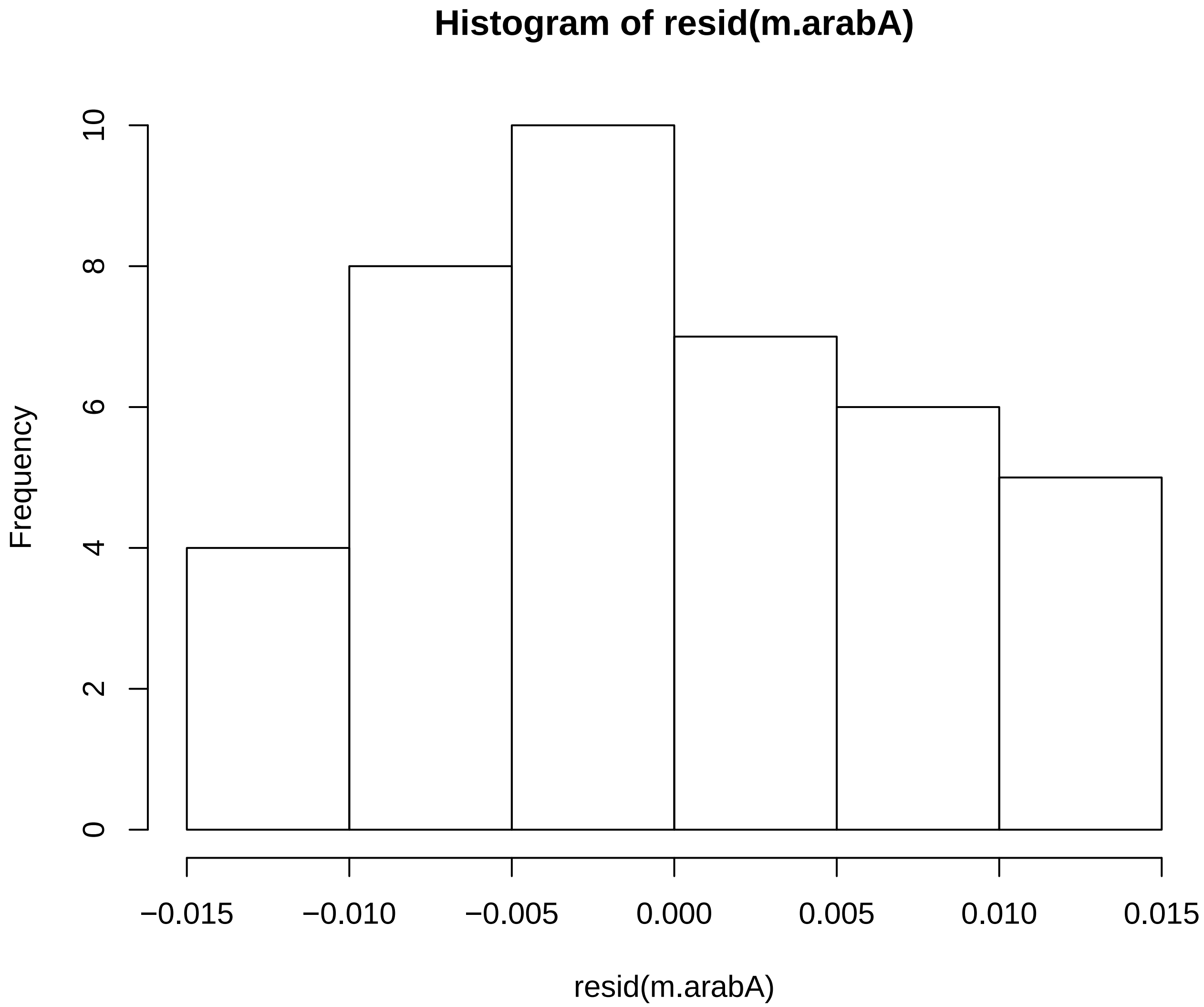


```
normal.arabA <- shapiro.test(resid(m.arabA))
normal.arabA$p.value
```

[1] 0.4264054

```
hist(resid(m.arabNA))
```

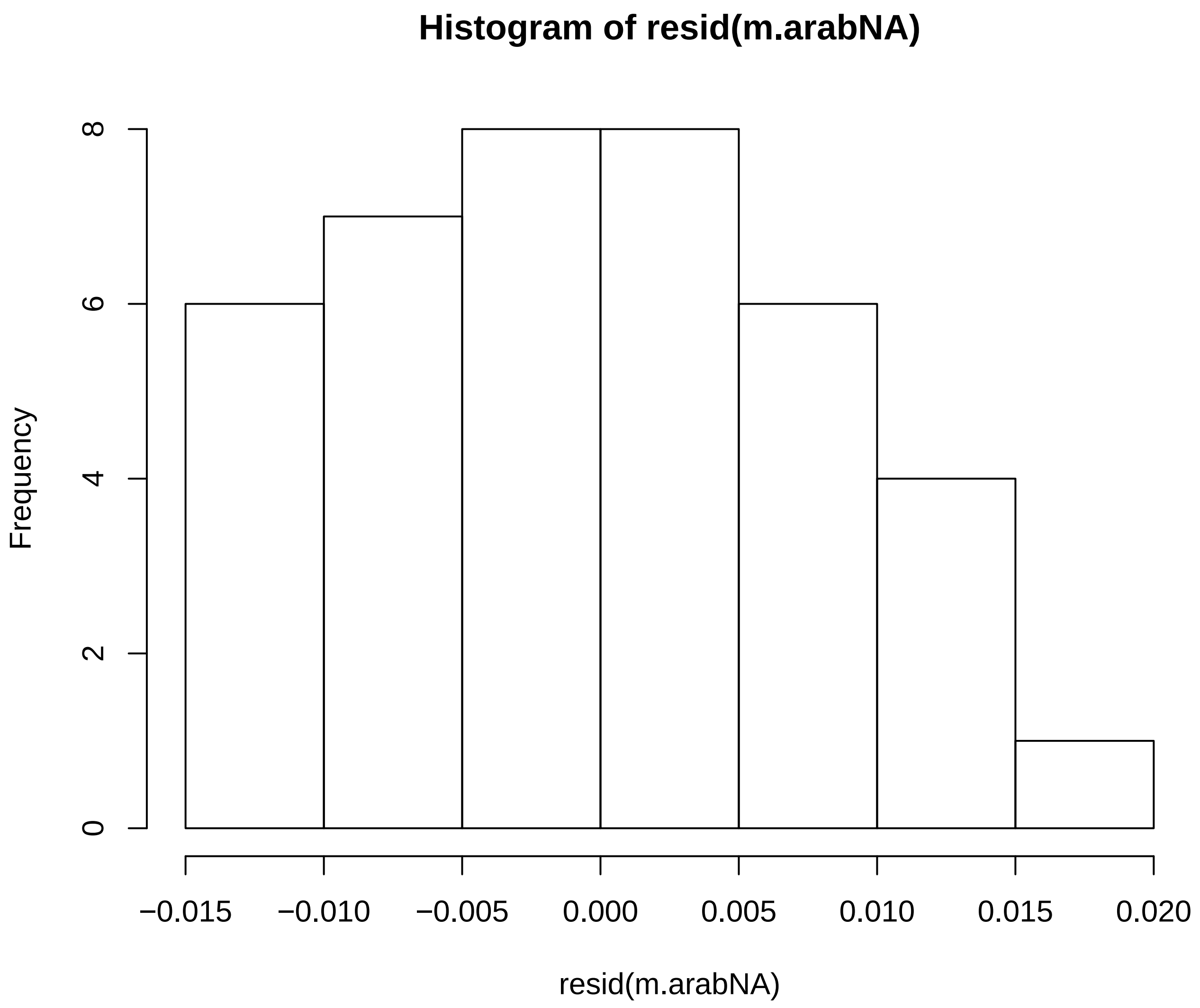


```
normal.arabNA <- shapiro.test(resid(m.arabNA))
normal.arabNA$p.value
```

[1] 0.1467622

```
#
hist(resid(m.dmelA))
```

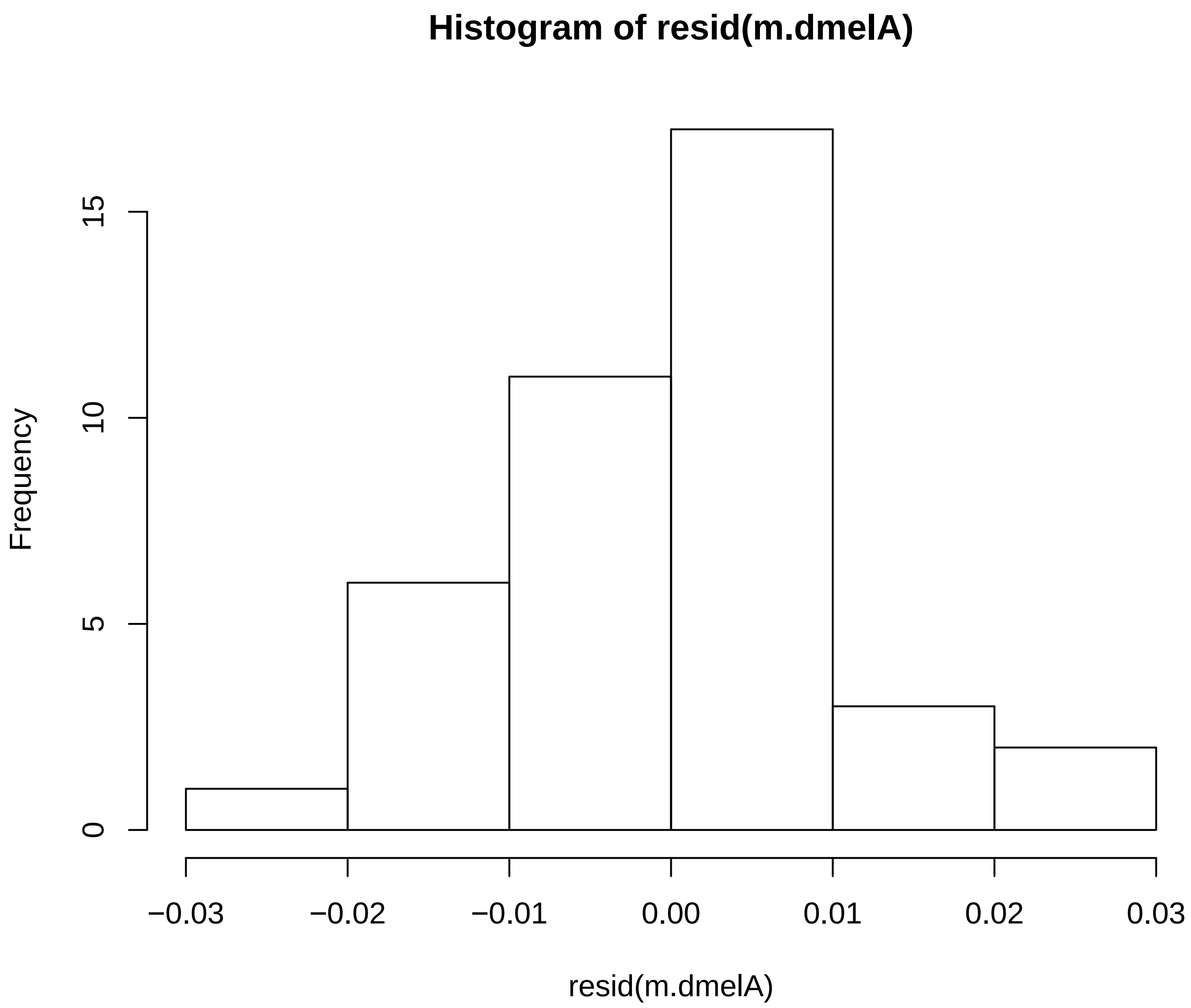


```
normal.dmelA <- shapiro.test(resid(m.dmelA))
normal.dmelA$p.value
```

[1] 0.923666

```
hist(resid(m.dmelNA))
```

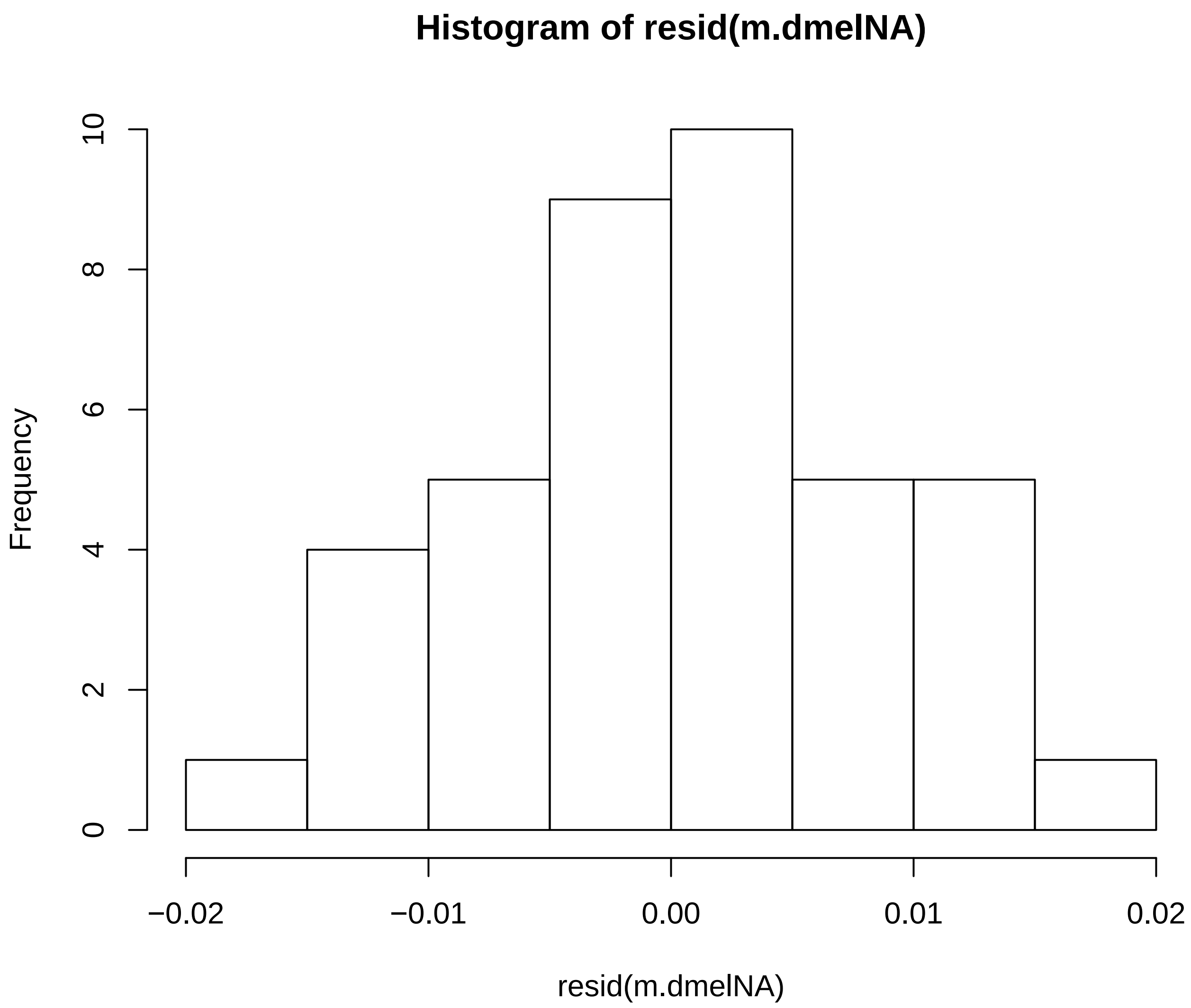


```
normal.dmelNA <- shapiro.test(resid(m.dmelNA))
normal.dmelNA$p.value
```

[1] 0.8044075

```
## checking the homogeneicity of the error:
library(lmtest)
hm.arabA <- hmctest(m.arabA)
hm.arabA$p.value
```

[1] 0.503

```
hm.arabNA <- hmctest(m.arabNA)
hm.arabNA$p.value
```

[1] 0.435

```
#
hm.dmelA <- hmctest(m.dmelA)
hm.dmelA$p.value
```

[1] 0.922

Table 10: Relative contribution of RSA and protein disorder

species	variable	RSA	Disorder	inter	resid
Arabidopsis	omegaA	95.57022	3.517072	0.2577141	0.6549903

```
hm.dmelNA <- hmctest(m.dmelNA)
hm.dmelNA$p.value
```

[1] 0.638

```
## checking if the errors of each point are independent:
dw.arabA <- dwtest(m.arabA)
dw.arabA$p.value
```

[1] 0.8732475

```
dw.arabNA <- dwtest(m.arabNA)
dw.arabNA$p.value
```

[1] 0.8931914

```
#
dw.dmelA <- dwtest(m.dmelA)
dw.dmelA$p.value
```

[1] 0.4362952

```
dw.dmelNA <- dwtest(m.dmelNA)
dw.dmelNA$p.value
```

[1] 0.7481026

```
## now lets do an ANOVA to see the percentage of the variance that each variable (RSA and Dis) explains
a.arabA <- anova(m.arabA)
a.arabNA <- anova(m.arabNA)
#
a.dmelA <- anova(m.dmelA)
a.dmelNA <- anova(m.dmelNA)

# checking the percentage of contribution of each variable:
# arab, omegaA
prop.arabA <- a.arabA$`Mean Sq` *100/ sum(a.arabA$`Mean Sq`)
RSA <- prop.arabA[1]
Disorder <-  prop.arabA[2]
inter <-  prop.arabA[3]
resid <-  prop.arabA[4]
tbl.arabA <- data.frame(species = "Arabidopsis",variable = "omegaA", RSA, Disorder, inter, resid)
kable(tbl.arabA, caption = "Relative contribution of RSA and protein disorder")
```

```
# arab, omegaNA
prop.arabNA <- a.arabNA$`Mean Sq` *100/ sum(a.arabNA$`Mean Sq`)
RSA <- prop.arabNA[1]
Disorder <-  prop.arabNA[2]
inter <-  prop.arabNA[3]
resid <-  prop.arabNA[4]
tbl.arabNA <- data.frame(species = "Arabidopsis",variable = "omegaNA", RSA, Disorder, inter, resid)
```

Table 11: Relative contribution of RSA and protein disorder

species	variable	RSA	Disorder	inter	resid
Arabidopsis	omegaNA	99.65678	0.0125986	0.1301738	0.2004436

Table 12: Relative contribution of RSA and protein disorder

species	variable	RSA	Disorder	inter	resid
Drosophila	omegaA	87.32906	12.01763	0.0047397	0.6485733

```
kable(tbl.arabNA, caption = "Relative contribution of RSA and protein disorder")
```

```
# dmel, omegaA
prop.dmelA <- a.dmelA$`Mean Sq` *100/ sum(a.dmelA$`Mean Sq`)
RSA <- prop.dmelA[1]
Disorder <-  prop.dmelA[2]
inter <-  prop.dmelA[3]
resid <-  prop.dmelA[4]
tbl.dmelA <- data.frame(species = "Drosophila",  variable = "omegaA", RSA, Disorder, inter, resid)
kable(tbl.dmelA, caption = "Relative contribution of RSA and protein disorder")
```

```
# dmel, omegaNA
prop.dmelNA <- a.dmelNA$`Mean Sq` *100/ sum(a.dmelNA$`Mean Sq`)
RSA <- prop.dmelNA[1]
Disorder <-  prop.dmelNA[2]
inter <-  prop.dmelNA[3]
resid <-  prop.dmelNA[4]
tbl.dmelNA <- data.frame(species = "Drosophila",variable = "omegaNA", RSA, Disorder, inter, resid)
kable(tbl.dmelNA, caption = "Relative contribution of RSA and protein disorder")
```

```
#
```

Table 13: Relative contribution of RSA and protein disorder

species	variable	RSA	Disorder	inter	resid
Drosophila	omegaNA	62.83181	30.37997	1.033083	5.755141
