# Supplementary material for: The Impact of Protein Architecture on Adaptive Evolution
Source: Mol Biol Evol. 2019 May 30;36(9):2013–28. doi: 10.1093/molbev/msz134 (PMC6735723; doi:10.1093/molbev/msz134)
Supplement: msz134_Supplementary_Data [file msz134_supplementary_data.zip › FileS6.pdf]

Relationship between variables

This notebook describes the R scripts used to analyze the relationship between variables. It will include the relationships between:

Continuous variables:
files.continuous[1] <- Breadth/Mean Gene Expression
files.continuous[2] <- Protein length/Gene expression
files.continuous[3] <- RSA/Residue intrinsic disorder
files.continuous[4] <- RSA/Gene Expression
files.continuous[5] <- RSA/Protein length

Continuous/Discrete:
files.discrete[1] <- Protein Functional Class/RSA
files.discrete[2] <- Intrinsic Disorder/Secondary structure
files.discrete[3] <- Secondary Structure/RSA

The first chunk describes the analyses performed for comparing continuous variables.

```r
setwd("/Users/moutinho/Dropbox/Data/VariablesRelationship/Continuous/")
# Libraries
library(plyr)
library(dplyr)
library(data.table)
library(ggplot2)
library(reshape2)
library(stringr)
library(knitr)
library(kableExtra)
#

# calling all output tables
files.continuous <- list.files(".", ".csv")

# reading each table into a list
tbl.list <- lapply(files.continuous, fread, header = TRUE)
```

```r
# now plotting the relationship
# theme of the plot
theme.plot <- function(x) {
  theme(axis.title = element_text(face = "bold", color = "black", size=12,
                                  family = "Times"),
        text = element_text(size=12),
        axis.title.x = element_text(margin = margin(t = 9, r = 10, b = 0, l = 0)),
        axis.title.y = element_text(margin = margin(t = 9, r = 10, b = 0, l = 0)),
        panel.grid.minor=element_blank(),
        panel.grid.major = element_line(colour = "grey", linetype = "dashed", size = 0.1),
        panel.grid.major.y=element_blank(),
        strip.text.y = element_blank(),
        axis.text.x = element_text(angle = 60, hjust = 1),
        strip.background.x = element_rect(colour = "grey", fill = "gray92"),
        legend.text = element_text(size = 7),
        legend.title = element_text(size = 9, face = "bold"))
}
```

```r
# will do the plots seperately due to the different scales
# breadth / mean gene expression
plot.breadth.mean <- ggplot(tbl.list[[1]], aes(med.var1, med.var2)) +
  geom_line(col = "black", size = 0.4) +
  geom_ribbon(aes(ymax=thirdQ, ymin=firstQ), alpha=0.2) +
  geom_point(size=.6) +
  geom_quantile(aes(var2, var1), quantiles = 0.5) +
  facet_grid(rows = vars(species)) +
  xlab(as.character(unique(str_split_fixed(tbl.list[[1]]$var, "\\-", 2)[,2]))) +
  ylab(as.character(unique(str_split_fixed(tbl.list[[1]]$var, "\\-", 2)[,1]))) +
  scale_x_continuous(limits = c(1, 7)) +
  theme_bw() +
  theme.plot()

plot.breadth.mean
```

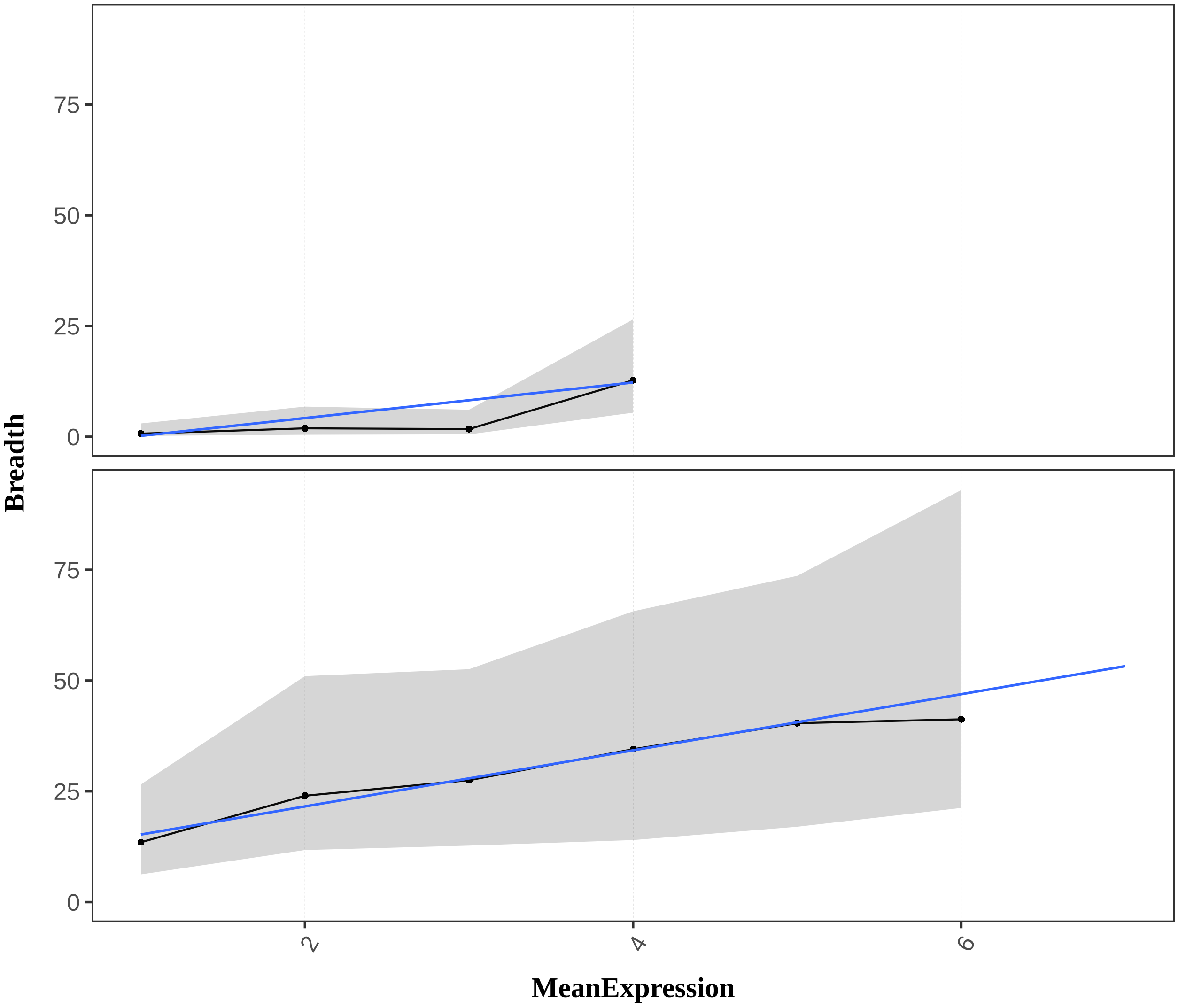


```r
#proten length/gene expression
plot.len.exp <- ggplot(tbl.list[[2]], aes(med.var1, med.var2)) +
```

```
  geom_line(col = "black", size = 0.4) +
  geom_ribbon(aes(ymax=thirdQ, ymin=firstQ), alpha=0.2) +
  geom_point(size=.6) +
  geom_quantile(aes(var1, var2), quantiles = 0.5) +
  facet_grid(rows = vars(species)) +
  xlab(as.character(unique(str_split_fixed(tbl.list[[2]]$var, "\\-", 2)[,2]))) +
  ylab(as.character(unique(str_split_fixed(tbl.list[[2]]$var, "\\-", 2)[,1]))) +
  scale_x_log10(limits = c(200, 15000)) +
  theme_bw() +
  theme.plot()

plot.len.exp
```

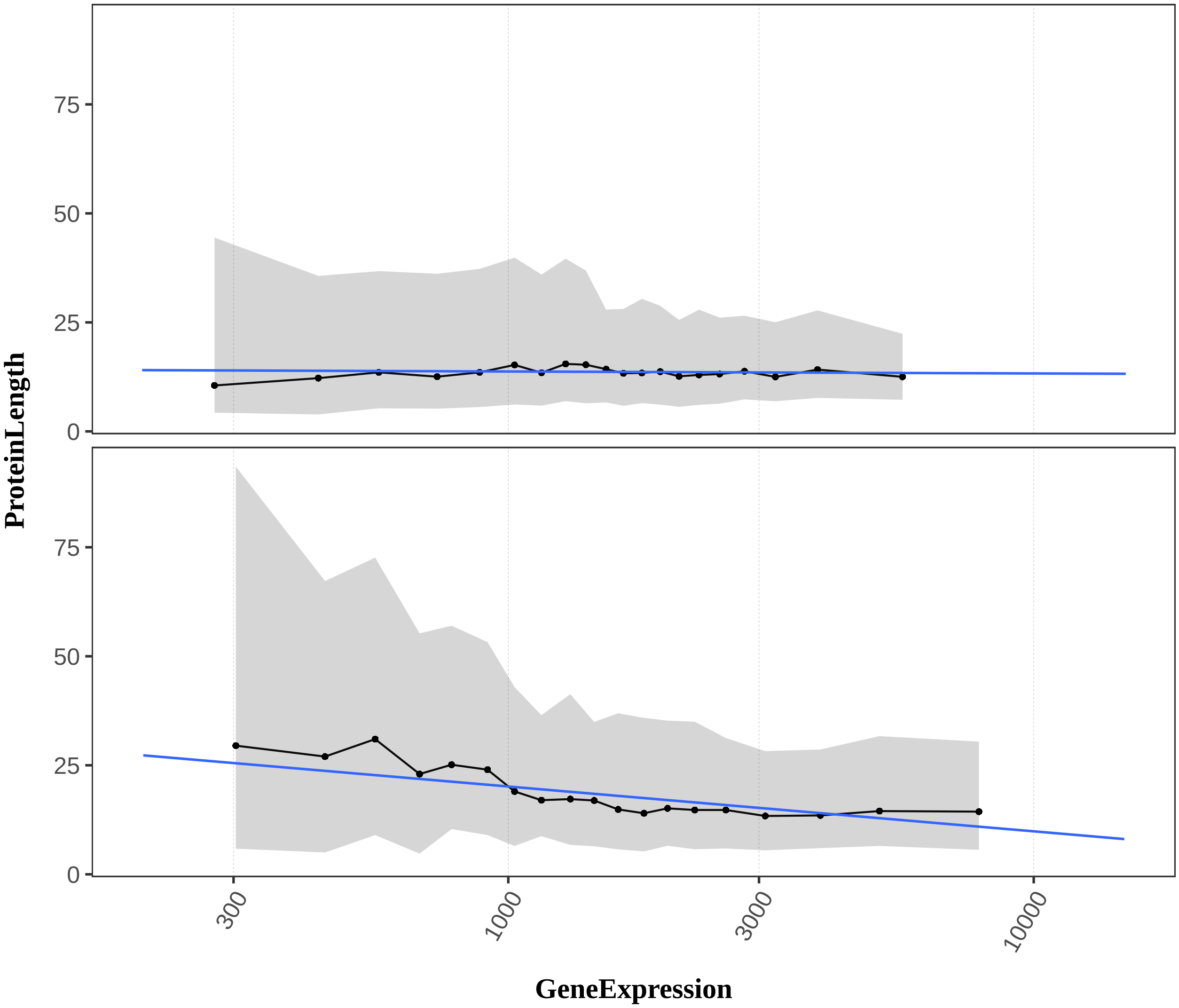


```
# residue intrinsic disorder/rsa
# sampled random 10000 rows to plot the linear model
sub.dis.rsa <- tbl.list[[3]][sample(nrow(tbl.list[[3]]), 10000), ]
plot.dis.rsa <- ggplot(tbl.list[[3]], aes(med.var1, med.var2)) +
  geom_line(col = "black", size = 0.4) +
  geom_ribbon(aes(ymax=thirdQ, ymin=firstQ), alpha=0.2) +
  geom_point(size=.6) +
```

```r
  geom_quantile(data = sub.dis.rsa,
                aes(var1, var2),
                quantiles = 0.5) +
  facet_grid(rows = vars(species)) +
  xlab(as.character(unique(str_split_fixed(tbl.list[[3]]$var, "\\-", 2)[,2]))) +
  ylab(as.character(unique(str_split_fixed(tbl.list[[3]]$var, "\\-", 2)[,1]))) +
  scale_x_log10(limits = c(0.015, 0.22)) +
  theme_bw() +
  theme.plot()

plot.dis.rsa
```

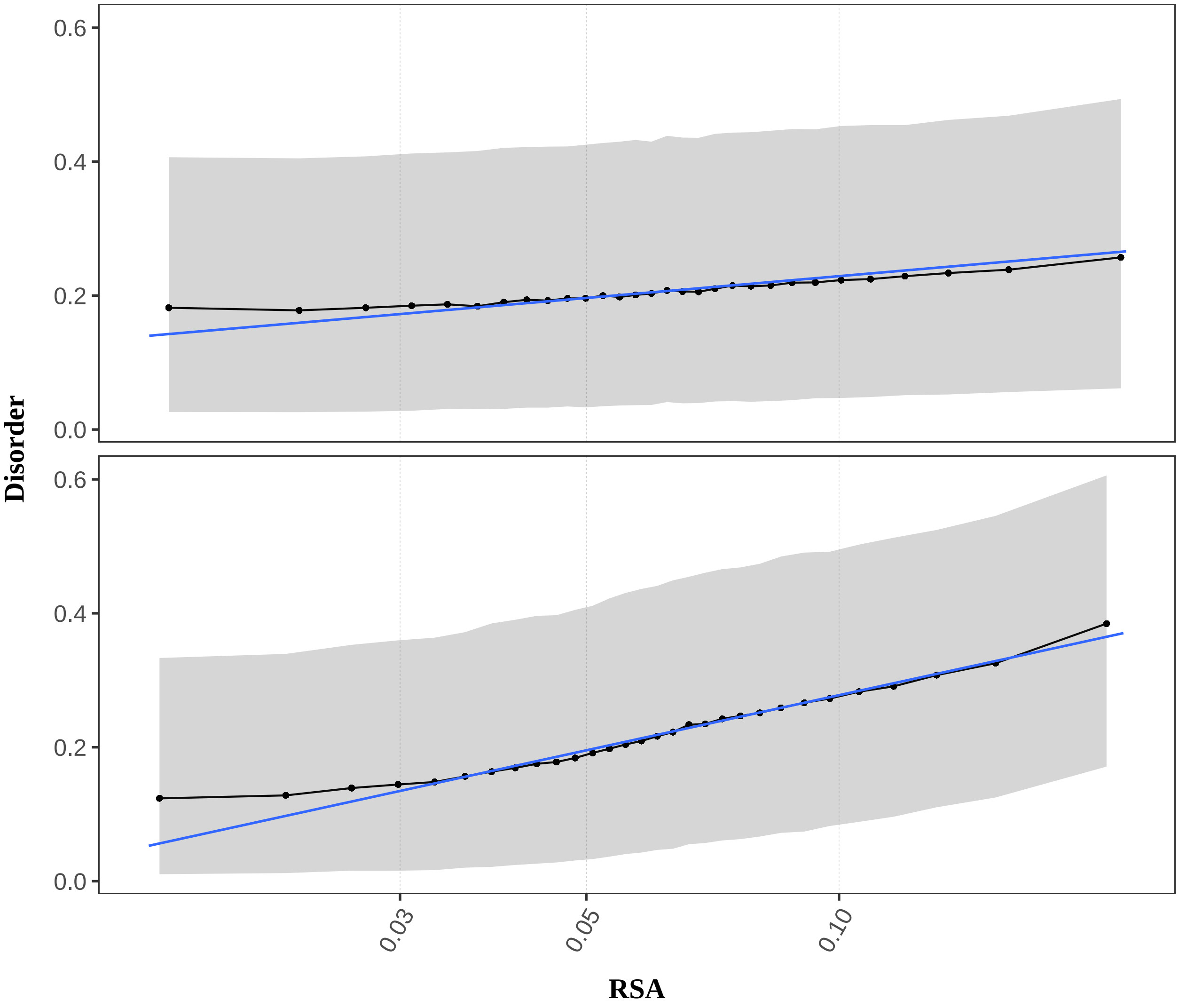


```r
# mean gene expression/solvent exposure
plot.rsa.exp <- ggplot(tbl.list[[4]], aes(med.var1, med.var2)) +
  geom_line(col = "black", size = 0.4) +
  geom_ribbon(aes(ymax=thirdQ, ymin=firstQ), alpha=0.2) +
  geom_point(size=.6) +
  geom_quantile(aes(var1, var2),
                quantiles = 0.5) +
  facet_grid(rows = vars(species)) +
```

```
  xlab(as.character(unique(str_split_fixed(tbl.list[[4]]$var, "\\-", 2)[,1]))) +
  ylab(as.character(unique(str_split_fixed(tbl.list[[4]]$var, "\\-", 2)[,2]))) +
  scale_x_continuous(limits = c(0, 180)) +
  theme_bw() +
  theme.plot()

plot.rsa.exp
```

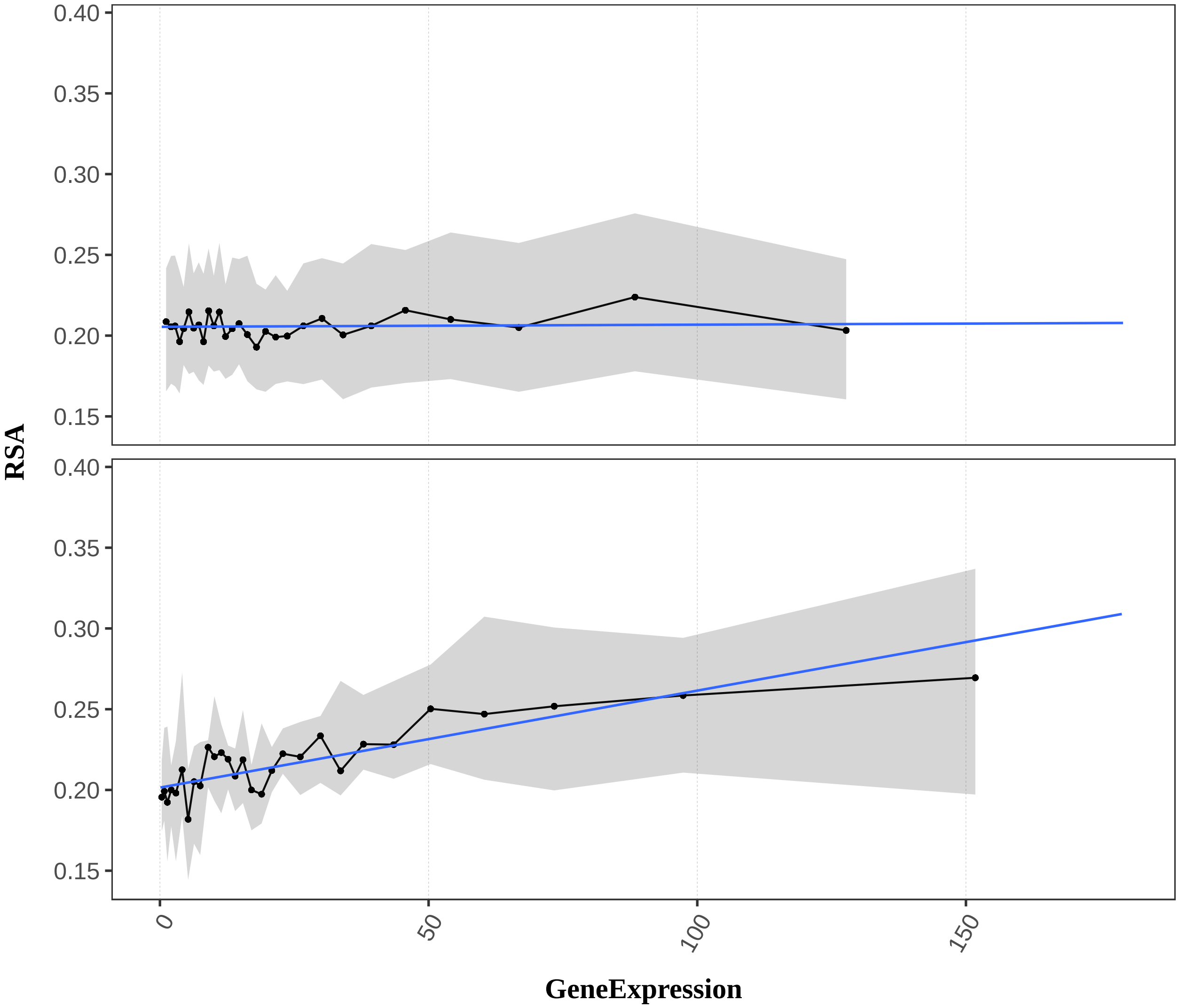


```
# protein length/proportion of exposed residues
plot.rsa.length <- ggplot(tbl.list[[5]], aes(med.var1, med.var2)) +
  geom_line(col = "black", size = 0.4) +
  geom_ribbon(aes(ymax=thirdQ, ymin=firstQ), alpha=0.2) +
  geom_point(size=.6) +
  geom_quantile(aes(var1, var2),
                quantiles = 0.5) +
  facet_grid(rows = vars(species)) +
  xlab(as.character(unique(str_split_fixed(tbl.list[[5]]$var, "\\-", 2)[,1]))) +
  ylab(as.character(unique(str_split_fixed(tbl.list[[5]]$var, "\\-", 2)[,2]))) +
  scale_x_log10(limits = c(100, 2000)) +
  theme_bw() +
```

```
  theme.plot()

plot.rsa.length
```

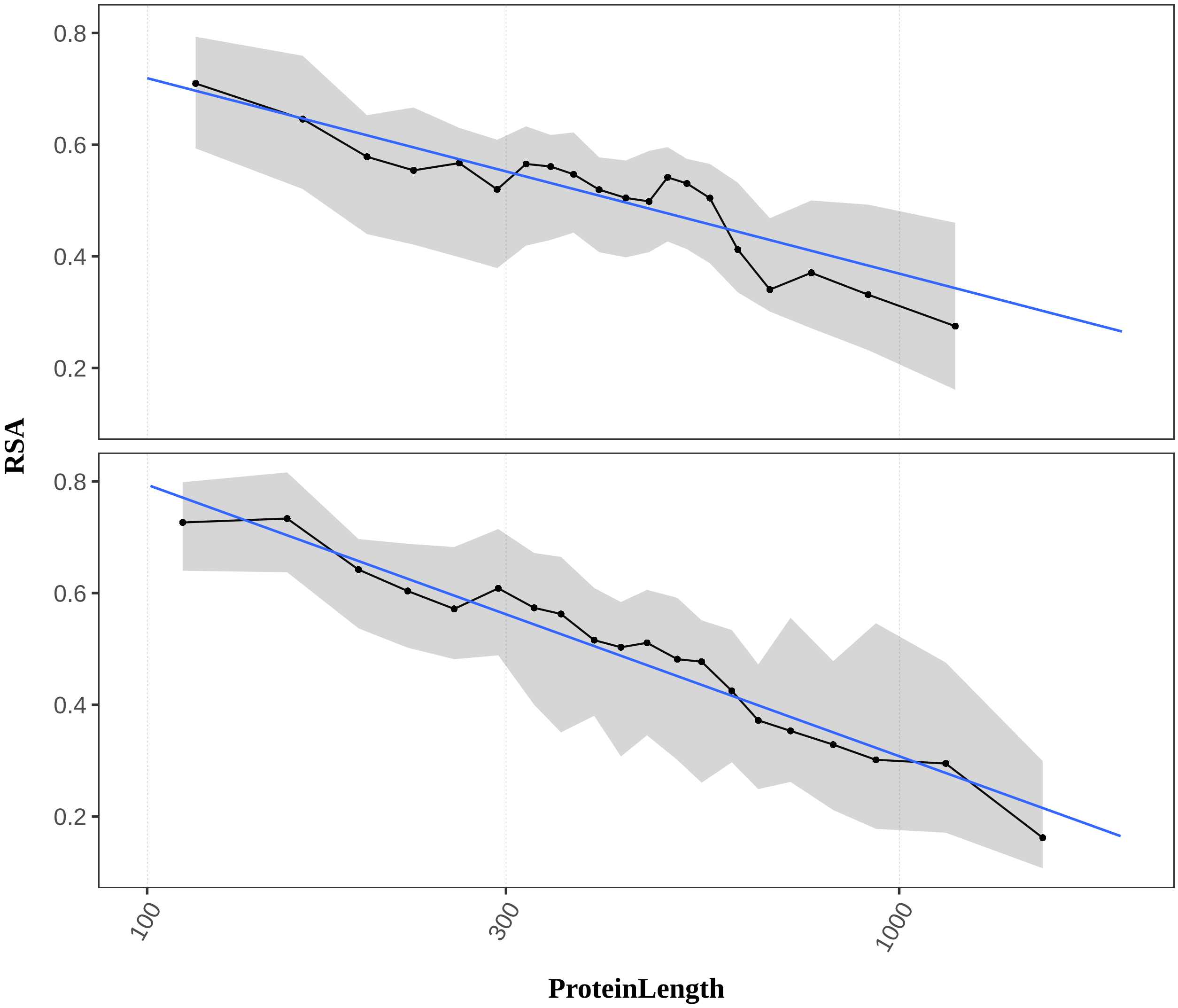

The next chunk presents the R script to estimate the statistics for every correlation.

```
# Breadth/Mean Gene Expression
tbls1 <- ddply(tbl.list[[1]], c("var","species"), function(x) {
  var <- as.numeric(factor(x$var1))
  variable.value <- as.numeric(factor(x$var2))
  corr = cor.test(var, variable.value, method = "kendall", exact = FALSE)
  Kendall.tau = corr$estimate
  p.value = corr$p.value
  dat = data.frame(Kendall.tau, p.value)
})

kable(tbls1, caption = "Statistics for Breadth/Mean Gene Expression")
```

```
# Protein Length/Gene Expression
tbls1 <- ddply(tbl.list[[2]], c("var","species"), function(x) {
```

Table 1: Statistics for Breadth/Mean Gene Expression

var	species	Kendall.tau	p.value
Breadth-MeanExpression	Arabidopsis	0.3376578	0
Breadth-MeanExpression	Drosophila	0.2170258	0

Table 2: Statistics for Protein Length/Gene Expression

var	species	Kendall.tau	p.value
ProteinLength-GeneExpression	Arabidopsis	-0.0147082	0.0122551
ProteinLength-GeneExpression	Drosophila	-0.0935642	0.0000000

```
  var <- as.numeric(factor(x$var1))
  variable.value <- as.numeric(factor(x$var2))
  corr = cor.test(var, variable.value, method = "kendall", exact = FALSE)
  Kendall.tau = corr$estimate
  p.value = corr$p.value
  dat = data.frame(Kendall.tau, p.value)
})

kable(tbls1, caption = "Statistics for Protein Length/Gene Expression")
```

```
# Residue disorder/RSA --> need to do the statistics by sampling points several times
rep.dis.rsa <- list()
nboots <- 1000
for (j in 1:nboots) {
  rep.dis.rsa[[j]] <- ddply(tbl.list[[3]], "species", function(x) {
    x[sample.int(10000, replace = TRUE), ]
 })}

# then compute the statistics for each sampling point
tbls2 <- lapply(rep.dis.rsa, function(x) {
  ddply(x, c("species"), function(y) {
    var <- as.numeric(factor(y$var1))
    variable.value <- as.numeric(factor(y$var2))
    corr <- cor.test(var, variable.value, method = "kendall", exact = FALSE)
    Kendall.tau <- corr$estimate
    p.value <- corr$p.value
    data.frame(Kendall.tau, p.value)
  })
})
stat.dis.rsa <- rbindlist(tbls2)

# take the median values
stats.tbl2 <- ddply(stat.dis.rsa, "species", function(x) {
  kendall.tau <- median(x$Kendall.tau)
  p.value <- median(x$p.value)
  data.frame(kendall.tau, p.value)
})
kable(stats.tbl2, caption = "Statistics for Intrinsic Disorder/RSA")
```

Table 3: Statistics for Intrinsic Disorder/RSA

species	kendall.tau	p.value
Arabidopsis	0.0014389	0.4726331
Drosophila	0.0151135	0.0256188

Table 4: Statistics for Gene Expression/RSA

species	var	Kendall.tau	p.value
Arabidopsis	GeneExpression-RSA	0.0162997	0.1037291
Drosophila	GeneExpression-RSA	0.3274048	0.0000000

```
# gene expression/RSA
tbls3 <- ddply(tbl.list[[4]], c("species", "var"), function(x) {
  var <- as.numeric(factor(x$var1))
  variable.value <- as.numeric(factor(x$var2))
  corr = cor.test(var, variable.value, method = "kendall", exact = FALSE)
  Kendall.tau = corr$estimate
  p.value = corr$p.value
  dat = data.frame(Kendall.tau, p.value)
})

kable(tbls3, caption = "Statistics for Gene Expression/RSA")
```

```
# protein length/proportion of exposed residues
tbls4 <- ddply(tbl.list[[5]], c("species", "var"), function(x) {
  var <- as.numeric(factor(x$var1))
  variable.value <- as.numeric(factor(x$var2))
  corr = cor.test(var, variable.value, method = "kendall", exact = FALSE)
  Kendall.tau = corr$estimate
  p.value = corr$p.value
  dat = data.frame(Kendall.tau, p.value)
})

kable(tbls4, caption = "Statistics for Protein Length/Solvent Exposure")
```

The next chunk describes the analyses between discrete and continuous variables, specifically: files.discrete[1] <- Disorder/Secondary structure files.discrete[2] <- RSA/Secondary structure

```
setwd("/Users/moutinho/Dropbox/Data/VariablesRelationship/Discrete/")

# calling all output tables
files.discrete <- list.files(".", ".csv")

# reading each table into a list
```

Table 5: Statistics for Protein Length/Solvent Exposure

species	var	Kendall.tau	p.value
Arabidopsis	ProteinLength-RSA	-0.3102155	0
Drosophila	ProteinLength-RSA	-0.4040298	0

```
tbl.discrete <- lapply(files.discrete, fread, header = TRUE)
```

The next chunck describes the R scripts used to plot these relationships.

```
# plotting the relationship between secondary structure and relative solvent accessibility
tbl.discrete[[2]] <- unique(tbl.discrete[[2]])
tbl.discrete[[3]] <- unique(tbl.discrete[[3]])
plot.discrete2 <- lapply(tbl.discrete[2:3], function(x) {
  ggplot(x, aes(cat.var1, med.var2)) +
    geom_point(size=.6, col= "black") +
    geom_errorbar(aes(ymin=firstQ, ymax=thirdQ), width=.2) +
    facet_grid(rows = vars(species)) +
    xlab(as.character(unique(str_split_fixed(x$var, "\\-", 2)[,1]))) +
    ylab(as.character(unique(str_split_fixed(x$var, "\\-", 2)[,2]))) +
    theme_bw() +
    theme.plot()
})

plot.discrete2
```

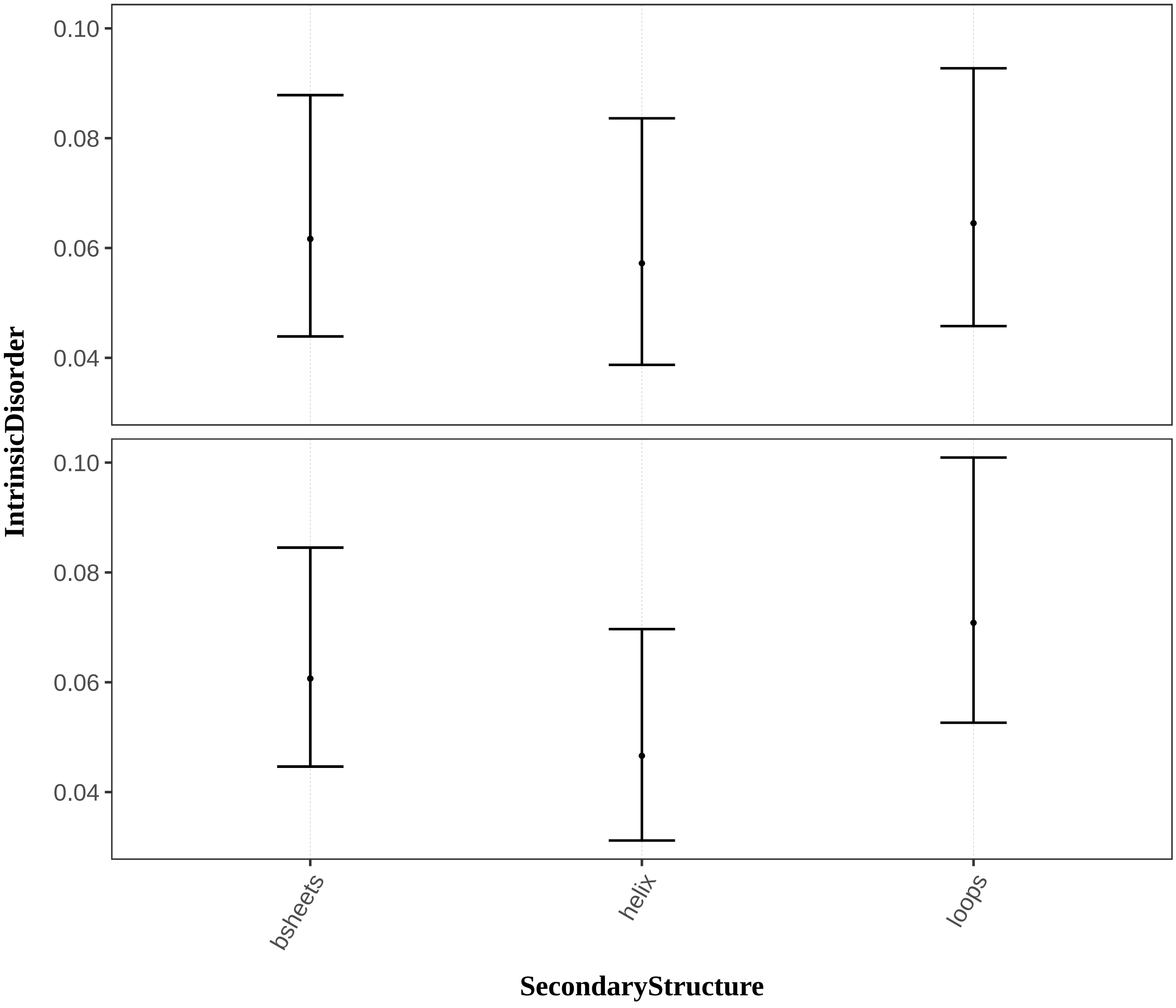

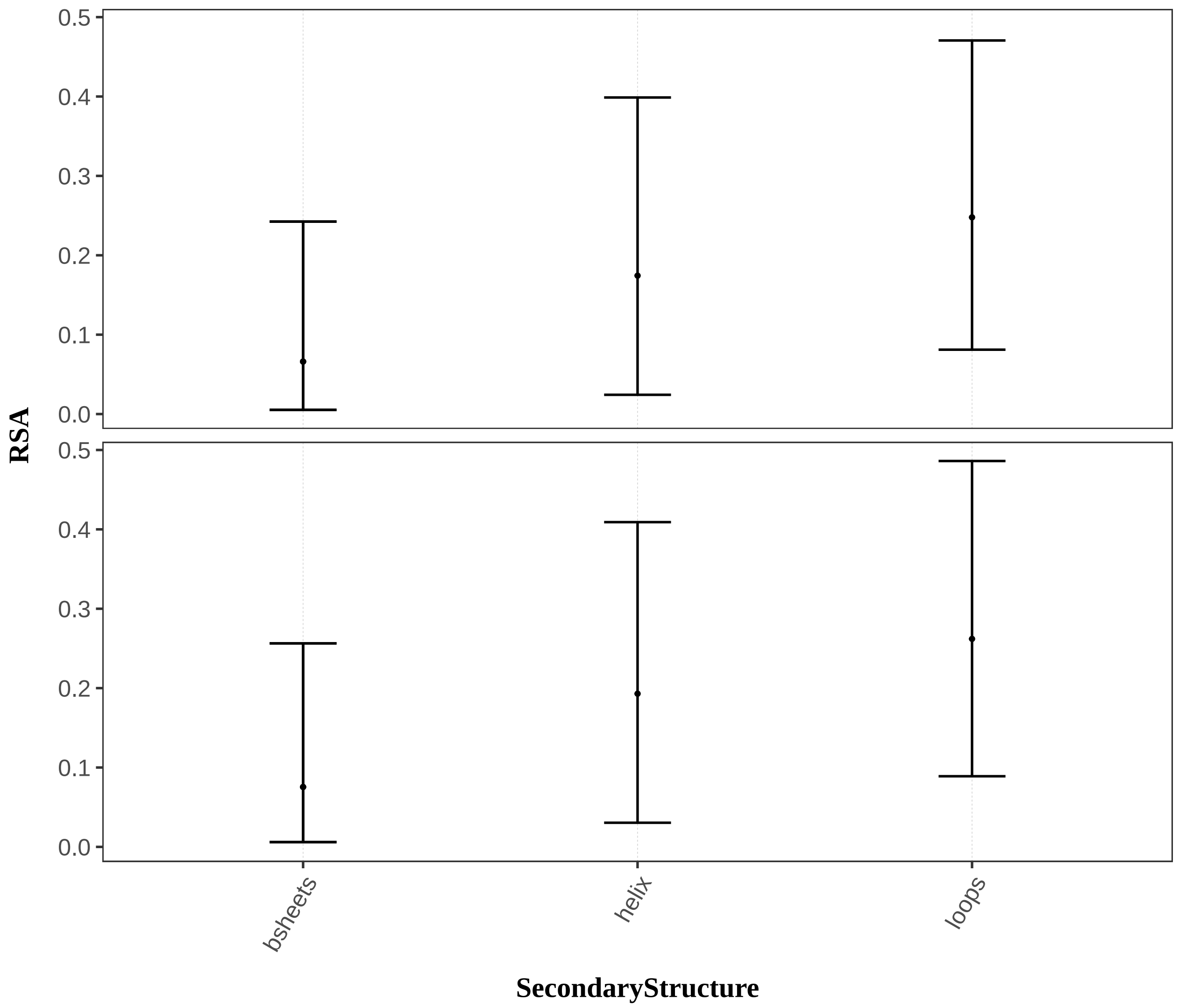


```r
# protein function/RSA
# Arabidopsis
tbl.arab <- subset(tbl.discrete[[1]], tbl.discrete[[1]]$species == "Arabidopsis")
plot.arab <- ggplot(tbl.arab, aes(x = cat.var1, y = med.var2)) +
  geom_point(size=0.5, col = "black") +
  geom_errorbar(aes(ymin=firstQ, ymax=thirdQ), width=.2) +
  ylab("Relative Solvent Accessibility") +
  xlab("Protein Functional Class") +
  scale_fill_grey() +
  scale_color_grey() +
  theme_bw() +
  theme.plot() +
  coord_flip()

plot.arab
```

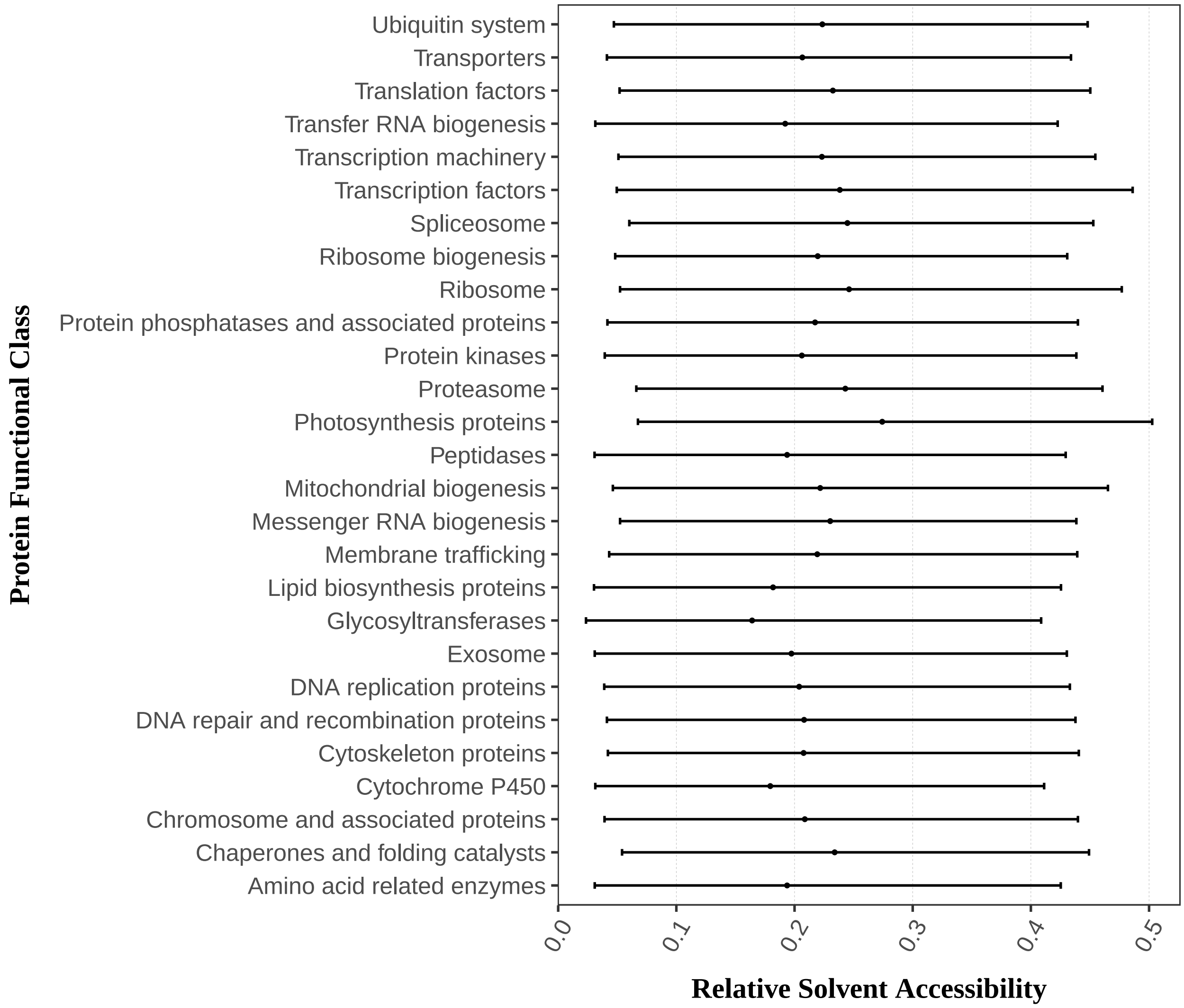


```r
# Drosophila
tbl.dmel <- subset(tbl.discrete[[1]], tbl.discrete[[1]]$species == "Drosophila")
plot.dmel <- ggplot(tbl.dmel, aes(x = cat.var1, y = med.var2)) +
  geom_point(size=0.5, col = "black") +
  geom_errorbar(aes(ymin=firstQ, ymax=thirdQ), width=.2) +
  ylab("Relative Solvent Accessibility") +
  xlab("Protein Functional Class") +
  scale_fill_grey() +
  scale_color_grey() +
  theme_bw() +
  theme.plot() +
  coord_flip()

plot.dmel
```

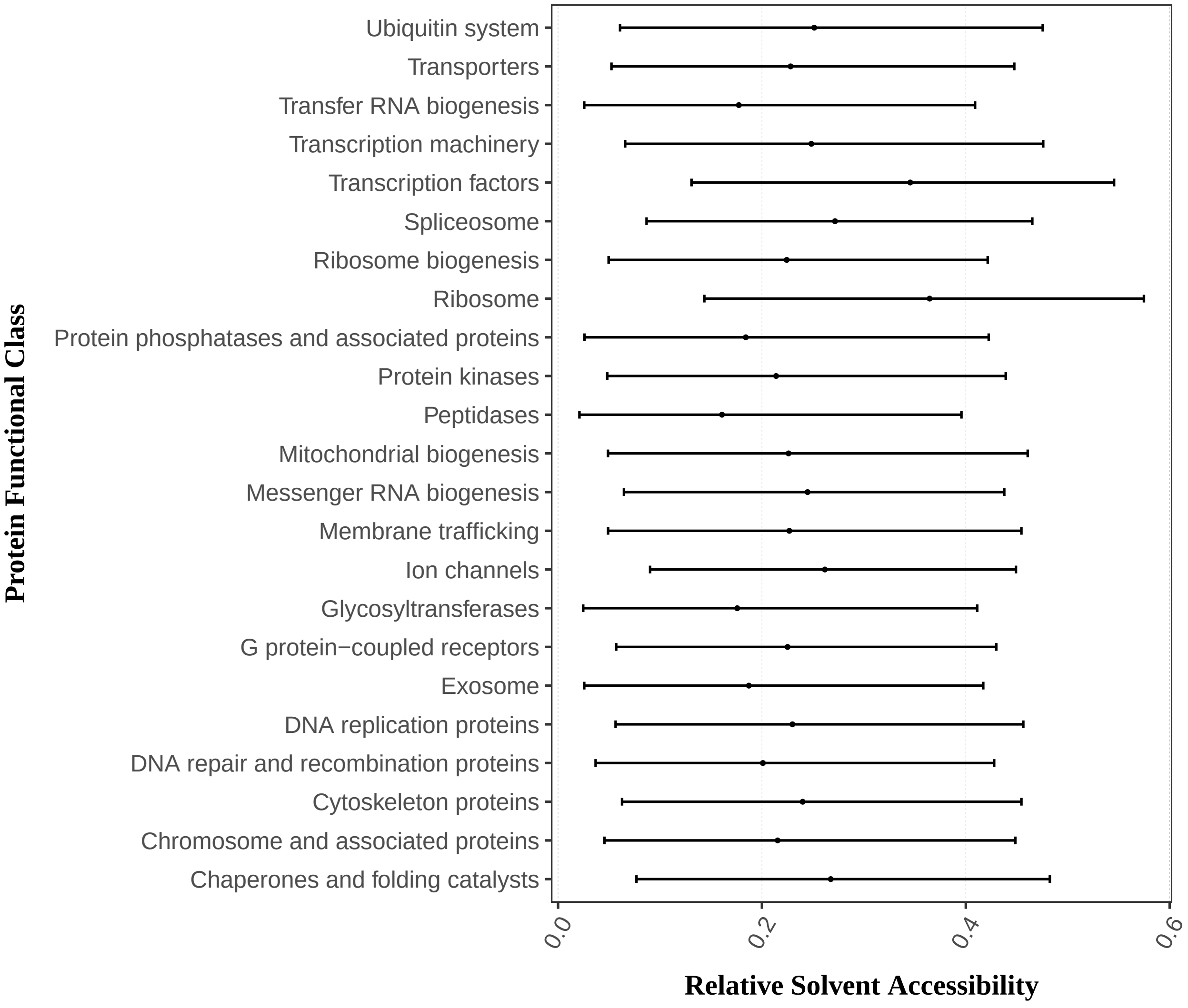
Protein Functional Class
Ubiquitin system
Transporters
Transfer RNA biogenesis
Transcription machinery
Transcription factors
Spliceosome
Ribosome biogenesis
Ribosome
Protein phosphatases and associated proteins
Protein kinases
Peptidases
Mitochondrial biogenesis
Messenger RNA biogenesis
Membrane trafficking
Ion channels
Glycosyltransferases
G protein−coupled receptors
Exosome
DNA replication proteins
DNA repair and recombination proteins
Cytoskeleton proteins
Chromosome and associated proteins
Chaperones and folding catalysts
0.0
0.2
0.4
0.6
Relative Solvent Accessibility
